# Assessment of Dentist Participation in Public Insurance Programs for Children in the US

**DOI:** 10.1001/jamanetworkopen.2022.21444

**Published:** 2022-07-11

**Authors:** Nicoleta Serban, Annalea Anderson, Grace Oberst, Neel Edupuganti, Rohit Ramachandran, Shalini R. Solipuram, Tina Lu

**Affiliations:** 1 Georgia Institute of Technology, H. Milton Stewart School of Industrial and Systems Engineering, Atlanta

## Abstract

**Importance:**

Evaluating the availability of dentists to provide dental care services to children is important for identifying interventions for improving access.

**Objective:**

To assess dental care availability for children in the US by public insurance participation, rural-urban setting, and dentist taxonomy (general, pediatric, or specialized).

**Design, Setting, and Participants:**

This cross-sectional study analyzed the availability of dentists from matching 3 data sets: the 2020 National Plan and Provider Enumeration System, the 2019-2020 State Board of Dentistry information acquired from each state, and the 2019 InsureKidsNow.org database. Data on active dentists in most states (including the District of Columbia [combined hereinafter with states] and excluding Hawaii and Washington) were included in the analysis. The study was conducted from January 2019 to March 2022.

**Main Outcomes and Measures:**

The number and percentage of dentists participating in public insurance programs (Medicaid and/or Children’s Health Insurance Program [CHIP]) were aggregated at the dental office and stratified by the rurality of their practice and taxonomy. State-level comparisons were derived between this study and reports from the Health Policy Institute of the American Dental Association, along with maps and summary statistics disseminated through a data portal and state reports.

**Results:**

Among 204 279 active dentists, participation in public insurance varied widely across states, especially for the states that manage the Medicaid and CHIP programs separately. Participation rates in Medicaid and CHIP varied substantially from those of the Health Policy Institute of the American Dental Association. Participation in Medicaid and CHIP was lowest among urban dentists (Medicaid, 26%; CHIP, 29%) and highest among rural dentists (Medicaid, 39%; CHIP, 40%), while urban dentists accounted for most of the dentist population (urban, 84%; rural, 5%). Similarly, participation in Medicaid and CHIP was substantially lower among general dentists (Medicaid, 28%; CHIP, 29%) vs pediatric dentists (57% in both programs), while each state’s dentist population consisted of notably more general (84%) than pediatric (3%) dentists. Nearly half of the states revealed wide variations in Medicaid and CHIP participation between counties, ranging from no participation (21 states) to full participation (22 states).

**Conclusions and Relevance:**

The findings of this study suggest that disparities in the availability of dentists for pediatric dental care are extensive, particularly for Medicaid- and CHIP-insured children, those living in rural communities, and those receiving specialized care. Lack of dentist availability for Medicaid- and CHIP-insured children appears to deter access to receiving dental care.

## Key Points

**Question:**

Are there disparities in dentist availability for delivering dental care to children within or between states?

**Findings:**

In this cross-sectional study of 204 279 active dentists across the US, dentist participation in public insurance programs varied substantially across states, especially for states that manage the Medicaid program and Children’s Health Insurance Program (CHIP) separately and within states. Dentist participation was lowest among dentists with urban practices and highest among those with rural practices, and notably lower among general dentists vs pediatric dentists.

**Meaning:**

The findings of this study suggest that limited availability of pediatric dental care services remains a challenge in the US, especially for Medicaid- and CHIP-insured children, those living in rural communities, and those receiving specialized care.

## Teaser

This cross-sectional study examines the availability of dental care for children throughout the US through the Medicaid and CHIP programs.

## Introduction

The availability of dentists varies widely in the US,^1^ with the rate of dentists per 100 000 people between 41.8 (Alabama) and 82.7 (Massachusetts) in 2019.^2^ The Health Policy Institute of the American Dental Association (HPI-ADA) published state reports on dentist participation in public insurance programs^1,3^ and patient geographic access to dental care.^4^ The HPI-ADA reports used limited data, not differentiating by rurality vs urbanicity of the dental offices, accounting for the dentist’s taxonomy (general, pediatric, or specialized), or differentiating between public insurance programs.

Evaluating the distribution of dentists by rurality vs urbanicity is important in targeting specific interventions for increasing the availability of dental care in rural communities, which are known to have limited access to care.^5^ Dentists are needed to provide both preventive and specialized care; hence, it would be useful to evaluate the availability of all dental taxonomies, particularly when considering participation in public insurance programs.

Public insurance for children includes Medicaid and the Children’s Health Insurance Program (CHIP), covering children from low-income families, with Medicaid also covering children with disability or in foster care. Medicaid has a larger enrollee population, and generally, higher minimum comprehensive coverage requirements than CHIP.^6^ Each state decides on whether to manage Medicaid and CHIP separately, under Medicaid expansion CHIP, or through a combination of the 2 methods.^7^ When the 2 insurance programs are not managed under a single program or administrator, states may apply differential fee schedules and policies for the 2 programs. These different policies in managing public insurance programs could result in variations in the dental care availability for Medicaid- and CHIP-insured children.

This study expands on existing research on the availability of dental care for children by reporting the distribution of the US dentist population characterized by taxonomy, urbanicity vs rurality of dental practices, and participation in public insurance programs. We present our analysis for 48 states and the District of Columbia (referred to in analysis hereinafter as a state); Hawaii and Washington were excluded because their Boards of Dentistry did not approve release of the dentistry licensure data. The summarized results of this article are available with a web data portal, providing outcome data on the availability of dental care for children for each state and maps by rurality/urbanicity and taxonomy, further presented in individual state reports. The outcome data are also summarized in state reports available in the data portal and in eFigure 2 in the Supplement.

## Methods

### Study Population and Data Sources

The study population consisted of the dentist population stratified by state, taxonomy, rurality vs urbanicity of their practice, and participation in public insurance programs. The 3 data sets used in this study were from the Boards of Dentistry (BOD), the National Plan and Provider Enumeration System (NPPES), and InsureKidsNow.gov (IKN) (eTable 1 in the Supplement).

The BOD data set was acquired from each state and provides information on all licensed dentists, regardless of whether they are in private practice or safety-net care settings. We considered only active-licensed dentists.

The NPPES database comprises all dentists who have applied for a National Provider Index.^8^ A National Provider Index is required by the federal Health Insurance Portability and Accountability Act for electronic transactions. The data provide each dentist’s name, address, and taxonomy classified into general dentistry, pediatric dentistry, or specialist. The data were cross-referenced with BOD to derive the taxonomy.

InsureKidsNow.gov includes all dentists who report their participation in Medicaid and/or CHIP.^9^ This database provides each dentist’s name or that of their practice, address, and taxonomy. We used the data to derive the dentists’ participation in Medicaid and/or CHIP and to cross-reference with BOD, differentiating by taxonomy. We assumed dentists included in the IKN data set participated in public insurance.

Institutional review board approval was not applicable because all data sources are publicly available and do not include health-protected information. The study followed the Strengthening the Reporting of Observational Studies in Epidemiology (STROBE) reporting guideline for cross-sectional studies. The study was conducted from January 2019 to March 2022.

### Matching and Classification

The NPPES and IKN databases provided consistent information for all states, and the BOD data presented differences in the data content across states. The data-matching process consisted of multiple steps, including NPPES and IKN processing and matching, providing an NPPES-IKN data merge, and BOD and NPPES-IKN matching, with average match rates of 94% for BOD and 79% for NPPES-IKN. Data matching was by first and last names. The addresses of the practice sites listed in IKN were used for dentists participating in Medicaid or CHIP. Match rate was not affected by addresses but potentially by name variations (eAppendix in the Supplement).

The dentist-level characteristics considered were (1) taxonomy (general, pediatric, and specialist), (2) rurality vs urbanicity (rural, suburban, and urban), and (3) public insurance participation (Medicaid, CHIP, and both). The rurality vs urbanicity classification was derived using the rural-urban commuting area (RUCA) System,^10^ with further grouping into urban (RUCAs 1-3), suburban (RUCAs 4-6), and rural (RUCAs 7-10) areas.

Because the IKN database provides data on the addresses of the participating dentist practices, we derived the portion of time spent by taxonomy at each practice address. For dentists with multiple practice offices, we assumed an equal amount of time spent across the practice offices; hence, only a proportion of a dentist’s caseload was allocated to 1 office location (eAppendix in the Supplement).

### Outcome Measures

The outcome measures included (1) number and percentage of dentists; (2) number and percentage of dentists participating in Medicaid, CHIP, or Medicaid and CHIP; and (3) number and percentage of dentists participating in public insurance (Medicaid or CHIP). The measures were aggregated at the dental office location, then stratified by rurality/urbanicity and taxonomy, and reported by state.

### Data Analysis

To perform the analysis, Python, version 3.7.4 (Python Software Foundation) was used with the following packages: pandas, numpy, matplotlib, altair, and geopandas. In addition, R, version 4.0.2 (R Foundation for Statistical Computing) was used.

## Results

eTable 1 in the Supplement presents the availability of various attributes of BOD. eTable 2 in the Supplement provides state-level statistics on the number of BOD-IKN–matched dentists. eTable 3 in the Supplement reports state-level percentages of dentists stratified by rurality/urbanicity and taxonomy. eFigure 1 in the Supplement presents dental participation rates in Medicaid, CHIP, and HPI-ADA by county. Demographic characteristics were not available.

Of 204 279 active dentists, the number of dentists per 100 000 people ratio varied widely, for example, 41 in South Dakota, 89 in California, and 111 in the District of Columbia. Across all states, most dentists practiced in urban communities (median, 84% [IQR, 23%]), followed by suburban (median, 11% [IQR, 11%]) and rural (median, 5% [IQR, 10%]) communities. Most were general dentists (median, 84% [IQR, 4%]), followed by specialists (median, 13% [IQR, 3%]) and pediatric dentists (median, 3% [IQR, 1%]). General dentists were noted in as much as 90% of a state’s dentist population, whereas pediatric dentists were found in as little as 1% of the population.

Table 1 provides dentist participation in Medicaid and/or CHIP, compared with the HPI-ADA public insurance participation rates.^1,3^ According to IKN, 20 states (eg, Arizona and Georgia) had the same participation rates for Medicaid and CHIP, meaning that dentists reported in the IKN database participated in both programs. Moreover, 8 states (eg, Maryland and South Carolina) showed participation in CHIP or Medicaid only, meaning that there were dentists participating in only 1 program; however, according to federal law, Medicaid and CHIP are required to provide dental benefits; hence, we assumed the same participation rates for the 2 programs. We considered cases with these assumptions as expanded Medicaid and noted the same participation rates for the 2 programs even though they had different management practices. The remaining 21 states had differences in Medicaid and CHIP participation rates, with some as high as 70%. For some states, there was a large gap in Medicaid vs CHIP participation (eg, Alabama, 5% vs 75%; Iowa, 2% vs 51%; and New Mexico, 42% vs 6%). Nine states had a higher participation rate in CHIP, with the largest difference in Alabama (70%). The remaining 12 states had a higher participation rate in Medicaid, with the largest difference in New Mexico (36%).

**Table 1.** Total Number of Dentists and Participation of Dentists in Public Insurance Programs^a^

State	Total No. of dentists	Percentage of dentists					
		Medicaid	CHIP	Medicaid and CHIP	Public insurance	Public insurance^b^	
						ADA 2018	ADA 2015
Alabama	2145	5	75	10	80	75	74
Alaska^c^	592	64	64	64	64	73	43
Arizona^d^	4178	29	29	29	29	28	28
Arkansas^d^	1354	49	49	49	49	NA	64
California^d^	35 126	11	11	11	11	29	16
Colorado	4199	21	43	29	64	57	60
Connecticut^d^	2847	40	40	40	40	45	47
Delaware^d^	418	63	63	63	63	66	59
District of Columbia^c^	766	25	25	25	25	NA	31
Florida	11 871	11	15	16	26	35	30
Georgia^d^	5139	23	23	23	23	25	28
Idaho^d^	1048	42	42	42	42	46	38
Illinois	9242	14	6	15	20	24	26
Indiana^d^	3541	40	40	40	40	NA	46
Iowa	1807	2	51	3	53	89	86
Kansas	1534	11	16	0	27	40	24
Kentucky^d^	2506	28	28	28	28	48	28
Louisiana	2269	28	12	12	39	41	41
Maine^d^	728	34	34	34	34	16	15
Maryland^c^	4308	30	30	30	30	28	28
Massachusetts^d^	6019	32	32	32	32	51	38
Michigan	6942	20	44	32	64	71	73
Minnesota^d^	3083	60	60	60	60	NA	64
Mississippi	1299	27	39	51	66	64	65
Missouri^d^	3176	19	19	19	19	32	24
Montana^d^	654	57	57	57	57	77	77
Nebraska^d^	1266	27	27	27	27	NA	51
Nevada	1708	21	17	24	38	NA	41
New Hampshire^c^	1079	6	6	6	6	13	16
New Jersey	8026	17	9	21	26	30	27
New Mexico	1070	42	6	18	49	60	52
New York	13 199	19	17	35	36	40	30
North Carolina	5454	20	3	7	23	36	30
North Dakota	558	24	23	45	47	72	74
Ohio^c^	6282	31	31	31	31	29	34
Oklahoma^d^	2012	44	44	44	44	47	48
Oregon^d^	2859	35	35	35	35	43	39
Pennsylvania	7941	10	44	19	54	NA	68
Rhode Island	579	35	1	1	36	25	20
South Carolina^c^	2685	38	38	38	38	NA	46
South Dakota^d^	367	42	42	42	42	NA	67
Tennessee	3000	14	12	1	26	30	30
Texas	15 981	18	23	30	41	57	50
Utah	2343	23	15	25	39	56	60
Vermont^c^	417	47	47	47	47	71	73
Virginia^d^	5708	27	27	27	27	29	30
West Virginia	850	34	30	57	64	NA	61
Wisconsin^d^	3761	29	29	29	29	40	34
Wyoming^c^	343	63	63	63	63	71	69

Abbreviations: ADA, American Dental Association; CHIP, Children’s Health Insurance Program; NA, not applicable.

^a^ Data stratified by participation in Medicaid, CHIP, Medicaid and CHIP (intersection of the 2), and Medicaid or CHIP (union of the 2). For some states, the data are the same because those states administer CHIP and Medicaid jointly.

^b^ Participation in public insurance derived by the Health Policy Institute of the ADA in 2018 (based on 2017 data) and 2015 (based on 2014 or earlier data) reports for comparison.

^c^ The state operates CHIP as an expansion of the state’s Medicaid program (ie, CHIP Medicaid expansion).

^d^ The state manages CHIP and Medicaid dental services under a single administration or other form of joint management.

We report the public insurance (Medicaid or CHIP) rates to compare with those published by HPI-ADA. The rates in our study were a mean (SD) of 40% (16%) and a median of 38% (IQR, 22%), which are slightly lower than the 2018 HPI-ADA rates (mean [SD], 46% [19%]; median, 43% [IQR, 33%])^3^ and the 2015 HPI-ADA rates (mean [SD], 45% [19%]; median, 41% [IQR, 31%]).^1^ Of 39 states, 8 (21%) had higher participation rates noted in our study than the 2018 HPI-ADA–reported rates (9 states and the District of Columbia were excluded from the 2018 HPI-ADA–reported rates); of 49 states included in the present analysis, 13 (27%) had higher participation rates than the 2015 HPI-ADA reported rates, and 1 state (Kentucky) displayed the same participation rate. The largest difference between the rates in our study and the rates reported by both HPI-ADA studies was for Iowa (53% in this study vs 89% in the 2018 HPI-ADA study and 86% in the 2015 HPI-ADA study).

Table 2 presents dentist participation in Medicaid or CHIP, stratified by rurality for each state. The greatest participation was found among rural dentists, and the lowest rate was among urban dentists. The median Medicaid rates were 39% (IQR, 29%) for rural dentists, 32% (IQR, 30%) for suburban dentists, and 26% (IQR, 16%) for urban dentists. The median CHIP rates were 40% (IQR, 30%) for rural dentists, 36% (IQR, 34%) for suburban dentists, and 29% (IQR, 22%) for urban dentists. Alabama presented the greatest differences in Medicaid vs CHIP participation among urban dentists (69%), suburban dentists (73%), and rural dentists (71%). Medicaid and CHIP participation reached as high as 82% for rural dentists in South Carolina vs the maximum of 68% among urban dentists in Wyoming. New Hampshire had the lowest participation in the 2 programs among urban (6%) and suburban (5%) dentists. California had the lowest participation among suburban (5%) and rural (3%) dentists, excluding Rhode Island, which had only 1 rural dentist.

**Table 2.** Total Number of Dentists and Participation in Public Insurance Programs by Rurality and Urbanicity^a^

State	Total No. of dentists	Percentage of dentists					
		Urban Medicaid	Urban CHIP	Suburban Medicaid	Suburban CHIP	Rural Medicaid	Rural CHIP
Alabama	2145	5	74	7	80	9	80
Alaska^b^	592	58	58	74	74	71	71
Arizona^c^	4178	29	29	32	32	40	40
Arkansas^c^	1354	45	45	55	55	66	66
California^c^	35 126	11	11	5	5	3	3
Colorado	4199	21	42	21	51	19	45
Connecticut^c^	2847	39	39	54	54	26	26
Delaware^c^	418	64	64	54	54	64	64
District of Columbia^b^	766	25	25	NA	NA	NA	NA
Florida	11 871	11	15	11	11	24	25
Georgia^c^	5139	22	22	26	26	39	39
Idaho^c^	1048	38	38	53	53	42	42
Illinois	9242	15	6	8	5	4	4
Indiana^c^	3541	38	38	51	51	50	50
Iowa	1807	2	45	1	56	3	68
Kansas	1534	9	13	15	24	18	33
Kentucky^c^	2506	20	20	36	36	59	59
Louisiana	2269	26	11	36	17	59	10
Maine^c^	728	29	29	39	39	41	41
Maryland^b^	4308	30	30	19	19	48	48
Massachusetts^c^	6019	32	32	40	40	21	21
Michigan	6942	20	45	19	42	23	46
Minnesota^c^	3083	57	57	72	73	67	67
Mississippi	1299	22	37	29	41	38	44
Missouri^c^	3176	17	17	27	27	40	40
Montana^c^	654	57	57	56	56	61	61
Nebraska^c^	1266	21	21	36	36	45	45
Nevada	1708	22	17	18	17	16	10
New Hampshire^b^	1079	6	6	5	5	11	11
New Jersey	8026	17	9	16	8	NA	NA
New Mexico	1070	40	6	50	6	49	8
New York	13 199	19	17	20	17	21	20
North Carolina	5454	19	3	27	5	30	7
North Dakota	558	22	22	26	24	29	25
Ohio^b^	6282	30	30	40	40	44	44
Oklahoma^c^	2012	38	38	55	55	65	65
Oregon^c^	2859	35	35	37	37	37	37
Pennsylvania	7941	9	43	10	52	16	53
Rhode Island	579	35	1	NA	NA	0	0
South Carolina^b^	2685	35	35	51	51	82	82
South Dakota^c^	367	34	34	49	49	49	49
Tennessee	3000	12	11	24	15	22	12
Texas	15 981	18	23	20	24	14	19
Utah	2343	22	15	30	15	36	24
Vermont^b^	417	44	44	45	45	51	51
Virginia^c^	5708	26	26	34	34	40	40
West Virginia	850	32	29	34	31	40	39
Wisconsin^c^	3761	27	27	30	30	39	39
Wyoming^b^	343	68	68	64	64	60	60

Abbreviation: CHIP, Children’s Health Insurance Program; NA, not applicable.

^a^ For some states, the data are the same because those states administer CHIP and Medicaid jointly.

^b^ The state operates CHIP as an expansion of the state’s Medicaid program (ie, CHIP Medicaid expansion).

^c^ The state manages CHIP and Medicaid dental services under a single administration or other form of joint management.

Table 3 reports the dentists accepting Medicaid or CHIP stratified by taxonomy for each state. When comparing public insurance participation by taxonomy, the highest median participation rates in Medicaid and CHIP were among pediatric dentists (Medicaid, 57% [IQR, 39%]; CHIP, 57% [IQR, 34%]), followed by general dentists (Medicaid, 28% [IQR, 20%]; CHIP, 29% [IQR, 28%]) and specialists (Medicaid, 25% [IQR, 17%]; CHIP, 24% [IQR, 26%]). Medicaid and CHIP participation reached as high as 94% for pediatric dentists (Alaska), yet only 65% for general dentists (Wyoming). Alabama and Rhode Island presented the largest differences in Medicaid vs CHIP participation among general dentists (73%), specialists (57%), and pediatric dentists (92%). New Hampshire showed the lowest participation in the 2 programs among general dentists (6%) and specialists (7%), and California had the lowest participation among pediatric dentists (11%).

**Table 3.** Total Number of Dentists and Participation in Public Insurance Programs by Taxonomy^a^

State	Total No. of dentists	Percentage of dentists					
		General Medicaid	General CHIP	Pediatric Medicaid	Pediatric CHIP	Specialist Medicaid	Specialist CHIP
Alabama	2145	5	78	16	68	4	61
Alaska^b^	592	63	63	94	94	63	63
Arizona^c^	4178	27	27	75	75	31	31
Arkansas^c^	1354	47	47	91	91	56	56
California^c^	35 126	11	11	11	11	8	8
Colorado	4199	19	47	36	48	30	20
Connecticut^c^	2847	37	37	83	83	40	40
Delaware^c^	418	62	62	79	79	65	65
District of Columbia^b^	766	26	26	42	42	14	14
Florida	11 871	10	14	27	36	9	15
Georgia^c^	5139	22	22	64	64	19	19
Idaho^c^	1048	41	41	80	80	32	32
Illinois	9242	15	6	14	9	10	5
Indiana^c^	3541	40	40	61	61	34	34
Iowa	1807	2	51	4	71	2	48
Kansas	1534	10	16	26	37	15	13
Kentucky^c^	2506	28	28	57	57	24	24
Louisiana	2269	28	12	52	25	19	9
Maine^c^	728	31	31	57	57	43	43
Maryland^b^	4308	29	29	65	65	28	28
Massachusetts^c^	6019	32	32	61	61	24	24
Michigan	6942	20	47	36	41	16	20
Minnesota^c^	3083	60	60	81	83	57	57
Mississippi	1299	26	40	43	47	26	33
Missouri^c^	3176	19	19	40	40	19	19
Montana^c^	654	60	60	68	68	40	40
Nebraska^c^	1266	28	28	54	54	10	10
Nevada	1708	20	16	48	38	23	22
New Hampshire^b^	1079	6	6	19	19	7	7
New Jersey	8026	16	9	32	18	17	10
New Mexico	1070	42	6	80	12	39	7
New York	13 199	18	17	35	33	17	14
North Carolina	5454	18	3	62	8	20	3
North Dakota	558	24	22	36	36	25	24
Ohio^b^	6282	32	32	71	71	20	20
Oklahoma^c^	2012	44	44	60	60	44	44
Oregon^c^	2859	34	34	65	65	33	33
Pennsylvania	7941	9	44	26	48	9	42
Rhode Island	579	34	1	92	0	27	0
South Carolina^b^	2685	38	38	75	75	29	29
South Dakota^c^	367	39	39	83	83	48	48
Tennessee	3000	12	10	41	34	24	14
Texas	15 981	18	23	32	44	10	19
Utah	2343	22	14	52	37	22	16
Vermont^b^	417	44	44	77	77	56	56
Virginia^c^	5708	25	25	74	74	30	30
West Virginia	850	34	31	42	42	26	26
Wisconsin^c^	3761	28	28	70	70	28	28
Wyoming^b^	343	65	65	89	89	41	41

Abbreviations: ADA, American Dental Association; CHIP, Children’s Health Insurance Program.

^a^ For some states, the data are the same because those states administer CHIP and Medicaid jointly.

^b^ The state operates CHIP as an expansion of the state’s Medicaid program (ie, CHIP Medicaid expansion).

^c^ The state manages CHIP and Medicaid dental services under a single administration or other form of joint management.

Table 4 presents summaries, including minimum, median, and maximum, for the Medicaid and CHIP county-level participation rates for each state. County-level differences were calculated as the state’s difference between maximum and minimum county-level participation rate. Nearly half of the states ranged from no participation to full participation between counties (0% to 100%). We observed the largest difference in public insurance participation between county-level (median, 96% [IQR, 35%]) vs state-level (64%) participation in Alaska.

**Table 4.** Participation of Dentists in Public Insurance Programs by County^a^

State	Percentage of dentists							
	Minimum Medicaid	Maximum Medicaid	Median Medicaid	Minimum CHIP	Maximum CHIP	Median CHIP	State Medicaid^b^	State CHIP^b^
Alabama	0	50	4	50	100	78	5	75
Alaska^c^	7	100	96	7	100	96	64	64
Arizona^d^	4	100	31	4	100	31	29	29
Arkansas^d^	0	100	59	0	100	59	49	49
California^d^	0	22	4	0	22	4	11	11
Colorado	0	85	19	0	100	48	21	43
Connecticut^d^	32	58	42	32	58	42	40	40
Delaware^d^	58	65	58	58	65	58	63	63
District of Columbia^c^	25	25	25	25	25	25	25	25
Florida	2	70	11	3	53	15	11	15
Georgia^d^	0	100	30	0	100	30	23	23
Idaho^d^	0	100	46	0	100	46	42	42
Illinois	0	62	4	0	50	1	14	6
Indiana^d^	0	100	42	0	100	42	40	40
Iowa	0	35	0	0	100	62	2	51
Kansas	0	100	7	0	100	25	11	16
Kentucky^d^	0	100	45	0	100	45	28	28
Louisiana	0	100	37	0	100	9	28	12
Maine^d^	18	57	40	18	57	40	34	34
Maryland^c^	9	78	33	9	78	33	30	30
Massachusetts^d^	4	48	28	4	48	28	32	32
Michigan	0	76	17	0	100	47	20	44
Minnesota^d^	0	100	66	0	100	66	60	60
Mississippi	6	67	38	6	78	45	27	39
Missouri^d^	0	100	33	0	100	33	19	19
Montana^d^	0	100	57	0	100	57	57	57
Nebraska^d^	0	100	33	0	100	33	27	27
Nevada	0	100	20	0	100	16	21	17
New Hampshire^c^	3	14	8	3	14	8	6	6
New Jersey	6	31	19	3	19	9	17	9
New Mexico	10	100	48	0	50	6	42	6
New York	0	56	20	2	43	20	19	17
North Carolina	0	100	27	0	29	4	20	3
North Dakota	0	50	25	0	50	24	24	23
Ohio^c^	0	100	36	0	100	36	31	31
Oklahoma^d^	0	100	55	0	100	55	44	44
Oregon^d^	0	100	34	0	100	34	35	35
Pennsylvania	2	52	10	21	83	46	10	44
Rhode Island	23	40	31	0	1	0	35	1
South Carolina^c^	17	100	52	17	100	52	38	38
South Dakota^d^	0	100	50	0	100	50	42	42
Tennessee	0	100	19	0	60	12	14	12
Texas	0	100	14	0	100	20	18	23
Utah	5	100	27	0	54	18	23	15
Vermont^c^	17	100	51	17	100	51	47	47
Virginia^d^	0	100	29	0	100	29	27	27
West Virginia	16	94	38	6	50	35	34	30
Wisconsin^d^	0	100	31	0	100	31	29	29
Wyoming^c^	14	100	68	14	100	68	63	63

Abbreviation: CHIP, Children’s Health Insurance Program.

^a^ For some states, the data are the same because those states administer CHIP and Medicaid jointly.

^b^ State-level participation rates provided for reference.

^c^ The state operates CHIP as an expansion of the state’s Medicaid program (ie, CHIP Medicaid expansion).

^d^ The state manages CHIP and Medicaid dental services under a single administration or other form of joint management.

## Discussion

This cross-sectional study analyzed dentist availability for pediatric dental care and their participation in public insurance programs. The analysis allowed for comparisons across states with inferences on variations by rurality vs urbanicity and taxonomy. Our overall findings suggest the need for more detailed analysis on dentist availability in delivering dental care to children.

There was a wide variation in dentist availability in rural communities, with many states having almost no rural dentists and few having a similar share of dentists across the 3 urbanicity-rurality levels. These differences were also subject to the rurality vs urbanicity status of each state; for example, Vermont had a higher percentage of dentists with rural practices (41%) and is a mostly rural state (60% of total communities).^11^ Overall, the percentage of dentists practicing in rural communities was low compared with the level of the state’s rurality status, for example, only 3% of the dentists practiced in rural communities in Georgia, and 40% of the state is considered rural.^11^ This finding suggests that the supply of dental care for children was insufficient in rural communities during the study period; interventions such as loan forgiveness programs^12,13^ or school-based mobile care may help address such shortages.^14,15,16^

The primary focus of this study was to derive dentist participation in CHIP or Medicaid programs. Only one-third of the states managed Medicaid and CHIP separately. For states with CHIP and Medicaid joint dental care delivery, the management of the 2 programs followed different practices, for example, management under a single administrator but separate enrollment (eg, Georgia^17^ and Virginia^18^), CHIP Medicaid expansion (eg, Alaska^7^), or a public program covering children and adults under Medicaid expansion (eg, California). For the remaining states with separate Medicaid and CHIP participation rates reported by the IKN data, the difference in the participation rates for the 2 programs varied widely, suggesting that policies instating a single administrator for the 2 programs in those states could result in higher participation of dentists in public insurance programs, because burdensome administrative requirements are one of the most common reasons for dentists not participating in public insurance programs.^19^

The difference in CHIP and Medicaid participation was also reflected in differences between the rates reported in this study vs those reported by the HPI-ADA.^1,3^ Because the HPI-ADA reports overall public insurance (Medicaid or CHIP) participation, we computed those rates for comparison. There were differences in the reported rates, with both higher and lower HPI-ADA rates vs those reported in our study. Some states had large differences in the reported rates, for example, Iowa (53% in this study vs 89% in the 2018 HPI-ADA database). Alabama had the greatest difference in Medicaid and CHIP participation (5% vs 75%), with the 2018 HPI-ADA report presenting only the overall rate (75%). These differences could be because of different data sets and approaches used. The 2015 HPI-ADA analysis^1^ used a similar approach as in this study with 2014 data, while the 2018 HPI-ADA analysis^3^ used the 2017 Transformed Medicaid Statistical Information System data instead of the 2018 IKN data set; IKN is used for finding participating dentists by those seeking dental care, and so is a pertinent source for identifying participation in public insurance programs. The 2018 HPI-ADA analysis^3^ was not complete, with data on only 41 states reported.

Although a low percentage of dentists practiced in rural communities, the greatest participation in Medicaid and CHIP was for rural dentists, followed by suburban dentists, then urban dentists. When comparing Medicaid vs CHIP rates by rurality and urbanicity, this study found large differences for the 2 programs. This finding suggests wide inconsistency in the availability of dentists across communities and insurance programs, pointing to the need for granular analysis toward informing a targeted policy to improve access.

This study also noted that median participation of pediatric dentists in Medicaid and CHIP was nearly twice as high as that of general and specialist dentists, in contrast to the makeup of dentist populations throughout the states, with pediatric dentists consisting of low percentages (as low as 1% for some states). This low rate suggests that future policies aiming to improve dental care access for children should consider the association between dentists’ taxonomy and participation in Medicaid and CHIP programs, for example, expansion of loan forgiveness programs for pediatric dentists. This difference in participation rates is particularly important because pediatric dentists commonly treat children with challenging conditions, eg, attention deficit/hyperactivity disorder or special needs.

This study also derived county-level dentist availability, with more detailed analysis available in a data portal. Nearly half of the states had counties with no participation as well as with 100% participation, most corresponding to small counties, with some states having very large differences in participation at the county vs state level. These variations may be associated with social and cultural preferences and regulatory and legal environments, possibly affecting the geographic distribution of dentists overall. This granular analysis noted wide disparities in the dentist participation in Medicaid and CHIP across communities, suggesting that interventions for increasing the availability need to be targeted to meet the demand at the community level.

### Limitations

This study had limitations. BOD data are primarily intended to serve as contact information; thus, a dentist may use an address of their residence vs their practice. Exceptions are Florida and California, which keep both home and practice addresses in separate columns. Moreover, most states’ BOD databases provided only 1 street address; however, most dentists practice at more than 1 location. Neither the BOD nor National Provider Index listings differentiate between dentists who are retired and no longer have active licenses and those who are retired but still have active licenses. This lack of distinction possibly resulted in overestimation of dentist availability. The IKN data listed each dentist for all the locations that their practice owns, but does not reflect the time a dentist spends in each location. Dentists listed in IKN data, although reported as participating in Medicaid or CHIP, may not necessarily accept children with public insurance; this limitation could result in lower participation rates for Medicaid and/or CHIP.^20^ Moreover, according to an HPI-ADA report,^1^ 4 states allow dentists to opt out of being included in publicly available lists, including Alabama, New Hampshire, North Carolina, and Oklahoma. These limitations could result in participation estimates that are different from the true values. In addition, the comparisons with the HPI-ADA were not for the same years; the more recent HPI-ADA estimates were also incomplete.

## Conclusions

This cross-sectional study noted wide disparities in dentist availability for pediatric dental care, particularly for Medicaid- and CHIP-insured children, for children living in rural communities, and for those receiving specialized care. Because dentist availability varied greatly across states, there is not a single policy or intervention that could reduce these disparities. State-level policies targeting different dimensions of the dental care system could address these disparities, for example, adopting joint administration of CHIP and Medicaid, funding public-health school programs, or expanding loan forgiveness programs.^21,22,23,24^ A complete analysis of the current availability of dental care in all states could provide a starting point on what each state may improve upon by benchmarking to other states.

Because of the wide disparities across communities within each state, local interventions are necessary; however, such interventions would best be targeted for the subpopulations in need of improved dentist availability. Existing measures for addressing the shortage of dentists focused primarily on the number of dentists within a community, discounting participation in public insurance for the child population.^25,26^ Because many decisions on improving dentist availability (eg, loan forgiveness programs) are targeted using shortage areas identified by the Health Resources & Services Administration, it is important that existing measures incorporate more rigorous methods in identifying shortage areas.

Although dental care benefits for children are mandated for Medicaid and CHIP programs,^7^ lack of dentist availability for children with public insurance deters access to such services. This finding aligns with results of a 2008 survey.^22^ Hence, 12 years after the 2008 data, limited availability of dental care for children remains a challenge in most of the US.

## Notes

Supplement. **eTable 1.** Analysis of Each State’s Board of Dentistry Dataset (Including Date of Acquisition and Dentist-Specific Characteristics) **eAppendix.** Data-Matching Algorithm **eTable 2.** BOD-IKN Matched Dentists Unable to Match With a Dentist in the NPPES Database **eTable 3.** Total Number of Dentists and Percentage of Dentists by Rurality/Urbanicity Classification (Urban, Suburban, Rural) and Taxonomy (General, Pediatric, Specialist) for Each State **eFigure 1.** Dentists’ Participation in CHIP, Medicaid, and Public Insurance (Medicaid or CHIP) for Each County **eFigure 2.** Dental Care Availability for Children in the United States: State Report
