# Supplementary material for: Assessment of Dentist Participation in Public Insurance Programs for Children in the US
Source: JAMA Netw Open. 2022 Jul 11;5(7):e2221444. doi: 10.1001/jamanetworkopen.2022.21444 (PMC9274318; doi:10.1001/jamanetworkopen.2022.21444)
Supplement: Supplement. — eTable 1. Analysis of Each State’s Board of Dentistry Dataset (Including Date of Acquisition and Dentist-Specific Characteristics) eAppendix. Data-Matching Algorithm eTable 2. BOD-IKN Matched Dentists Unable to Match With a Dentist in the NPPES Database eTable 3. Total Number of Dentists and Percentage of Dentists by Rurality/Urbanicity Classification (Urban, Suburban, Rural) and Taxonomy (General, Pediatric, Specialist) for Each State eFigure 1. Dentists’ Participation in CHIP, Medicaid, and Public Insurance (Medicaid or CHIP) for Each County eFigure 2. Dental Care Availability for Children in the United States: State Report [file jamanetwopen-e2221444-s001.pdf]

Supplementary Online Content

Serban N, Anderson A, Oberst G, et al. Assessment of dentist participation in public insurance programs for children in the US. *JAMA Netw Open*. 2022;5(7):e2221444. doi:10.1001/jamanetworkopen.2022.21444

eTable 1. Analysis of Each State's Board Of Dentistry Dataset (Including Date of Acquisition and Dentist-Specific Characteristics)

eAppendix. Data-Matching Algorithm

eTable 2. BOD-IKN Matched Dentists Unable to Match With a Dentist in the NPPES Database

eTable 3. Total Number of Dentists and Percentage of Dentists by Rurality/Urbanicity Classification (Urban, Suburban, Rural) and Taxonomy (General, Pediatric, Specialist) for Each State

eFigure 1. Dentists' Participation in CHIP, Medicaid, and Public Insurance (Medicaid or CHIP) for Each County

eFigure 2. Dental Care Availability for Children in the United States: State Report

This supplementary material has been provided by the authors to give readers additional information about their work.

© 2022 Serban N et al. *JAMA Network Open.*

Online-Supplemental Material A: Description of Data

Board of Dentistry (BOD)

Each state's Board of Dentistry (BOD) maintains a roster of dental care providers. Data were retrieved on a state-by-state basis. While rosters may vary in their columns/attributes, we focused on retrieving a standardized set of information from each state: the provider's full name (first, last and if applicable, middle), complete address (street address, city, state, zip code), county, license type and license expiration date. Certain states' BOD datasets had providers with incomplete names, addresses and/or counties. These anomalies were recorded in eTable 1.

©2022 Serban N et al. *JAMA Network Open*.

eTable 1. Analysis of each state's Board of Dentistry Dataset (including Date of Acquisition and Dentist-Specific Characteristics)[a]

State	Date Acquired	First Name	Last Name	Street Address	City	State	Zip	County
**AL**	11/20/2018	C	C	C	C	C	C	I
**AK**	10/8/2018	C	C	C	C	C	C	M
**AZ**	9/14/2019	C	C	C	C	C	C	M
**AR**	7/26/2019	C	I	I	I	I	I	I
**CA**	7/23/2020	I	C	C	C	C	C	C
**CO**	10/8/2018	C	C	C	C	C	C	I
**CT**	4/3/2019	C	C	I	I	C	C	I
**DC**	7/20/2020	C	C	I	C	I	C	M
**DE**	9/5/2019	C	C	M	C	C	C	C
**FL**	9/22/2018	C	C	C	C	C	C	M
**GA**	11/12/2019	C	C	C	C	C	C	C
**HI**	M	M	M	M	M	M	M	M
**ID**	9/3/2019	C	C	C	C	C	C	C
**IL**	1/28/2020	C	C	C	C	C	C	C
**IN**	7/25/2019	C	C	C	C	C	C	C
**IA**	7/23/2019	C	C	C	C	C	C	I
**KS**	9/30/2019	C	C	C	C	C	C	M
**KY**	9/4/2019	C	C	I	I	I	I	I
**LA**	2/5/2020	C	C	C	C	C	C	M
**ME**	10/8/2018	C	C	M	C	C	C	M
**MD**	9/10/2019	C	C	C	C	C	C	C
**MA**	10/8/2018	C	C	C	C	C	C	C
**MI**	11/18/2019	C	C	C	C	C	C	M
**MN**	11/18/2019	C	C	C	C	C	C	C
**MS**	10/11/2019	C	C	C	C	C	C	I
**MO**	7/21/2019	C	C	C	C	C	C	C
**MT**	7/25/2019	C	C	C	C	C	C	M
**NE**	7/25/2019	C	C	C	C	C	C	C
**NV**	9/30/2019	C	C	C	C	C	C	M
**NH**	9/25/2019	C	C	C	C	C	C	M
**NJ**	10/8/2018	C	C	C	C	C	C	C
**NM**	8/19/2020	C	C	C	C	C	C	C
**NY**	11/15/2019	C	C	I	C	C	I	C
**NC**	9/24/2019	C	C	C	C	C	C	M
**ND**	9/18/2019	C	C	C	C	C	C	M
**OH**	4/4/2019	C	C	C	C	C	C	M
**OK**	9/25/2019	C	C	C	C	C	C	I
**OR**	9/5/2019	C	C	C	C	C	C	C
**PA**	7/29/2019	C	C	C	C	C	C	C
**RI**	9/12/2018	C	C	C	C	I	C	M
**SC**	8/29/2019	C	C	C	C	C	C	C
**SD**	9/4/2019	C	C	C	C	C	C	M
**TN**	4/4/2019	C	C	C	C	I	I	C
**TX**	7/21/2019	C	C	C	C	C	C	C
**UT**	9/4/2019	C	C	C	C	C	C	M
**VT**	10/8/2018	C	C	I	I	I	I	M
**VA**	7/26/2019	C	C	C	C	C	C	M
**WA**	M	M	M	M	M	M	M	M
**WV**	4/5/2019	C	C	C	C	C	C	I
**WI**	7/26/2019	C	C	I	C	C	C	M
**WY**	8/29/2019	C	C	C	C	C	C	M

[a] Analysis of specific characteristics of the data provided by each state's Board of Dentistry. Entries may be denoted by "C" (Complete) meaning >90% of rows have data for a specific category, "I" (Incomplete) meaning >=10% of rows do not have data, or "M" (Missing) meaning there is no data.

©2022 Serban N et al. *JAMA Network Open*.

When we had incomplete or missing data attributes, we modified the data matching algorithm to cross-reference names and other information from the other two datasets to fill in the incomplete information. For dentists with a missing county in the BOD database, we were able to obtain census tract and county data using Texas A&M Geoservices. For all dentists who had an incomplete address in the BOD database and were not found in the IKN database, their address in the NPPES data was assigned as their office location. However, if this address was out of state, or if the dentist was unable to match with a dentist in the NPPES data, then we performed manual search using Google and Facebook pages. If there was no in-state address found in any of the three databases or via manual search, then we discarded the rows if the number of dentists was insignificant in terms of the state's dentist population. 0.66% and 2.2% of dentists in Tennessee and New York, respectively, were discarded using this method.

The Arkansas BOD dataset provided the dentists' mailing address instead of their office address. For the dentists listed in the BOD data who were unable to match with a dentist in both the IKN and NPPES datasets, the mailing address was used as the dental office. The address in the NPPES data was used for 33% of the state's dentist population, and the mailing address provided in the BOD data was used for 18% of the state's dentists.

For 14.2% of the dentists in Wisconsin, the address listed in the BOD dataset consisted of just the city, state, and zip code of the dental office. The first of three approaches to extract address information for these dentists entailed sampling data from the existing complete addresses. Specifically, we listed all the complete addresses grouped by zip code and then assigned one of those addresses to a provider with an incomplete address, ensuring that the provider‘s zip code stayed the same. This procedure ensured that the rurality/urbanicity classification assigned to each provider would remain unchanged, since each zip code mapped to a specific RUCA code ranging from 1 to 10. This procedure was able to cover 11.4% of the state's dentist population. It could not cover those providers whose zip codes were not found in the list of existing addresses. The second method consisted of searching the dentist with the given zip code to try to find their complete address with the same zip code, using Google Maps and Facebook company pages. This technique worked for most of the remaining incomplete addresses (2.4% of the dentist population). The last method involved entering the city, state, and zip code into Google Maps and then picking an address that included the same information, which was used for the remaining dentists (0.4% of the dentist population).

Washington D.C.'s BOD dataset also contained incomplete addresses for several dentists (9.8% of the dentist population), which consisted of the city, state, and zip code. For the dentists listed in the BOD data which were unable to match with a dentist in both the IKN and NPPES datasets, the zip code provided in the BOD dataset was used to extract the corresponding census tract, and an address in the same census tract was assigned to the dentist.

National Plan and Provider Enumeration System (NPPES)

The NPPES database includes all dental care providers (active or inactive), without the specification of which providers are currently active. We acquired the data from Centers for Medicare and Medicaid Services (CMS) in 2020. CMS has developed the NPPES to assign a unique identifier for health care providers and health plans. The database is updated monthly; however not all providers update their information as soon as their information changes, such as their office location.

NPPES contains a registry of all dental healthcare providers with a National Provider Identifier (NPI), a 10-digit random number unique to each healthcare provider or organization. Each NPI is unique although there may be multiple NPIs with the same address; when this is the case, it means that multiple providers practice at the same address. The attributes in the data used in our analysis were:

- Entity Type: Entity 1 for individual (physicians, sole proprietors); Entity 2 for dental care practices.
- Address: Each NPI record has two specified addresses: a billing address and a practice address. Among the two, CMS verifies the second, but there is not control on whether the physician effectively works in the provided address.
- Taxonomy Code: A Code designating the provider type, classification, and specialization. Each provider can specify up to 15 different taxonomy codes. The taxonomy codes considered in this analysis were:

    - Dentist - 122300000X (General Dentist)

©2022 Serban N et al. *JAMA Network Open*.

- Dental Public Health - 1223D0001X (Other Licensed Professional)
- Dentist Anesthesiologist - 1223D0004X (Specialist)
- Endodontics - 1223E0200X (Specialist)
- General Practice - 1223G0001X (General Dentist)
- Oral and Maxillofacial Pathology - 1223P0106X (Specialist)
- Oral and Maxillofacial Radiology - 1223X0008X (Specialist)
- Oral and Maxillofacial Surgery - 1223S0112X (Specialist)
- Orthodontics and Dentofacial Orthopedics - 1223X0400X (Specialist)
- Pediatric Dentistry - 1223P0221X (Pediatric Dentistry)
- Periodontics - 1223P0300X (Specialist)
- Prosthodontics - 1223P0700X (Specialist)
- The taxonomies listed above were chosen because they represent the types of providers where children would receive preventive or restorative care. We further group these taxonomies as follows:
- General = 'General Practice', 'Dentist', 'Dental Public Health'
- Pediatric = 'Pediatric Dentistry'
- Specialist = 'Orthodontics and Dentofacial Orthopedics', 'Oral and Maxillofacial Surgery', 'Endodontics', 'Periodontics', 'Prosthodontics', 'Oral and Maxillofacial Pathology', Dentist Anesthesiologist',' Oral and Maxillofacial Radiology'.

We included multiple types of dentists into the category of specialized dentists because of their overall low participation in public insurance programs; further analysis by specialization is possible using our data however we didn’t include such results to keep the description of the data simple.

InsureKidsNow.gov (IKN)

The IKN database consists of all dental care providers reporting that accept public insurance in their practice. These data include the name of the provider, or of his/her practice, the address of the provider and his/her specialty. There are several challenges with this data. The IKN data may have repeats (same name and same address), which were removed prior to using the data matching algorithm. However, multiple providers have the same name but with different addresses. This can arise from multiple reasons, the most common being that a given dentist practices at multiple addresses.

Online-Supplemental Material B: Data Matching Algorithm

BOD Processing

With each state, the matching procedure commenced by processing each state’s BOD data. In the processing step, the goal was to establish a set of dentists with the following criteria:

1. Have an active license;
2. Have an in-state address; and
3. Have a current practice.

When the column of license status was included in the data, we considered those who are active or on probation but dropped those whose licenses have been suspended or revoked. When no information of license status is provided, we then used license expiration date to drop off the inactive dentists.

NPPES and IKN Processing and Matching

After the processing of the BOD data, the IKN and NPPES databases were then processed and matched for each state. Unlike the BOD data, the IKN and NPPES data could largely be pre-processed on a national level. This preprocessing included tasks such as adding a specialty column to the NPPES data, including the re-grouping of the listed taxonomy, as provided in Supplemental Material A, and standardizing the names and addresses of the IKN data, so that duplicates could be dropped out. These pre-processed databases were read into python algorithm code for each state.

The IKN and NPPES databases were then matched using an inner join on the National Provider Index (NPI). Generally, because of blank or inaccurate NPI values in the IKN data, another layer of matching needed to be

©2022 Serban N et al. *JAMA Network Open*.

completed using an inner join of first and last name pairs. Afterwards, the two databases joined separately on NPI's and names were combined and further filtered to remove duplicate address and full name combinations. If the matching was performed correctly, the IKN match rate usually was around 96%.

BOD to IKN-NPPES Matching

The BOD data were matched using an inner join on first and last name pairs with the previously matched IKN-NPPES database. For dentists that did not match, Python FuzzyWuzzy function was used to pair more dentists between the databases. The matching score obtained using Python FuzzyWuzzy needed to be greater than 95 to qualify as a match. Finally, the matches obtained from both methods, FuzzyWuzzy and the inner join, were concatenated into one BOD-IKN-NPPES database.

The goal of the matching was to maximize the IKN match rate with both the NPPES and BOD databases. Therefore, dentists in the IKN data that were not matched into the BOD-IKN-NPPES database were cycled through another matching process with just the BOD first and last name pairs, again requiring a matching score of at least 95 to qualify as a match. Because this new BOD-IKN set of dentists had not matched with the NPPES database, the set required generating a specialty for each dentist, either general, pediatric, or specialized. The numbers and percentages of these BOD-IKN matched dentists are provided in eTable 2.

©2022 Serban N et al. *JAMA Network Open*.

eTable 2.BOD-IKN Matched Dentists Unable to Match with a Dentist in the NPPES Database [a]

State	Total number of dentists	Total number of BOD-IKN Matched dentists	Proportion of dentist population (%)
**AL**	2145	152	7
**AK**	592	16	3
**AZ**	4178	103	2
**AR**	1354	28	2
**CA**	35126	236	1
**CO**	4199	101	2
**CT**	2847	82	3
**DE**	418	21	5
**DC**	766	70	9
**FL**	11871	184	2
**GA**	5139	191	4
**ID**	1048	15	1
**IL**	9242	176	2
**IN**	3541	68	2
**IA**	1807	122	7
**KS**	1534	13	1
**KY**	2506	23	1
**LA**	2269	93	4
**ME**	728	6	1
**MD**	4308	68	2
**MA**	6019	127	2
**MI**	6942	226	3
**MN**	3083	101	3
**MS**	1299	77	6
**MO**	3176	25	1
**MT**	654	13	2
**NE**	1266	13	1
**NV**	1708	28	2
**NH**	1079	24	2
**NJ**	8026	200	2
**NM**	1070	22	2
**NY**	13199	242	2
**NC**	5454	119	2
**ND**	558	13	2
**OH**	6282	67	1
**OK**	2012	56	3
**OR**	2859	44	2
**PA**	7941	289	4
**RI**	579	10	2
**SC**	2685	53	2
**SD**	367	19	5
**TN**	3000	44	1
**TX**	15981	151	1
**UT**	2343	49	2
**VT**	417	14	3
**VA**	5708	109	2
**WV**	850	56	7

[a] The first column displays the total number of dentists in each state. The second column displays the total number of dentists in the IKN database that only matched with a dentist in the state's BOD database, and the third column displays this number as a percentage of the state's dentist population.

©2022 Serban N et al. *JAMA Network Open*.

**WI**	3761	45	1
**WY**	343	14	4

Existing data in the IKN and/or BOD datasets were used to assign a specialty for these dentists, such as the name of their practice provided in the IKN data. For the providers with no specialty information in both the BOD and IKN databases, we further searched for their practice using a common search engine (e.g. Google). If their practice was not found using this method, then we sampled their specialty from a multinomial distribution with the probability values estimated using the derived IKN-BOD-NPPES matches. Finally, the BOD-IKN-NPPES matched database and the BOD-IKN matched database with the simulated specialty keys were merged into one final database.

Stratification by Public Insurance Program Participation

The data were stratified depending on the 'Program Type' of each dentist. The IKN data shows that dentists fall into one of the following categories:

1. Dentists accepting Medicaid only;
2. Dentists accepting CHIP only;
3. Dentists accepting Medicaid and CHIP; and
4. Dentists not accepting either insurance program.

A binary value was assigned to each dentist to signify which insurance programs they accepted.

Caseload Distribution of Dentists Accepting Public Insurance

Some of the dentists had more than one appearance in the IKN data generally because of a dentist having multiple offices. For these dentists, a key assumption was that such a dentist gives an equal amount of time to each of his/her offices. Each dentist was given a 'head value' of 1.0, which would be split equally based on the number of locations in which he/she practiced and whether the dentist accepted Medicaid, CHIP, or both programs at each location. If a provider accepted both Medicaid and CHIP at a single location, then this would be counted as two 'appearances' to account for the provider's participation in both programs.

Based upon the head values of dentists with their corresponding specialty designation, each address was assigned a total 'head count' of dentists within each specialty. Thus, for each office location, we have information on whether the dentists are accepting public insurance programs as well as the total head count of dentists by provider type.

Dentists Not Accepting Public Insurance

Using each state's list of dentists in the IKN data, dentists in the BOD data were extracted that did not match with a dentist in the IKN data. Next, this list of names was matched with the NPPES database using an inner join of first and last name pairs to derive the provider type for these dentists. The remaining dentists not matched into the BOD-NPPES database were assigned a specialty using existing data provided in the BOD dataset (e.g. license type). If no specialty information could be extracted from either dataset, we sampled their specialty from a multinomial distribution with the probability values estimated using the derived matches in the BOD-NPPES database.

Location Information

Using Texas A&M GeoServices, census tract and GPS coordinates were obtained for the addresses of the dentists not accepting public insurance. The addresses provided in the IKN database already had GPS coordinates and county information. The use of Texas A&M's GeoServices allowed for further validation of the matching process to confirm that the addresses were accurate. The geocoded addresses were further used for deriving the rurality/urbanicity classification of the dental offices as well as in mapping.

Online-Supplemental Material C: Additional Results

The results in eTable 3 and eFigure 1 complement the results in the main manuscript.

©2022 Serban N et al. *JAMA Network Open*.

eTable 3. Total Number of Dentists and Percentage of Dentists by Rurality/Urbanicity Classification (Urban, Suburban, Rural)and Taxonomy (General, Pediatric, Specialist) for Each State

State	Total number of dentists	Urban (%)[a]	Suburban (%)	Rural (%)	General (%)	Pediatric (%)	Specialist (%)
**AL**	2145	82	11	7	80	5	16
**AK**	592	57	18	25	90	3	7
**AZ**	4178	94	4	2	84	4	13
**AR**	1354	70	16	13	87	3	9
**CA**	35126	97	2	1	85	3	12
**CO**	4199	89	6	5	81	4	15
**CT**	2847	97	2	0	79	4	17
**DE**	418	87	10	3	80	3	17
**DC**	766	100	0	0	81	4	15
**FL**	11871	98	1	1	83	3	14
**GA**	5139	90	7	3	83	4	13
**ID**	1048	71	18	10	85	3	12
**IL**	9242	94	3	3	89	2	9
**IN**	3541	84	11	5	86	3	11
**IA**	1807	67	12	20	84	3	13
**KS**	1534	73	17	10	88	2	10
**KY**	2506	68	20	12	83	3	14
**LA**	2269	91	6	4	85	3	12
**ME**	728	60	16	24	84	3	13
**MD**	4308	97	2	1	82	3	15
**MA**	6019	98	1	1	79	4	17
**MI**	6942	87	7	6	88	1	10
**MN**	3083	79	11	10	87	2	11
**MS**	1299	54	30	15	86	4	10
**MO**	3176	83	9	8	86	2	12
**MT**	654	40	31	28	85	3	12
**NE**	1266	73	15	13	86	3	11
**NV**	1708	93	5	3	86	4	11
**NH**	1079	66	24	10	79	2	18
**NJ**	8026	99	1	0	78	4	18
**NM**	1070	77	15	8	88	2	10
**NY**	13199	94	3	2	80	4	16
**NC**	5454	84	11	5	86	4	10
**ND**	558	58	23	20	86	2	12
**OH**	6282	85	11	4	83	3	14
**OK**	2012	70	20	10	88	2	10
**OR**	2859	83	12	5	85	4	11
**PA**	7941	92	6	3	82	3	15
**RI**	579	100	0	0	78	4	18
**SC**	2685	86	11	3	85	3	13
**SD**	367	50	28	21	83	3	14
**TN**	3000	81	12	7	83	4	13
**TX**	15981	93	5	3	84	4	12
**UT**	2343	88	7	5	82	5	13
**VT**	417	37	22	41	84	3	13
**VA**	5708	93	3	4	83	3	14

[a] Percentages for each state (urban, suburban, rural; general, pediatric, specialist) may not add to 100 because of rounding.

©2022 Serban N et al. *JAMA Network Open*.

**WV**	850	76	12	13	87	2	12
**WI**	3761	77	9	14	86	3	11
**WY**	343	31	38	31	88	3	9

eFigure 1: Dentists' Participation in CHIP, Medicaid and Public Insurance (Medicaid or CHIP) for Each County

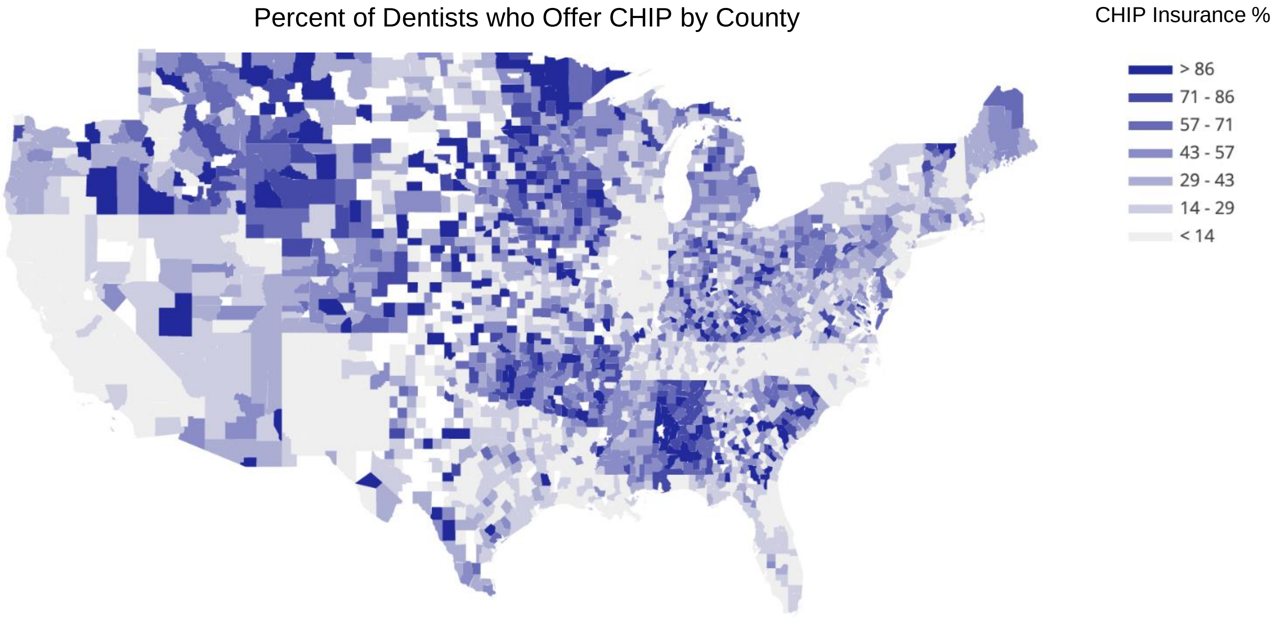

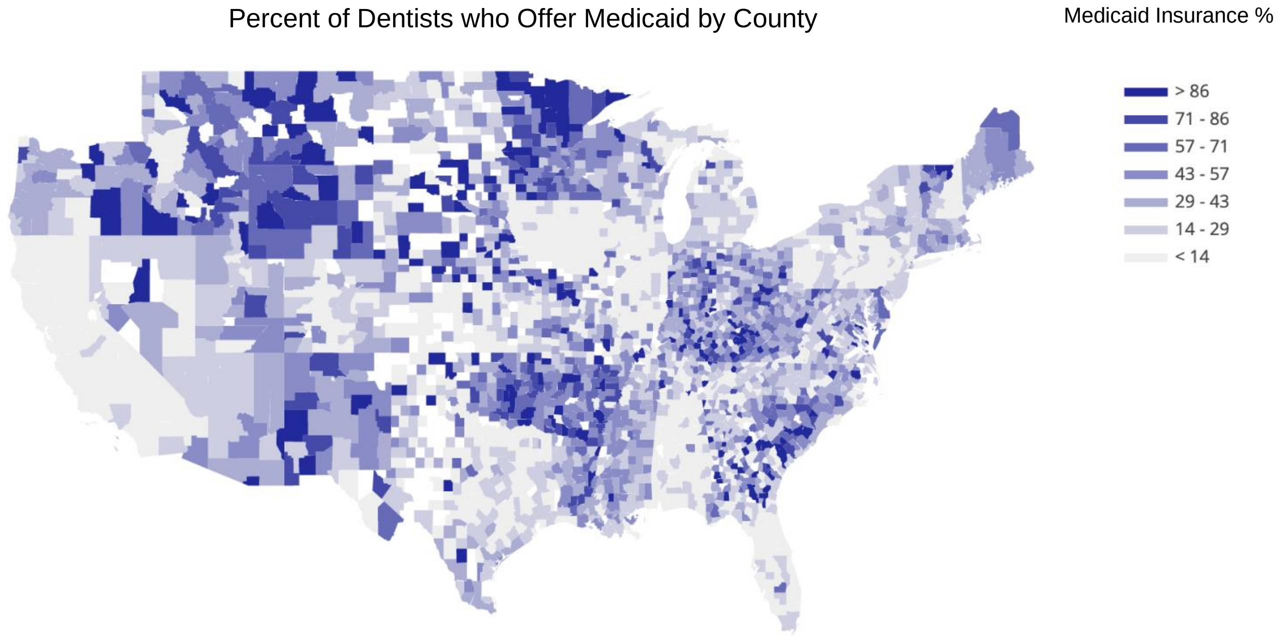

©2022 Serban N et al. *JAMA Network Open*.

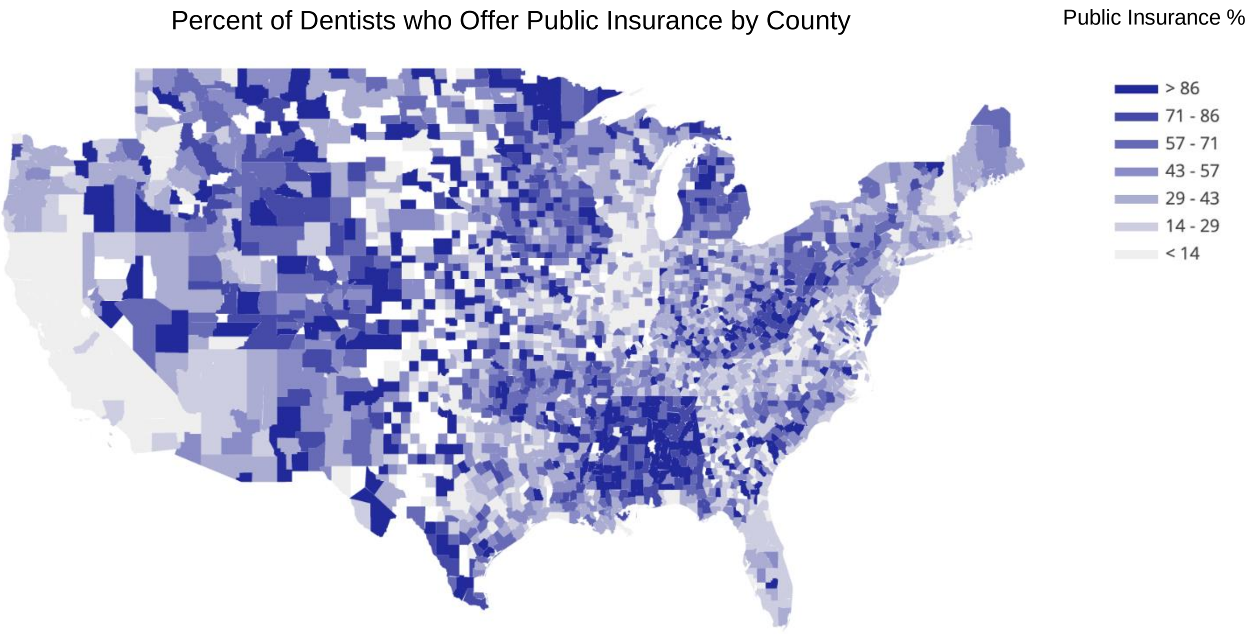

©2022 Serban N et al. *JAMA Network Open*.

eFigure 2. Dental Care Availability for Children in the United States: State Reports

State reports for all states excluding Alaska, Hawaii, and Washington, and also including Washington D.C. are attached in this section.

©2022 Serban N et al. *JAMA Network Open*.

Dental Care Supply
ALABAMA

This state report summarizes data on the dental care supply, differentiated by type of insurance program, provider taxonomy, and rurality-urbanicity of practice address.

Percentage of dentists by provider taxonomy

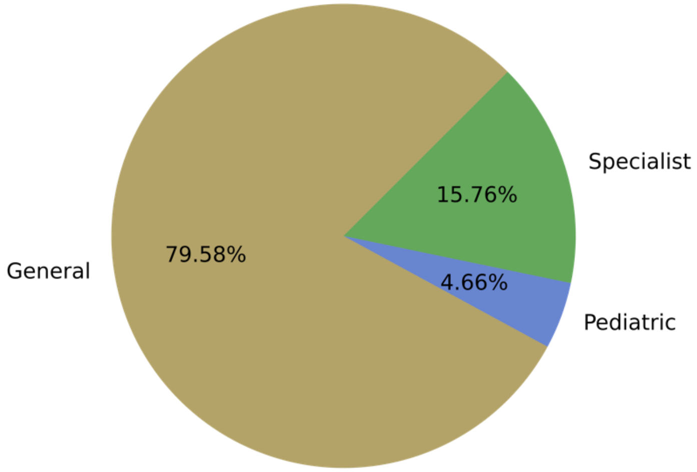

Number of dentists by rurality-urbanicity & provider taxonomy

Rurality-Urbanicity	General	Pediatric	Specialist
Urban	1358	90	312
Suburban	200	8	19
Rural	150	2	8

Percentage of dentists by participation in public insurance programs

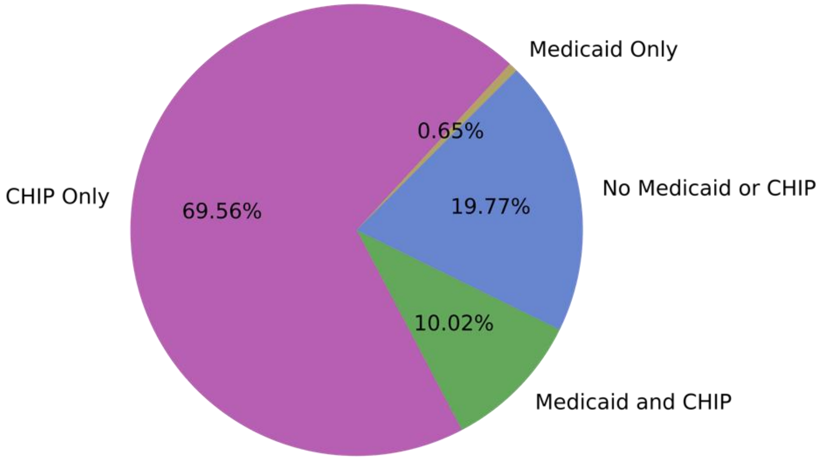

Number of dentists by rurality-urbanicity & participation in public insurance programs

Rurality-Urbanicity	Medicaid Only	CHIP Only	Medicaid and CHIP	No Medicaid or CHIP
Urban	10	1209	163	377
Suburban	2	166	30	28
Rural	2	116	22	19

Number of dentists by provider taxonomy & participation in public insurance programs

Provider Type	Medicaid Only	CHIP Only	Medicaid and CHIP	No Medicaid or CHIP
General	10	1249	159	289
Pediatric	2	54	28	16
Specialist	2	189	28	119

©2022 Serban N et al. *JAMA Network Open*.

Number of dentists

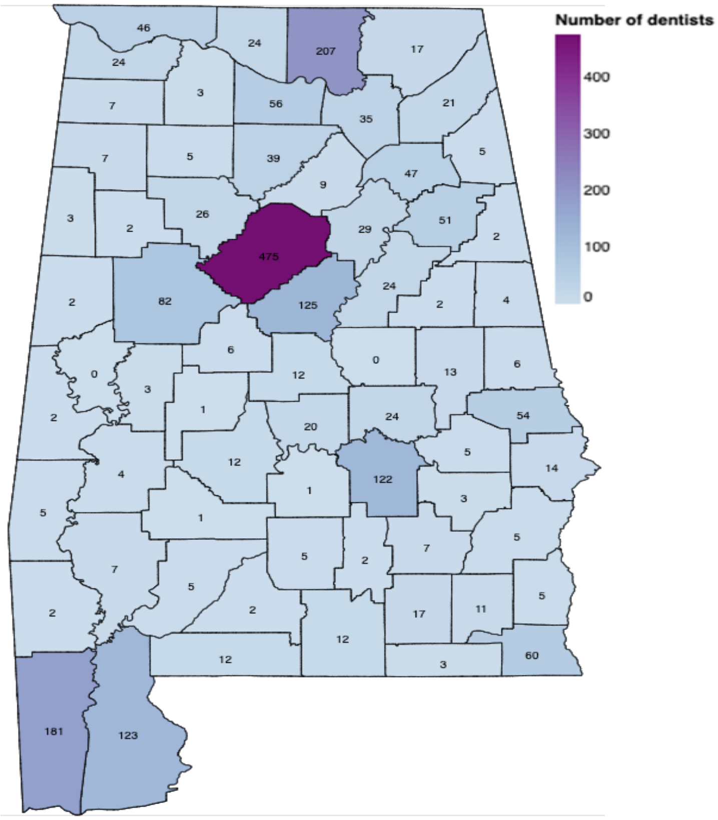

Number of dental hygienists

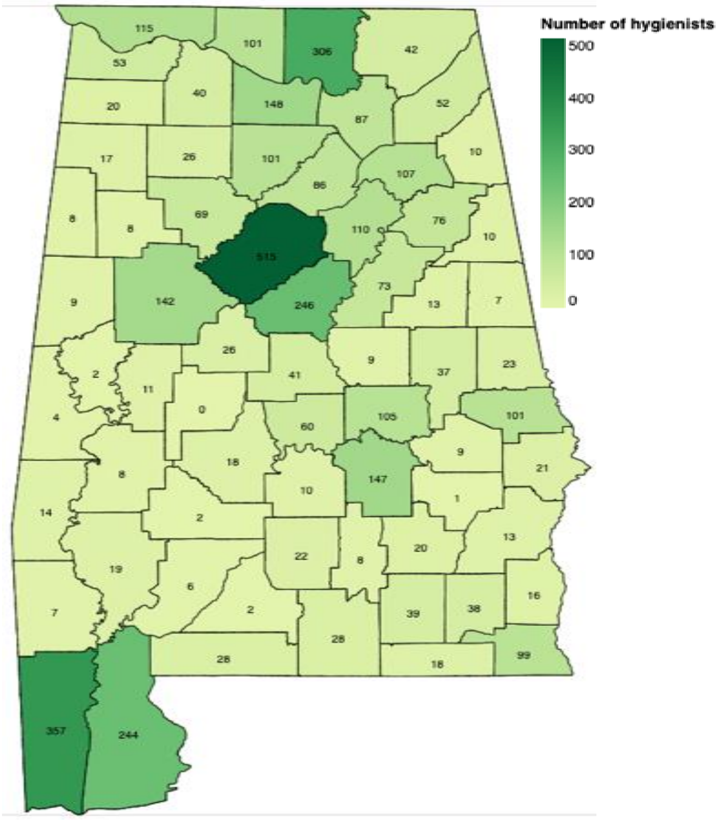

Number of dentists participating in Medicaid

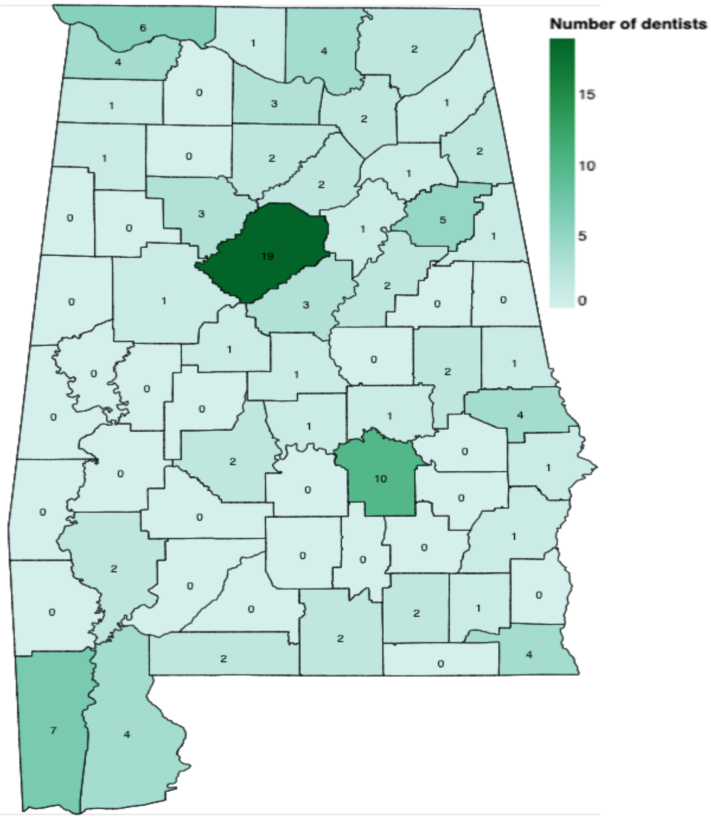

Number of dentists participating in CHIP

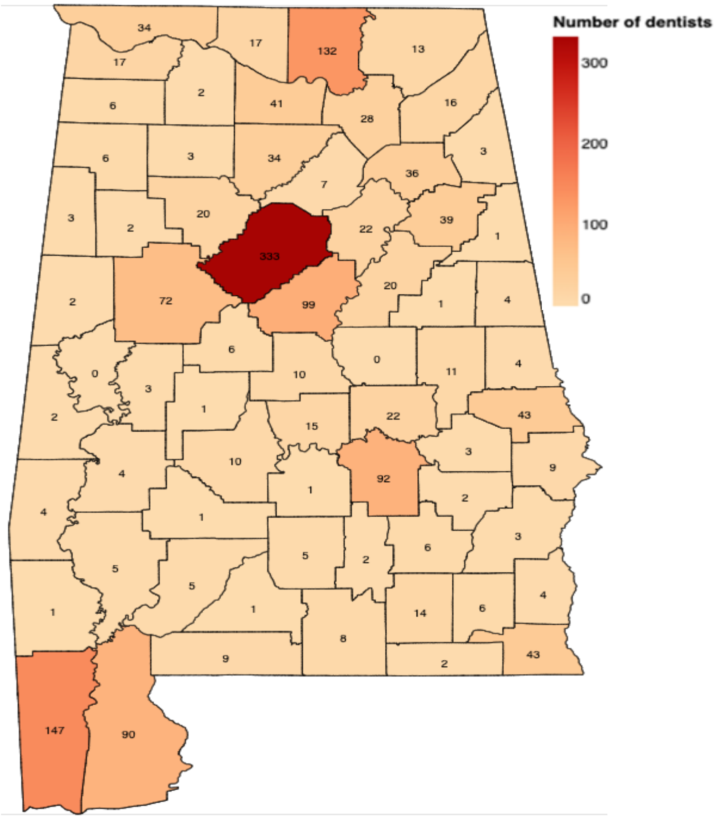

©2022 Serban N et al. *JAMA Network Open*.

Dental Care Supply
ARIZONA

This state report summarizes data on the dental care supply, differentiated by type of insurance program, provider taxonomy, and rurality-urbanicity of practice address.

Percentage of dentists by provider taxonomy

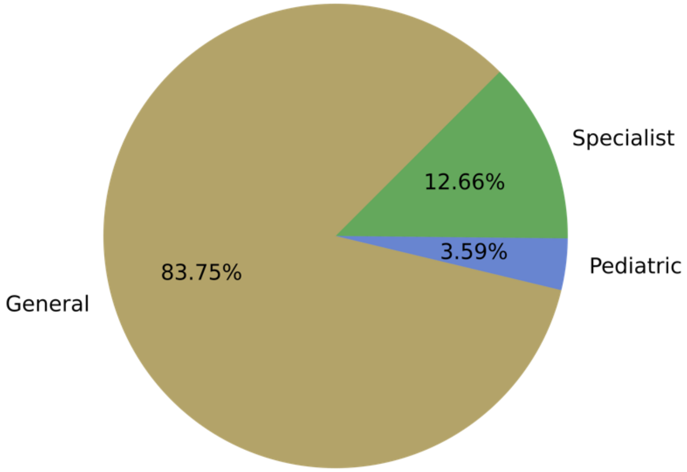

Number of dentists by rurality-urbanicity & provider taxonomy

Rurality-Urbanicity	General	Pediatric	Specialist
Urban	3308	143	515
Suburban	135	7	15
Rural	83	1	3

Percentage of dentists by participation in public insurance programs

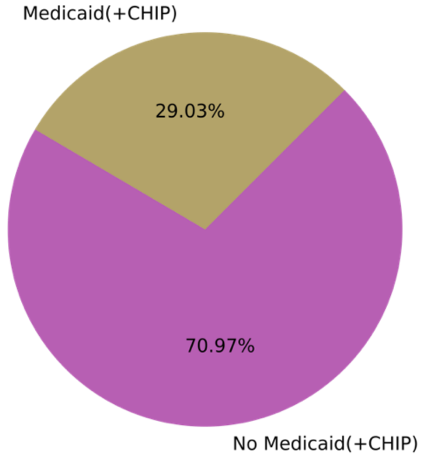

Number of dentists by rurality-urbanicity & participation in public insurance programs

Rurality-Urbanicity	Medicaid(+CHIP)	No Medicaid(+CHIP)
Urban	1138	2828
Suburban	50	107
Rural	34	52

Number of dentists by provider taxonomy & participation in public insurance programs

Provider Type	Medicaid(+CHIP)	No Medicaid(+CHIP)
General	943	2582
Pediatric	113	38
Specialist	166	367

©2022 Serban N et al. *JAMA Network Open*.

Number of dentists

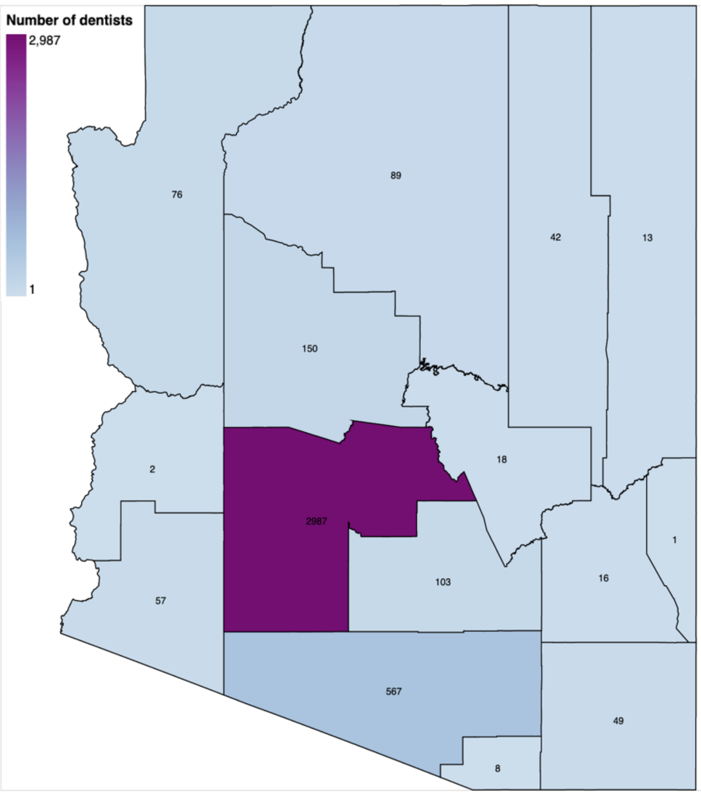

Number of dental hygienists

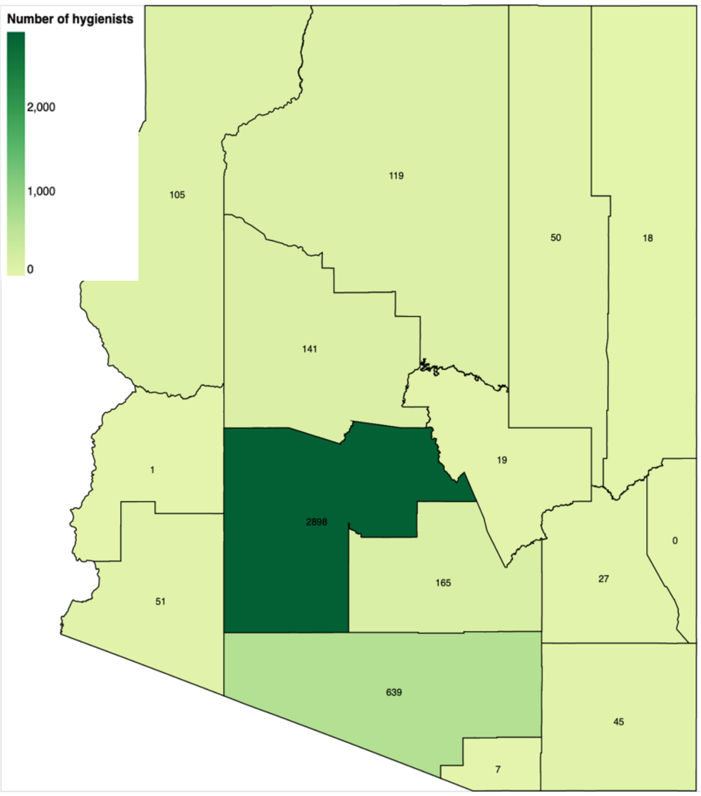

Number of dentists participating in Medicaid(+CHIP)

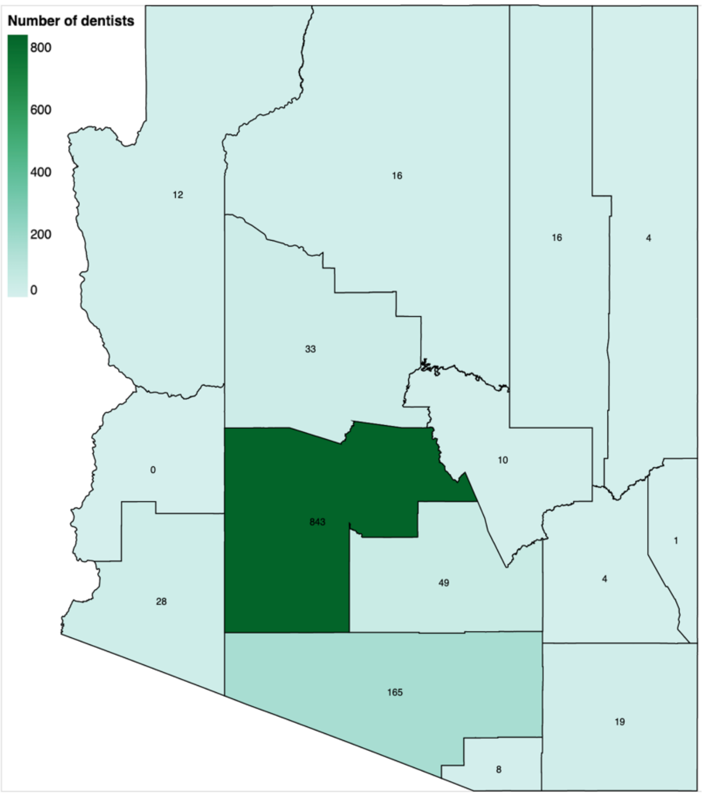

©2022 Serban N et al. *JAMA Network Open*.

Dental Care Supply
ARKANSAS

This state report summarizes data on the dental care supply, differentiated by type of insurance program, provider taxonomy, and rurality-urbanicity of practice address.

Percentage of dentists by provider taxonomy

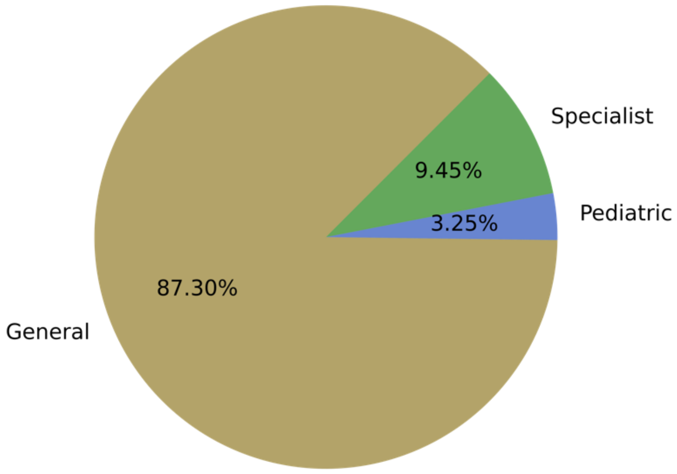

Number of dentists by rurality-urbanicity & provider taxonomy

Rurality-Urbanicity	General	Pediatric	Specialist
Urban	804	36	114
Suburban	202	7	13
Rural	176	0	2

Percentage of dentists by participation in public insurance programs

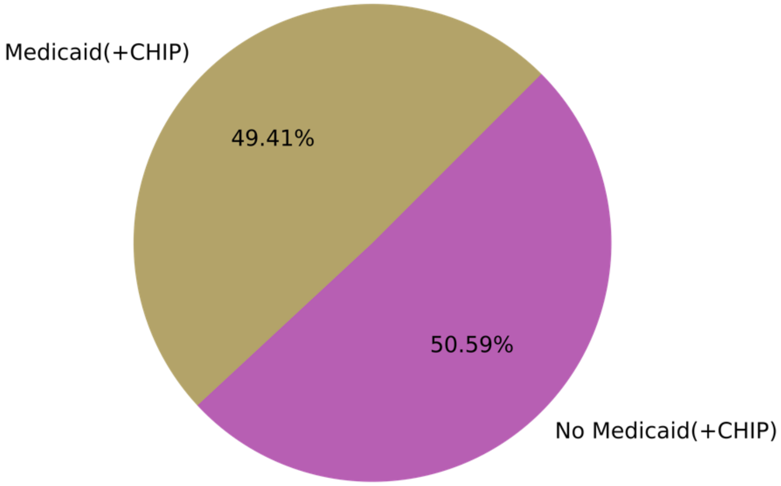

Number of dentists by rurality-urbanicity & participation in public insurance programs

Rurality-Urbanicity	Medicaid(+CHIP)	No Medicaid(+CHIP)
Urban	430	524
Suburban	122	100
Rural	116	61

Number of dentists by provider taxonomy & participation in public insurance programs

Provider Type	Medicaid(+CHIP)	No Medicaid(+CHIP)
General	558	624
Pediatric	40	4
Specialist	72	57

©2022 Serban N et al. *JAMA Network Open*.

Number of dentists

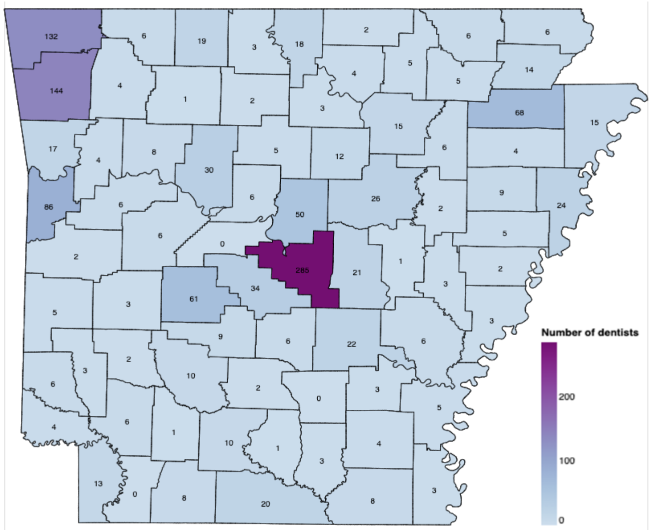

Number of dental hygienists

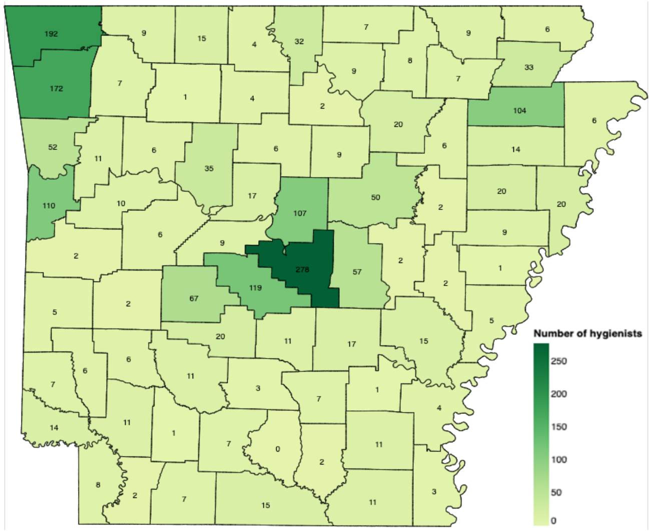

Number of dentists participating in Medicaid(+CHIP)

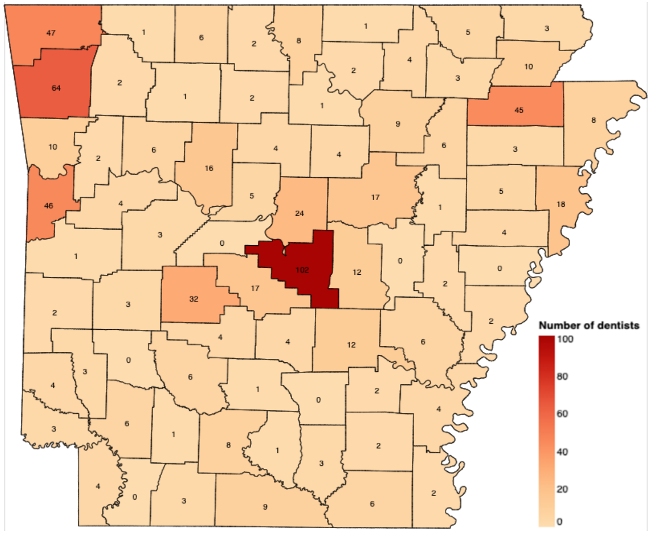

©2022 Serban N et al. *JAMA Network Open*.

Dental Care Supply
CALIFORNIA

This state report summarizes data on the dental care supply, differentiated by type of insurance program, provider taxonomy, and rurality-urbanicity of practice address.

Percentage of dentists by provider taxonomy

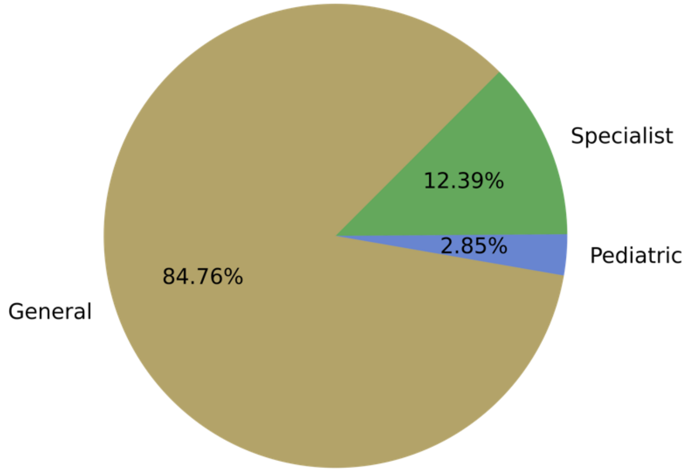

Number of dentists by rurality-urbanicity & provider taxonomy

Rurality-Urbanicity	General	Pediatric	Specialist
Urban	28778	981	4262
Suburban	724	18	80
Rural	303	3	13

Percentage of dentists by participation in public insurance programs

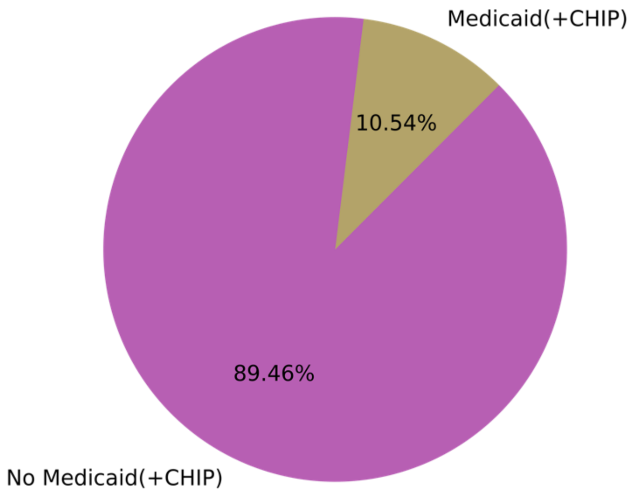

Number of dentists by rurality-urbanicity & participation in public insurance programs

Rurality-Urbanicity	Medicaid(+CHIP)	No Medicaid(+CHIP)
Urban	3652	30368
Suburban	42	781
Rural	10	309

Number of dentists by provider taxonomy & participation in public insurance programs

Provider Type	Medicaid(+CHIP)	No Medicaid(+CHIP)
General	3251	26560
Pediatric	111	891
Specialist	345	4011

©2022 Serban N et al. *JAMA Network Open*.

Number of dentists

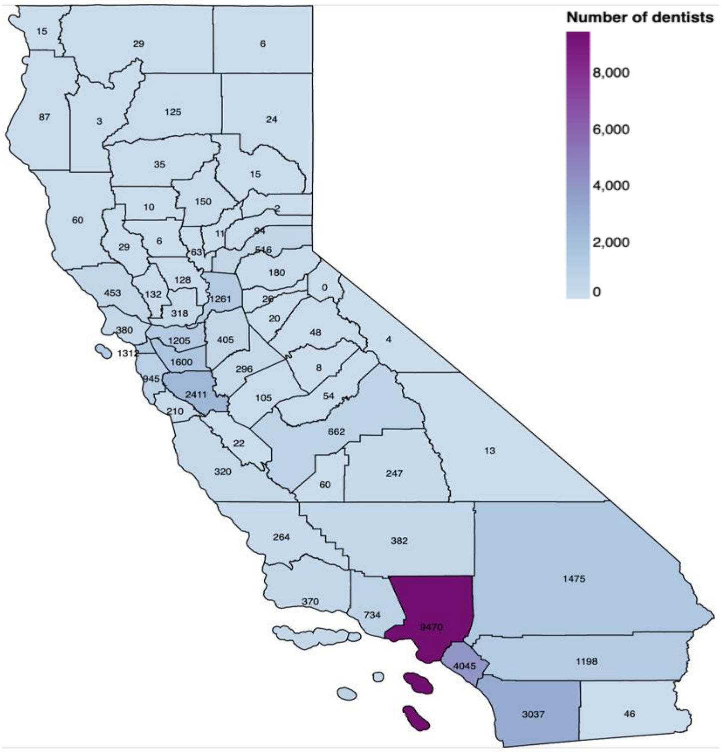

Number of dental hygienists

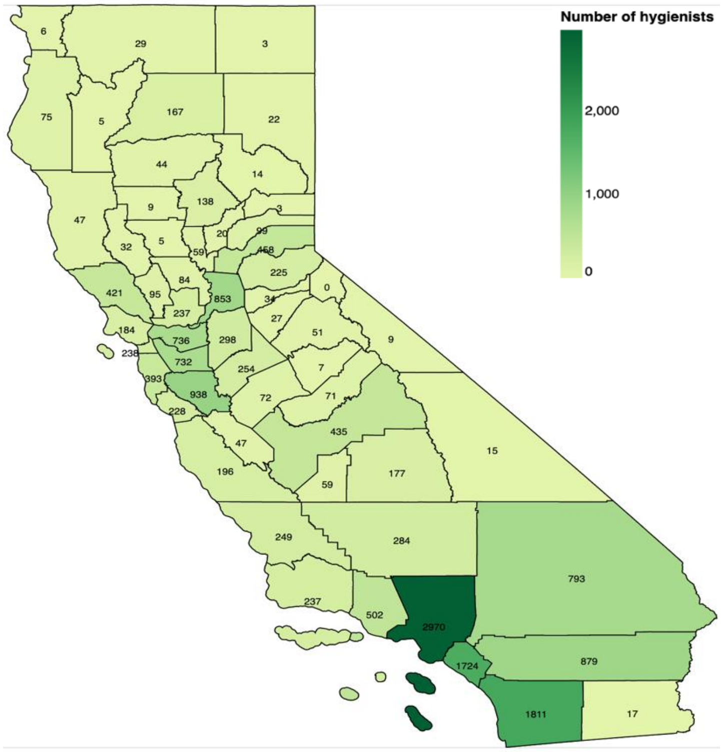

Number of dentists participating in Medicaid(+CHIP)

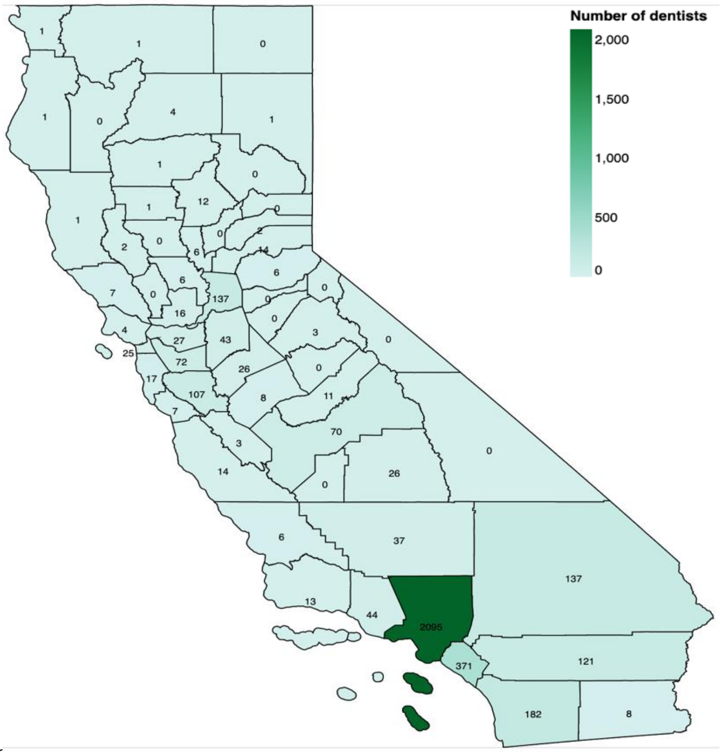

©2022 Serban N et al. *JAMA Network Open.*

Dental Care Supply
COLORADO

This state report summarizes data on the dental care supply, differentiated by type of insurance program, provider taxonomy, and rurality-urbanicity of practice address.

Percentage of dentists by provider taxonomy

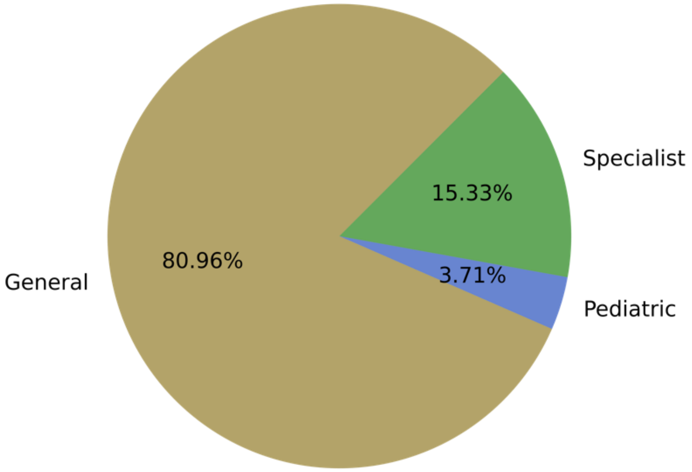

Number of dentists by rurality-urbanicity & provider taxonomy

Rurality-Urbanicity	General	Pediatric	Specialist
Urban	2997	143	605
Suburban	215	9	26
Rural	188	4	13

Percentage of dentists by participation in public insurance programs

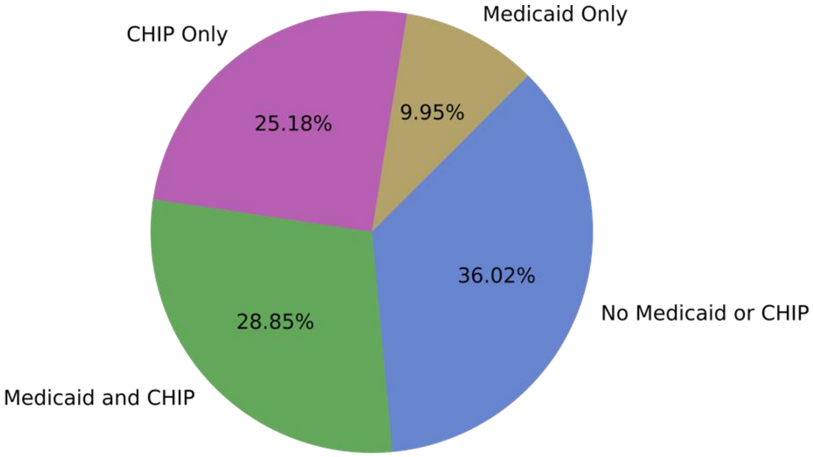

Number of dentists by rurality-urbanicity & participation in public insurance programs

Rurality-Urbanicity	Medicaid Only	CHIP Only	Medicaid and CHIP	No Medicaid or CHIP
Urban	382	931	1063	1369
Suburban	23	70	87	70
Rural	13	57	62	73

Number of dentists by provider taxonomy & participation in public insurance programs

Provider Type	Medicaid Only	CHIP Only	Medicaid and CHIP	No Medicaid or CHIP
General	240	979	1015	1167
Pediatric	20	15	96	25
Specialist	158	64	101	321

©2022 Serban N et al. *JAMA Network Open*.

Number of dentists

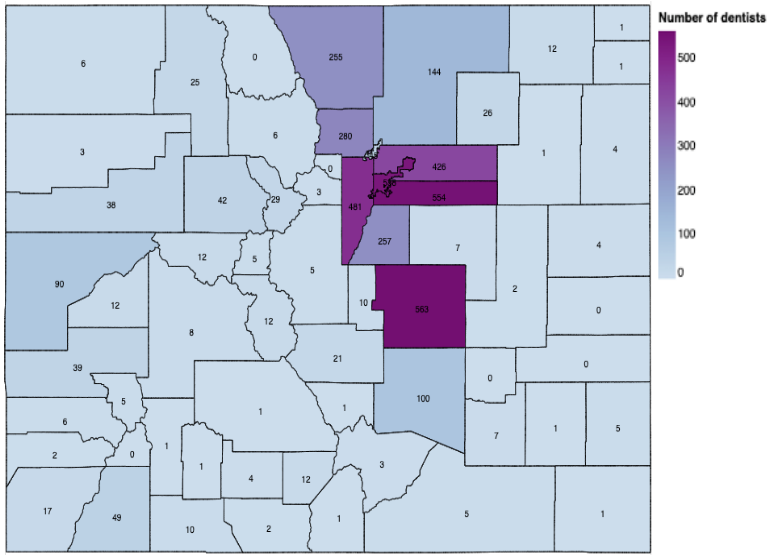

Number of dental hygienists

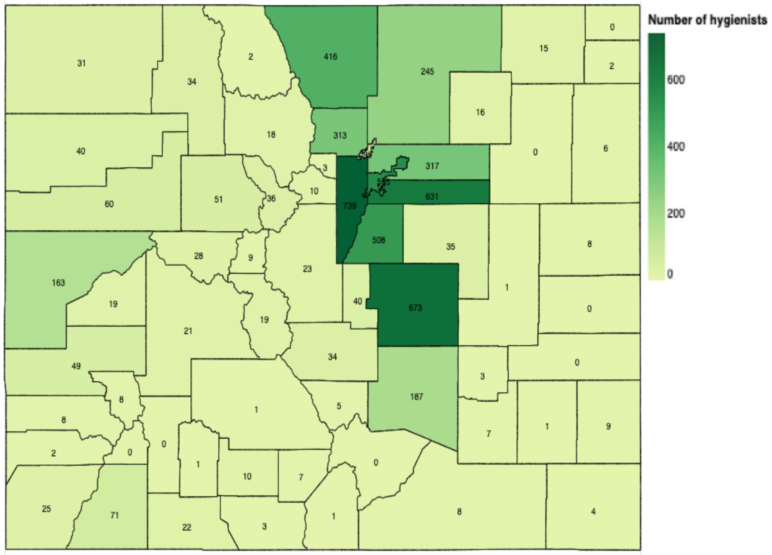

Number of dentists participating in Medicaid

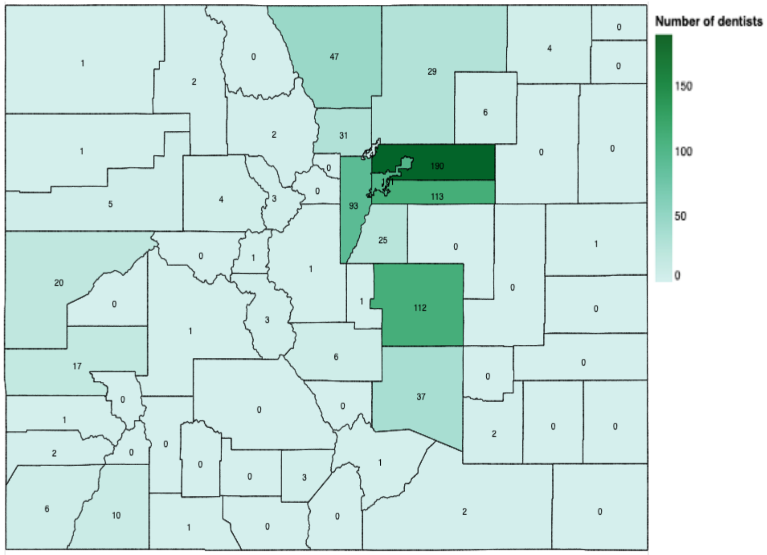

Number of dentists participating in CHIP

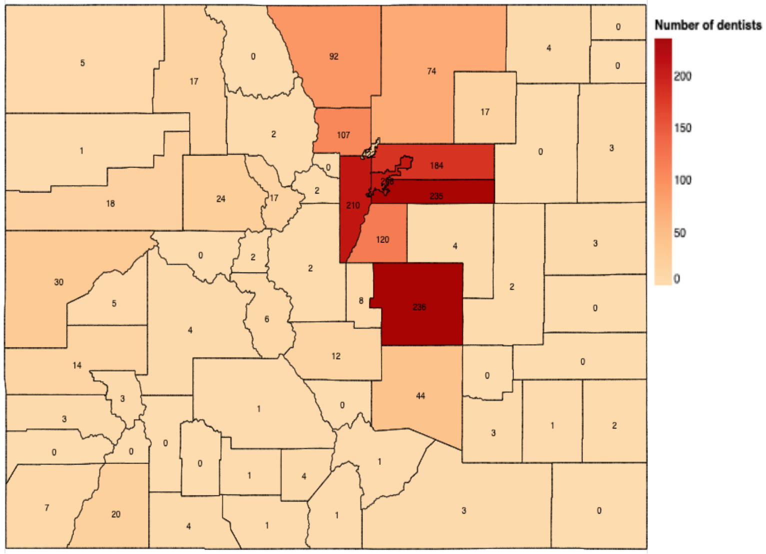

©2022 Serban N et al. *JAMA Network Open*.

Dental Care Supply
CONNECTICUT

This state report summarizes data on the dental care supply, differentiated by type of insurance program, provider taxonomy, and rurality-urbanicity of practice address.

Percentage of dentists by provider taxonomy

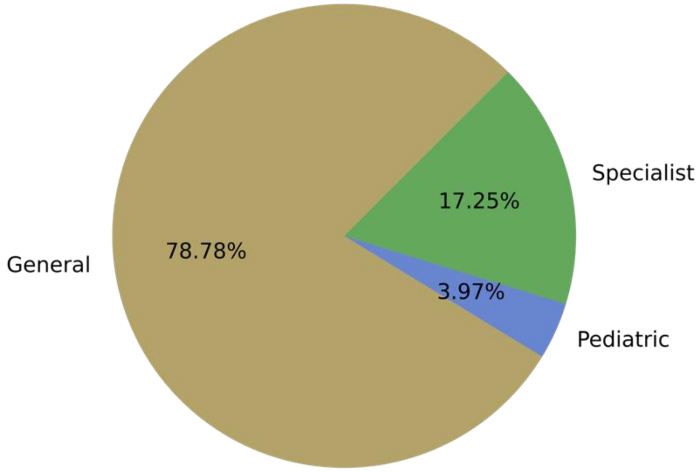

Number of dentists by rurality-urbanicity & provider taxonomy

Rurality-Urbanicity	General	Pediatric	Specialist
Urban	2176	110	481
Suburban	57	3	9
Rural	10	0	1

Percentage of dentists by participation in public insurance programs

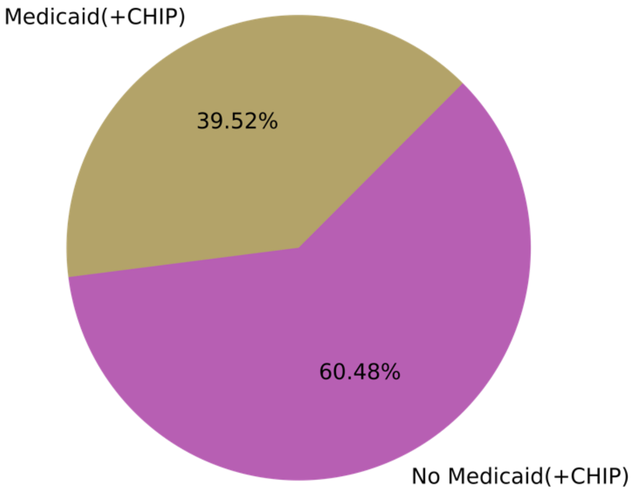

Number of dentists by rurality-urbanicity & participation in public insurance programs

Rurality-Urbanicity	Medicaid(+CHIP)	No Medicaid(+CHIP)
Urban	1085	1682
Suburban	37	32
Rural	3	8

Number of dentists by provider taxonomy & participation in public insurance programs

Provider Type	Medicaid(+CHIP)	No Medicaid(+CHIP)
General	837	1406
Pediatric	94	19
Specialist	194	297

©2022 Serban N et al. *JAMA Network Open*.

Number of dentists

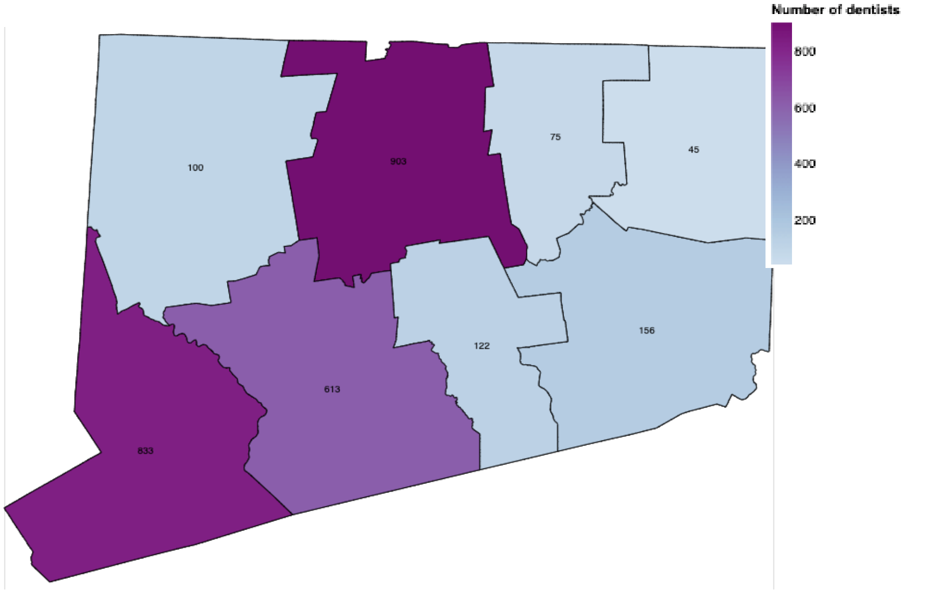

Number of dental hygienists

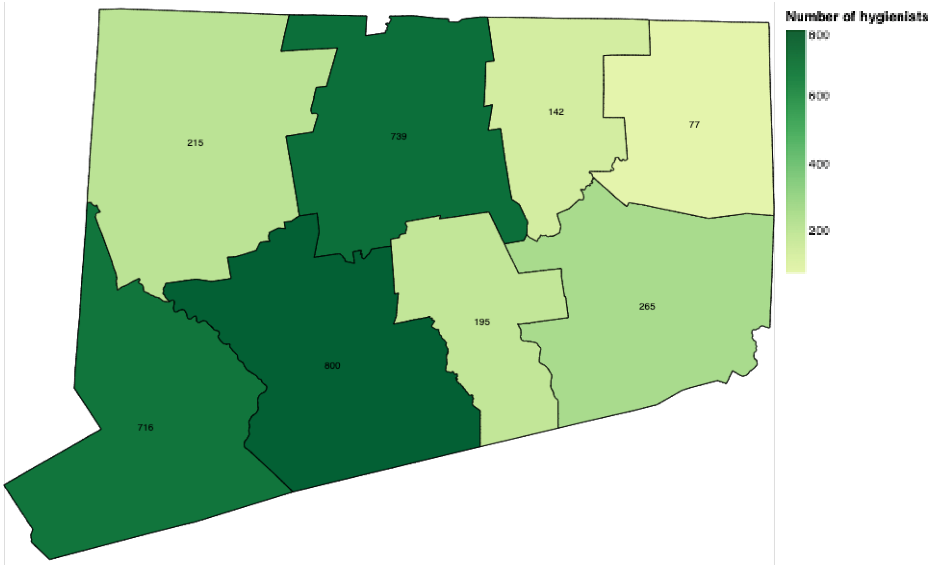

Number of dentists participating in Medicaid(+CHIP)

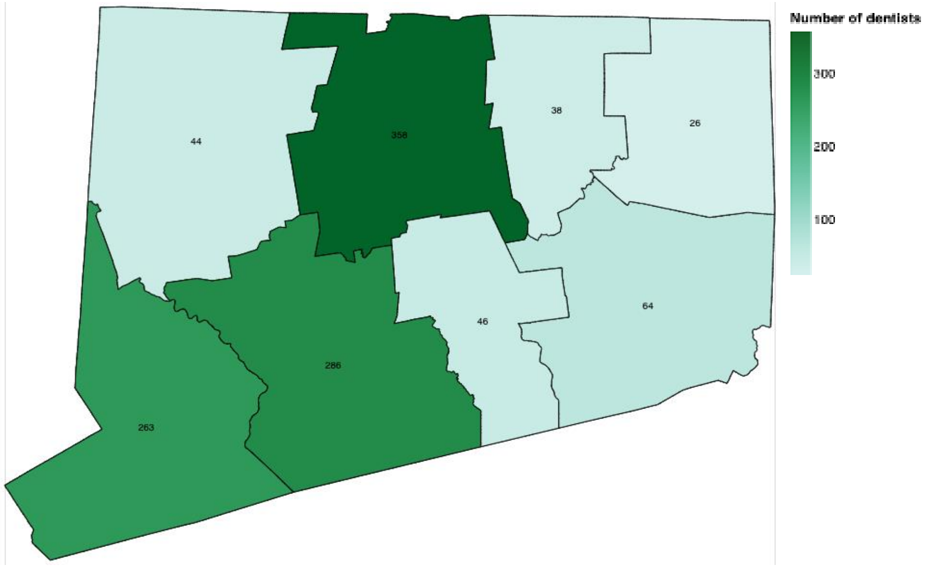

©2022 Serban N et al. *JAMA Network Open*.

Dental Care Supply
DELAWARE

This state report summarizes data on the dental care supply, differentiated by type of insurance program, provider taxonomy, and rurality-urbanicity of practice address.

Percentage of dentists by provider taxonomy

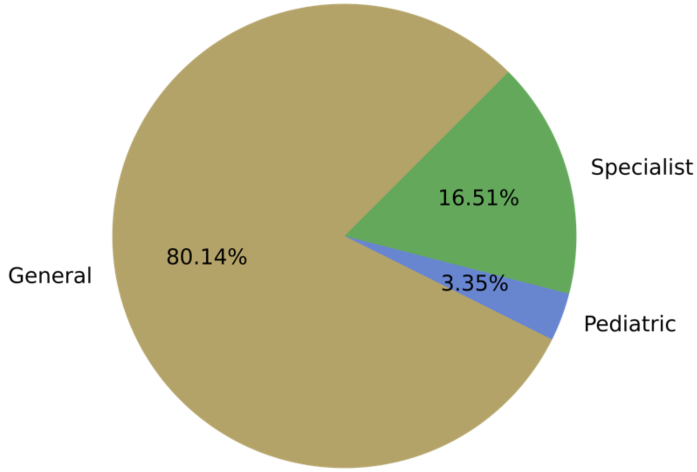

Number of dentists by rurality-urbanicity & provider taxonomy

Rurality-Urbanicity	General	Pediatric	Specialist
**Urban**	292	12	61
**Suburban**	32	2	8
**Rural**	11	0	0

Percentage of dentists by participation in public insurance programs

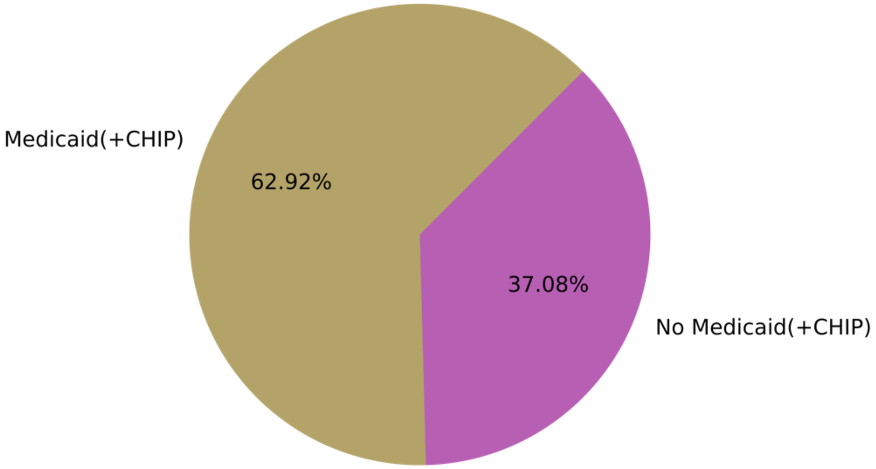

Number of dentists by rurality-urbanicity & participation in public insurance programs

Rurality-Urbanicity	Medicaid(+CHIP)	No Medicaid(+CHIP)
**Urban**	234	132
**Suburban**	22	19
**Rural**	7	4

Number of dentists by provider taxonomy & participation in public insurance programs

Provider Type	Medicaid(+CHIP)	No Medicaid(+CHIP)
**General**	207	128
**Pediatric**	11	3
**Specialist**	45	24

©2022 Serban N et al. *JAMA Network Open*.

Number of dentists

Number of dental hygienists

Number of dentists participating in Medicaid(+CHIP)

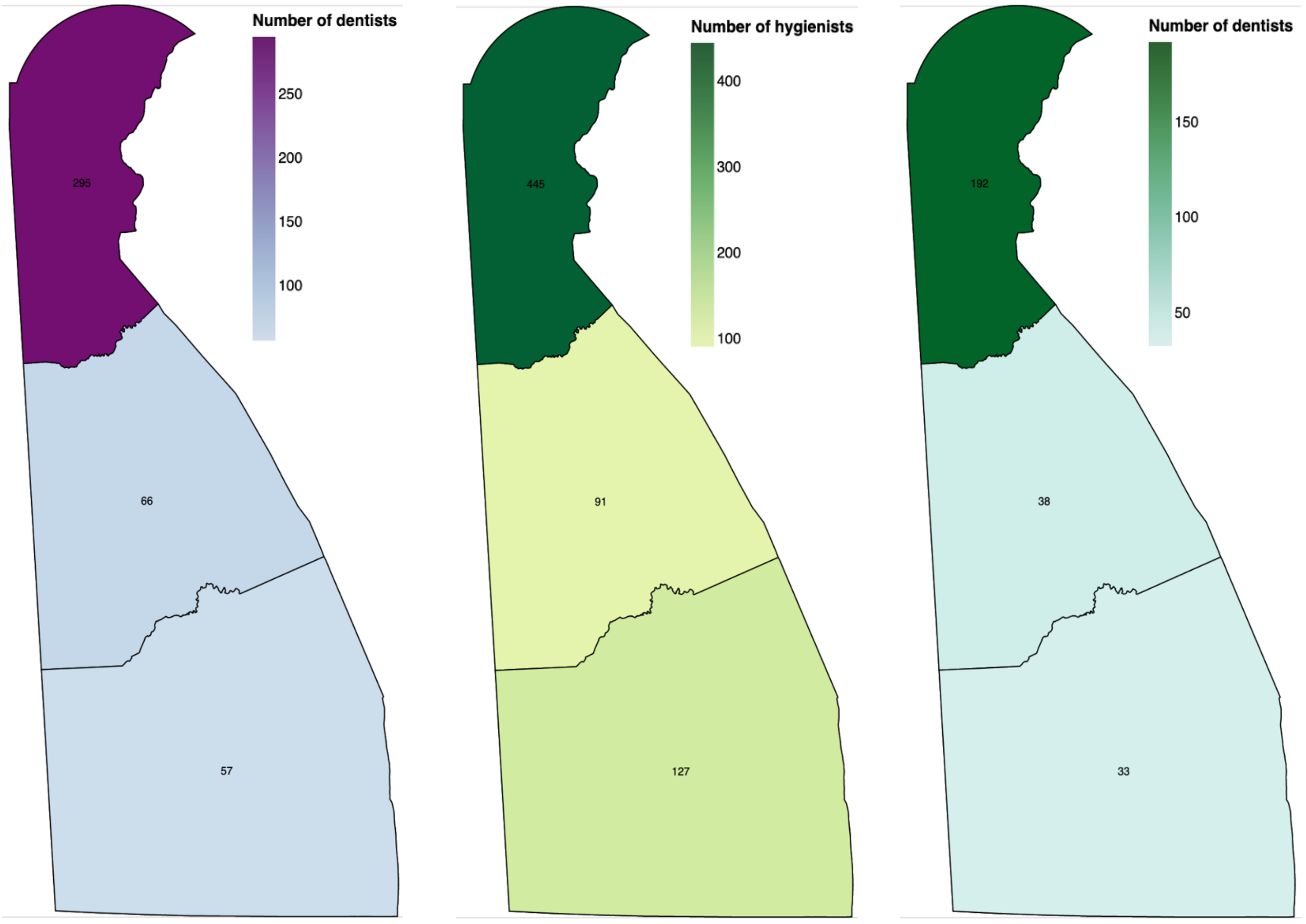

©2022 Serban N et al. *JAMA Network Open*.

Dental Care Supply
WASHINGTON D.C.

This state report summarizes data on the dental care supply, differentiated by type of insurance program, provider taxonomy, and rurality-urbanicity of practice address.

Percentage of dentists by provider taxonomy

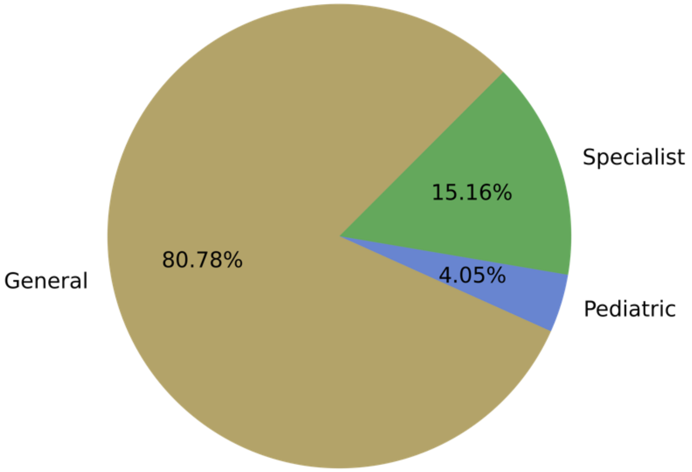

Number of dentists by rurality-urbanicity & provider taxonomy

Rurality-Urbanicity	General	Pediatric	Specialist
**Urban**	618	31	116
**Suburban**	0	0	0
**Rural**	0	0	0

Percentage of dentists by participation in public insurance programs

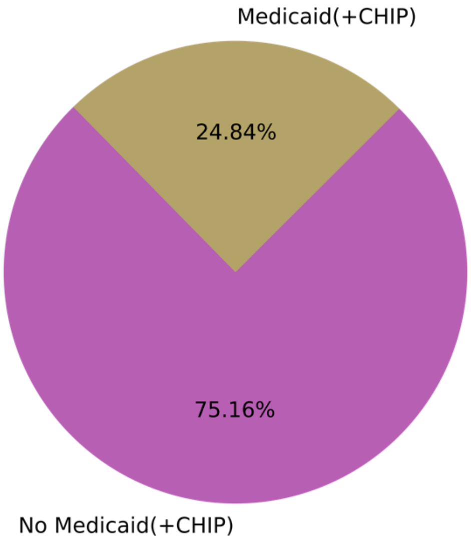

Number of dentists by rurality-urbanicity & participation in public insurance programs

Rurality-Urbanicity	Medicaid(+CHIP)	No Medicaid(+CHIP)
**Urban**	191	575
**Suburban**	0	0
**Rural**	0	0

Number of dentists by provider taxonomy & participation in public insurance programs

Provider Type	Medicaid(+CHIP)	No Medicaid(+CHIP)
**General**	161	457
**Pediatric**	13	18
**Specialist**	16	100

©2022 Serban N et al. *JAMA Network Open*.

Number of dentists

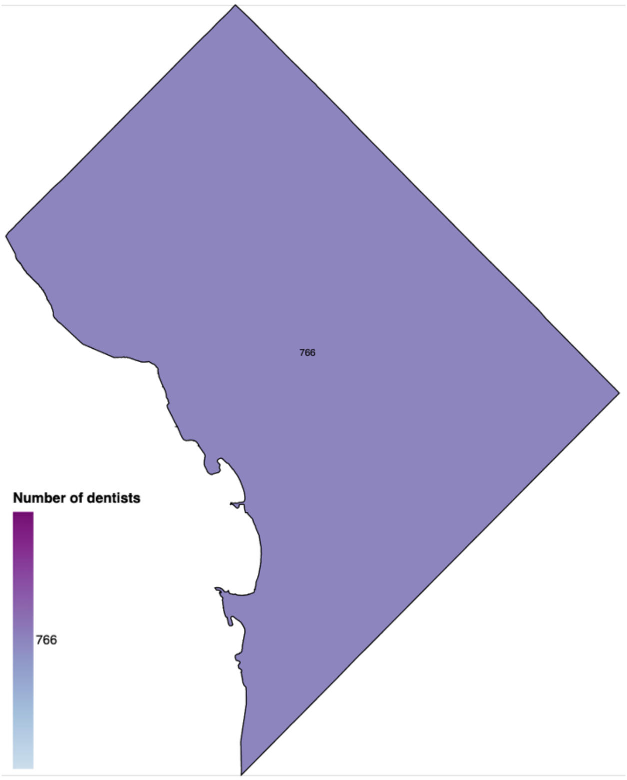

Number of dental hygienists

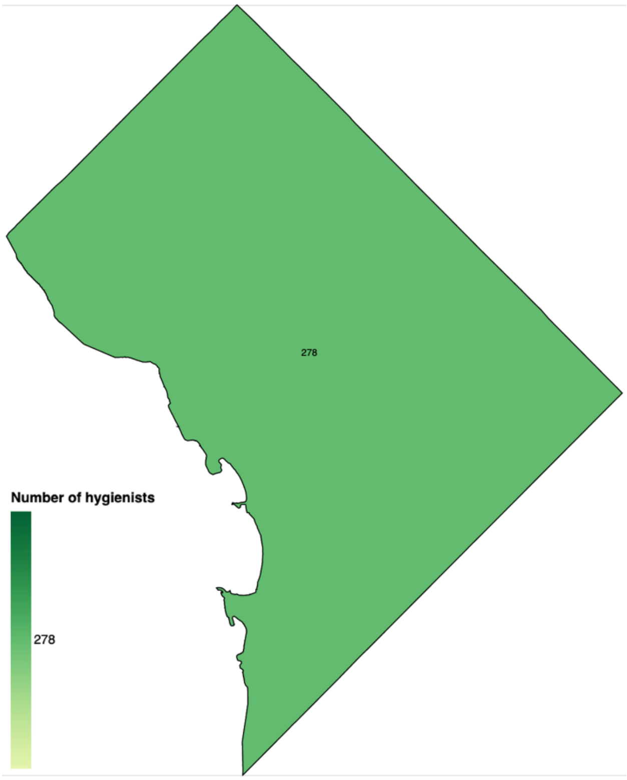

Number of dentists participating in Medicaid(+CHIP)

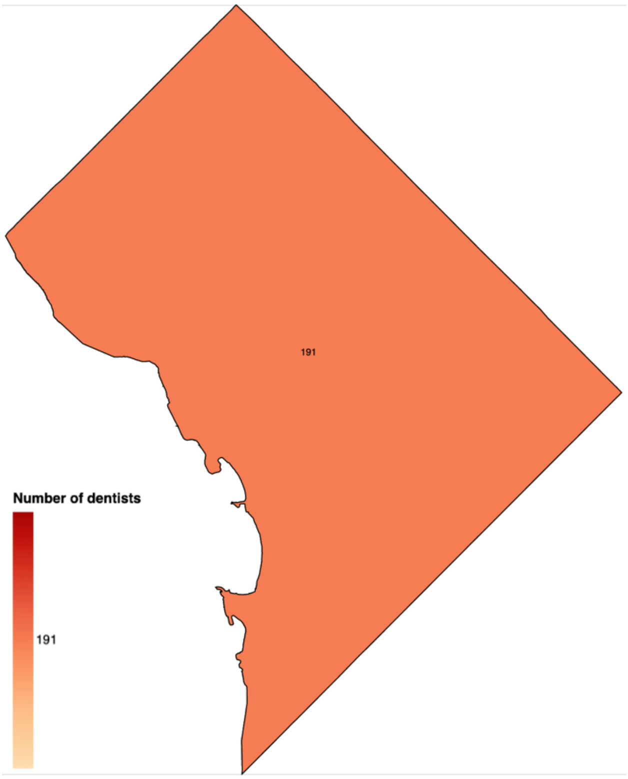

©2022 Serban N et al. *JAMA Network Open*.

Dental Care Supply
FLORIDA

This state report summarizes data on the dental care supply, differentiated by type of insurance program, provider taxonomy, and rurality-urbanicity of practice address.

Percentage of dentists by provider taxonomy

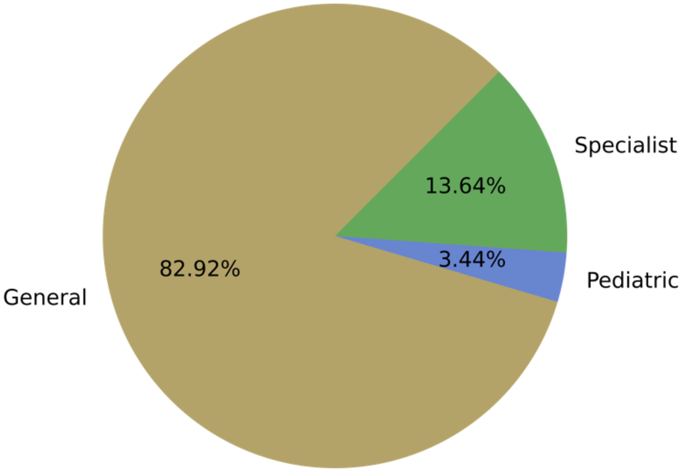

Number of dentists by rurality-urbanicity & provider taxonomy

Rurality-Urbanicity	General	Pediatric	Specialist
Urban	9641	400	1601
Suburban	135	4	17
Rural	71	3	2

Percentage of dentists by participation in public insurance programs

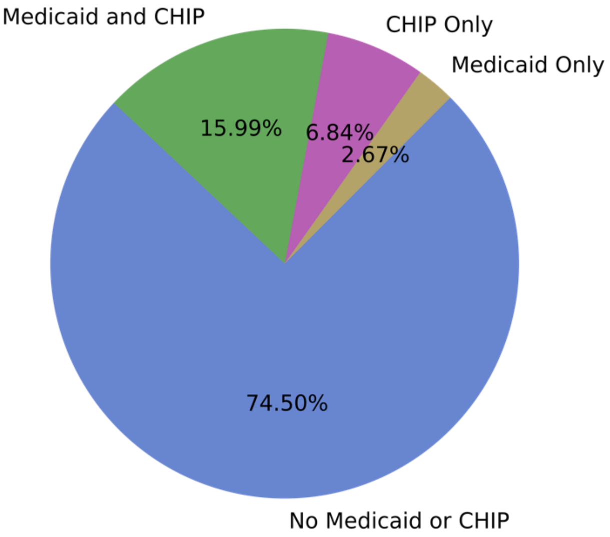

Number of dentists by rurality-urbanicity & participation in public insurance programs

Rurality-Urbanicity	Medicaid Only	CHIP Only	Medicaid and CHIP	No Medicaid or CHIP
Urban	305	795	1854	8688
Suburban	7	4	24	120
Rural	6	8	24	39

Number of dentists by provider taxonomy & participation in public insurance programs

Provider Type	Medicaid Only	CHIP Only	Medicaid and CHIP	No Medicaid or CHIP
General	258	616	1513	7476
Pediatric	18	56	185	151
Specialist	42	142	204	1235

©2022 Serban N et al. *JAMA Network Open*.

Number of dentists

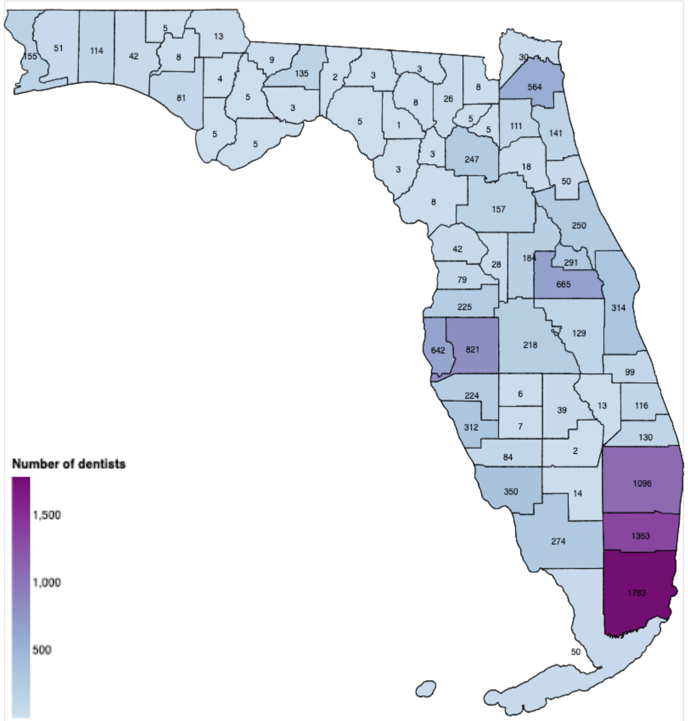

Number of dental hygienists

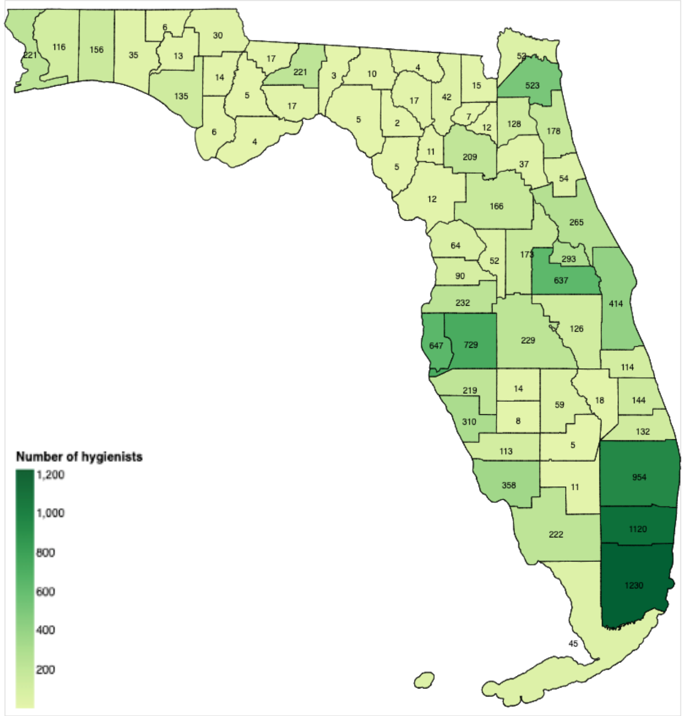

Number of dentists participating in Medicaid

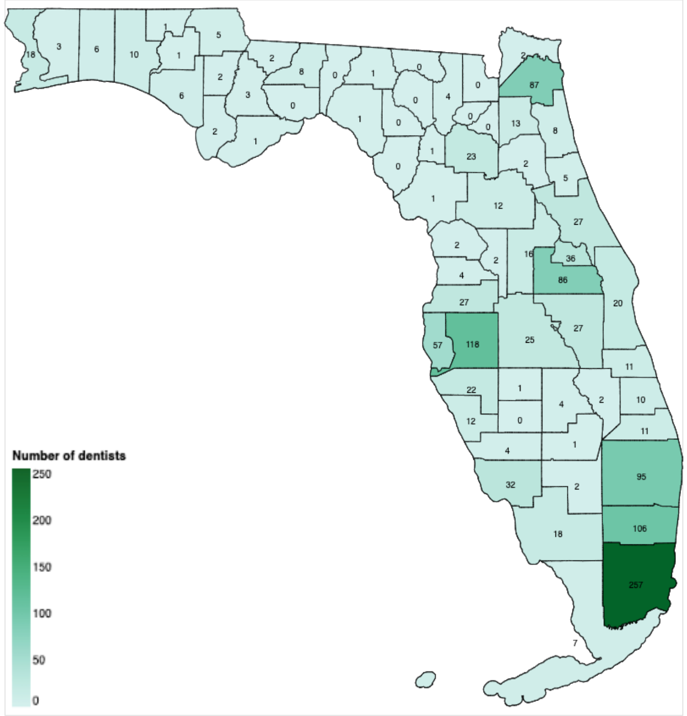

Number of dentists participating in CHIP

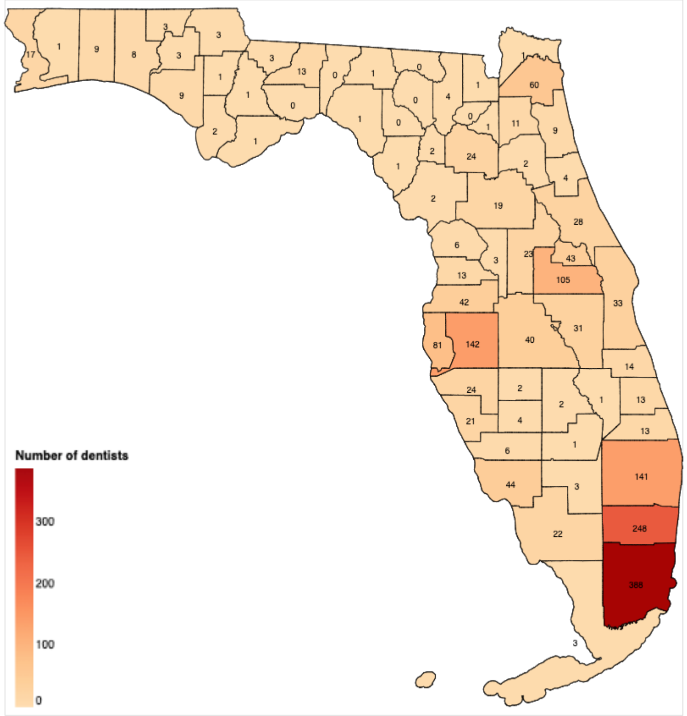

©2022 Serban N et al. *JAMA Network Open*.

Dental Care Supply
GEORGIA

This state report summarizes data on the dental care supply, differentiated by type of insurance program, provider taxonomy, and rurality-urbanicity of practice address.

Percentage of dentists by provider taxonomy

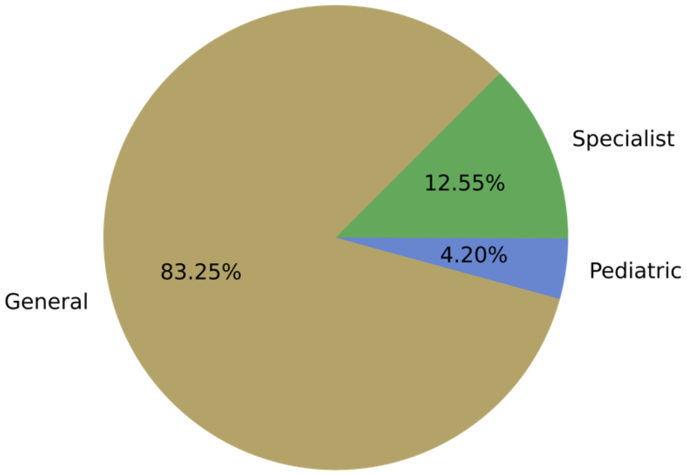

Number of dentists by rurality-urbanicity & provider taxonomy

Rurality-Urbanicity	General	Pediatric	Specialist
Urban	3808	207	601
Suburban	307	7	38
Rural	164	2	5

Percentage of dentists by participation in public insurance programs

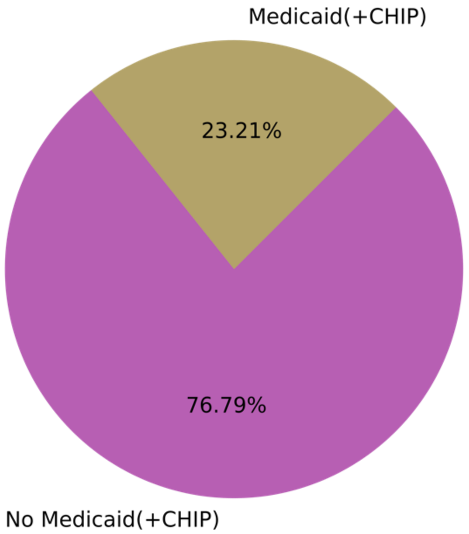

Number of dentists by rurality-urbanicity & participation in public insurance programs

Rurality-Urbanicity	Medicaid(+CHIP)	No Medicaid(+CHIP)
Urban	1036	3580
Suburban	90	262
Rural	67	104

Number of dentists by provider taxonomy & participation in public insurance programs

Provider Type	Medicaid(+CHIP)	No Medicaid(+CHIP)
General	935	3343
Pediatric	138	78
Specialist	120	525

©2022 Serban N et al. *JAMA Network Open*.

Number of dentists

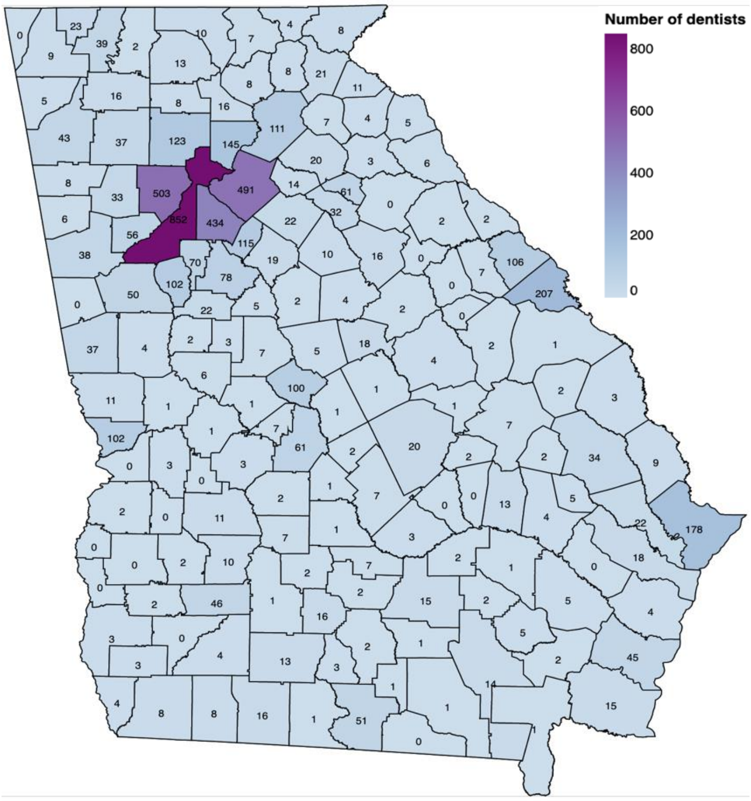

Number of dental hygienists

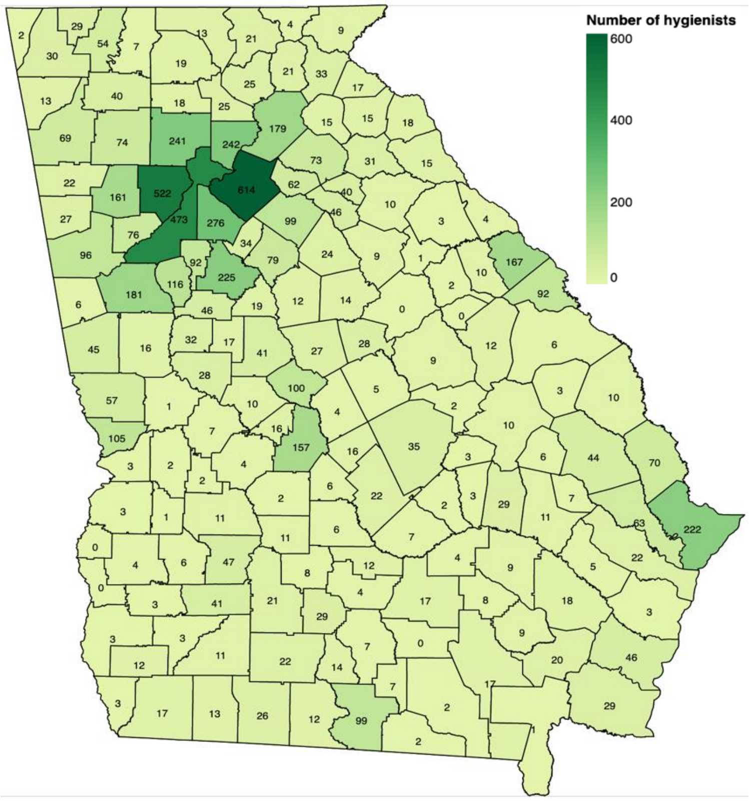

Number of dentists participating in Medicaid(+CHIP)

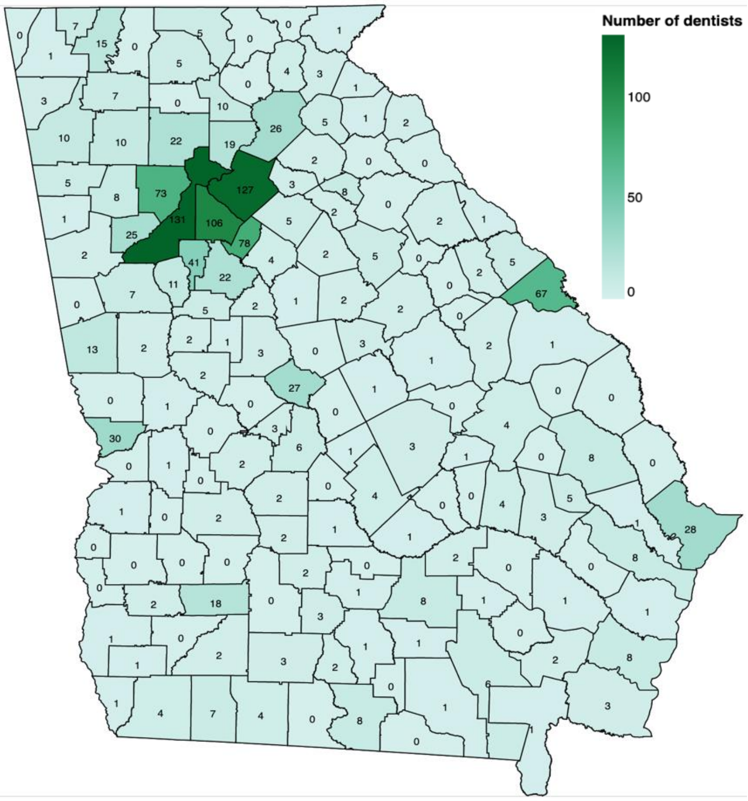

©2022 Serban N et al. *JAMA Network Op...*

Dental Care Supply
IDAHO

This state report summarizes data on the dental care supply, differentiated by type of insurance program, provider taxonomy, and rurality-urbanicity of practice address.

Percentage of dentists by provider taxonomy

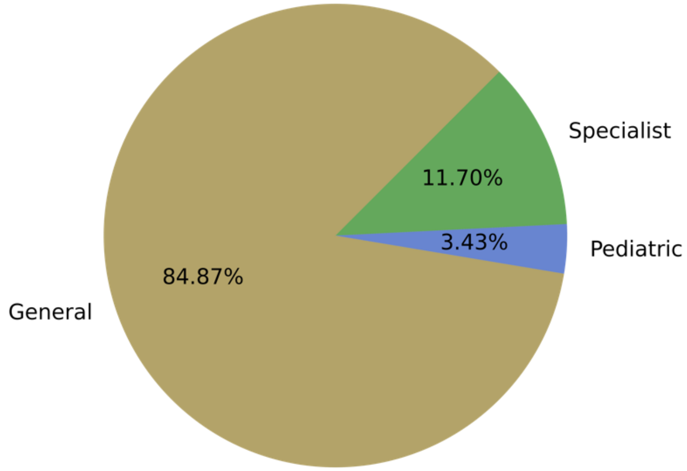

Number of dentists by rurality-urbanicity & provider taxonomy

Rurality-Urbanicity	General	Pediatric	Specialist
**Urban**	625	25	100
**Suburban**	169	8	15
**Rural**	97	3	8

Percentage of dentists by participation in public insurance programs

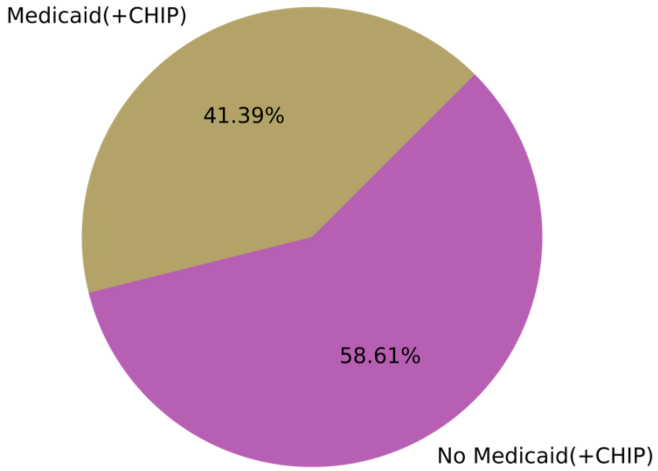

Number of dentists by rurality-urbanicity & participation in public insurance programs

Rurality-Urbanicity	Medicaid(+CHIP)	No Medicaid(+CHIP)
**Urban**	287	463
**Suburban**	102	90
**Rural**	45	63

Number of dentists by provider taxonomy & participation in public insurance programs

Provider Type	Medicaid(+CHIP)	No Medicaid(+CHIP)
**General**	368	524
**Pediatric**	29	7
**Specialist**	38	85

©2022 Serban N et al. *JAMA Network Open*.

Number of dentists

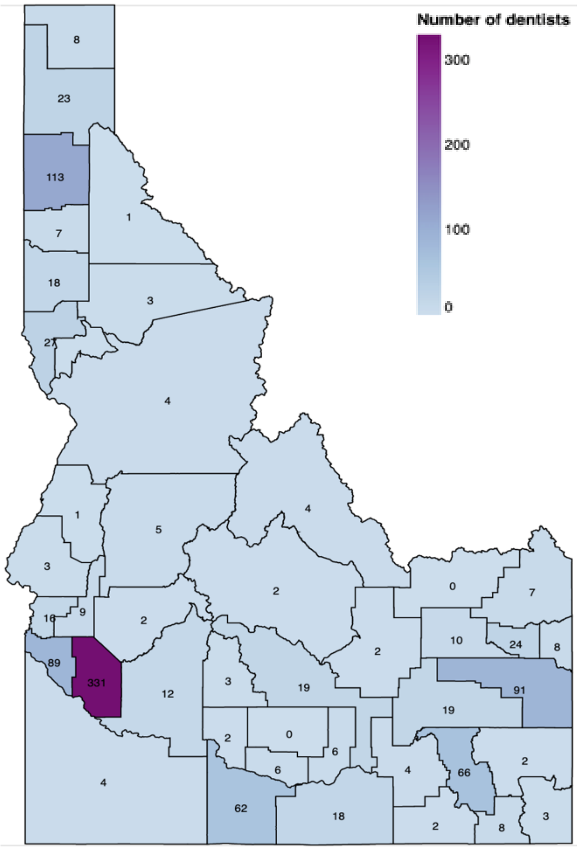

Number of dental hygienists

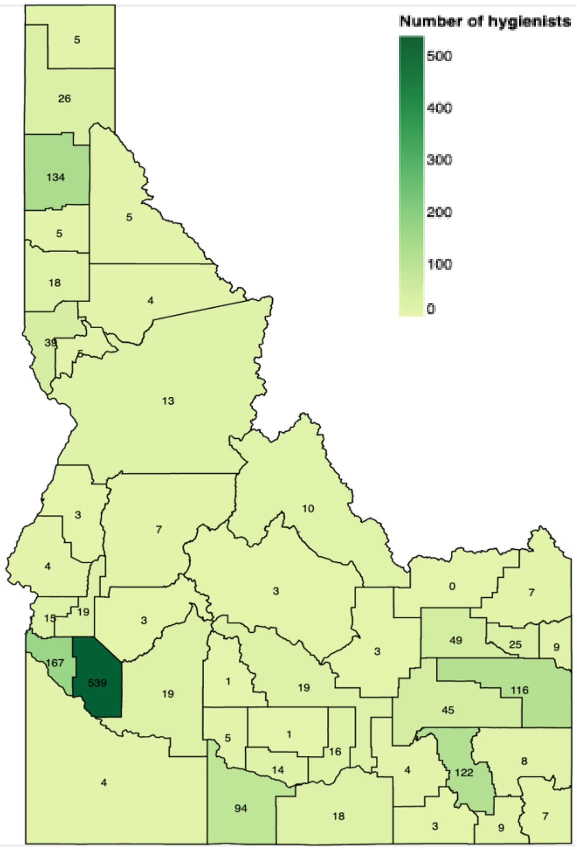

Number of dentists participating in Medicaid(+CHIP)

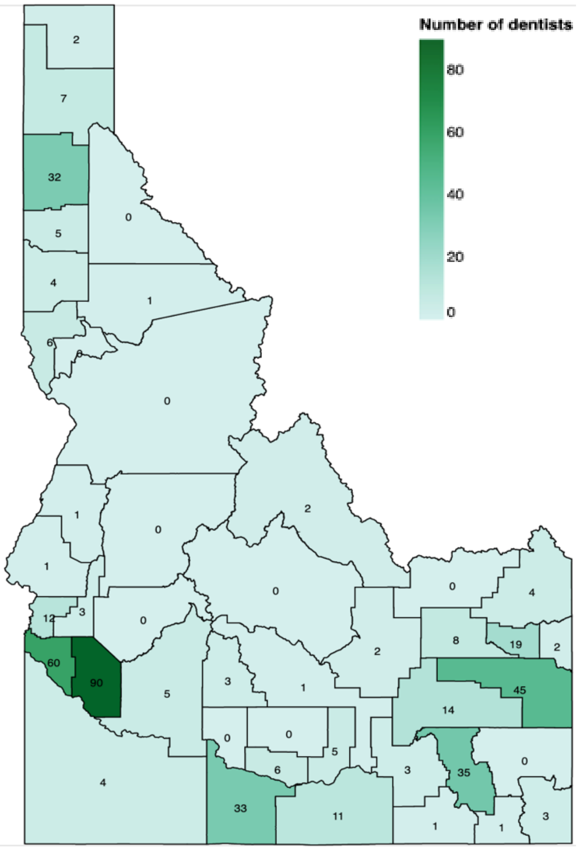

©2022 Serban N et al. *JAMA Network Open*.

Dental Care Supply
ILLINOIS

This state report summarizes data on the dental care supply, differentiated by type of insurance program, provider taxonomy, and rurality-urbanicity of practice address.

Percentage of dentists by provider taxonomy

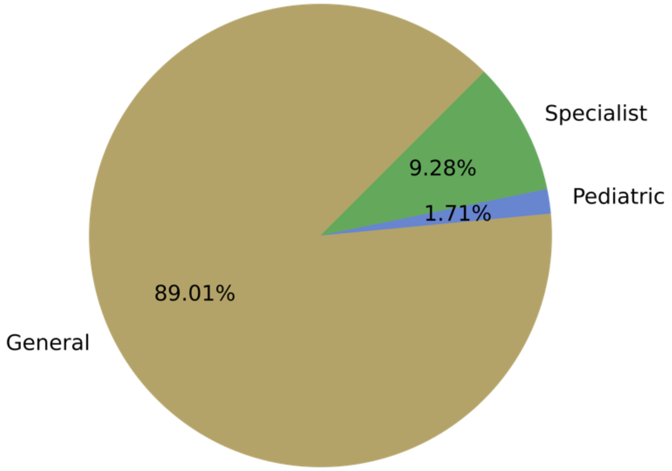

Number of dentists by rurality-urbanicity & provider taxonomy

Rurality-Urbanicity	General	Pediatric	Specialist
Urban	7700	152	827
Suburban	292	2	17
Rural	232	4	14

Percentage of dentists by participation in public insurance programs

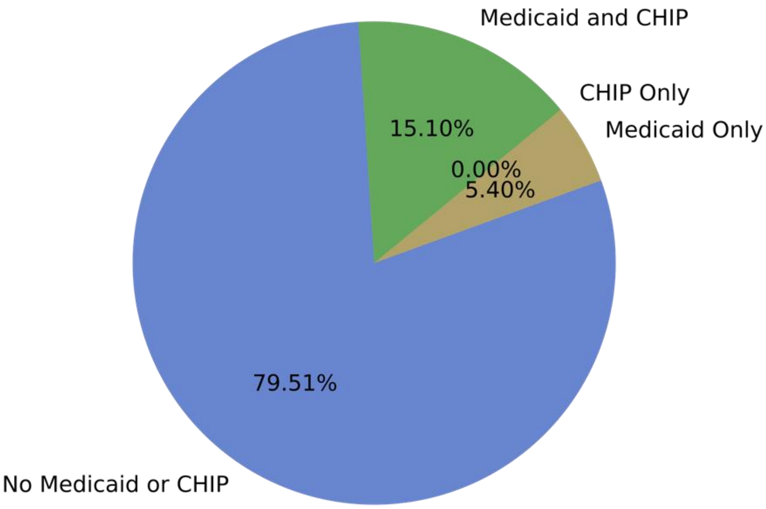

Number of dentists by rurality-urbanicity & participation in public insurance programs

Rurality-Urbanicity	Medicaid Only	CHIP Only	Medicaid and CHIP	No Medicaid or CHIP
Urban	486	0	1352	6856
Suburban	9	0	31	267
Rural	4	0	11	226

Number of dentists by provider taxonomy & participation in public insurance programs

Provider Type	Medicaid Only	CHIP Only	Medicaid and CHIP	No Medicaid or CHIP
General	465	0	1273	6494
Pediatric	4	0	32	122
Specialist	30	0	91	737

©2022 Serban N et al. *JAMA Network Open*.

Number of dentists

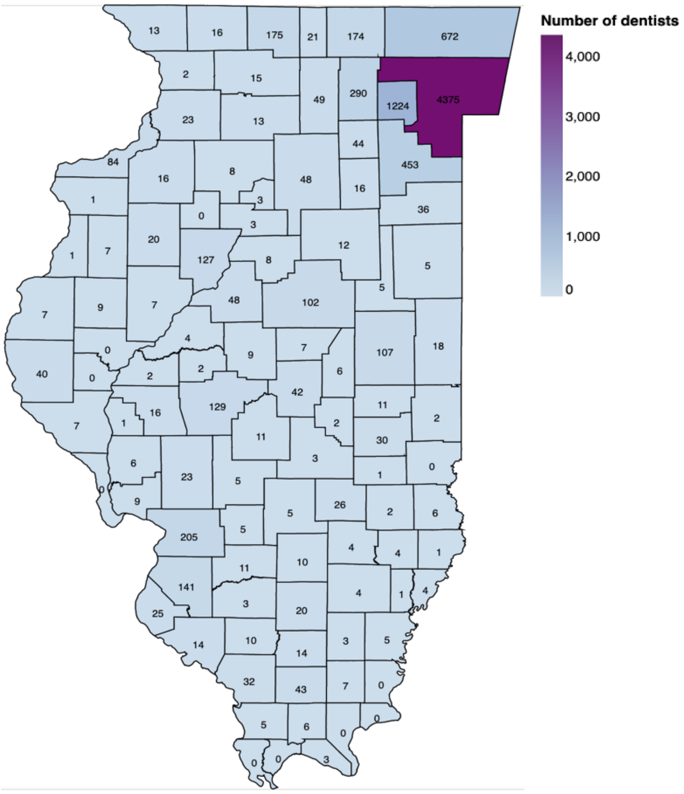

Number of dental hygienists

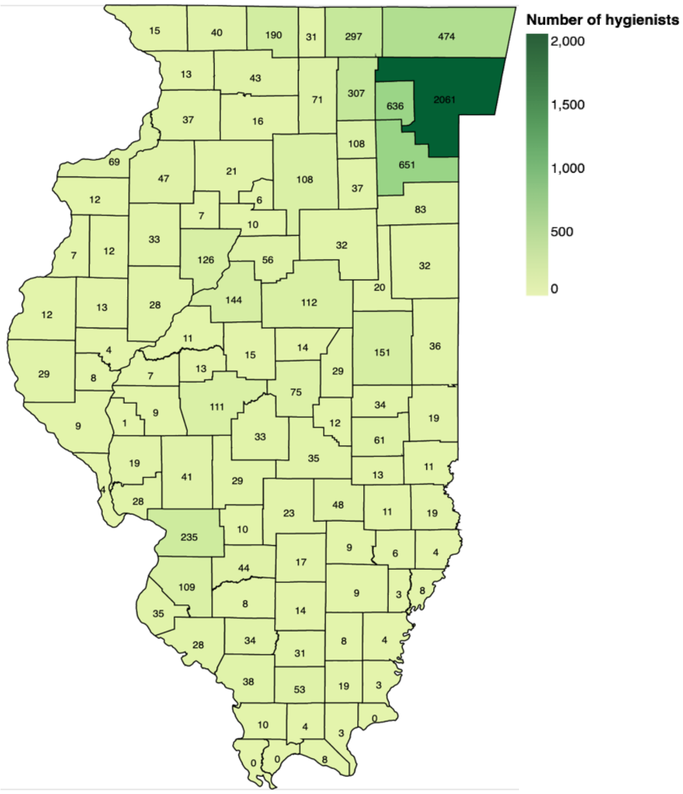

Number of dentists participating in Medicaid

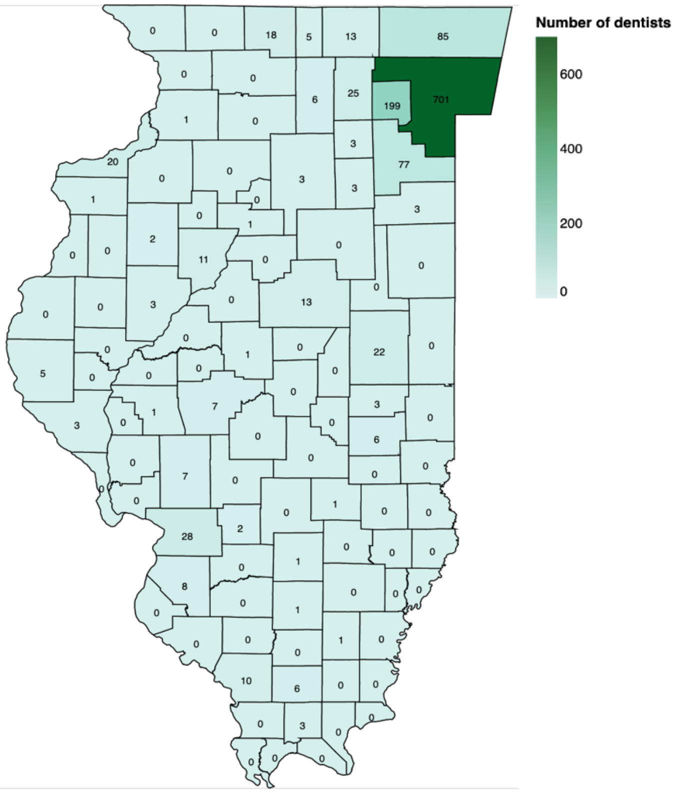

Number of dentists participating in

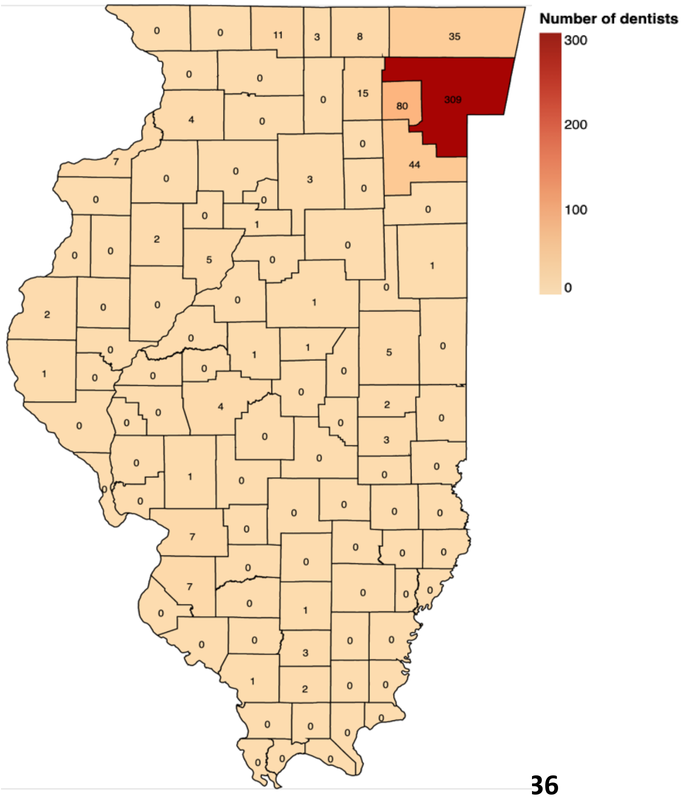

©2022 Serban N et al. *JAMA Network Open*.

Dental Care Supply
INDIANA

This state report summarizes data on the dental care supply, differentiated by type of insurance program, provider taxonomy, and rurality-urbanicity of practice address.

Percentage of dentists by provider taxonomy

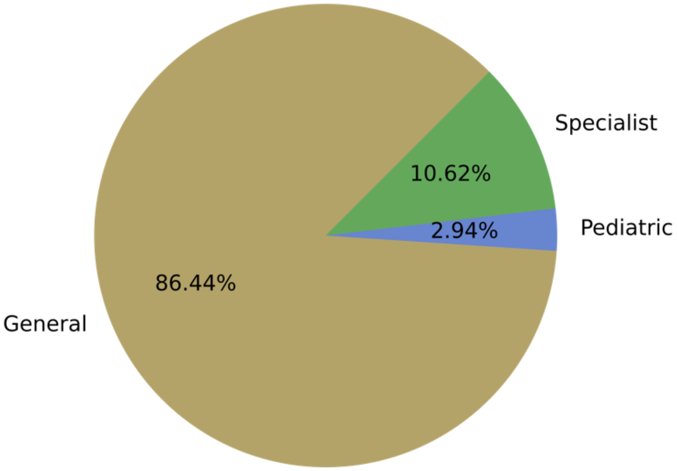

Number of dentists by rurality-urbanicity & provider taxonomy

Rurality-Urbanicity	General	Pediatric	Specialist
Urban	2529	96	347
Suburban	353	5	27
Rural	179	3	3

Percentage of dentists by participation in public insurance programs

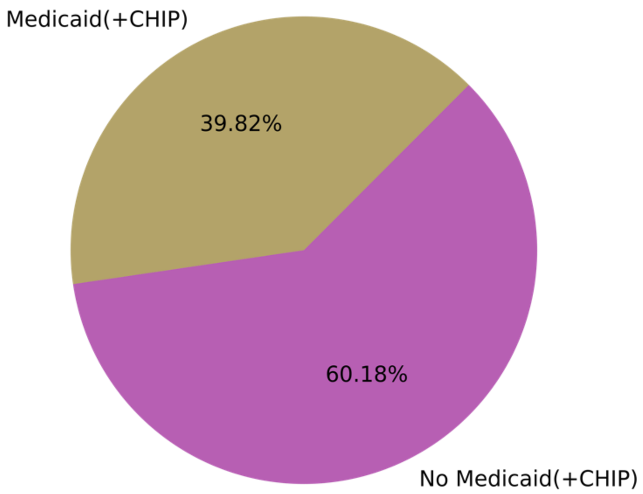

Number of dentists by rurality-urbanicity & participation in public insurance programs

Rurality-Urbanicity	Medicaid(+CHIP)	No Medicaid(+CHIP)
Urban	1120	1852
Suburban	197	187
Rural	93	92

Number of dentists by provider taxonomy & participation in public insurance programs

Provider Type	Medicaid(+CHIP)	No Medicaid(+CHIP)
General	1218	1843
Pediatric	63	41
Specialist	129	247

©2022 Serban N et al. *JAMA Network Open*.

Number of dentists

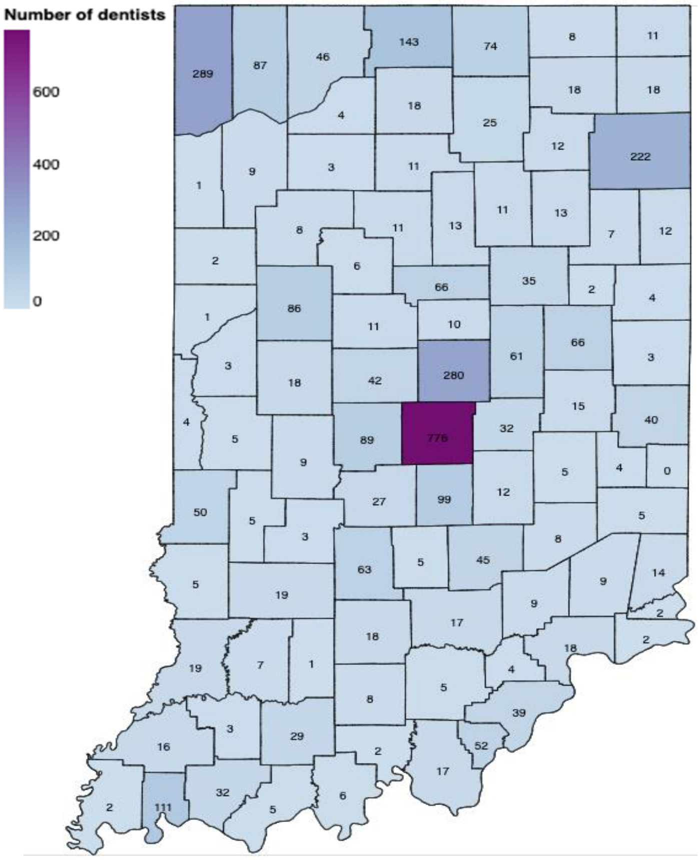

Number of dental hygienists

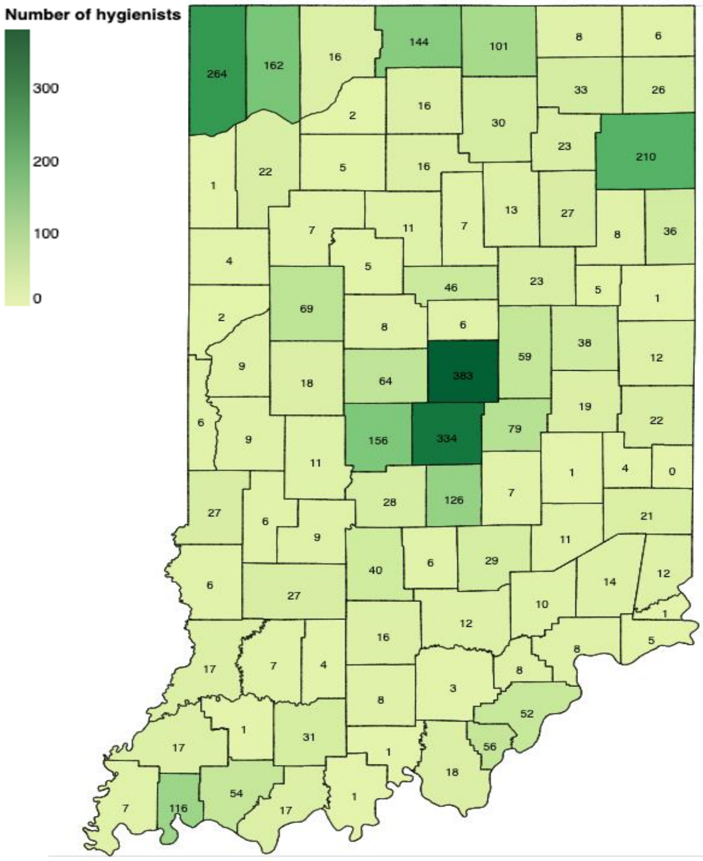

Number of dentists participating in Medicaid(+CHIP)

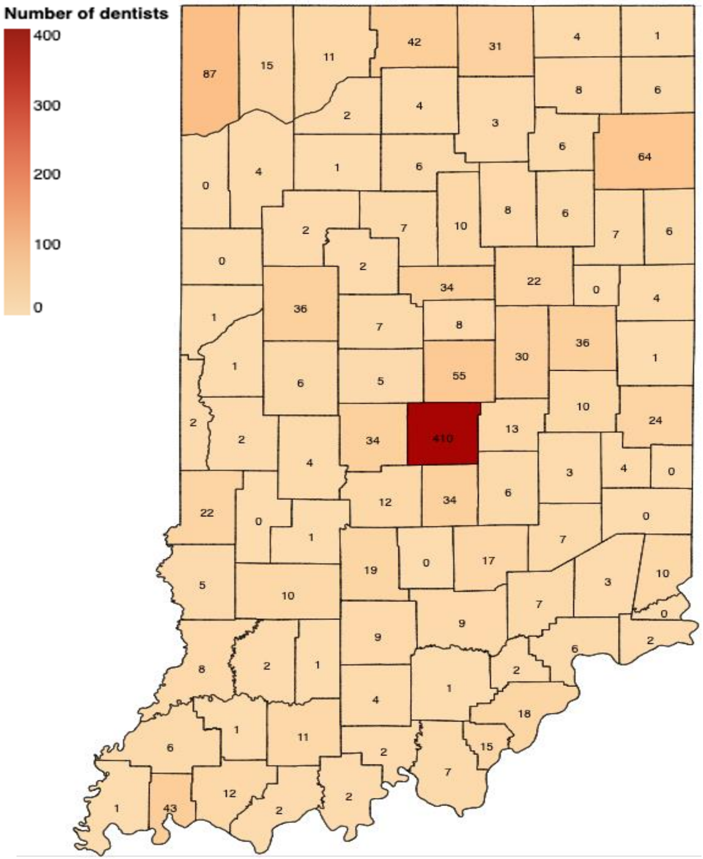

©2022 Serban N et al. *JAMA Network Open*.

Dental Care Supply
IOWA

This state report summarizes data on the dental care supply, differentiated by type of insurance program, provider taxonomy, and rurality-urbanicity of practice address.

Percentage of dentists by provider taxonomy

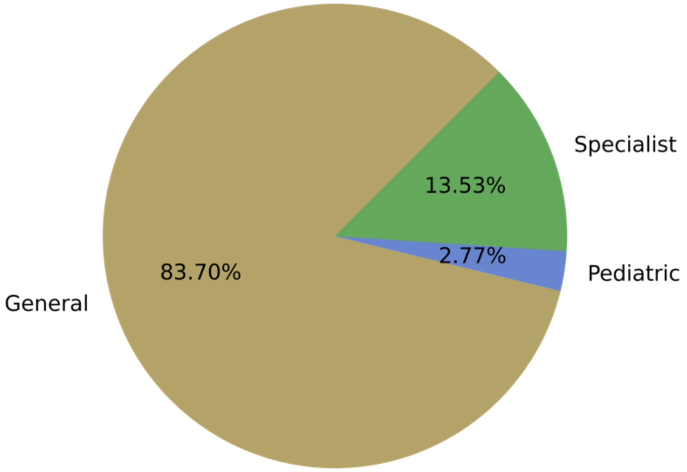

Number of dentists by rurality-urbanicity & provider taxonomy

Rurality-Urbanicity	General	Pediatric	Specialist
Urban	991	41	199
Suburban	186	6	26
Rural	341	4	18

Percentage of dentists by participation in public insurance programs

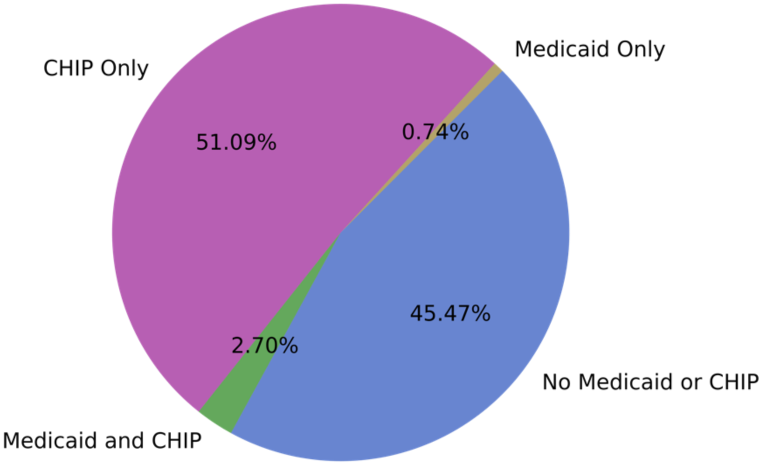

Number of dentists by rurality-urbanicity & participation in public insurance programs

Rurality-Urbanicity	Medicaid Only	CHIP Only	Medicaid and CHIP	No Medicaid or CHIP
Urban	8	573	31	651
Suburban	0	121	7	92
Rural	6	253	13	104

Number of dentists by provider taxonomy & participation in public insurance programs

Provider Type	Medicaid Only	CHIP Only	Medicaid and CHIP	No Medicaid or CHIP
General	14	805	37	723
Pediatric	0	37	4	13
Specialist	0	123	10	123

©2022 Serban N et al. *JAMA Network Open*.

Number of dentists

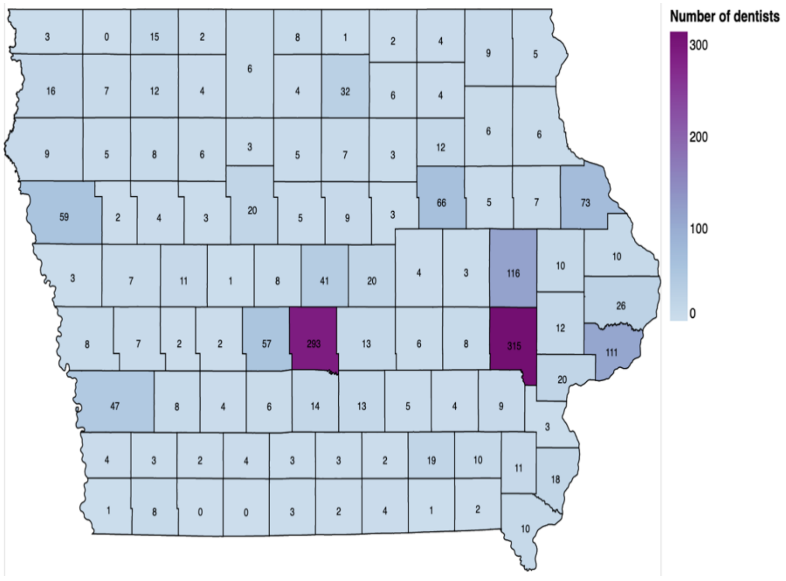

Number of dental hygienists

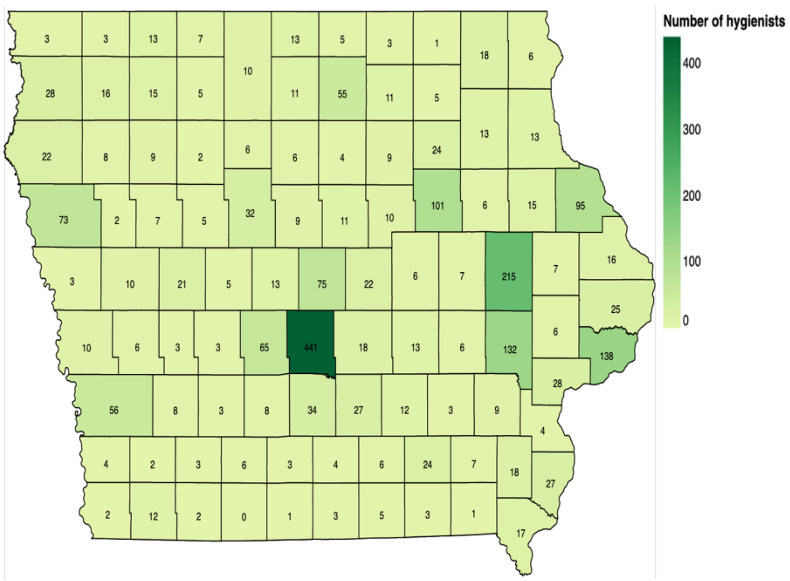

Number of dentists participating in Medicaid

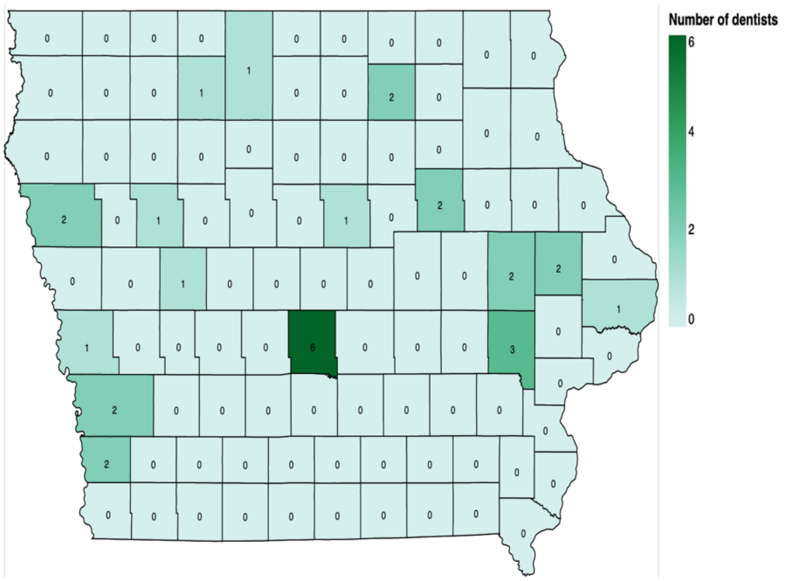

Number of dentists participating in CHIP

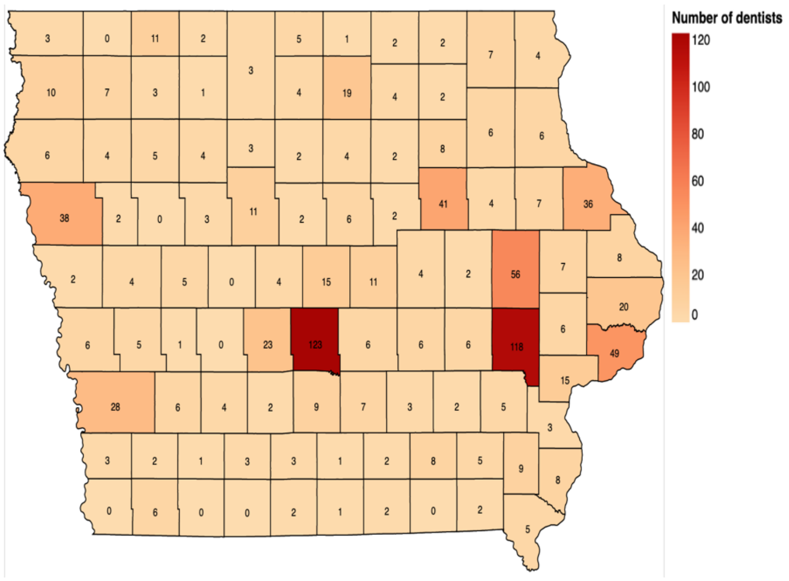

©2022 Serban N et al. *JAMA Network Open*.

Dental Care Supply
KANSAS

This state report summarizes data on the dental care supply, differentiated by type of insurance program, provider taxonomy, and rurality-urbanicity of practice address.

Percentage of dentists by provider taxonomy

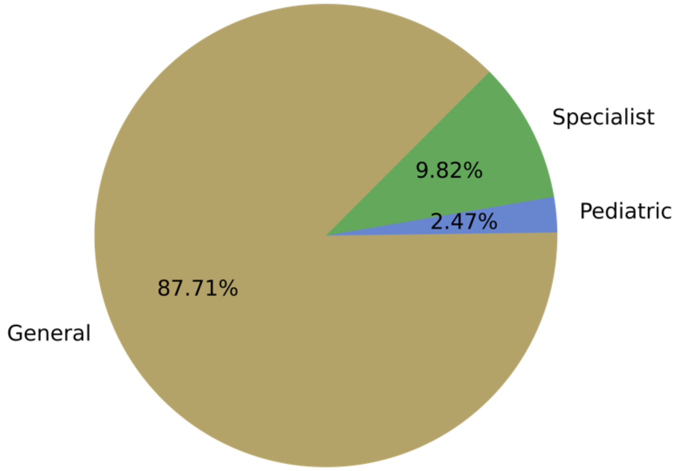

Number of dentists by rurality-urbanicity & provider taxonomy

Rurality-Urbanicity	General	Pediatric	Specialist
Urban	958	37	124
Suburban	238	1	25
Rural	154	0	2

Percentage of dentists by participation in public insurance programs

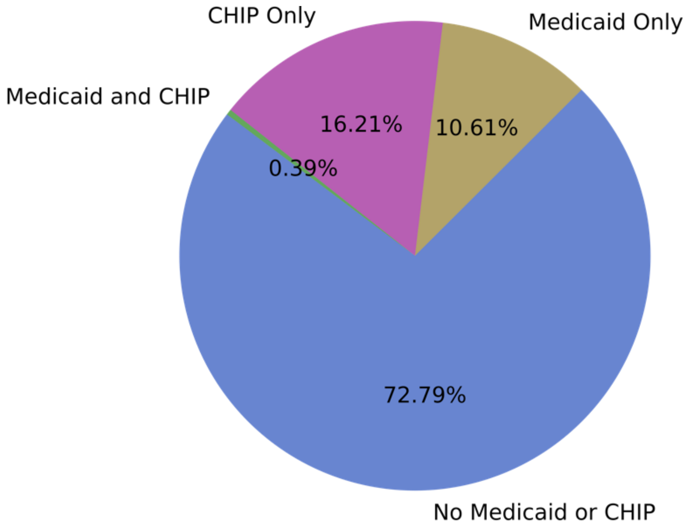

Number of dentists by rurality-urbanicity & participation in public insurance programs

Rurality-Urbanicity	Medicaid Only	CHIP Only	Medicaid and CHIP	No Medicaid or CHIP
Urban	100	138	2	878
Suburban	36	59	4	164
Rural	28	52	0	76

Number of dentists by provider taxonomy & participation in public insurance programs

Provider Type	Medicaid Only	CHIP Only	Medicaid and CHIP	No Medicaid or CHIP
General	131	216	6	994
Pediatric	10	14	0	14
Specialist	22	19	0	110

©2022 Serban N et al. *JAMA Network Open*.

Number of dentists

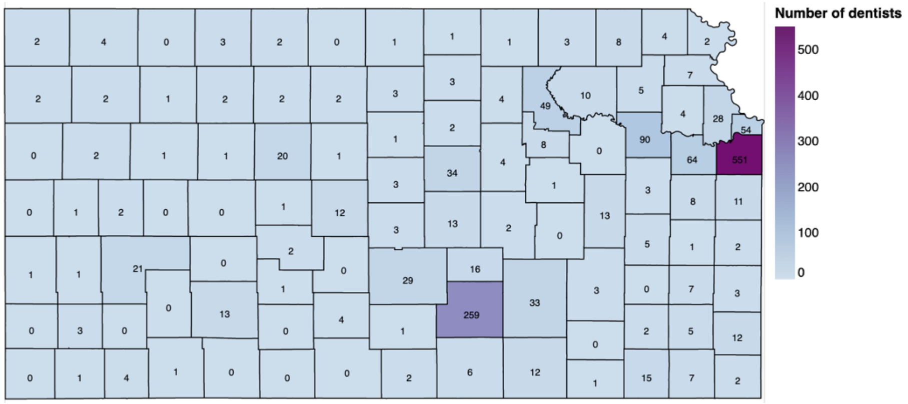

Number of dental hygienists

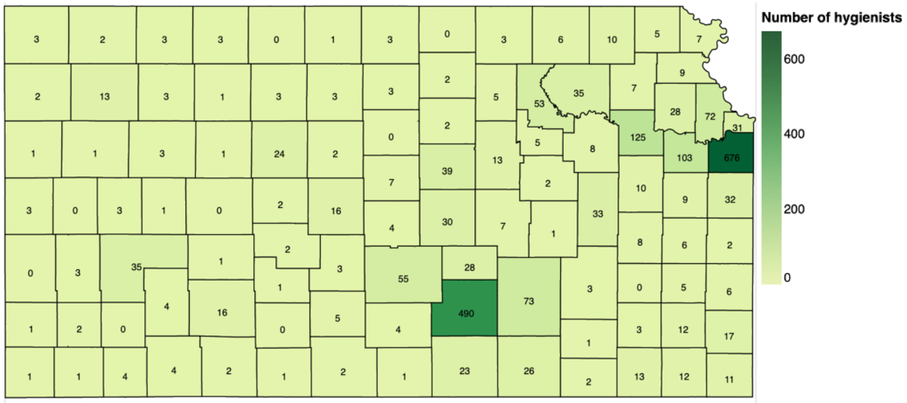

Number of dentists participating in Medicaid

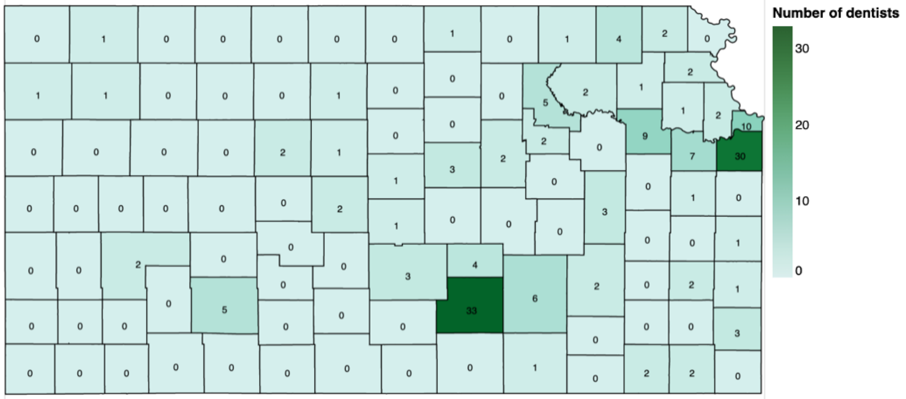

Number of dentists participating in CHIP

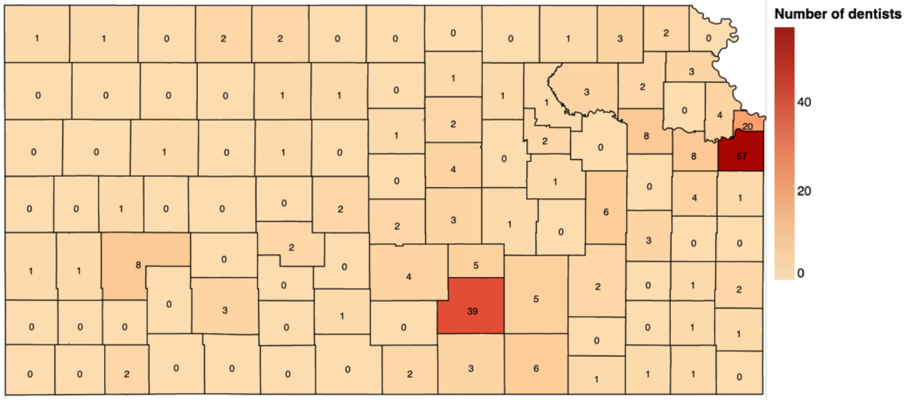

©2022 Serban N et al. *JAMA Network Open*.

Dental Care Supply
KENTUCKY

This state report summarizes data on the dental care supply, differentiated by type of insurance program, provider taxonomy, and rurality-urbanicity of practice address.

Percentage of dentists by provider taxonomy

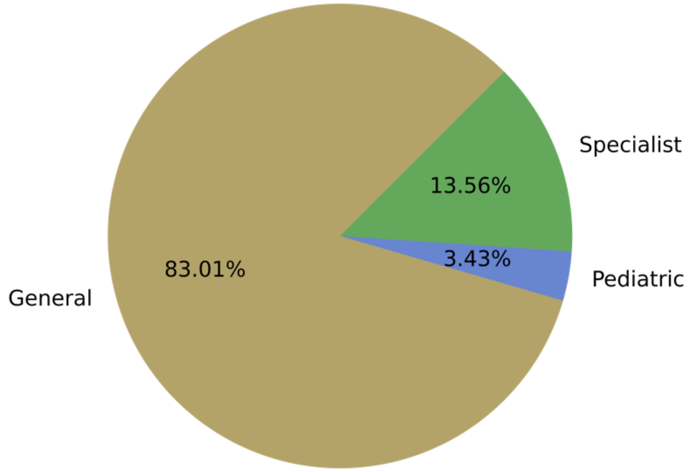

Number of dentists by rurality-urbanicity & provider taxonomy

Rurality-Urbanicity	General	Pediatric	Specialist
Urban	1395	55	254
Suburban	400	25	71
Rural	285	6	15

Percentage of dentists by participation in public insurance programs

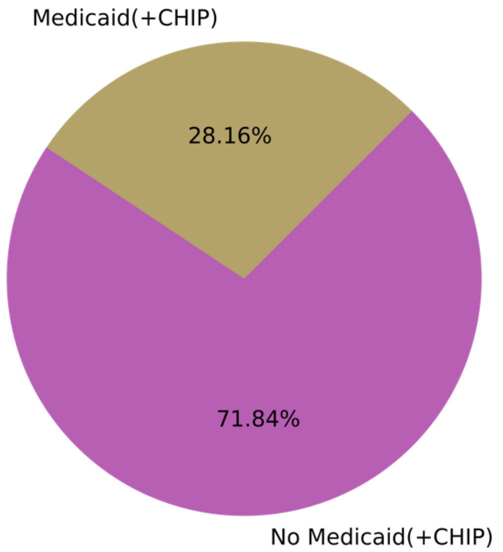

Number of dentists by rurality-urbanicity & participation in public insurance programs

Rurality-Urbanicity	Medicaid(+CHIP)	No Medicaid(+CHIP)
Urban	346	1357
Suburban	179	318
Rural	181	125

Number of dentists by provider taxonomy & participation in public insurance programs

Provider Type	Medicaid(+CHIP)	No Medicaid(+CHIP)
General	575	1506
Pediatric	49	37
Specialist	82	258

©2022 Serban N et al. *JAMA Network Open*.

Number of dentists

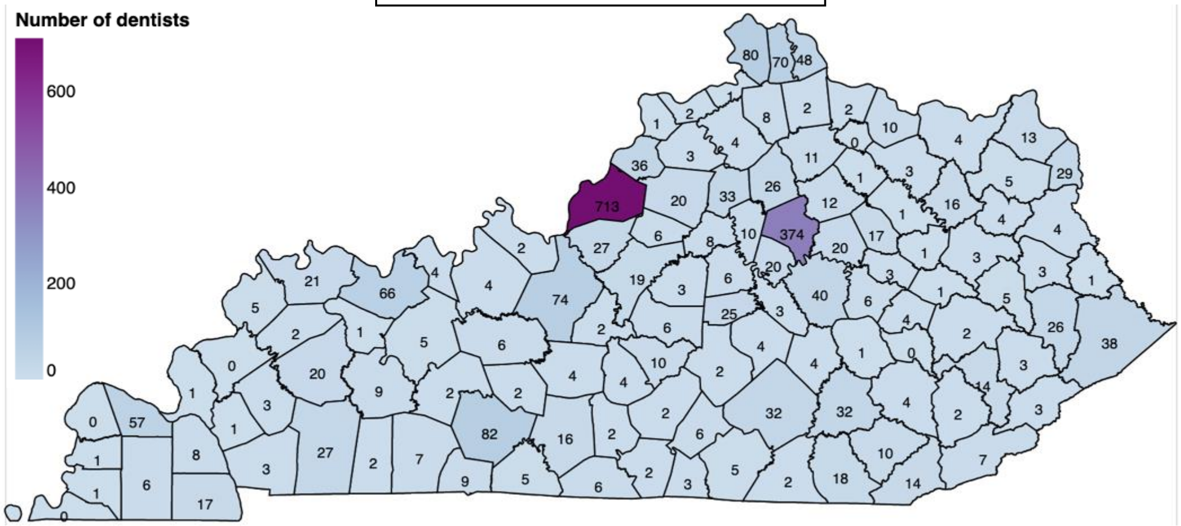

Number of dental hygienists

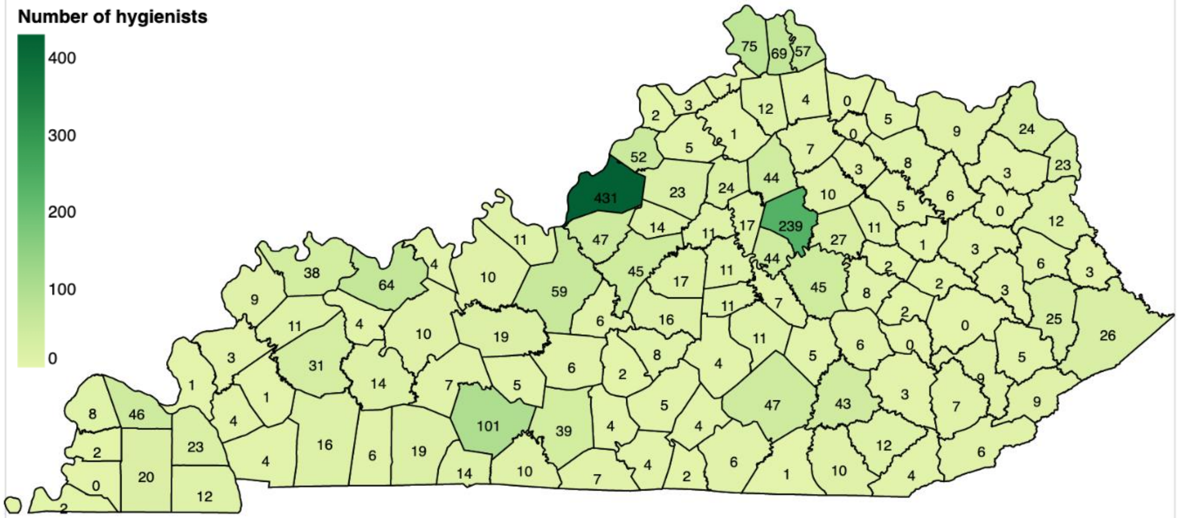

Number of dentists participating in Medicaid(+CHIP)

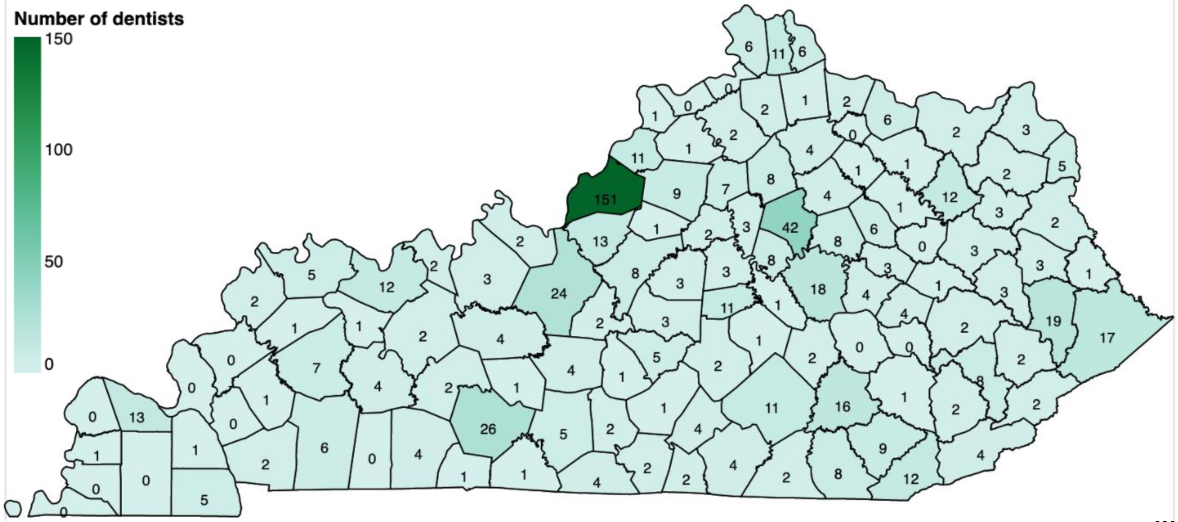

©2022 Serban N et al. *JAMA Network Open*.

Dental Care Supply
LOUISIANA

This state report summarizes data on the dental care supply, differentiated by type of insurance program, provider taxonomy, and rurality-urbanicity of practice address.

Percentage of dentists by provider taxonomy

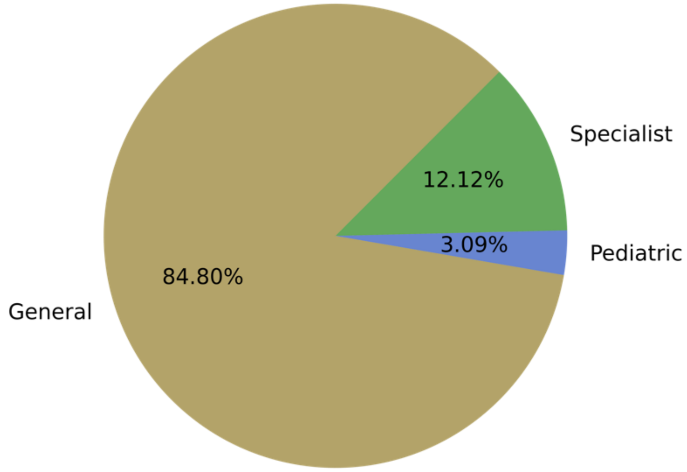

Number of dentists by rurality-urbanicity & provider taxonomy

Rurality-Urbanicity	General	Pediatric	Specialist
Urban	1731	65	261
Suburban	112	4	11
Rural	81	1	3

Percentage of dentists by participation in public insurance programs

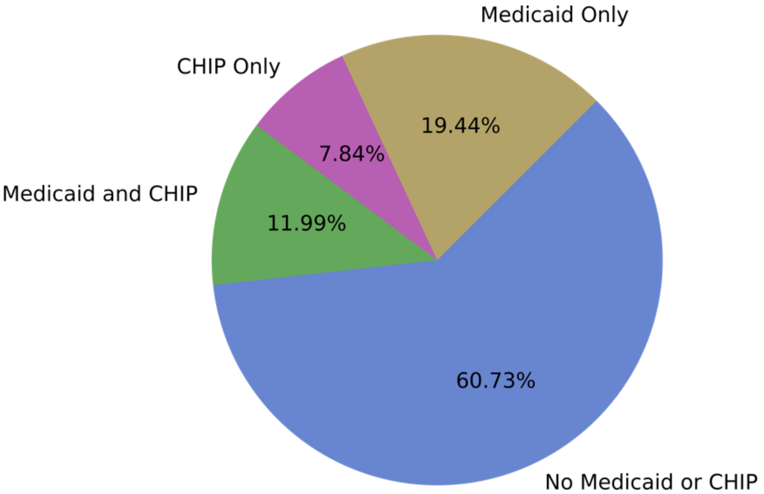

Number of dentists by rurality-urbanicity & participation in public insurance programs

Rurality-Urbanicity	Medicaid Only	CHIP Only	Medicaid and CHIP	No Medicaid or CHIP
Urban	363	156	245	1292
Suburban	38	17	12	59
Rural	39	5	14	27

Number of dentists by provider taxonomy & participation in public insurance programs

Provider Type	Medicaid Only	CHIP Only	Medicaid and CHIP	No Medicaid or CHIP
General	386	155	217	1166
Pediatric	15	6	33	16
Specialist	40	17	22	196

©2022 Serban N et al. *JAMA Network Open*.

Number of dentists

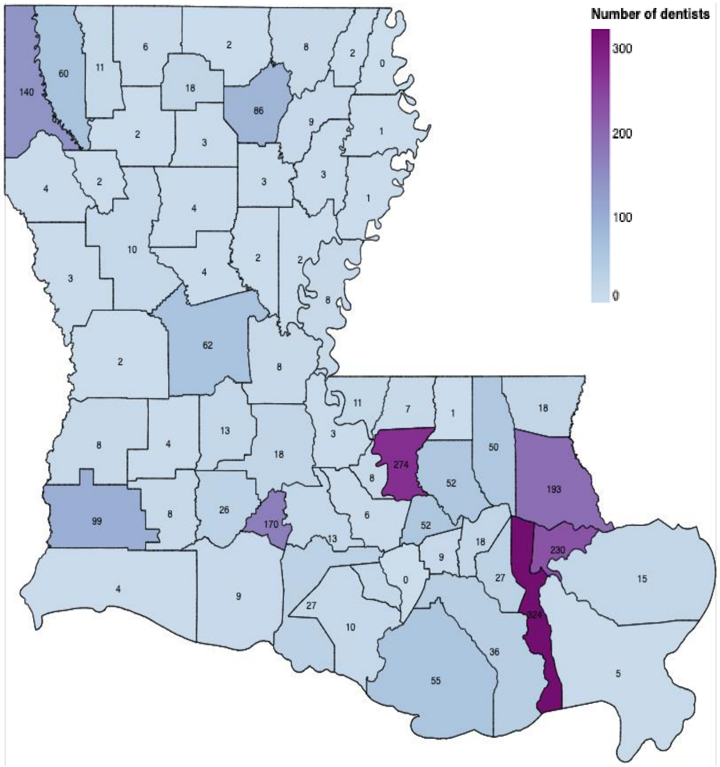

Number of dental hygienists

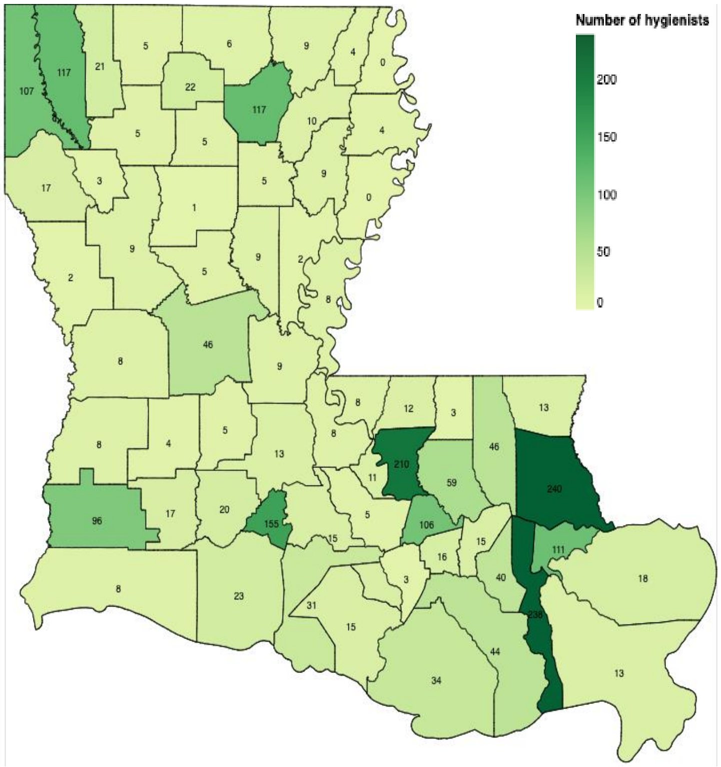

Number of dentists participating in Medicaid

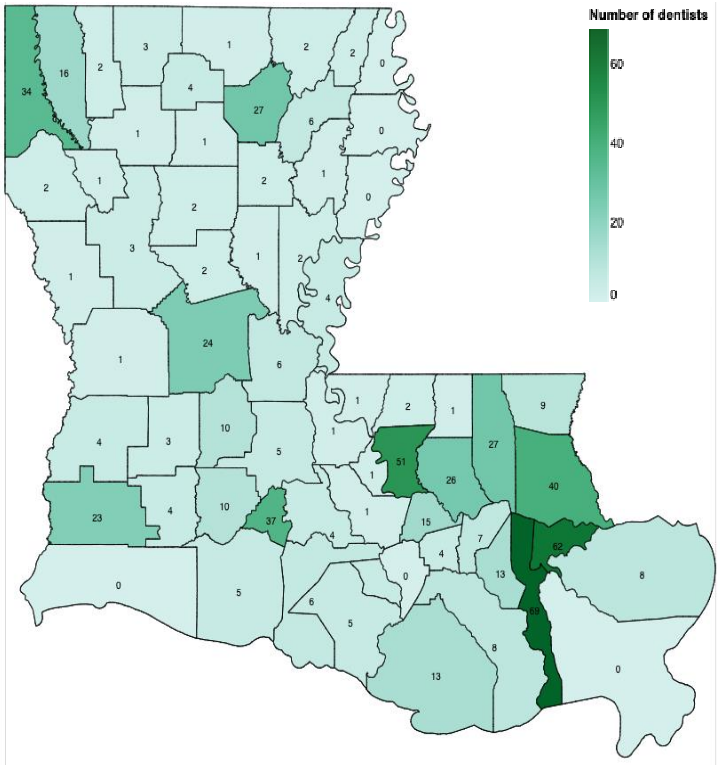

Number of dentists participating in CHIP

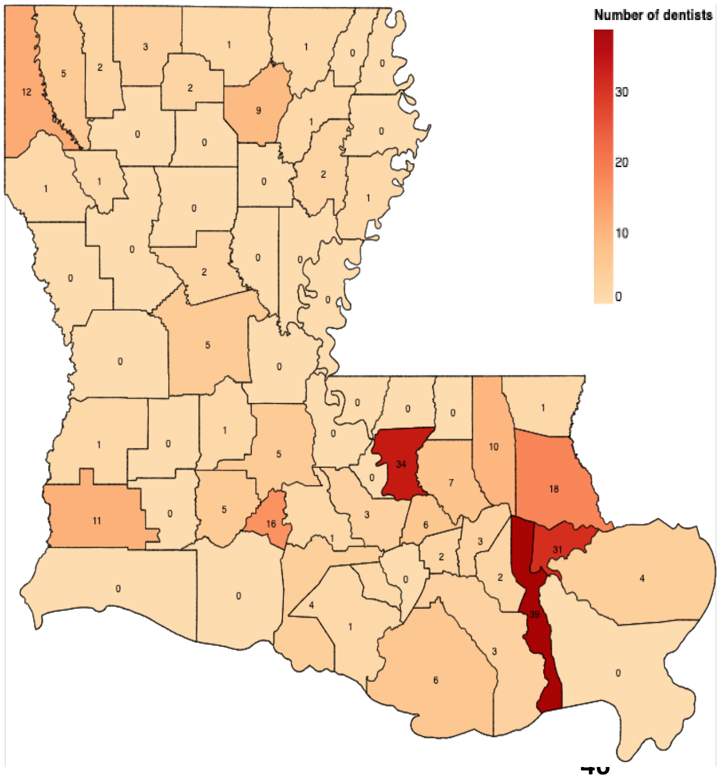

©2022 Serban N et al. *JAMA Network Open*.

Dental Care Supply
MAINE

This state report summarizes data on the dental care supply, differentiated by type of insurance program, provider taxonomy, and rurality-urbanicity of practice address.

Percentage of dentists by provider taxonomy

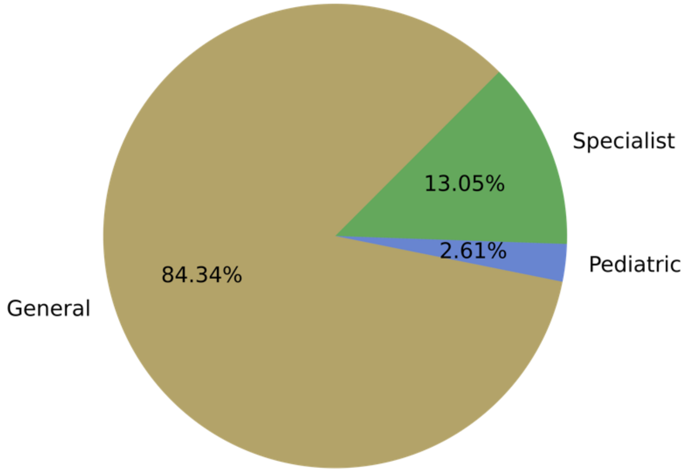

Number of dentists by rurality-urbanicity & provider taxonomy

Rurality-Urbanicity	General	Pediatric	Specialist
**Urban**	359	12	66
**Suburban**	98	4	12
**Rural**	158	3	17

Percentage of dentists by participation in public insurance programs

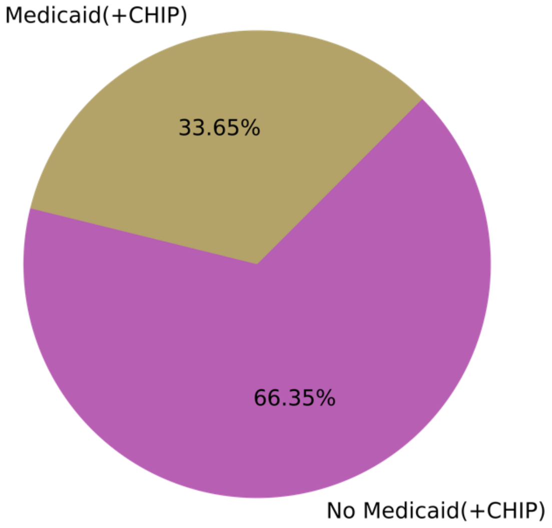

Number of dentists by rurality-urbanicity & participation in public insurance programs

Rurality-Urbanicity	Medicaid(+CHIP)	No Medicaid(+CHIP)
**Urban**	128	309
**Suburban**	44	70
**Rural**	73	104

Number of dentists by provider taxonomy & participation in public insurance programs

Provider Type	Medicaid(+CHIP)	No Medicaid(+CHIP)
**General**	193	421
**Pediatric**	11	8
**Specialist**	41	54

©2022 Serban N et al. *JAMA Network Open*.

Number of dentists

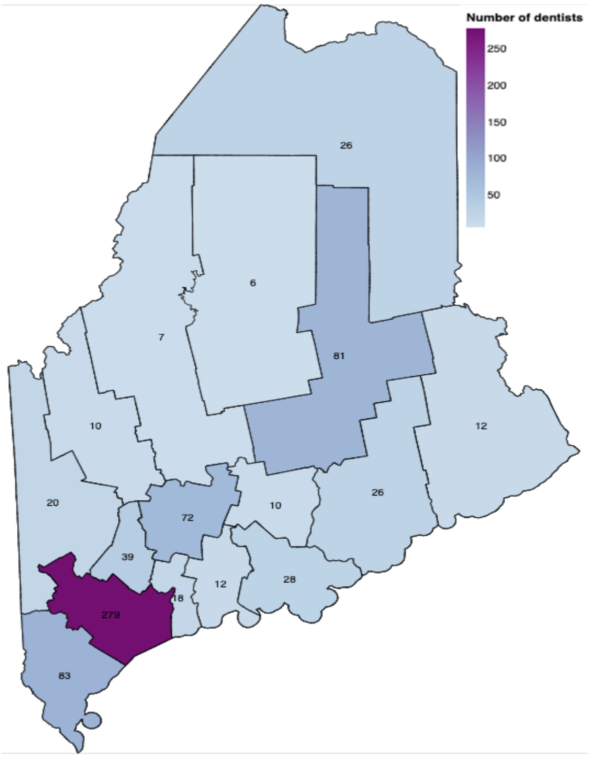

Number of dental hygienists

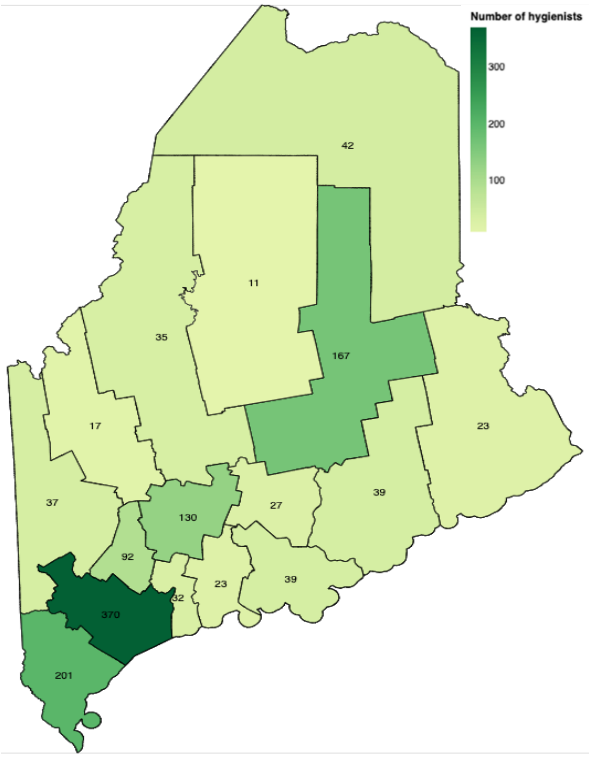

Number of dentists participating in Medicaid(+CHIP)

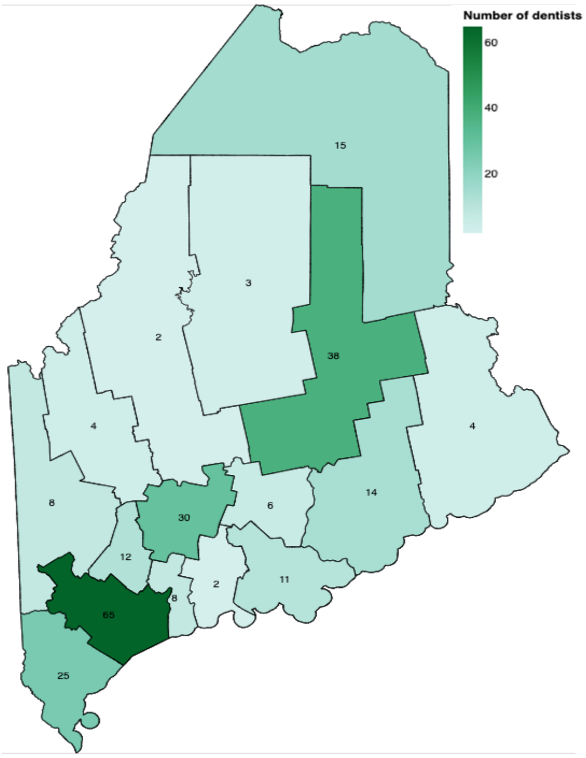

©2022 Serban N et al. *JAMA Network Open*.

Dental Care Supply
MARYLAND

This state report summarizes data on the dental care supply, differentiated by type of insurance program, provider taxonomy, and rurality-urbanicity of practice address.

Percentage of dentists by provider taxonomy

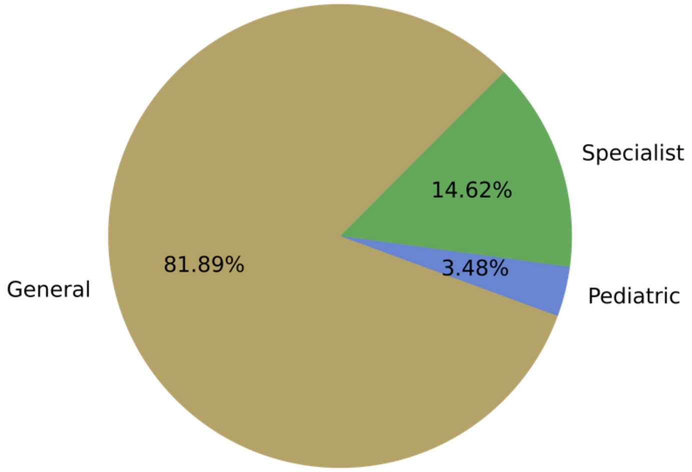

Number of dentists by rurality-urbanicity & provider taxonomy

Rurality-Urbanicity	General	Pediatric	Specialist
Urban	3434	147	619
Suburban	65	3	11
Rural	29	0	0

Percentage of dentists by participation in public insurance programs

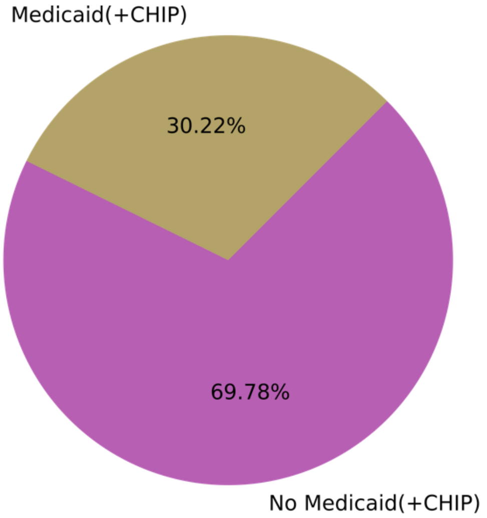

Number of dentists by rurality-urbanicity & participation in public insurance programs

Rurality-Urbanicity	Medicaid(+CHIP)	No Medicaid(+CHIP)
Urban	1273	2927
Suburban	15	64
Rural	14	15

Number of dentists by provider taxonomy & participation in public insurance programs

Provider Type	Medicaid(+CHIP)	No Medicaid(+CHIP)
General	1026	2502
Pediatric	97	53
Specialist	179	451

©2022 Serban N et al. *JAMA Network Open*.

Number of dentists

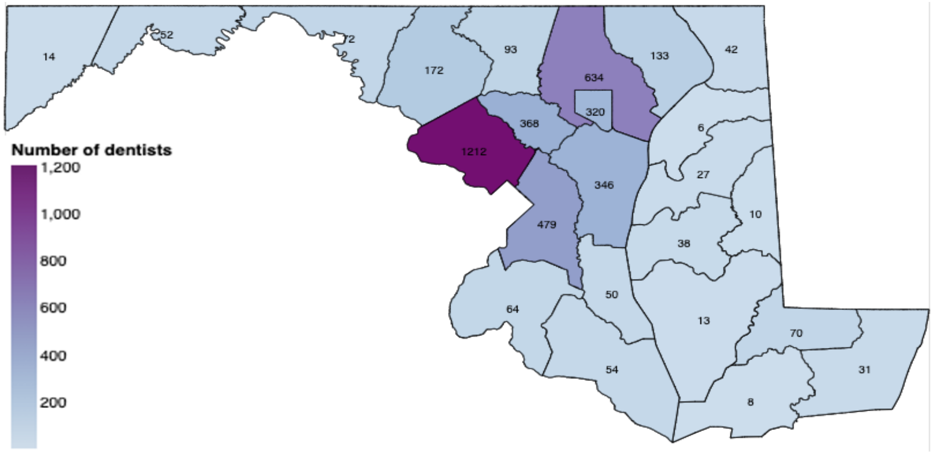

Number of dental hygienists

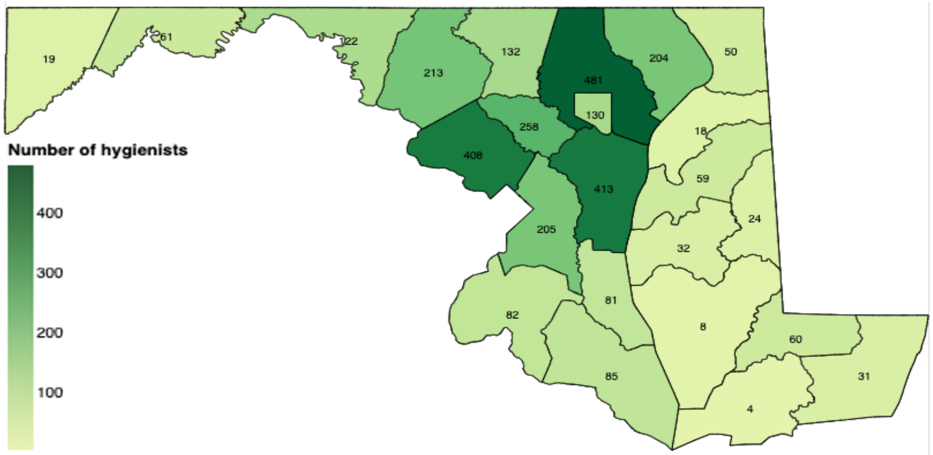

Number of dentists participating in Medicaid(+CHIP)

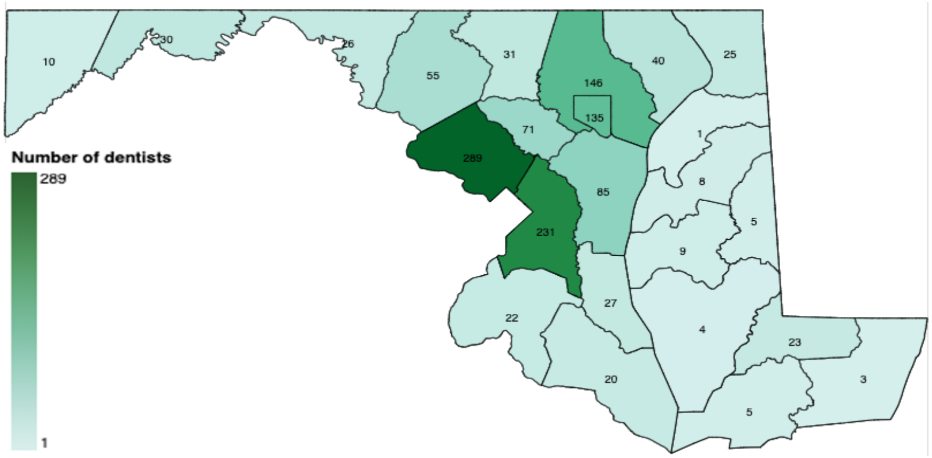

©2022 Serban N et al. *JAMA Network Open*.

Dental Care Supply
MASSACHUSETTS

This state report summarizes data on the dental care supply, differentiated by type of insurance program, provider taxonomy, and rurality-urbanicity of practice address.

Percentage of dentists by provider taxonomy

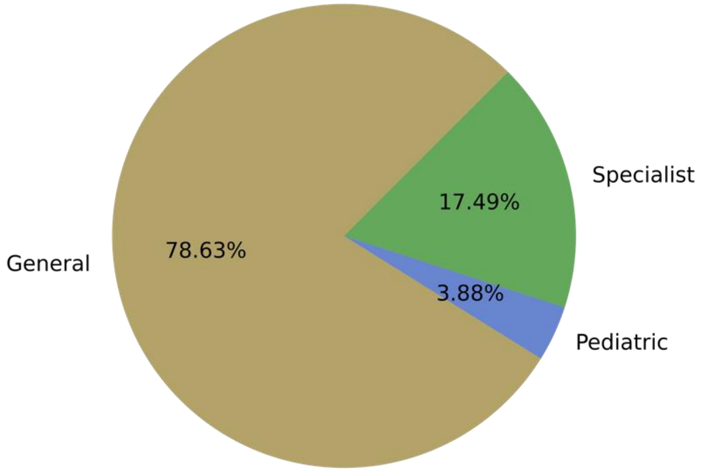

Number of dentists by rurality-urbanicity & provider taxonomy

Rurality-Urbanicity	General	Pediatric	Specialist
**Urban**	4647	230	1034
**Suburban**	59	2	11
**Rural**	34	2	8

Percentage of dentists by participation in public insurance programs

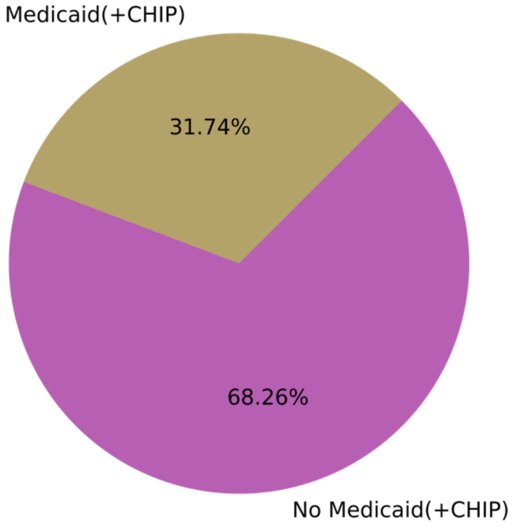

Number of dentists by rurality-urbanicity & participation in public insurance programs

Rurality-Urbanicity	Medicaid(+CHIP)	No Medicaid(+CHIP)
**Urban**	1875	4037
**Suburban**	29	43
**Rural**	9	35

Number of dentists by provider taxonomy & participation in public insurance programs

Provider Type	Medicaid(+CHIP)	No Medicaid(+CHIP)
**General**	1515	3225
**Pediatric**	143	91
**Specialist**	255	799

©2022 Serban N et al. *JAMA Network Open*.

Number of dentists

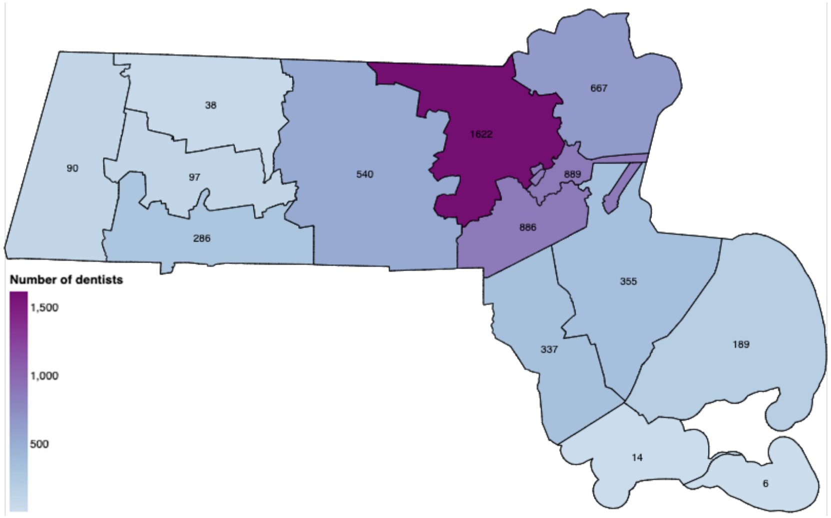

Number of dental hygienists

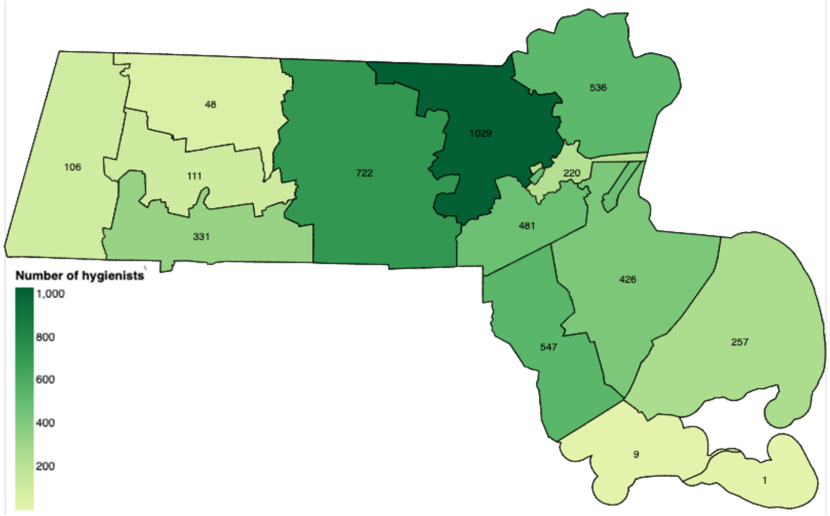

Number of dentists participating in Medicaid(+CHIP)

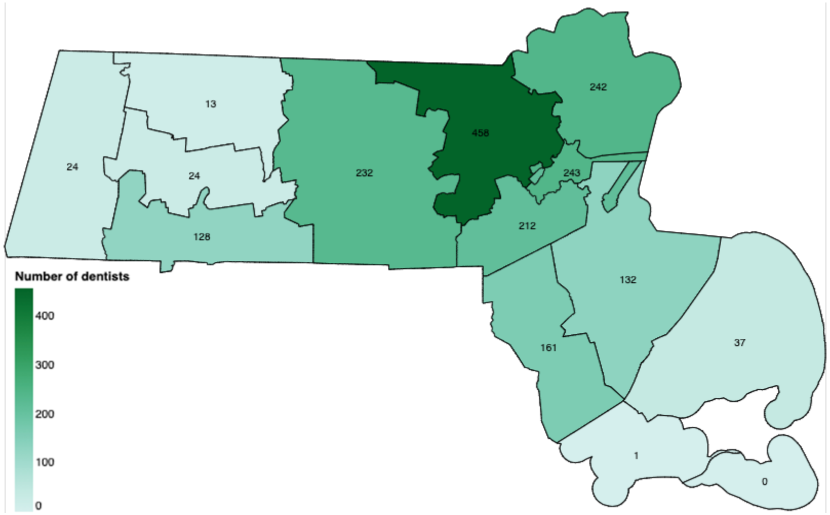

©2022 Serban N et al. *JAMA Network Open*.

Dental Care Supply
MICHIGAN

This state report summarizes data on the dental care supply, differentiated by type of insurance program, provider taxonomy, and rurality-urbanicity of practice address.

Percentage of dentists by provider taxonomy

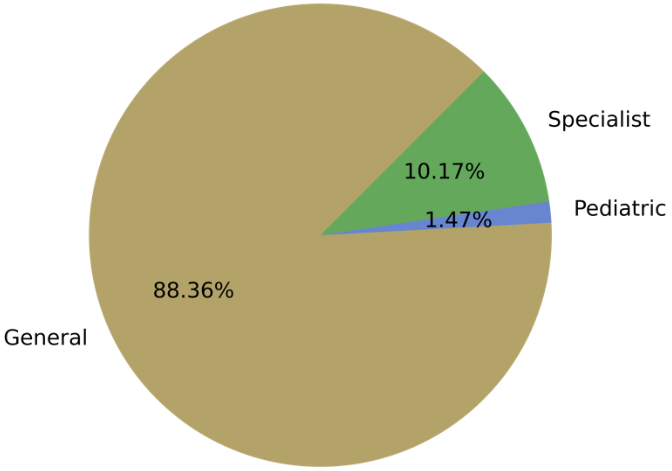

Number of dentists by rurality-urbanicity & provider taxonomy

Rurality-Urbanicity	General	Pediatric	Specialist
Urban	5304	94	636
Suburban	441	6	48
Rural	389	2	21

Percentage of dentists by participation in public insurance programs

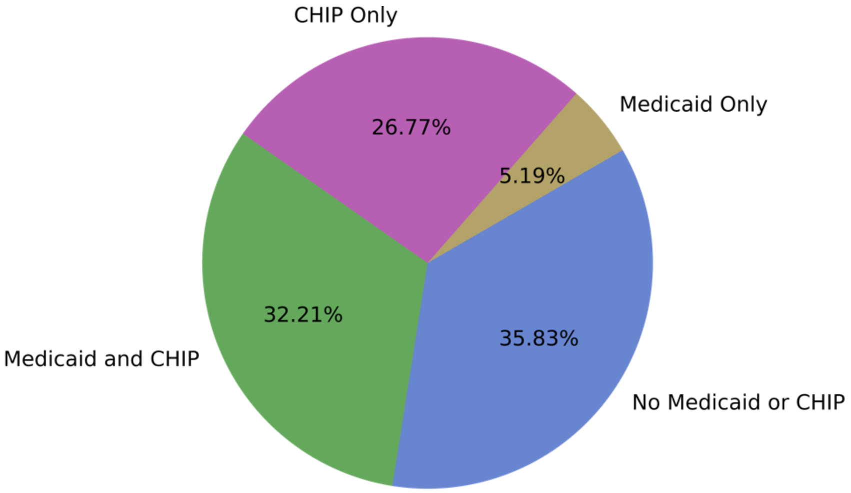

Number of dentists by rurality-urbanicity & participation in public insurance programs

Rurality-Urbanicity	Medicaid Only	CHIP Only	Medicaid and CHIP	No Medicaid or CHIP
Urban	295	1607	1973	2168
Suburban	34	123	137	189
Rural	31	128	127	129

Number of dentists by provider taxonomy & participation in public insurance programs

Provider Type	Medicaid Only	CHIP Only	Medicaid and CHIP	No Medicaid or CHIP
General	284	1773	2060	2017
Pediatric	6	10	63	23
Specialist	70	75	113	447

©2022 Serban N et al. *JAMA Network Open*.

Number of dentists

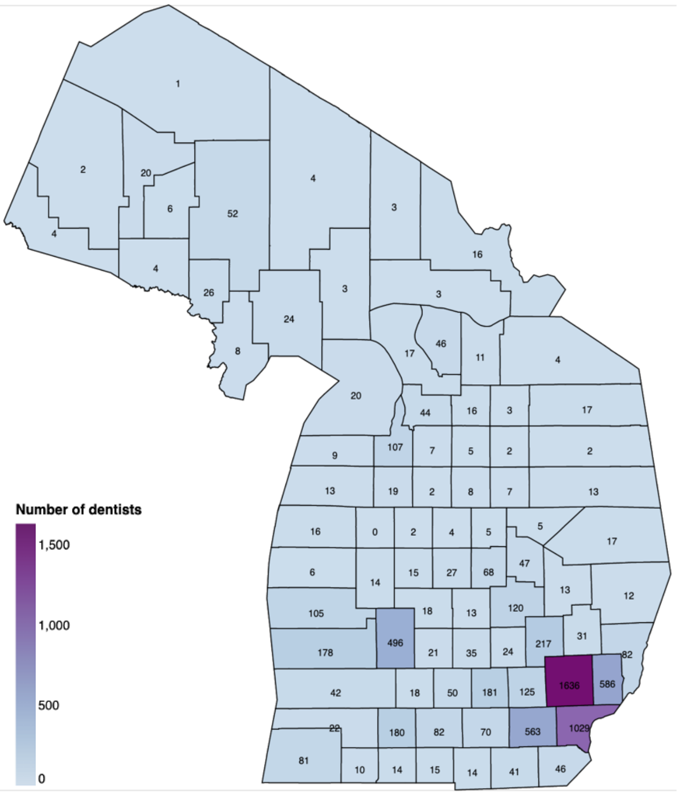

Number of dental hygienists

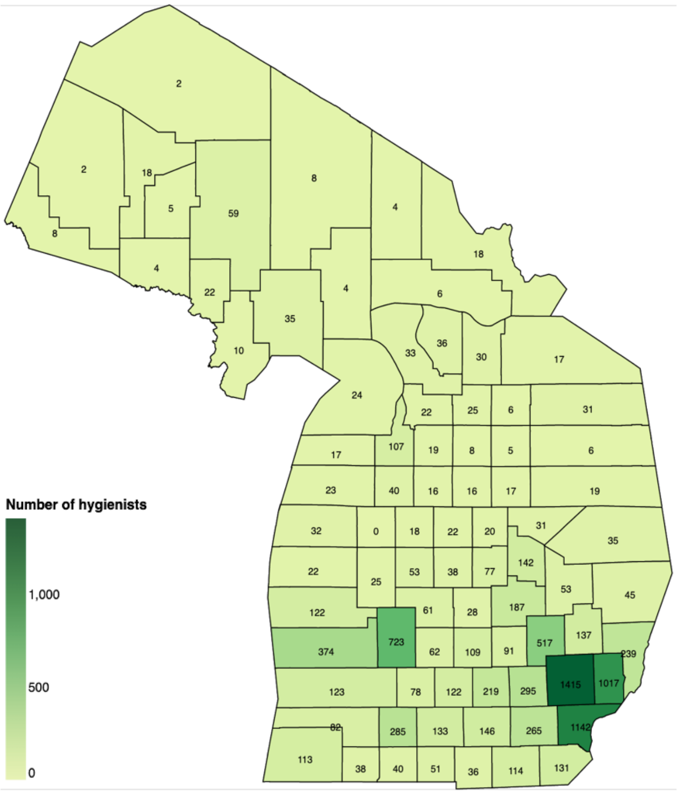

Number of dentists participating in Medicaid

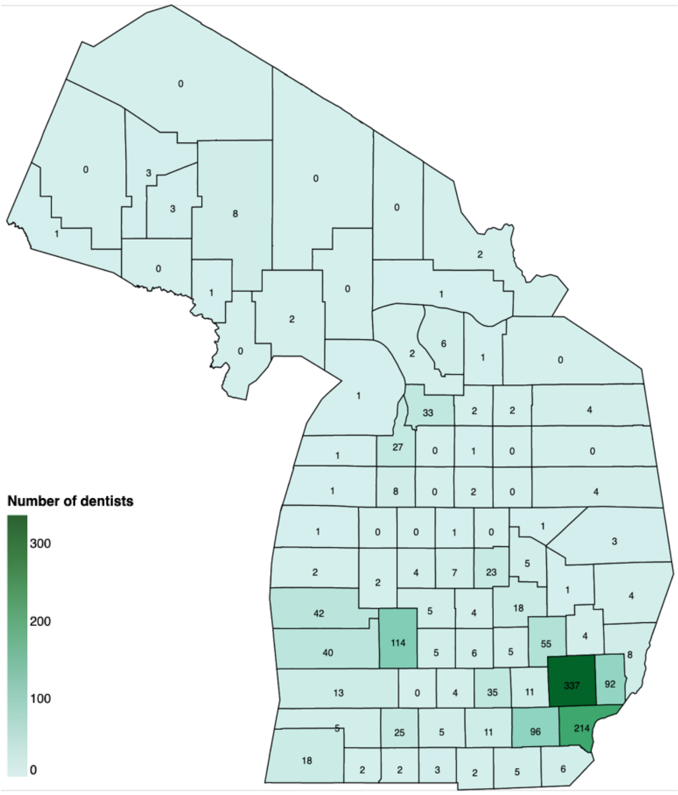

Number of dentists participating in CHIP

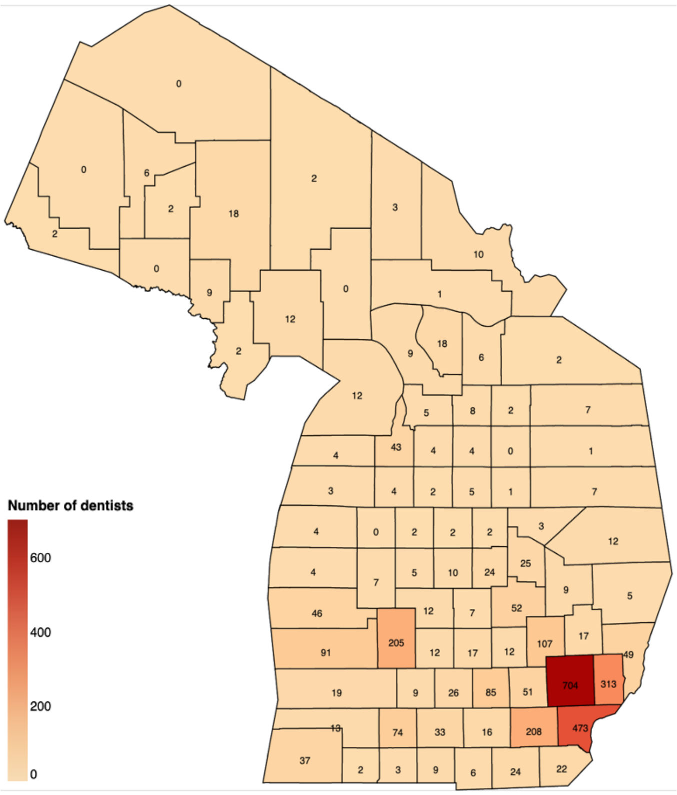

©2022 Serban N et al. *JAMA Network Open*.

Dental Care Supply
MINNESOTA

This state report summarizes data on the dental care supply, differentiated by type of insurance program, provider taxonomy, and rurality-urbanicity of practice address.

Percentage of dentists by provider taxonomy

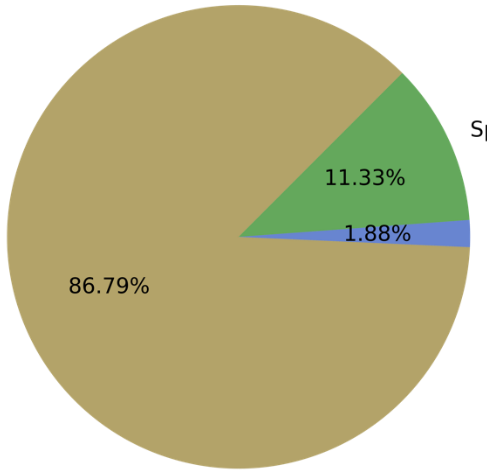

Number of dentists by rurality-urbanicity & provider taxonomy

Rurality-Urbanicity	General	Pediatric	Specialist
Urban	2081	53	307
Suburban	300	5	30
Rural	300	1	13

Percentage of dentists by participation in public insurance programs

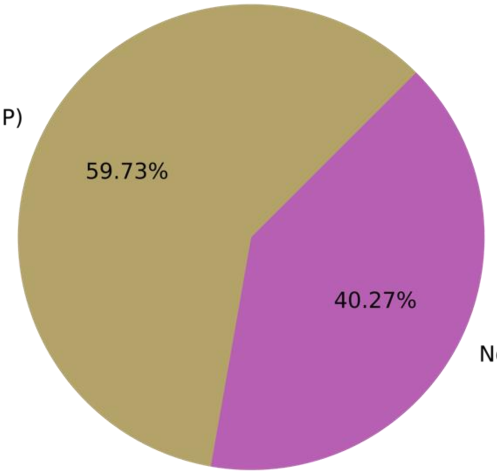

Number of dentists by rurality-urbanicity & participation in public insurance programs

Rurality-Urbanicity	Medicaid(+CHIP)	No Medicaid(+CHIP)
Urban	1394	1047
Suburban	240	95
Rural	212	102

Number of dentists by provider taxonomy & participation in public insurance programs

Provider Type	Medicaid(+CHIP)	No Medicaid(+CHIP)
General	1599	1082
Pediatric	47	11
Specialist	199	151

©2022 Serban N et al. *JAMA Network Open*.

Number of dentists

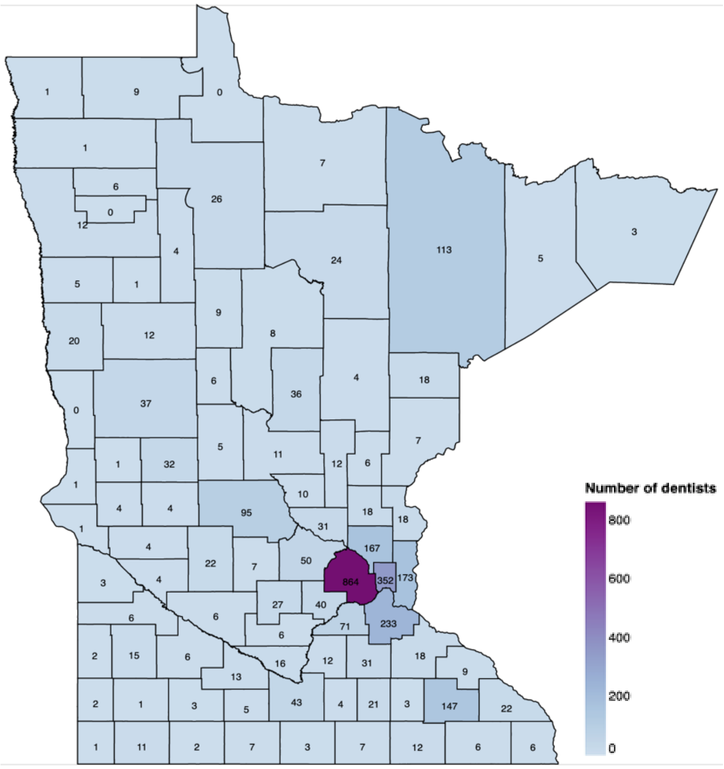

Number of dental hygienists

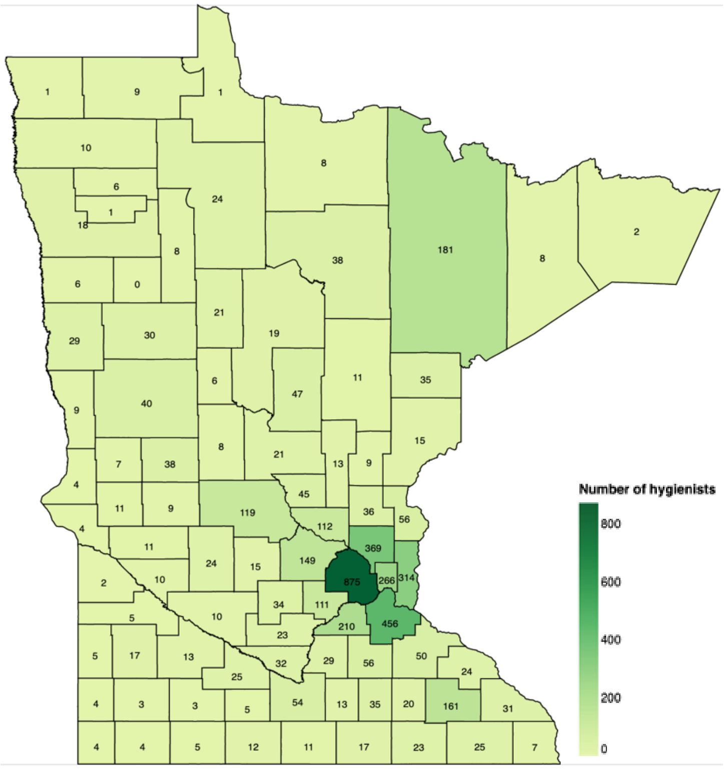

Number of dentists participating in Medicaid(+CHIP)

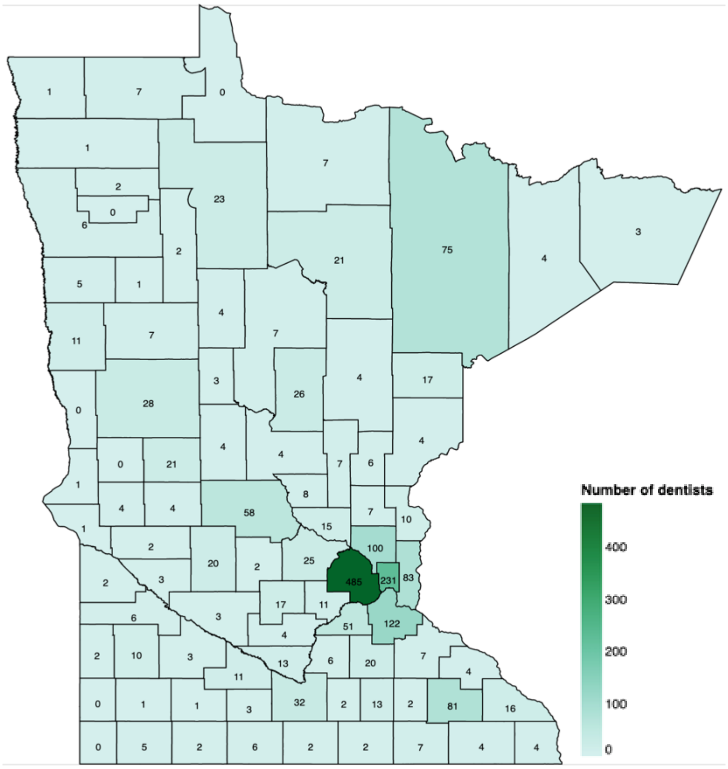

©2022 Serban N et al. *JAMA Network Open*.

Dental Care Supply
MISSISSIPPI

This state report summarizes data on the dental care supply, differentiated by type of insurance program, provider taxonomy, and rurality-urbanicity of practice address.

Percentage of dentists by provider taxonomy

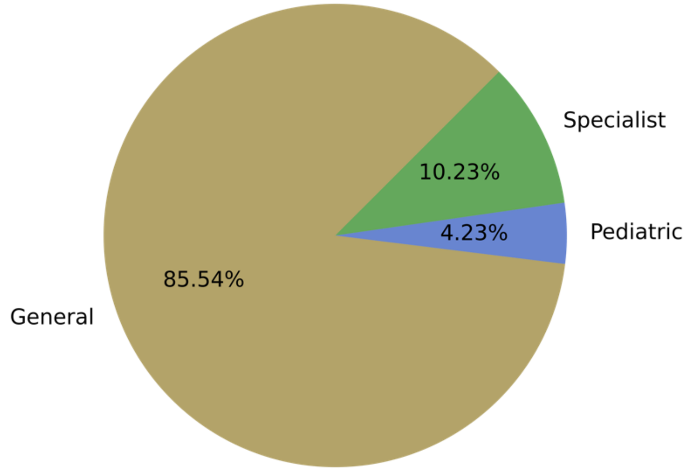

Number of dentists by rurality-urbanicity & provider taxonomy

Rurality-Urbanicity	General	Pediatric	Specialist
**Urban**	581	38	88
**Suburban**	341	16	36
**Rural**	190	1	9

Percentage of dentists by participation in public insurance programs

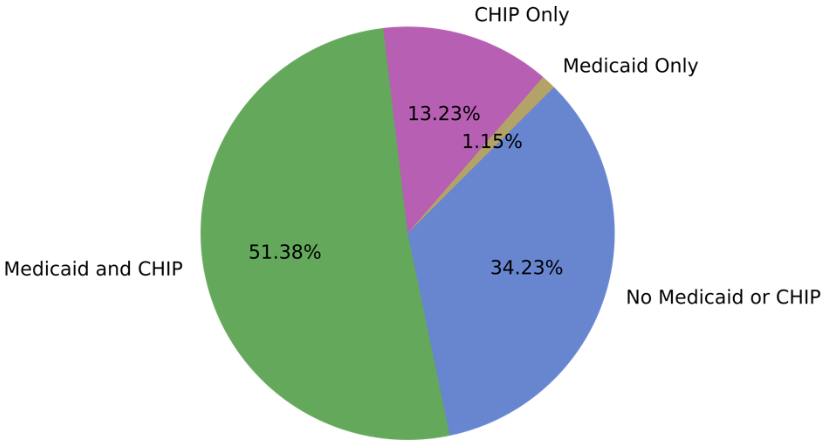

Number of dentists by rurality-urbanicity & participation in public insurance programs

Rurality-Urbanicity	Medicaid Only	CHIP Only	Medicaid and CHIP	No Medicaid or CHIP
**Urban**	5	102	312	289
**Suburban**	7	54	212	119
**Rural**	3	16	144	37

Number of dentists by provider taxonomy & participation in public insurance programs

Provider Type	Medicaid Only	CHIP Only	Medicaid and CHIP	No Medicaid or CHIP
**General**	13	162	552	385
**Pediatric**	0	2	47	6
**Specialist**	2	8	69	54

©2022 Serban N et al. *JAMA Network Open*.

Number of dentists

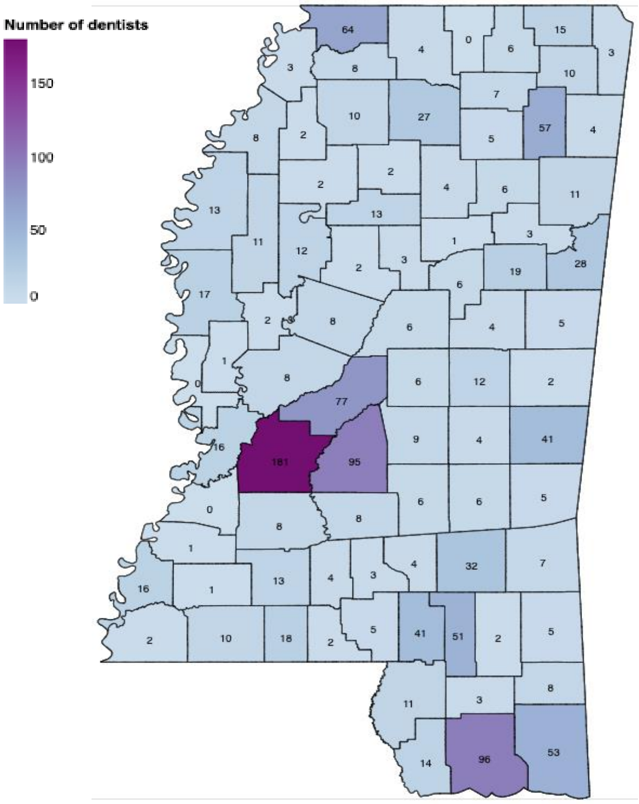

Number of dental hygienists

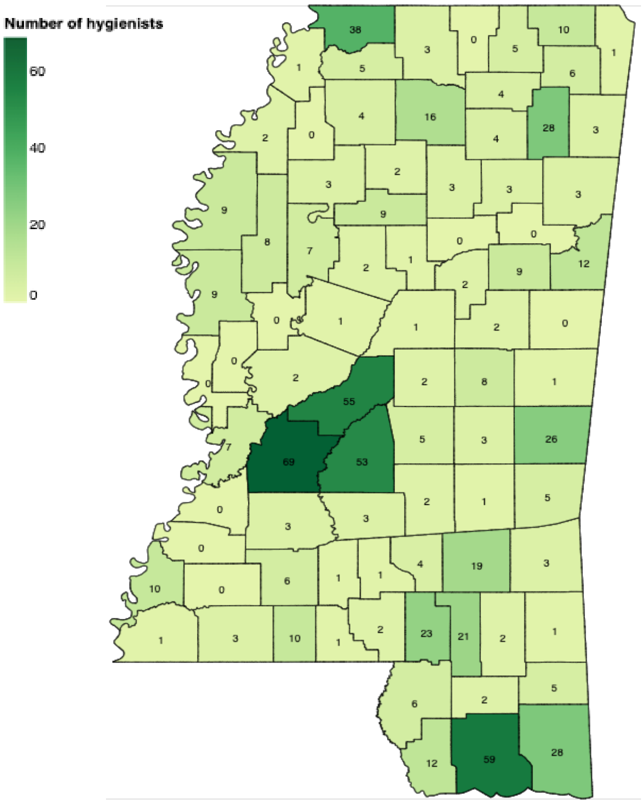

Number of dentists participating in Medicaid

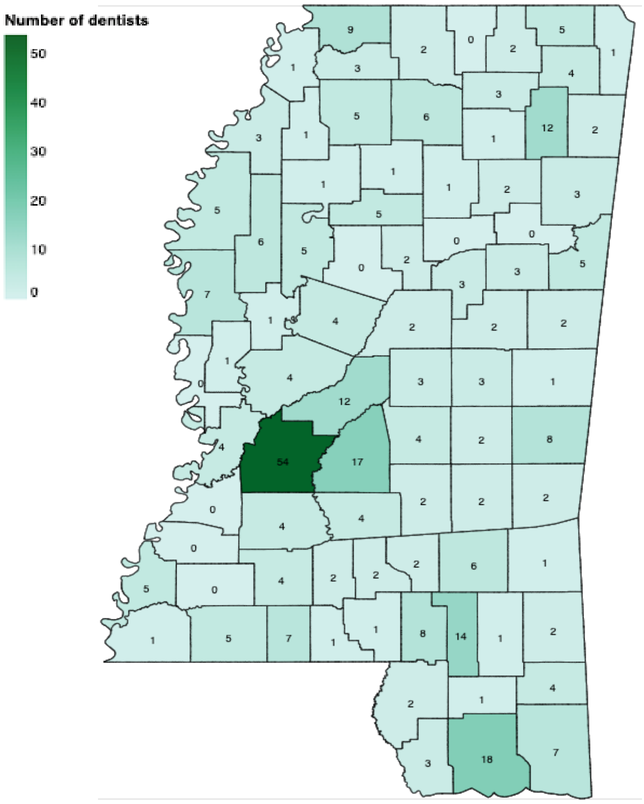

Number of dentists participating in CHIP

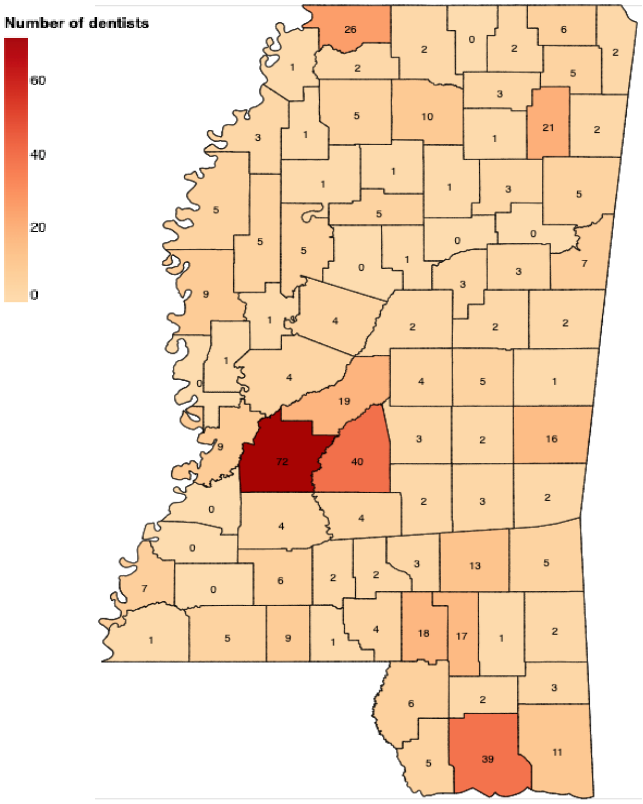

©2022 Serban N et al. *JAMA Network Open*.

Dental Care Supply
MISSOURI

This state report summarizes data on the dental care supply, differentiated by type of insurance program, provider taxonomy, and rurality-urbanicity of practice address.

Percentage of dentists by provider taxonomy

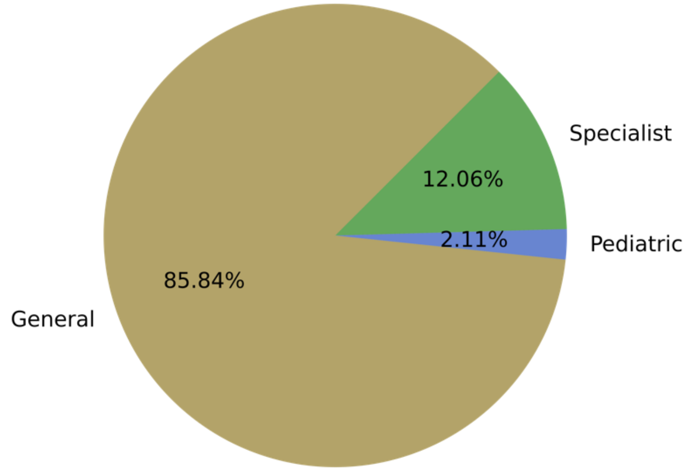

Number of dentists by rurality-urbanicity & provider taxonomy

Rurality-Urbanicity	General	Pediatric	Specialist
Urban	2226	60	341
Suburban	258	6	34
Rural	243	1	8

Percentage of dentists by participation in public insurance programs

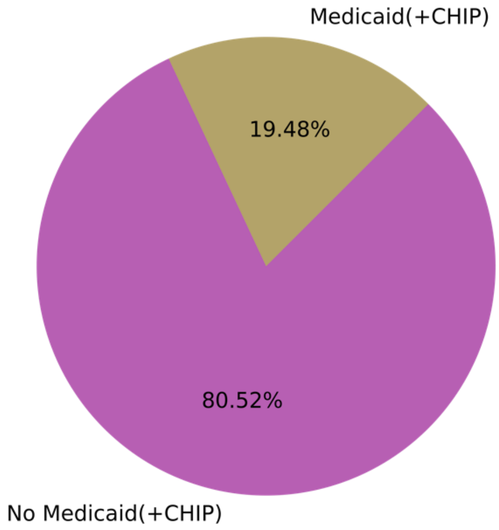

Number of dentists by rurality-urbanicity & participation in public insurance programs

Rurality-Urbanicity	Medicaid(+CHIP)	No Medicaid(+CHIP)
Urban	437	2190
Suburban	81	217
Rural	102	150

Number of dentists by provider taxonomy & participation in public insurance programs

Provider Type	Medicaid(+CHIP)	No Medicaid(+CHIP)
General	519	2208
Pediatric	27	40
Specialist	73	310

©2022 Serban N et al. *JAMA Network Open*.

Number of dentists

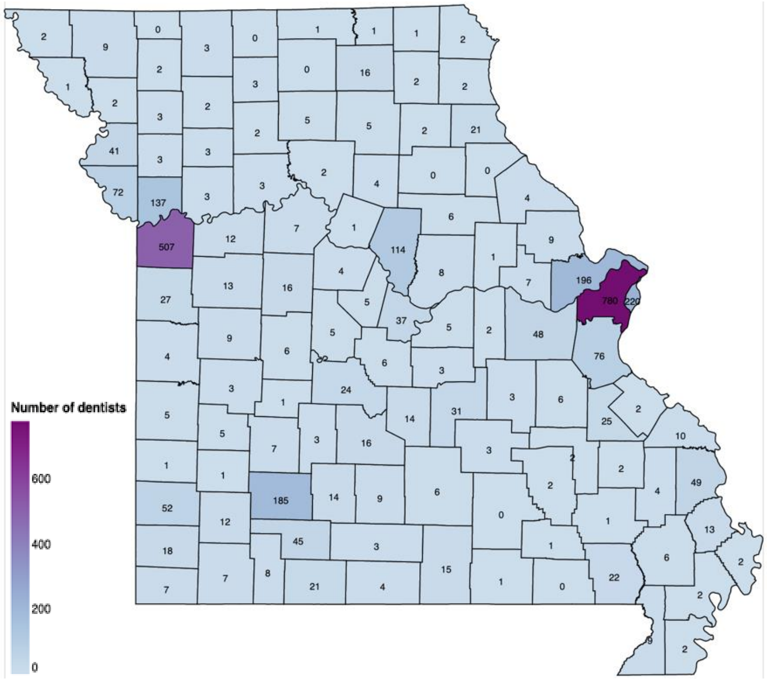

Number of dental hygienists

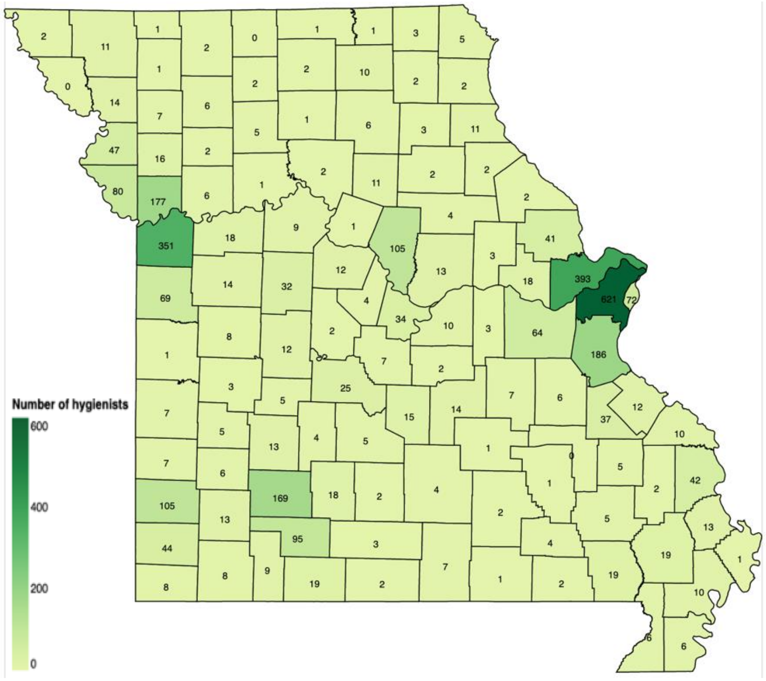

Number of dentists participating in Medicaid(+CHIP)

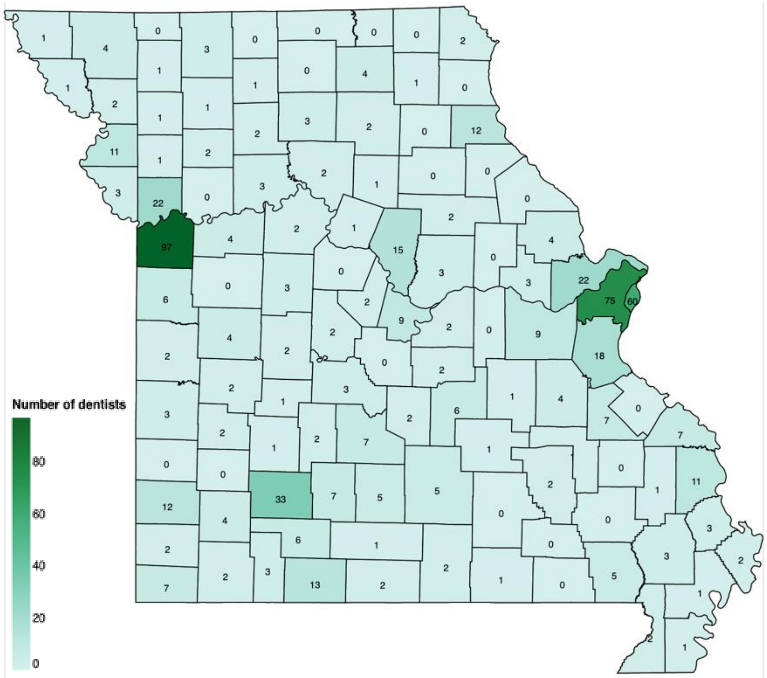

©2022 Serban N et al. *JAMA Network Open*.

Dental Care Supply
MONTANA

This state report summarizes data on the dental care supply, differentiated by type of insurance program, provider taxonomy, and rurality-urbanicity of practice address.

Percentage of dentists by provider taxonomy

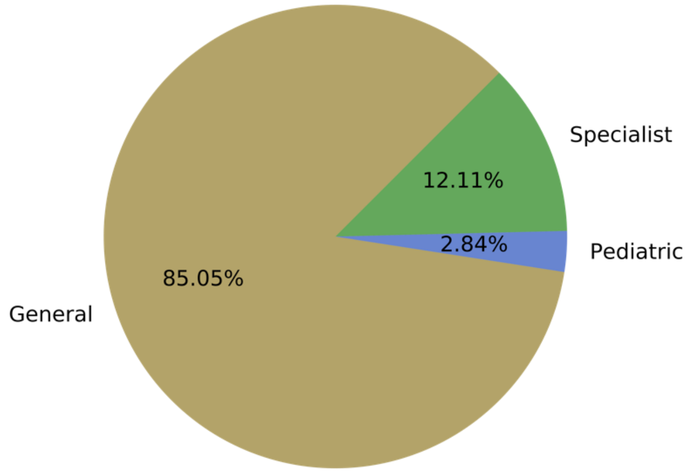

Number of dentists by rurality-urbanicity & provider taxonomy

Rurality-Urbanicity	General	Pediatric	Specialist
Urban	216	13	41
Suburban	168	5	36
Rural	185	1	4

Percentage of dentists by participation in public insurance programs

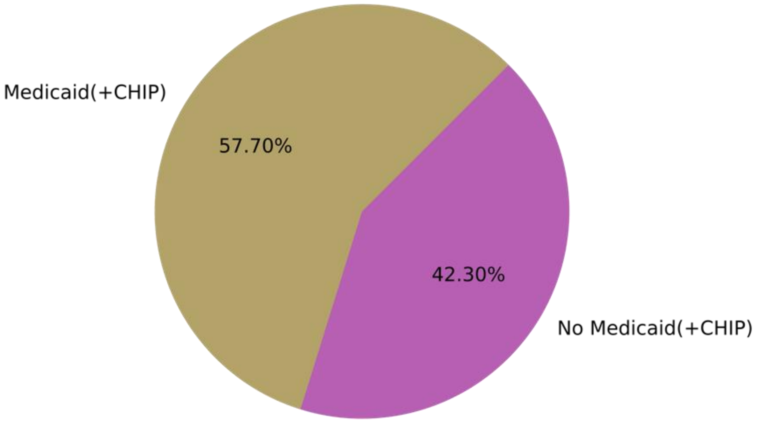

Number of dentists by rurality-urbanicity & participation in public insurance programs

Rurality-Urbanicity	Medicaid(+CHIP)	No Medicaid(+CHIP)
Urban	154	116
Suburban	117	92
Rural	115	75

Number of dentists by provider taxonomy & participation in public insurance programs

Provider Type	Medicaid(+CHIP)	No Medicaid(+CHIP)
General	341	228
Pediatric	13	6
Specialist	32	49

©2022 Serban N et al. *JAMA Network Open*.

Number of dentists

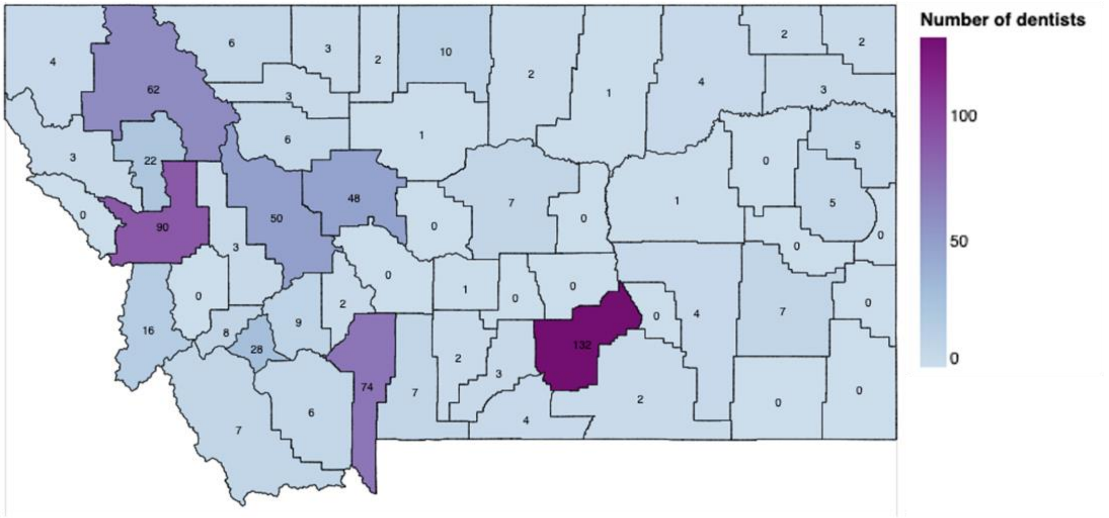

Number of dental hygienists

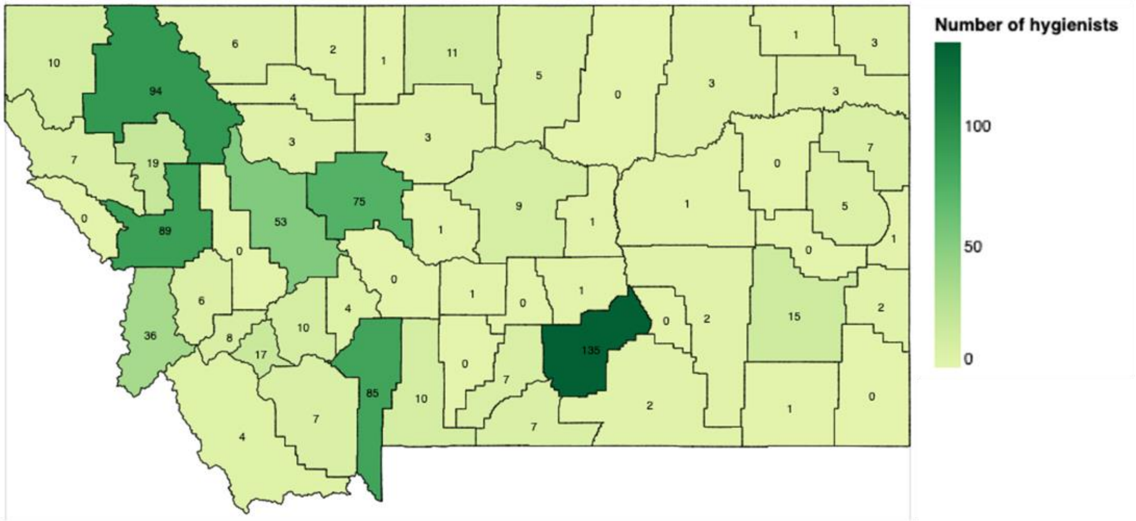

Number of dentists participating in Medicaid(+CHIP)

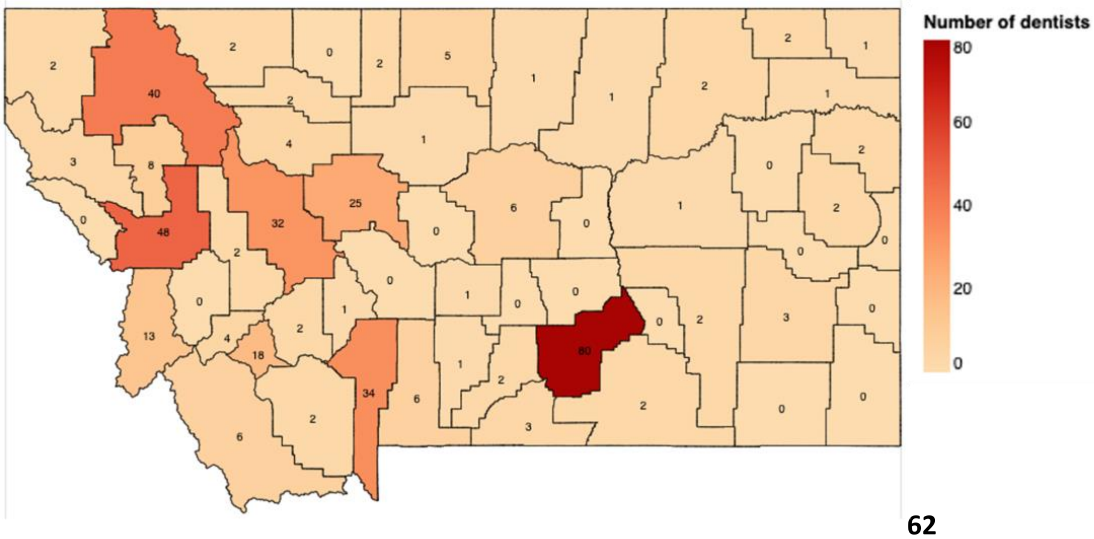

©2022 Serban N et al. *JAMA Network Open*.

Dental Care Supply
NEBRASKA

This state report summarizes data on the dental care supply, differentiated by type of insurance program, provider taxonomy, and rurality-urbanicity of practice address.

Percentage of dentists by provider taxonomy

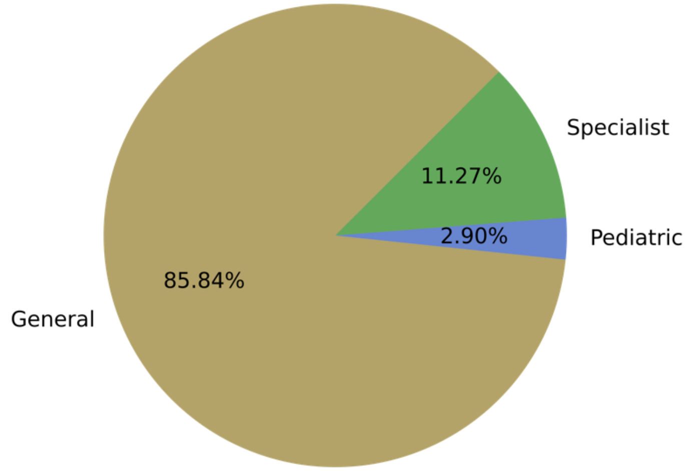

Number of dentists by rurality-urbanicity & provider taxonomy

Rurality-Urbanicity	General	Pediatric	Specialist
Urban	778	32	118
Suburban	162	5	18
Rural	157	0	7

Percentage of dentists by participation in public insurance programs

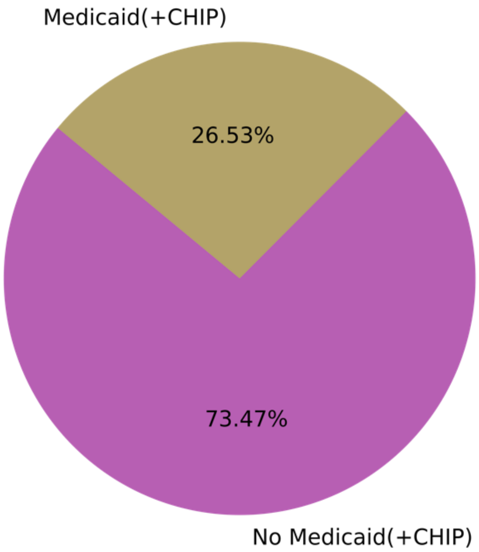

Number of dentists by rurality-urbanicity & participation in public insurance programs

Rurality-Urbanicity	Medicaid(+CHIP)	No Medicaid(+CHIP)
Urban	198	730
Suburban	68	118
Rural	73	91

Number of dentists by provider taxonomy & participation in public insurance programs

Provider Type	Medicaid(+CHIP)	No Medicaid(+CHIP)
General	304	793
Pediatric	20	17
Specialist	15	129

©2022 Serban N et al. *JAMA Network Open*.

Number of dentists

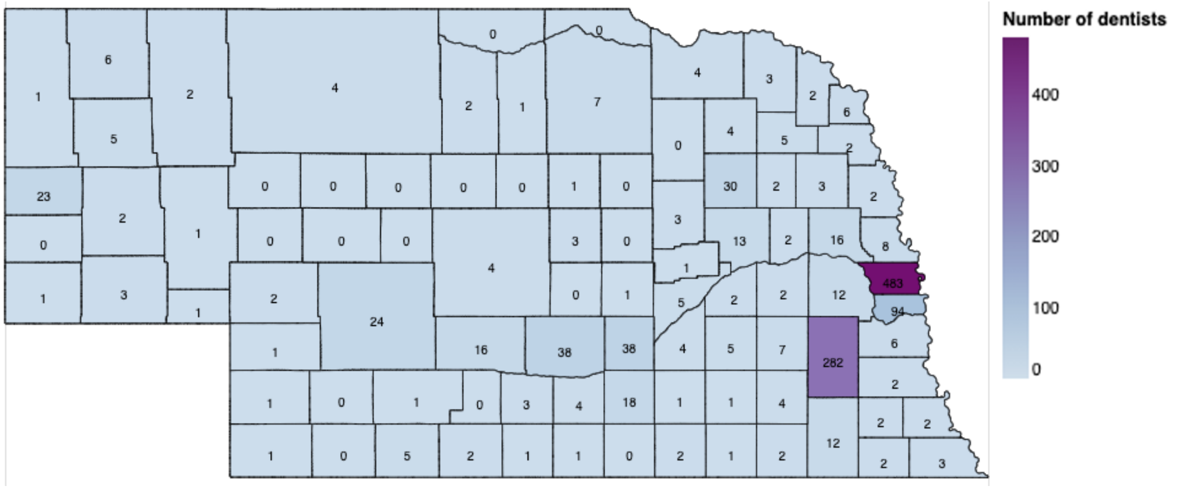

Number of dental hygienists

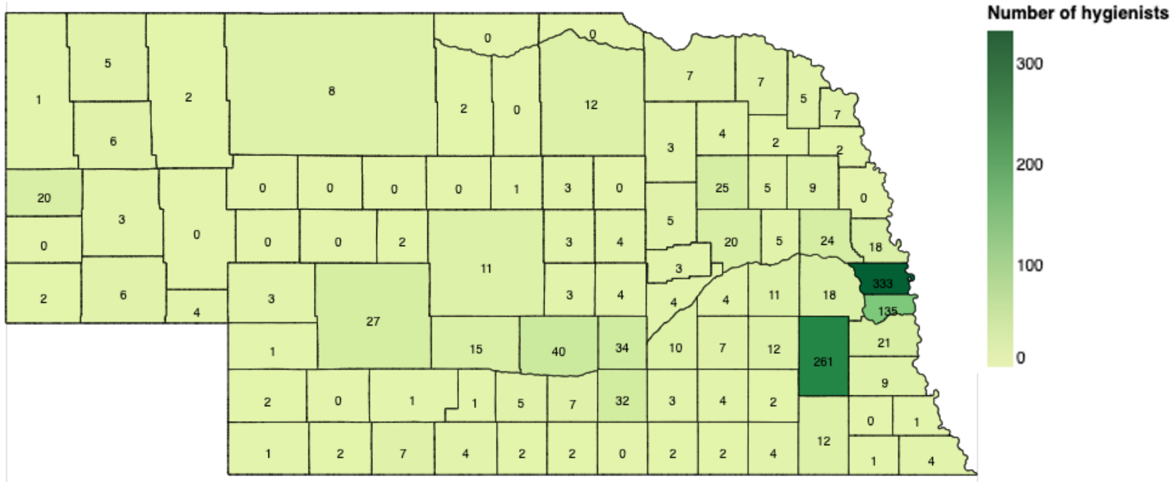

Number of dentists participating in Medicaid(+CHIP)

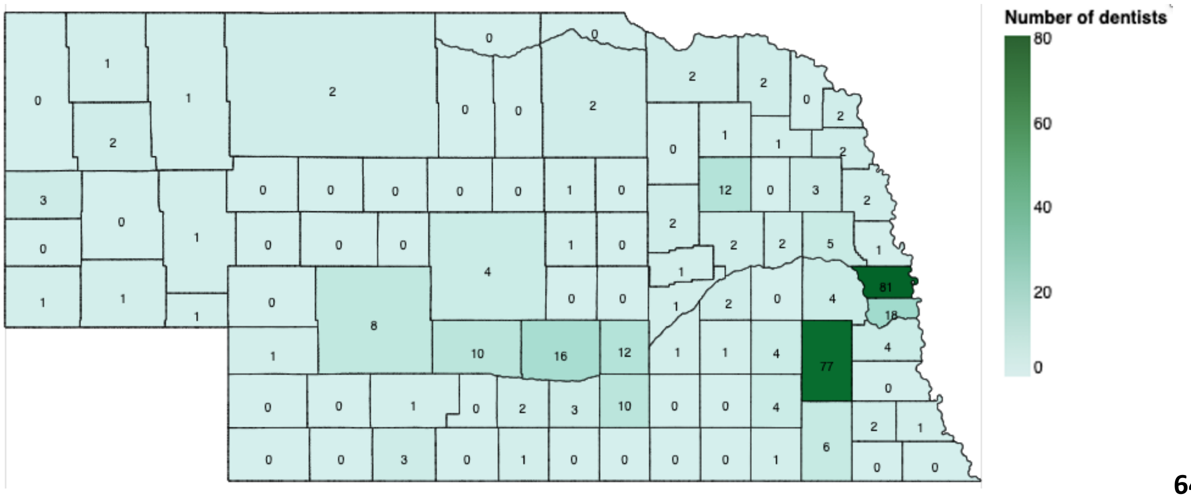

©2022 Serban N et al. *JAMA Network Open*.

Dental Care Supply
NEVADA

This state report summarizes data on the dental care supply, differentiated by type of insurance program, provider taxonomy, and rurality-urbanicity of practice address.

Percentage of dentists by provider taxonomy

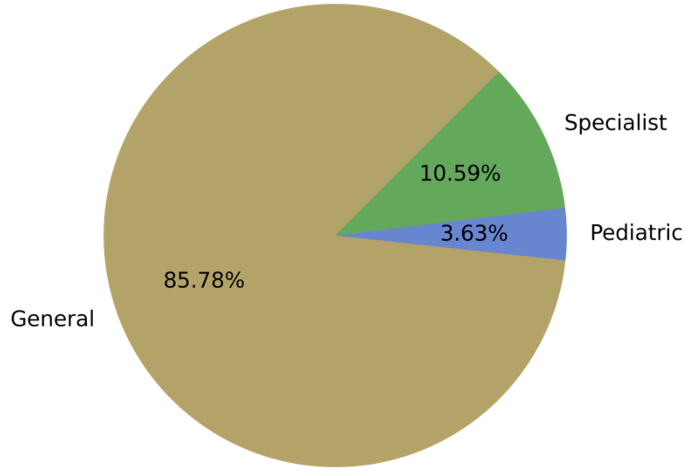

Number of dentists by rurality-urbanicity & provider taxonomy

Rurality-Urbanicity	General	Pediatric	Specialist
Urban	1360	56	171
Suburban	66	6	5
Rural	39	0	4

Percentage of dentists by participation in public insurance programs

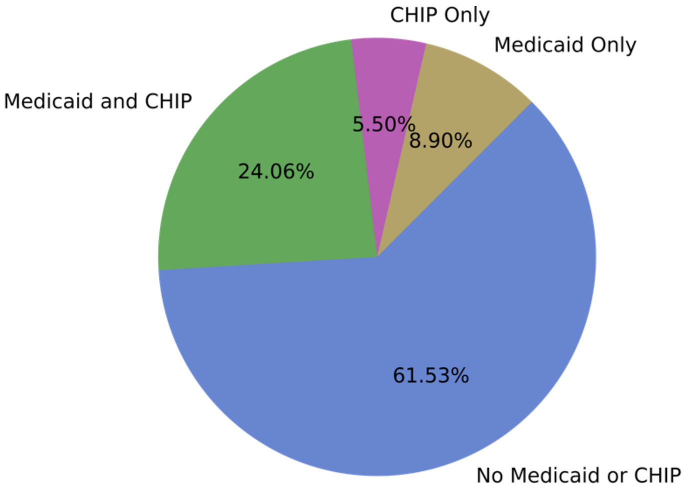

Number of dentists by rurality-urbanicity & participation in public insurance programs

Rurality-Urbanicity	Medicaid Only	CHIP Only	Medicaid and CHIP	No Medicaid or CHIP
Urban	145	88	385	969
Suburban	5	5	17	50
Rural	2	1	8	32

Number of dentists by provider taxonomy & participation in public insurance programs

Provider Type	Medicaid Only	CHIP Only	Medicaid and CHIP	No Medicaid or CHIP
General	120	67	336	943
Pediatric	14	9	30	9
Specialist	18	18	45	99

©2022 Serban N et al. *JAMA Network Open*.

Number of dentists

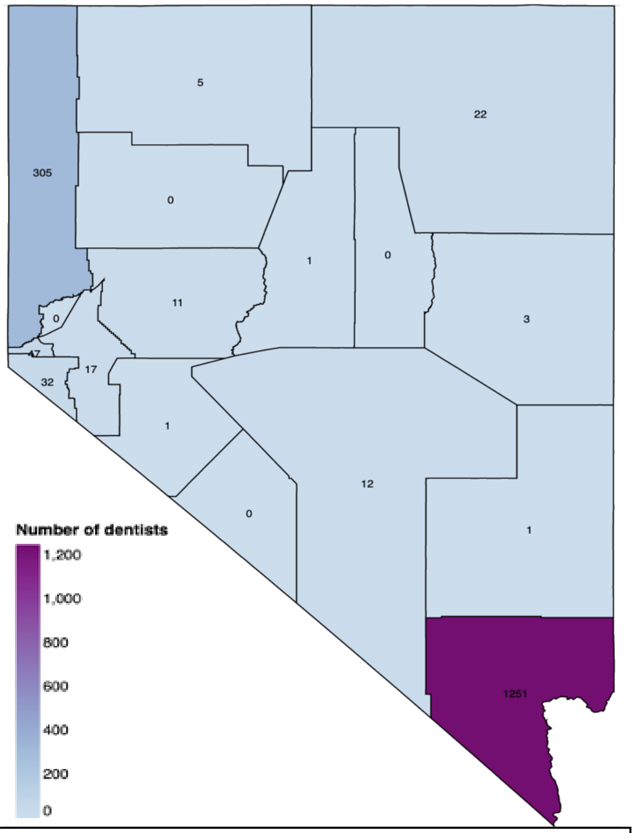

Number of dental hygienists

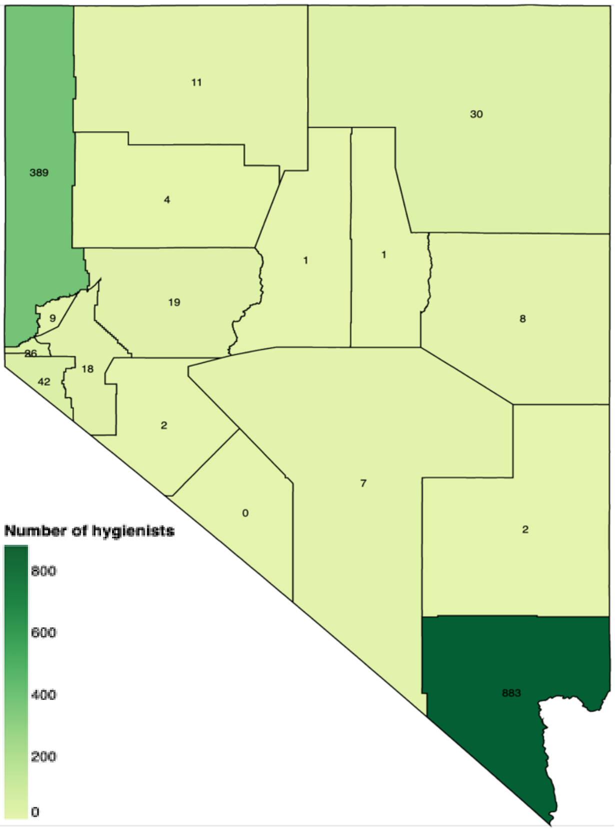

Number of dentists participating in Medicaid

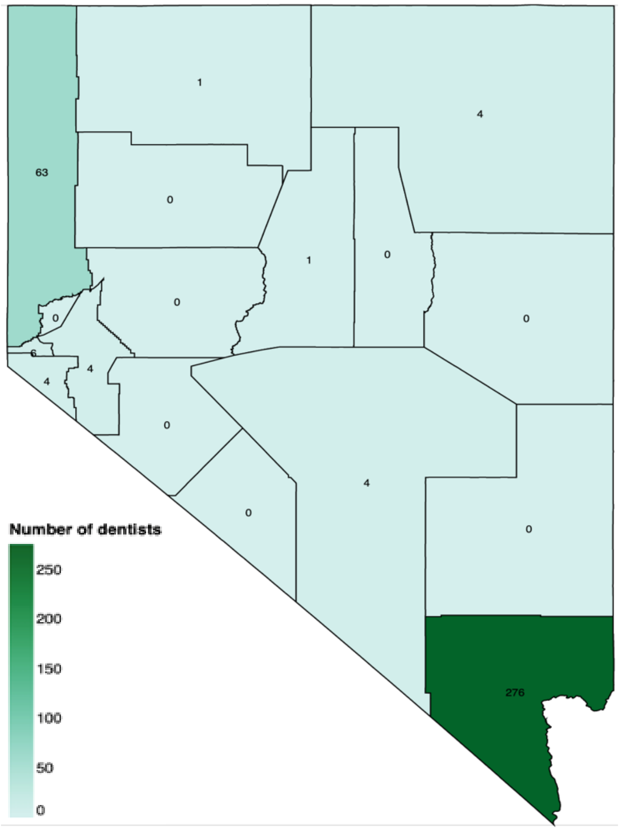

Number of dentists participating in CHIP

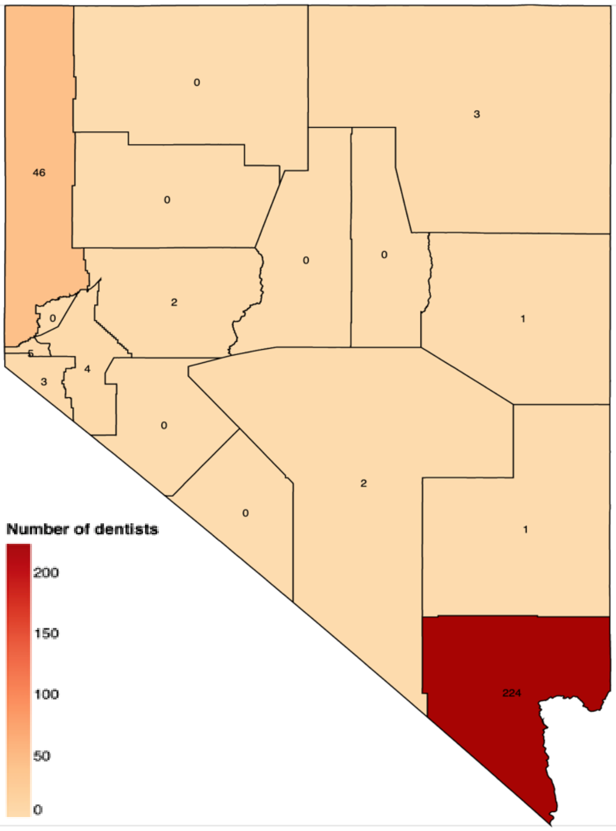

©2022 Serban N et al. *JAMA Network Open*.

Dental Care Supply
NEW HAMPSHIRE

This state report summarizes data on the dental care supply, differentiated by type of insurance program, provider taxonomy, and rurality-urbanicity of practice address.

Percentage of dentists by provider taxonomy

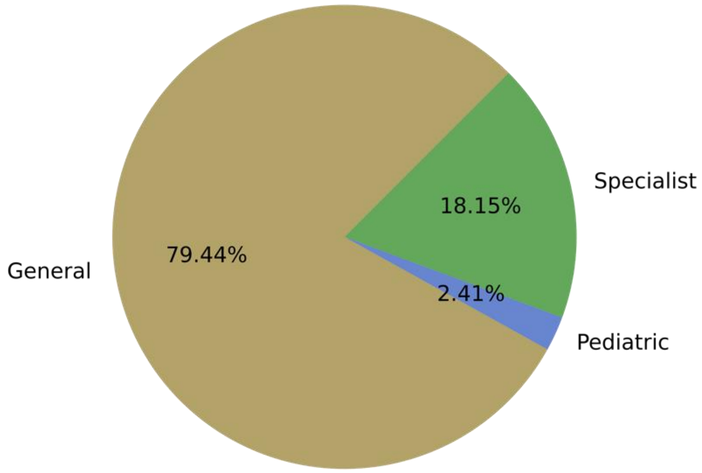

Number of dentists by rurality-urbanicity & provider taxonomy

Rurality-Urbanicity	General	Pediatric	Specialist
**Urban**	562	16	138
**Suburban**	197	8	49
**Rural**	99	2	9

Percentage of dentists by participation in public insurance programs

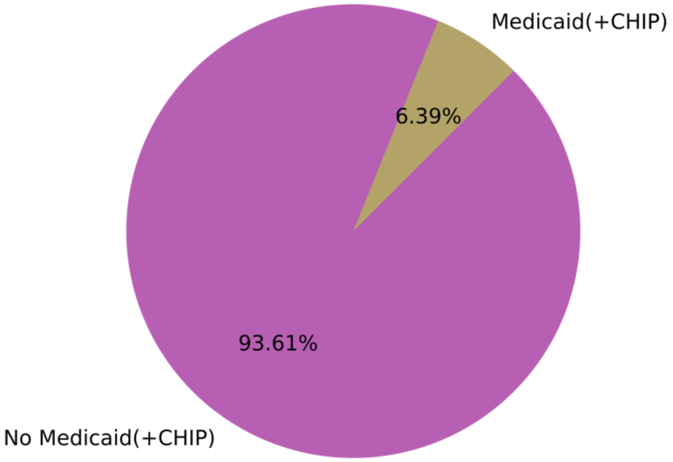

Number of dentists by rurality-urbanicity & participation in public insurance programs

Rurality-Urbanicity	Medicaid(+CHIP)	No Medicaid(+CHIP)
**Urban**	44	671
**Suburban**	13	241
**Rural**	12	98

Number of dentists by provider taxonomy & participation in public insurance programs

Provider Type	Medicaid(+CHIP)	No Medicaid(+CHIP)
**General**	50	807
**Pediatric**	5	21
**Specialist**	14	182

©2022 Serban N et al. *JAMA Network Open*.

Number of dentists

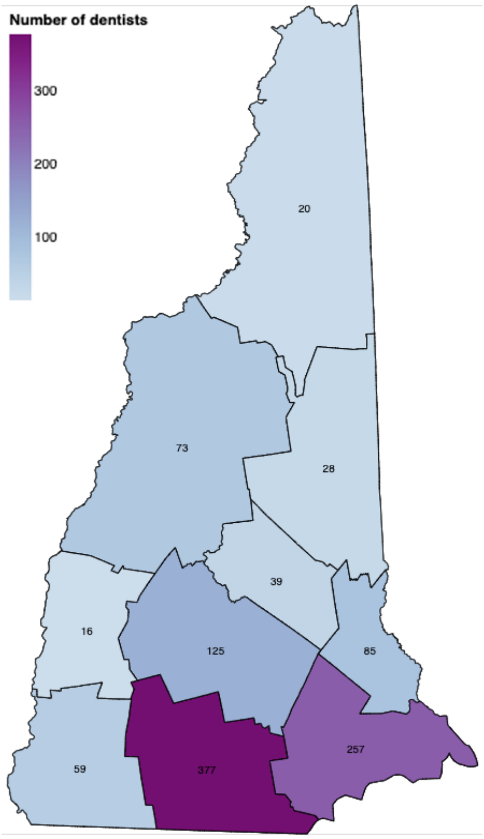

Number of dental hygienists

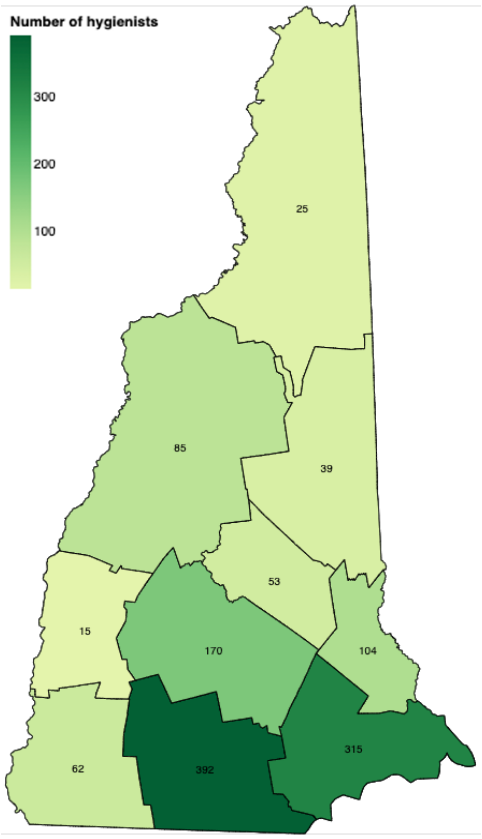

Number of dentists participating in Medicaid(+CHIP)

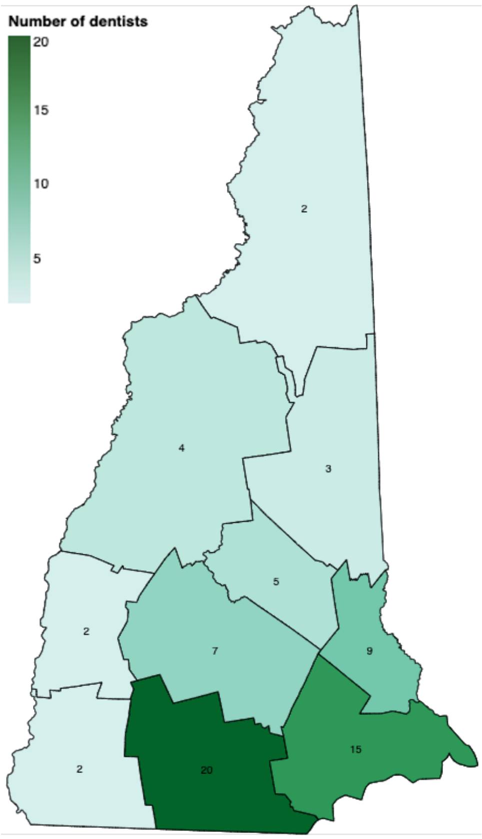

©2022 Serban N et al. *JAMA Network Open*.

Dental Care Supply
NEW JERSEY

This state report summarizes data on the dental care supply, differentiated by type of insurance program, provider taxonomy, and rurality-urbanicity of practice address.

Percentage of dentists by provider taxonomy

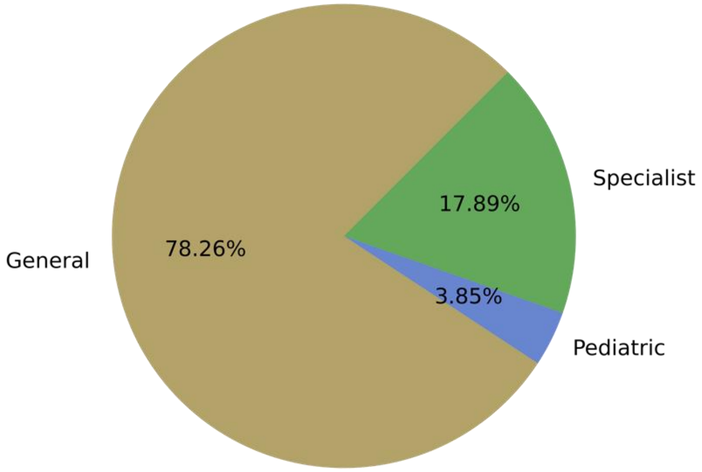

Number of dentists by rurality-urbanicity & provider taxonomy

Rurality-Urbanicity	General	Pediatric	Specialist
Urban	6194	308	1423
Suburban	88	1	12
Rural	0	0	0

Percentage of dentists by participation in public insurance programs

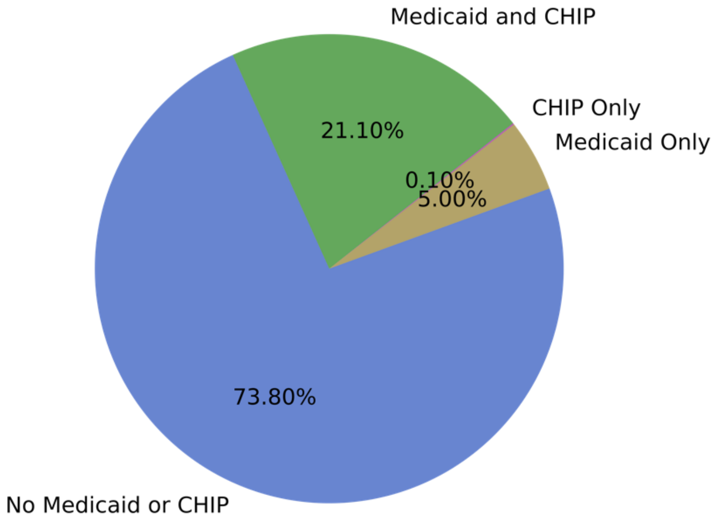

Number of dentists by rurality-urbanicity & participation in public insurance programs

Rurality-Urbanicity	Medicaid Only	CHIP Only	Medicaid and CHIP	No Medicaid or CHIP
Urban	396	9	1674	5848
Suburban	5	0	20	75
Rural	0	0	0	0

Number of dentists by provider taxonomy & participation in public insurance programs

Provider Type	Medicaid Only	CHIP Only	Medicaid and CHIP	No Medicaid or CHIP
General	297	7	1264	4714
Pediatric	30	1	124	154
Specialist	74	0	306	1056

©2022 Serban N et al. *JAMA Network Open*.

Number of dentists

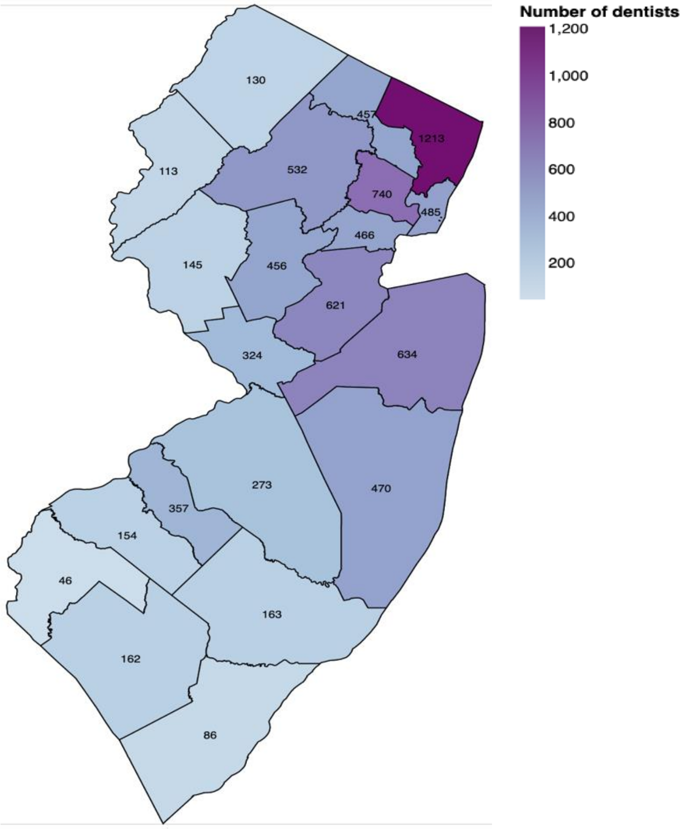

Number of dental hygienists

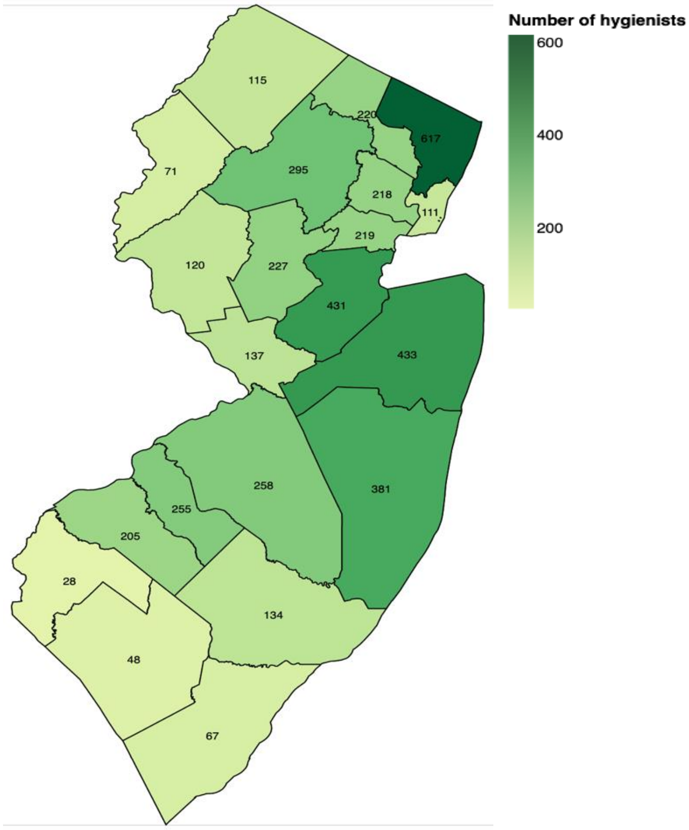

Number of dentists participating in Medicaid

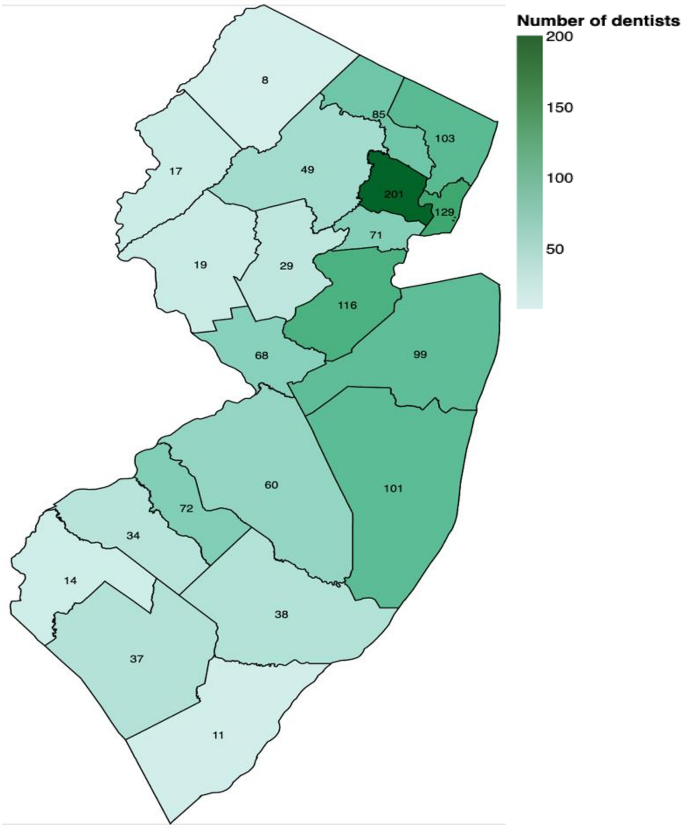

Number of dentists participating in CHIP

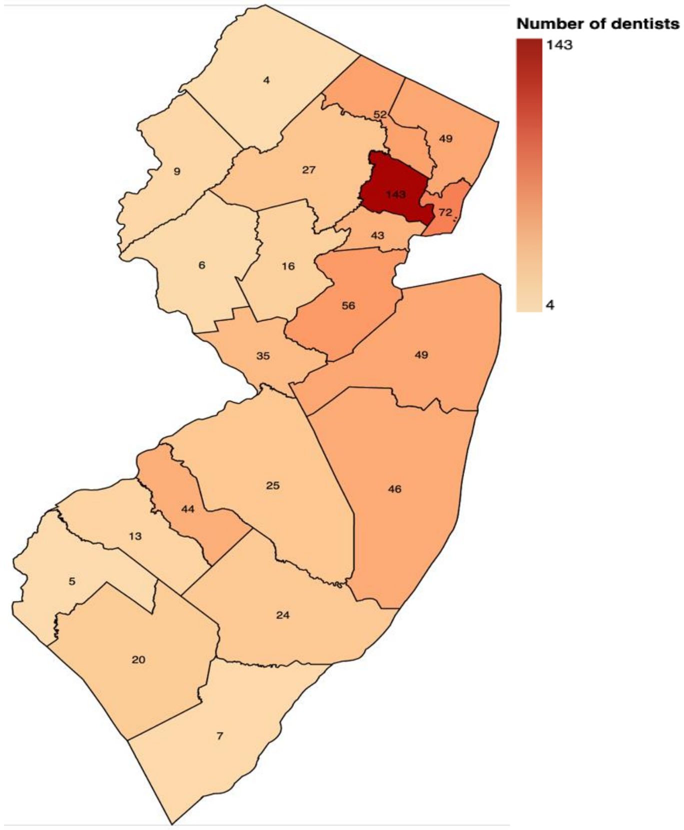

©2022 Serban N et al. *JAMA Network Open*.

Dental Care Supply
NEW MEXICO

This state report summarizes data on the dental care supply, differentiated by type of insurance program, provider taxonomy, and rurality-urbanicity of practice address.

Percentage of dentists by provider taxonomy

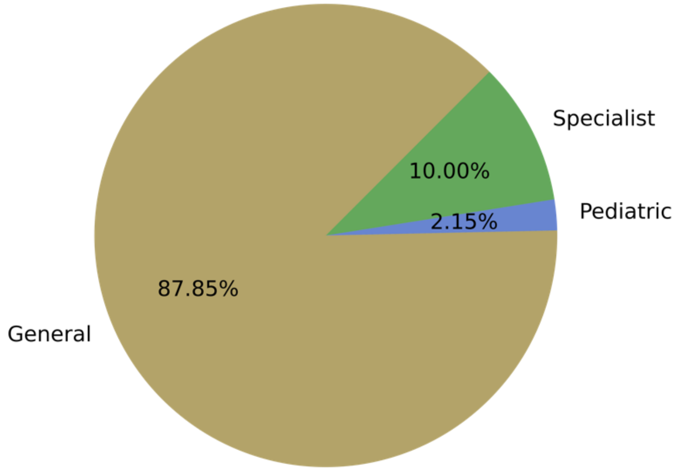

Number of dentists by rurality-urbanicity & provider taxonomy

Rurality-Urbanicity	General	Pediatric	Specialist
Urban	714	17	94
Suburban	146	5	10
Rural	80	1	2

Percentage of dentists by participation in public insurance programs

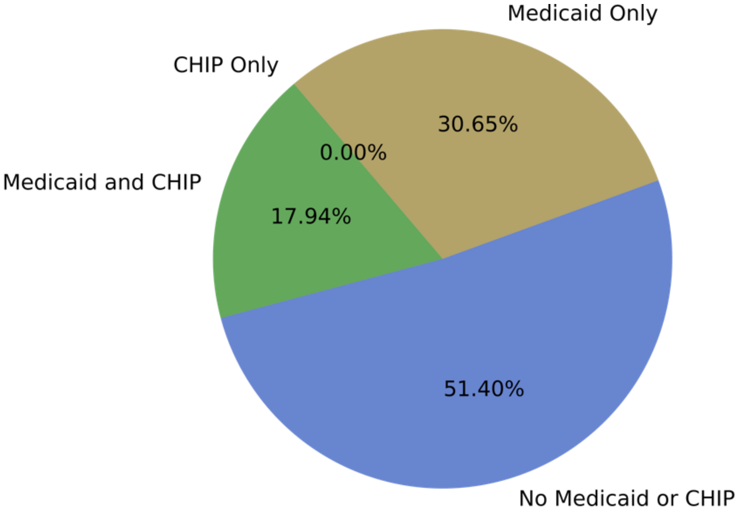

Number of dentists by rurality-urbanicity & participation in public insurance programs

Rurality-Urbanicity	Medicaid Only	CHIP Only	Medicaid and CHIP	No Medicaid or CHIP
Urban	247	0	136	443
Suburban	56	0	33	72
Rural	25	0	23	35

Number of dentists by provider taxonomy & participation in public insurance programs

Provider Type	Medicaid Only	CHIP Only	Medicaid and CHIP	No Medicaid or CHIP
General	284	0	165	491
Pediatric	13	0	8	2
Specialist	31	0	19	57

©2022 Serban N et al. *JAMA Network Open*.

Number of dentists

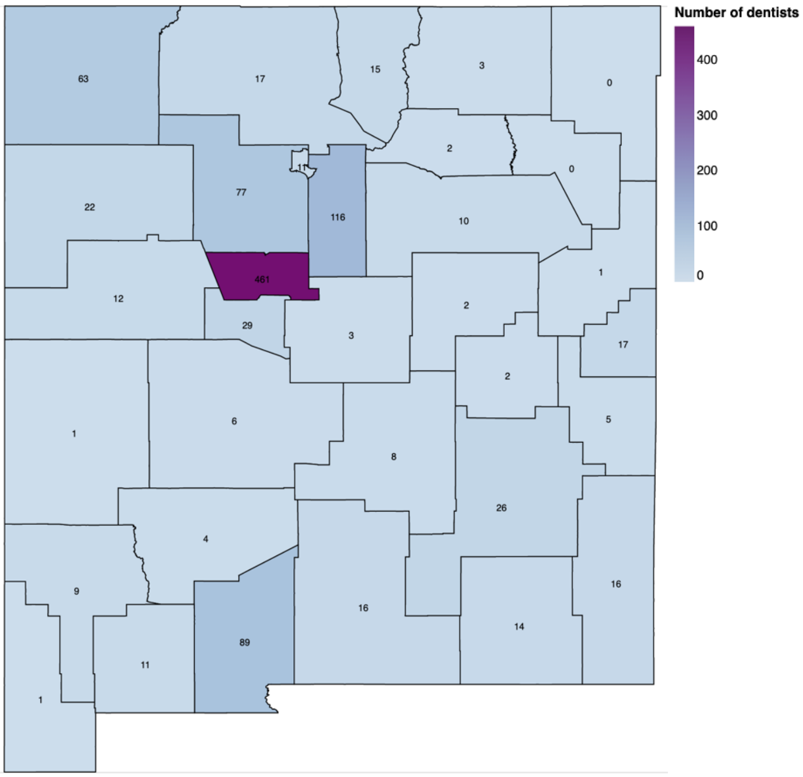

Number of dental hygienists

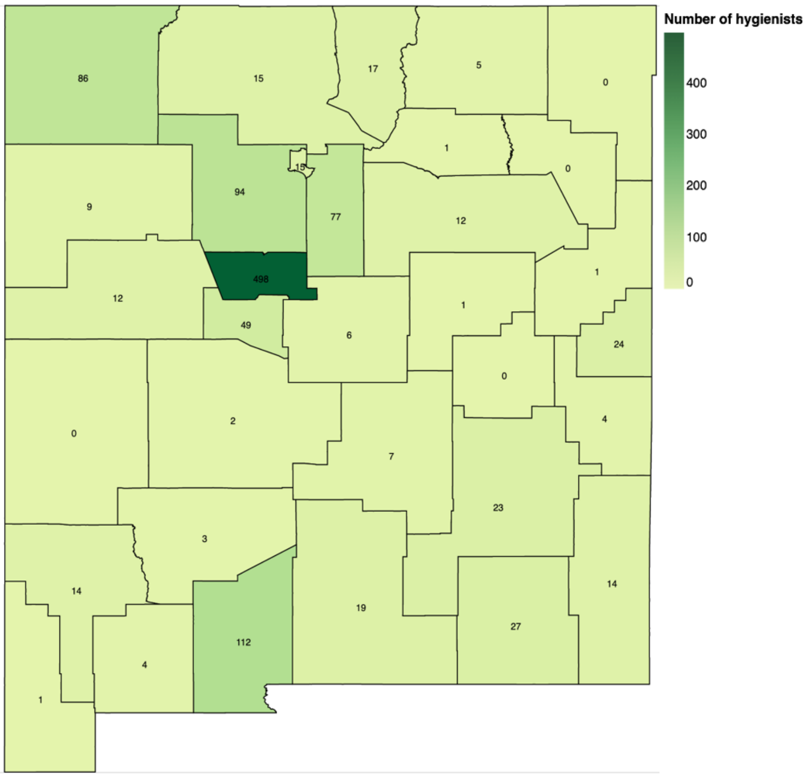

Number of dentists participating in Medicaid

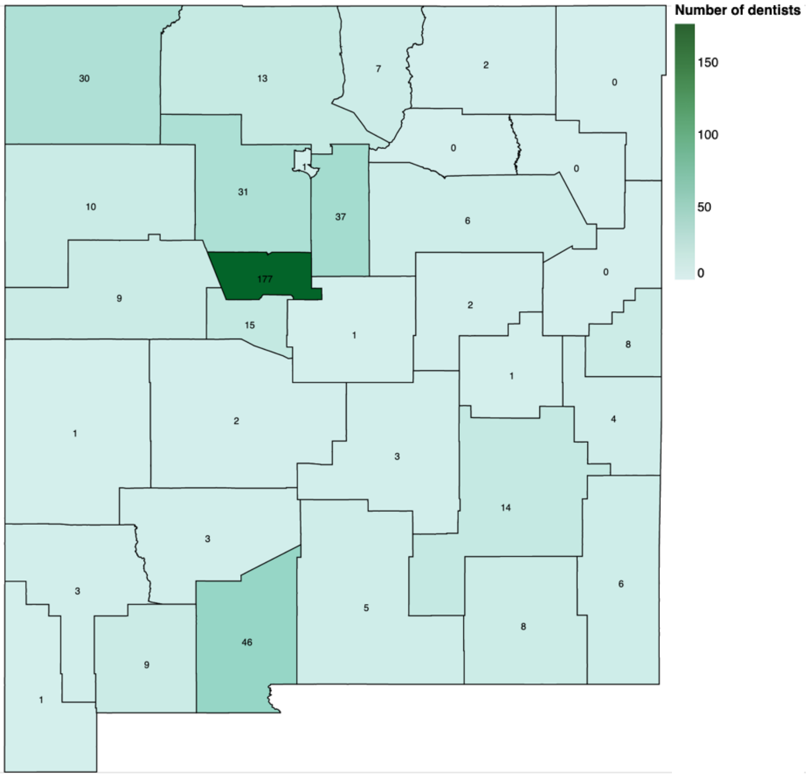

Number of dentists participating in CHIP

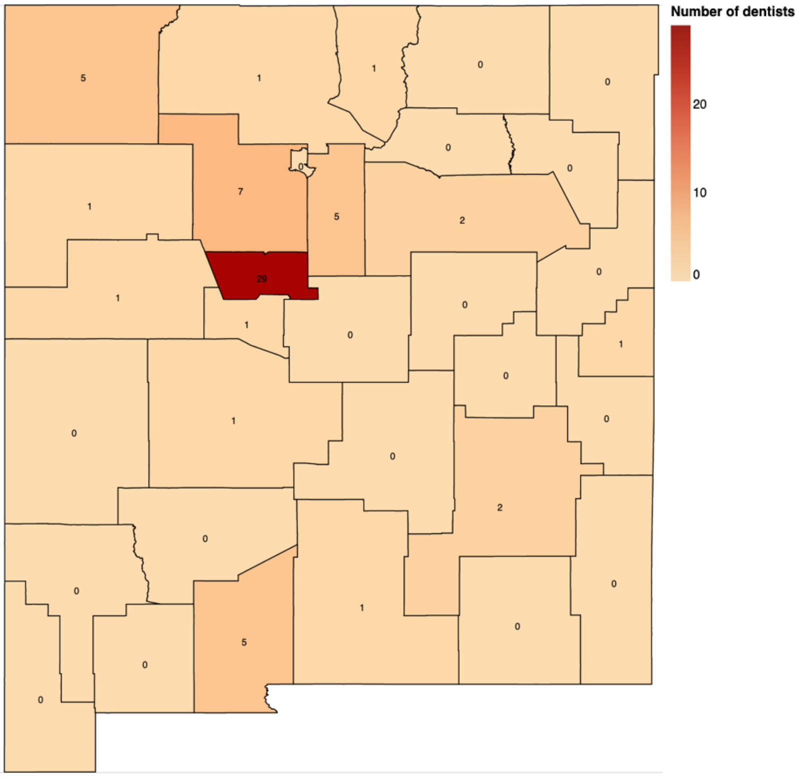

©2022 Serban N et al. *JAMA Network Open*.

Dental Care Supply
NEW YORK

This state report summarizes data on the dental care supply, differentiated by type of insurance program, provider taxonomy, and rurality-urbanicity of practice address.

Percentage of dentists by provider taxonomy

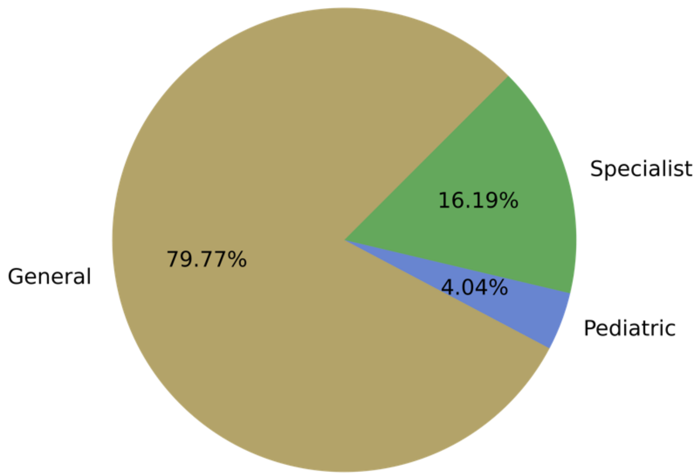

Number of dentists by rurality-urbanicity & provider taxonomy

Rurality-Urbanicity	General	Pediatric	Specialist
Urban	9918	518	2065
Suburban	341	11	56
Rural	298	7	22

Percentage of dentists by participation in public insurance programs

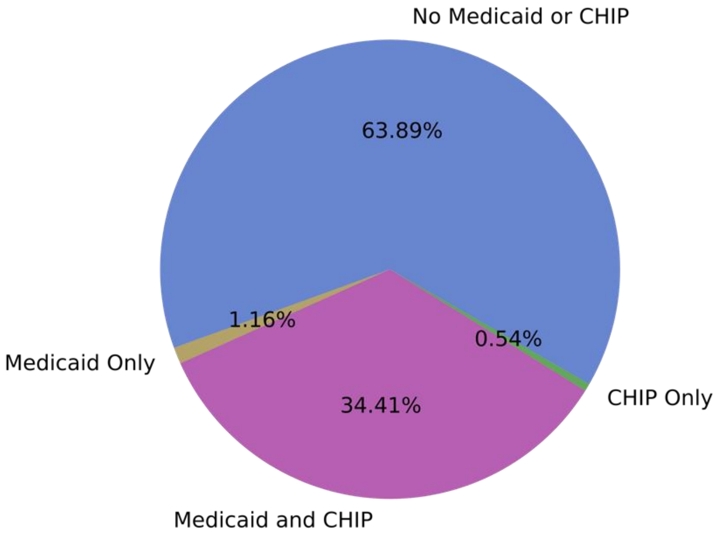

Number of dentists by rurality-urbanicity & participation in public insurance programs

Rurality-Urbanicity	Medicaid Only	CHIP Only	Medicaid and CHIP	No Medicaid or CHIP
Urban	136	64	4293	8008
Suburban	10	1	140	256
Rural	8	6	120	192

Number of dentists by provider taxonomy & participation in public insurance programs

Provider Type	Medicaid Only	CHIP Only	Medicaid and CHIP	No Medicaid or CHIP
General	117	66	3565	6809
Pediatric	7	5	351	172
Specialist	30	0	638	1475

©2022 Serban N et al. *JAMA Network Open*.

Number of dentists

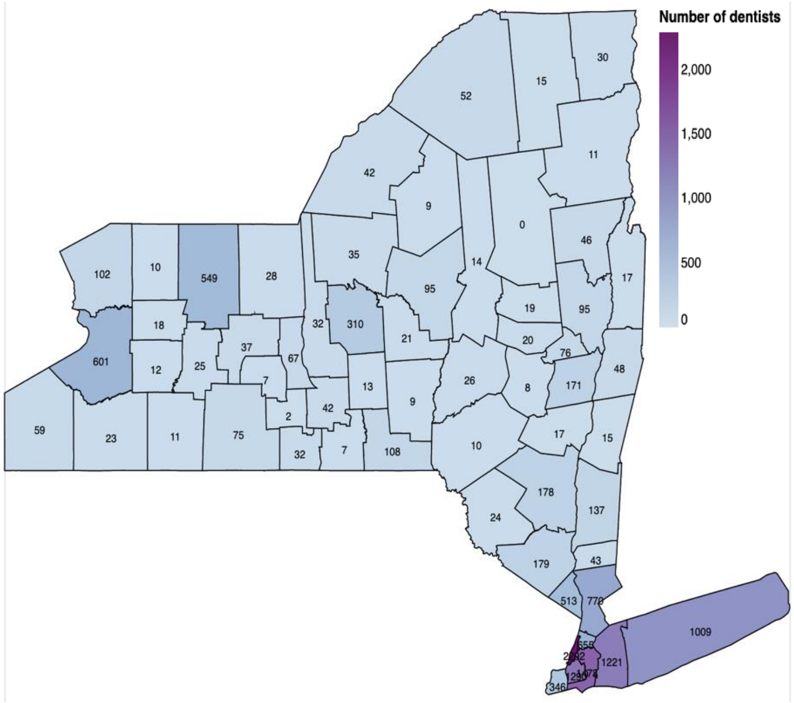

Number of dental hygienists

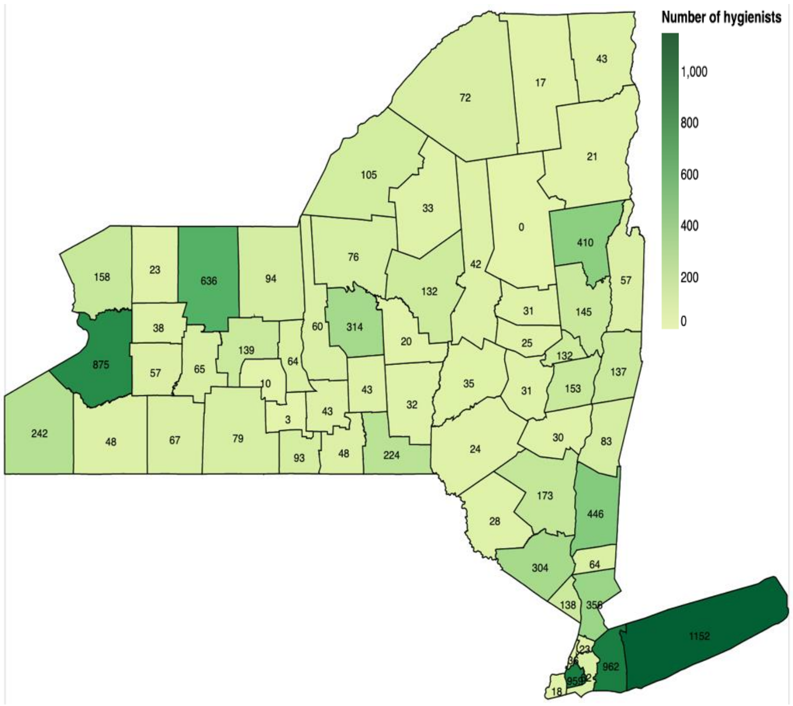

Number of dentists participating in Medicaid

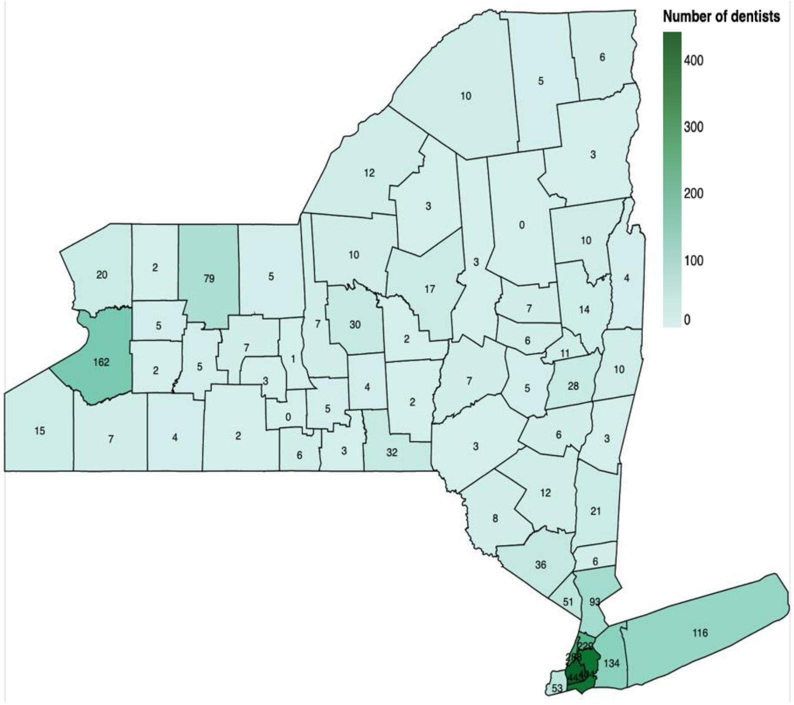

Number of dentists participating in CHIP

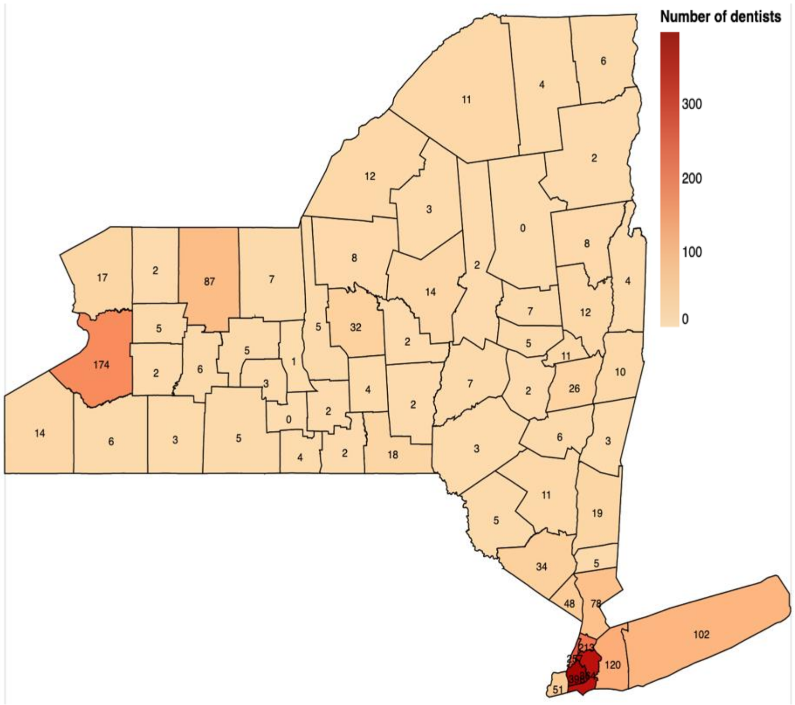

©2022 Serban N et al. *JAMA Network Open*.

Dental Care Supply
NORTH CAROLINA

This state report summarizes data on the dental care supply, differentiated by type of insurance program, provider taxonomy, and rurality-urbanicity of practice address.

Percentage of dentists by provider taxonomy

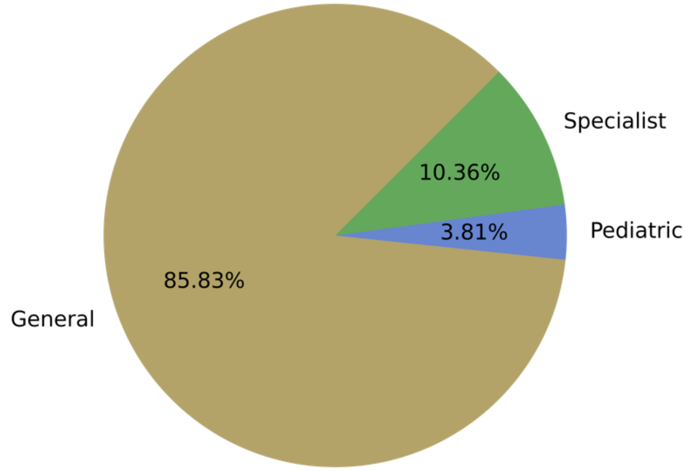

Number of dentists by rurality-urbanicity & provider taxonomy

Rurality-Urbanicity	General	Pediatric	Specialist
Urban	3930	161	484
Suburban	540	33	52
Rural	209	15	28

Percentage of dentists by participation in public insurance programs

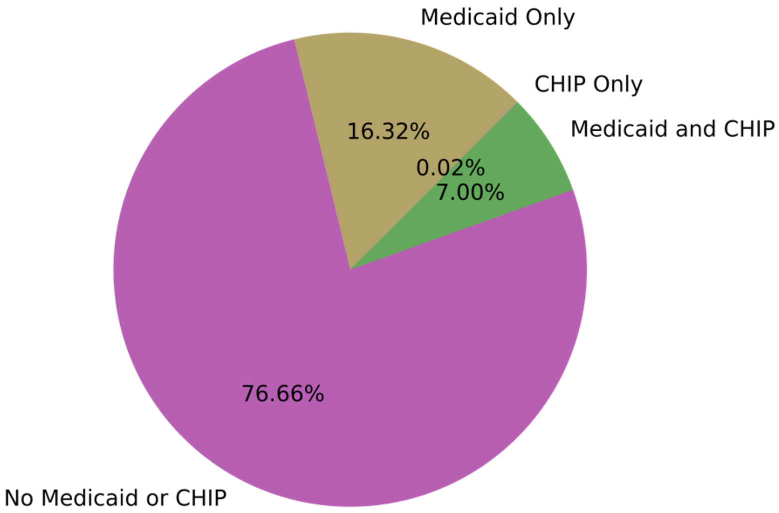

Number of dentists by rurality-urbanicity & participation in public insurance programs

Rurality-Urbanicity	Medicaid Only	CHIP Only	Medicaid and CHIP	No Medicaid or CHIP
Urban	704	1	271	3599
Suburban	131	0	72	422
Rural	54	0	39	158

Number of dentists by provider taxonomy & participation in public insurance programs

Provider Type	Medicaid Only	CHIP Only	Medicaid and CHIP	No Medicaid or CHIP
General	698	0	300	3683
Pediatric	100	0	45	63
Specialist	92	1	37	435

©2022 Serban N et al. *JAMA Network Open*.

Number of dentists

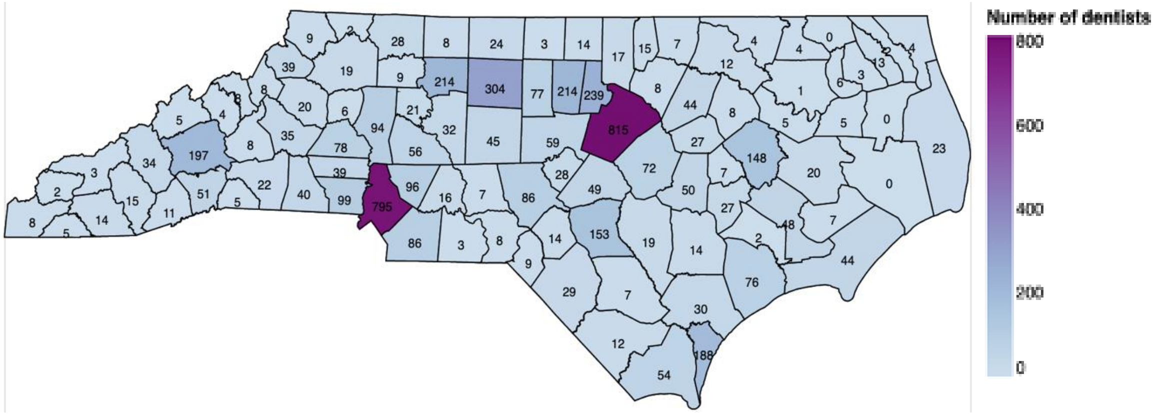

Number of dental hygienists

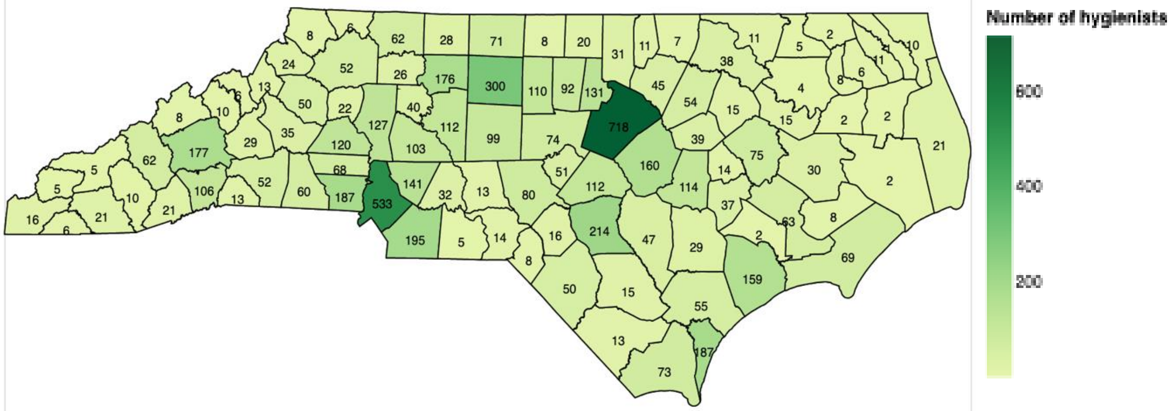

Number of dentists participating in Medicaid

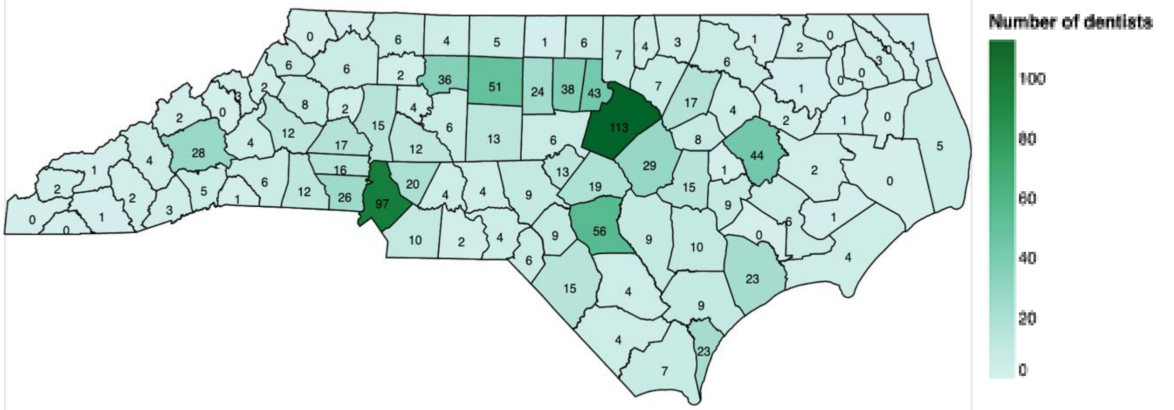

Number of dentists participating in CHIP

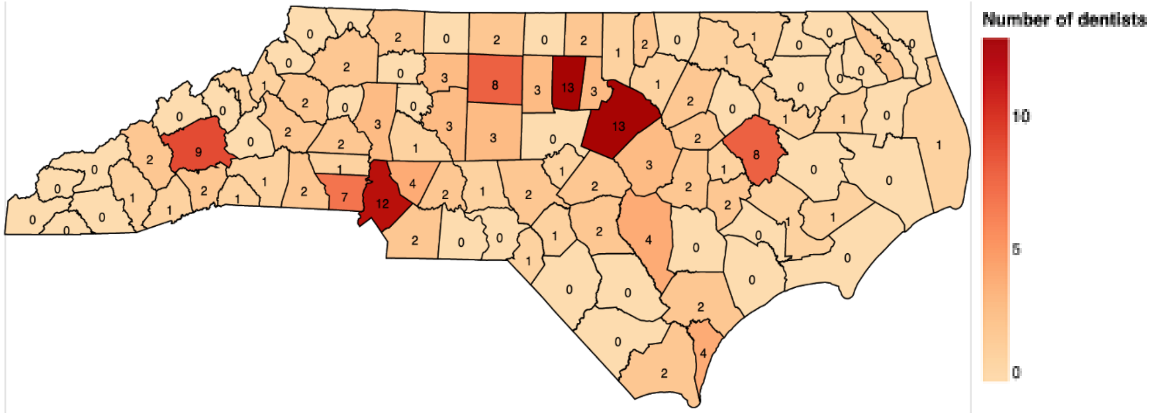

©2022 Serban N et al. *JAMA Network Open*.

Dental Care Supply
NORTH DAKOTA

This state report summarizes data on the dental care supply, differentiated by type of insurance program, provider taxonomy, and rurality-urbanicity of practice address.

Percentage of dentists by provider taxonomy

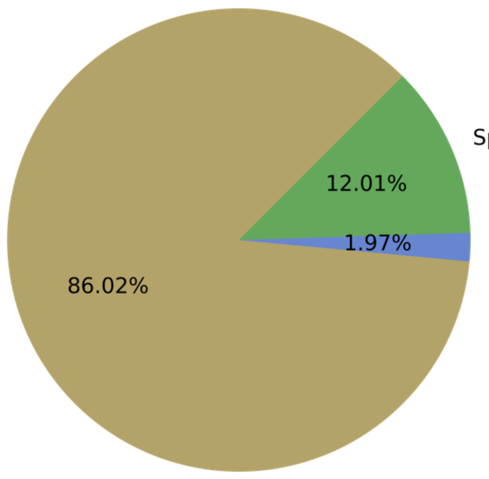

Number of dentists by rurality-urbanicity & provider taxonomy

Rurality-Urbanicity	General	Pediatric	Specialist
**Urban**	264	7	50
**Suburban**	108	3	16
**Rural**	107	1	1

Percentage of dentists by participation in public insurance programs

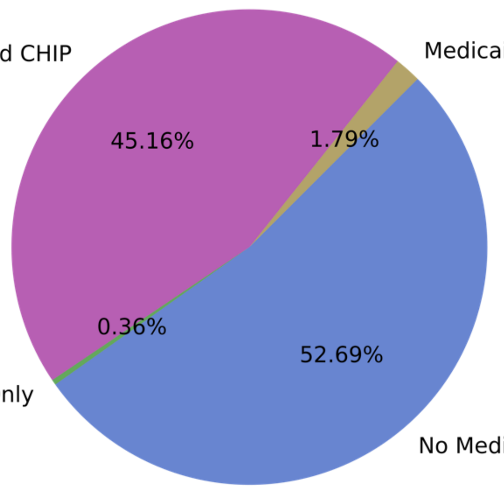

Number of dentists by rurality-urbanicity & participation in public insurance programs

Rurality-Urbanicity	Medicaid Only	CHIP Only	Medicaid and CHIP	No Medicaid or CHIP
**Urban**	3	2	136	180
**Suburban**	3	0	60	64
**Rural**	4	0	56	50

Number of dentists by provider taxonomy & participation in public insurance programs

Provider Type	Medicaid Only	CHIP Only	Medicaid and CHIP	No Medicaid or CHIP
**General**	9	2	212	257
**Pediatric**	0	0	8	3
**Specialist**	1	0	32	34

©2022 Serban N et al. *JAMA Network Open*.

Number of dentists

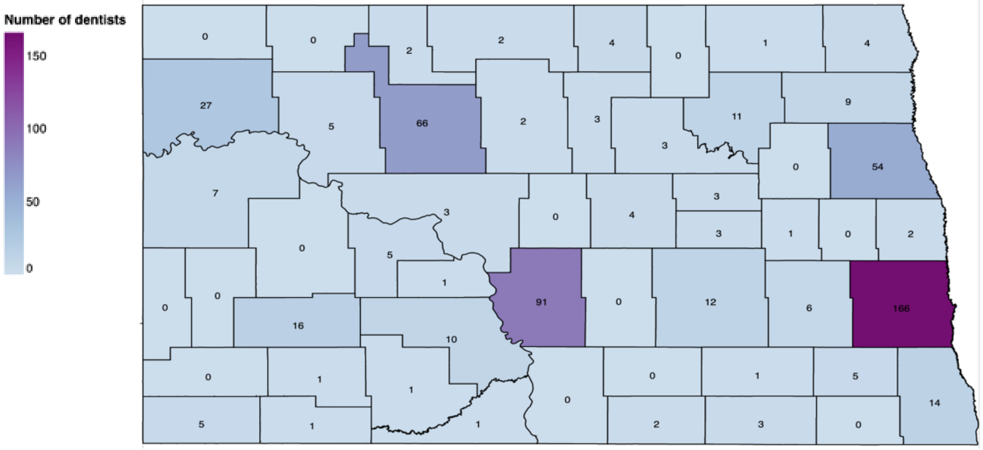

Number of dental hygienists

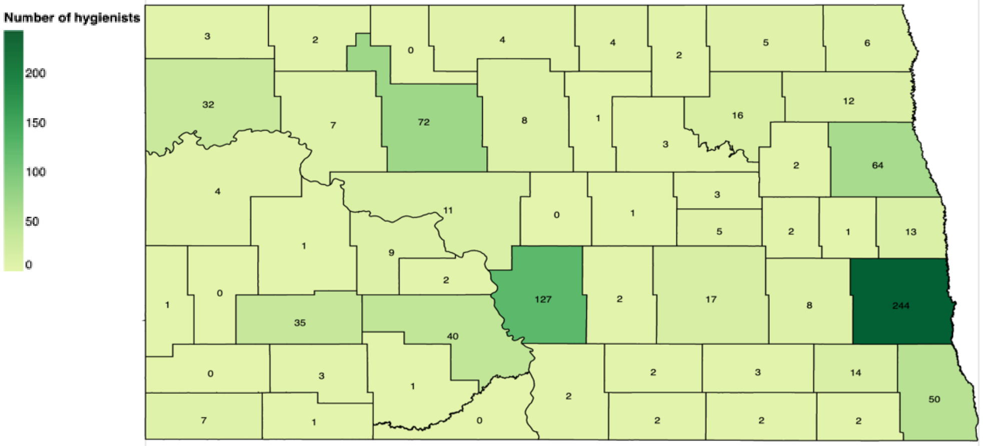

Number of dentists participating in Medicaid

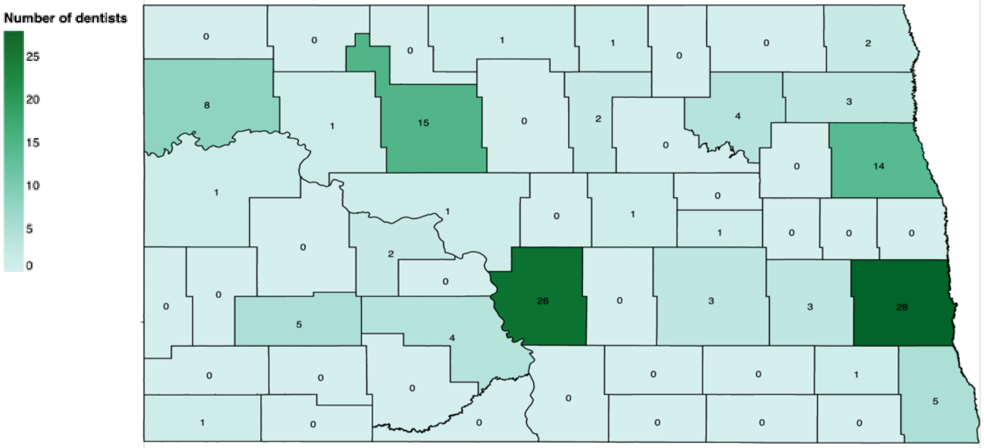

Number of dentists participating in CHIP

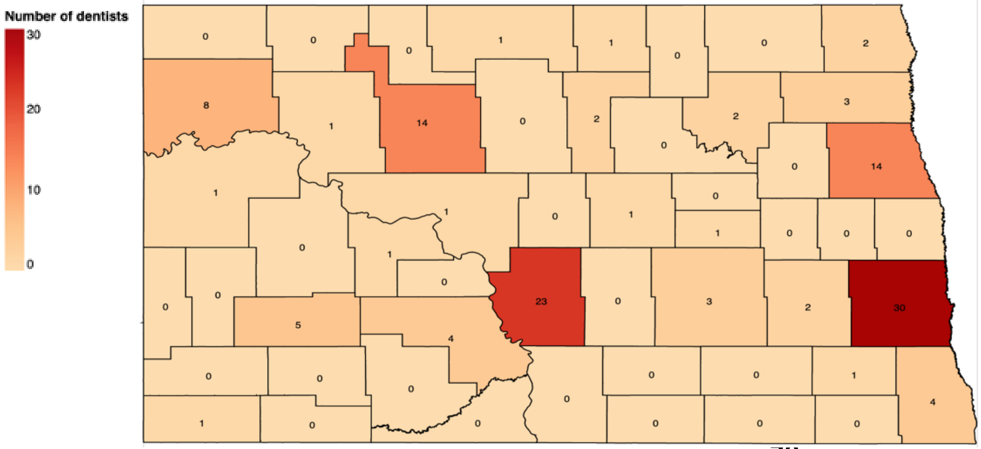

©2022 Serban N et al. *JAMA Network Open*.

Dental Care Supply
OHIO

This state report summarizes data on the dental care supply, differentiated by type of insurance program, provider taxonomy, and rurality-urbanicity of practice address.

Percentage of dentists by provider taxonomy

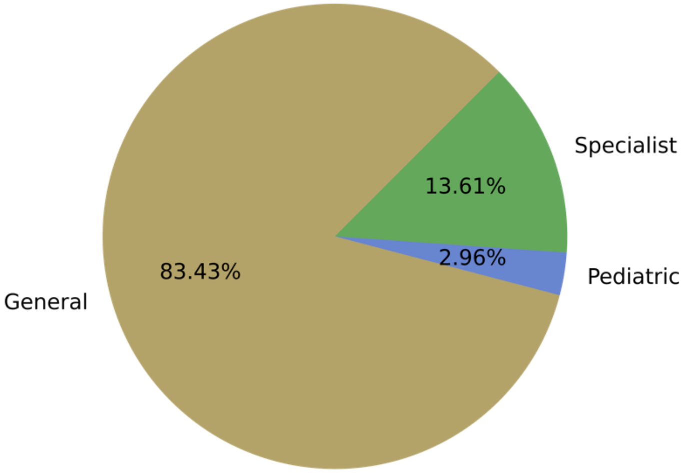

Number of dentists by rurality-urbanicity & provider taxonomy

Rurality-Urbanicity	General	Pediatric	Specialist
Urban	4426	164	760
Suburban	602	19	87
Rural	214	2	8

Percentage of dentists by participation in public insurance programs

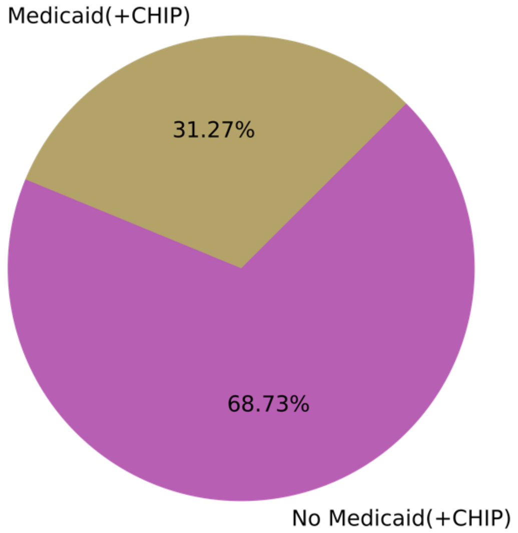

Number of dentists by rurality-urbanicity & participation in public insurance programs

Rurality-Urbanicity	Medicaid(+CHIP)	No Medicaid(+CHIP)
Urban	1581	3769
Suburban	285	423
Rural	99	125

Number of dentists by provider taxonomy & participation in public insurance programs

Provider Type	Medicaid(+CHIP)	No Medicaid(+CHIP)
General	1661	3585
Pediatric	132	54
Specialist	173	683

©2022 Serban N et al. *JAMA Network Open*.

Number of dentists

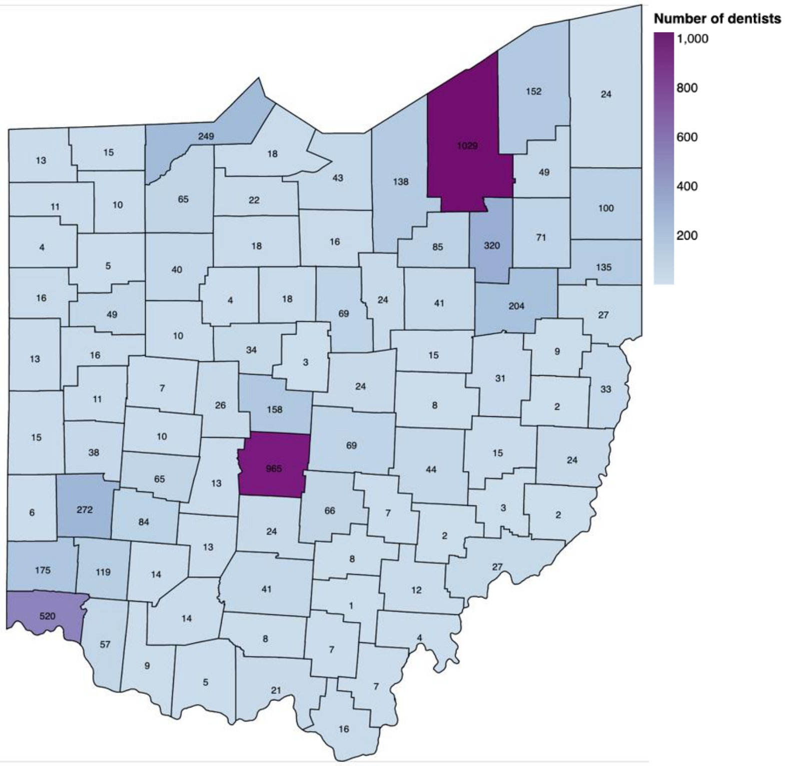

Number of dental hygienists

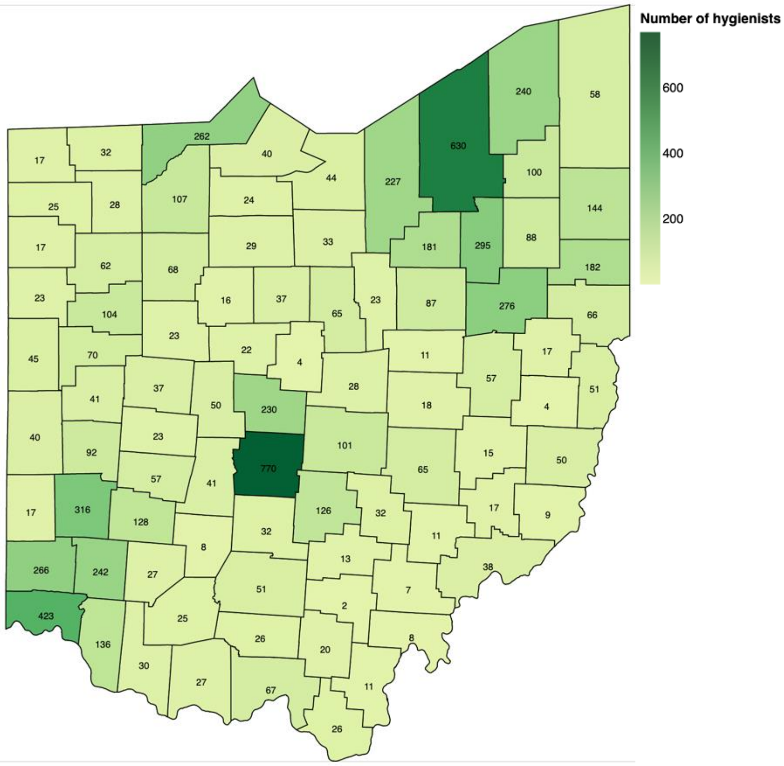

Number of dentists participating in Medicaid(+CHIP)

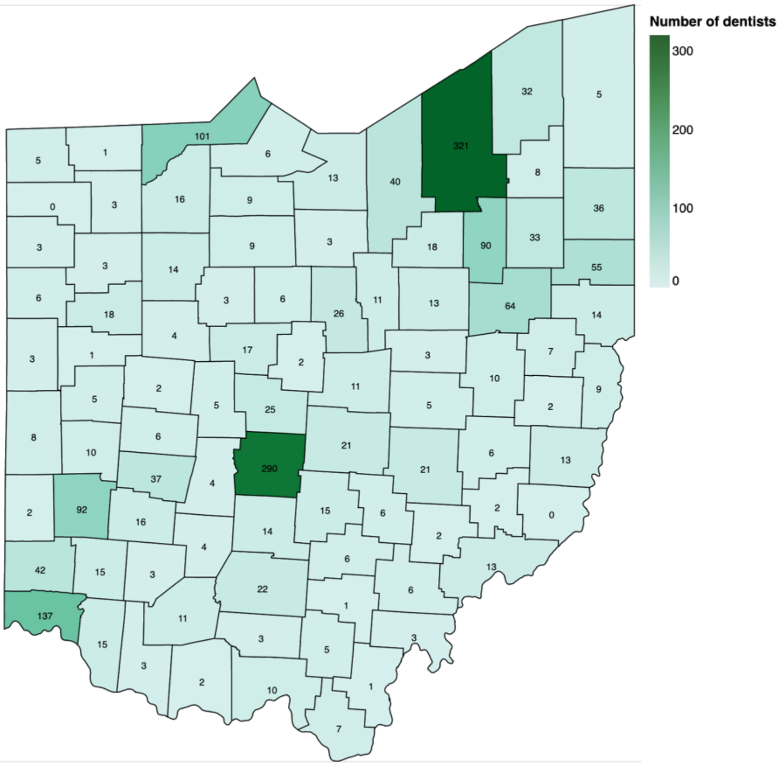

©2022 Serban N et al. *JAMA Network Open*.

Dental Care Supply
OKLAHOMA

This state report summarizes data on the dental care supply, differentiated by type of insurance program, provider taxonomy, and rurality-urbanicity of practice address.

Percentage of dentists by provider taxonomy

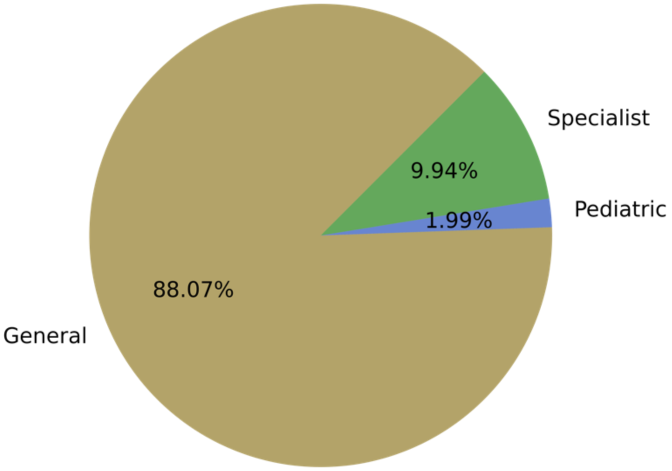

Number of dentists by rurality-urbanicity & provider taxonomy

Rurality-Urbanicity	General	Pediatric	Specialist
Urban	1203	33	172
Suburban	366	7	26
Rural	203	0	1

Percentage of dentists by participation in public insurance programs

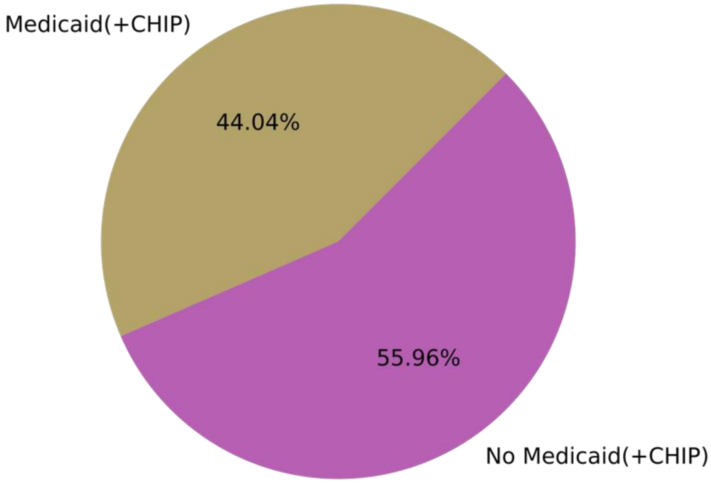

Number of dentists by rurality-urbanicity & participation in public insurance programs

Rurality-Urbanicity	Medicaid(+CHIP)	No Medicaid(+CHIP)
Urban	535	873
Suburban	218	181
Rural	132	72

Number of dentists by provider taxonomy & participation in public insurance programs

Provider Type	Medicaid(+CHIP)	No Medicaid(+CHIP)
General	773	999
Pediatric	24	16
Specialist	89	111

©2022 Serban N et al. *JAMA Network Open*.

Number of dentists

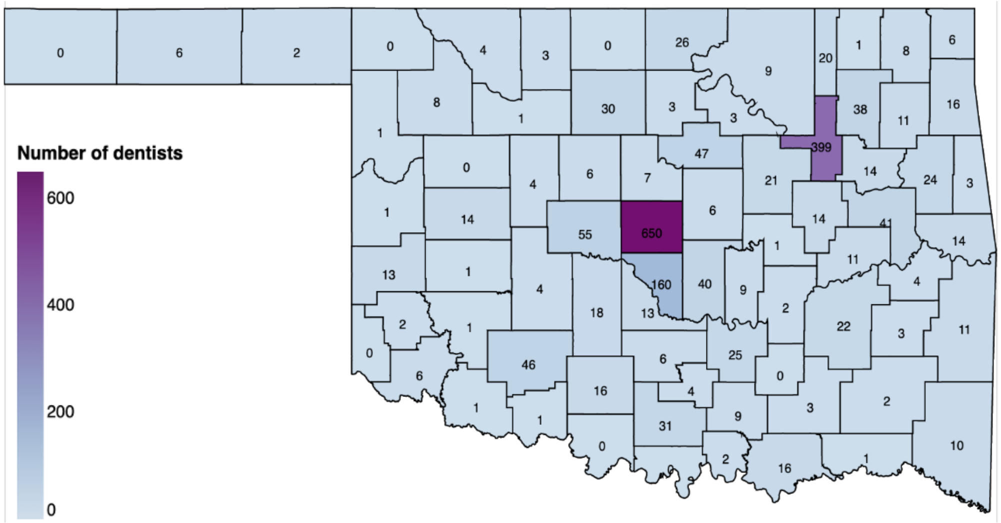

Number of dental hygienists

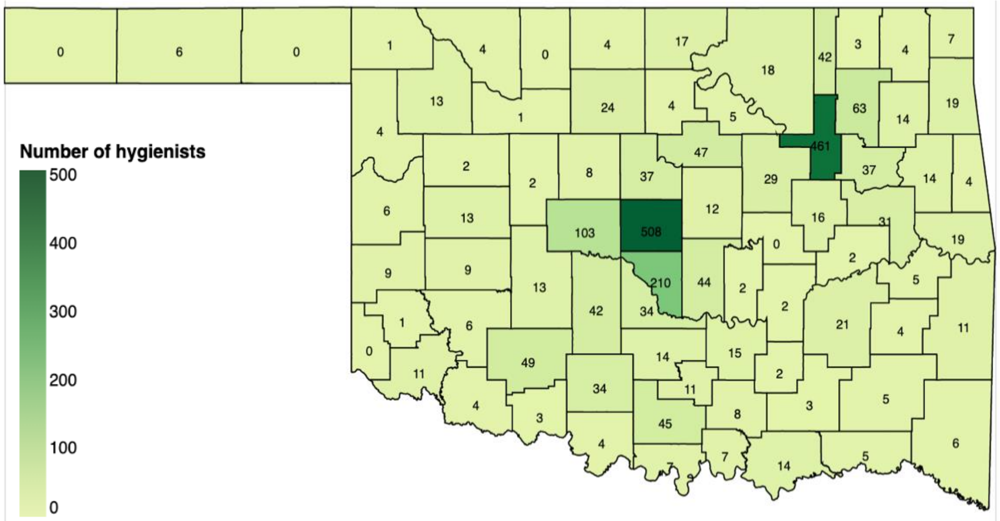

Number of dentists participating in Medicaid(+CHIP)

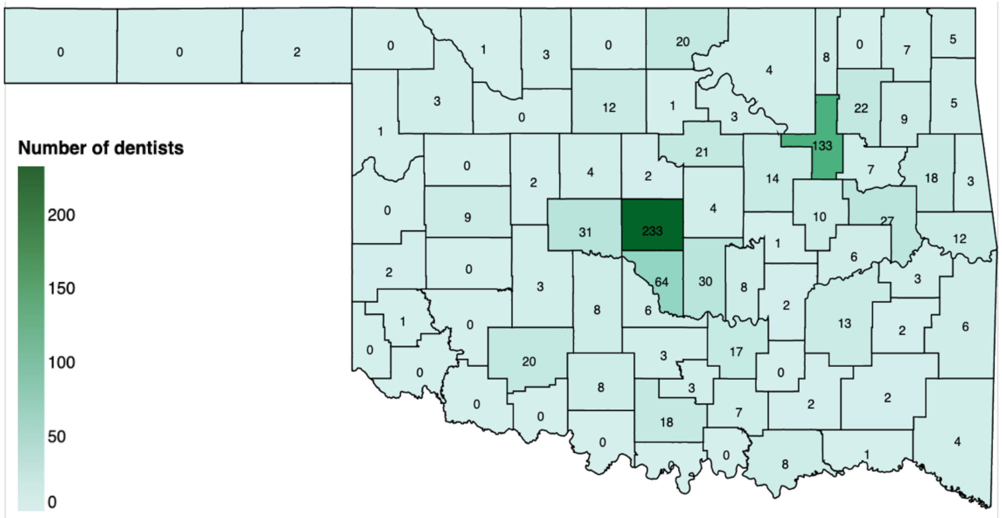

©2022 Serban N et al. *JAMA Network Open*.

Dental Care Supply
OREGON

This state report summarizes data on the dental care supply, differentiated by type of insurance program, provider taxonomy, and rurality-urbanicity of practice address.

Percentage of dentists by provider taxonomy

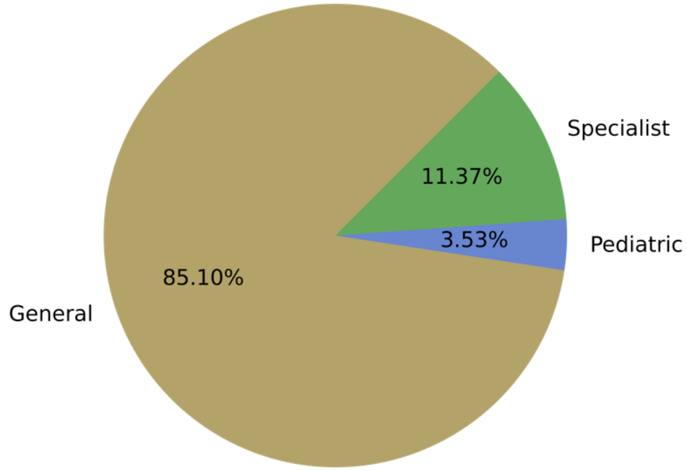

Number of dentists by rurality-urbanicity & provider taxonomy

Rurality-Urbanicity	General	Pediatric	Specialist
Urban	2001	85	290
Suburban	309	13	28
Rural	123	3	7

Percentage of dentists by participation in public insurance programs

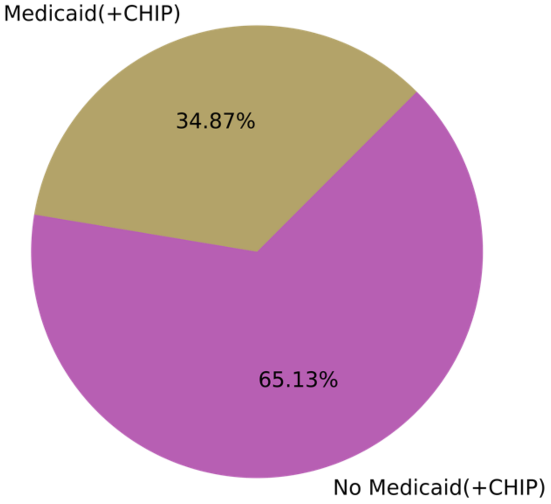

Number of dentists by rurality-urbanicity & participation in public insurance programs

Rurality-Urbanicity	Medicaid(+CHIP)	No Medicaid(+CHIP)
Urban	820	1556
Suburban	128	222
Rural	49	84

Number of dentists by provider taxonomy & participation in public insurance programs

Provider Type	Medicaid(+CHIP)	No Medicaid(+CHIP)
General	824	1609
Pediatric	66	35
Specialist	107	218

©2022 Serban N et al. *JAMA Network Open*.

Number of dentists

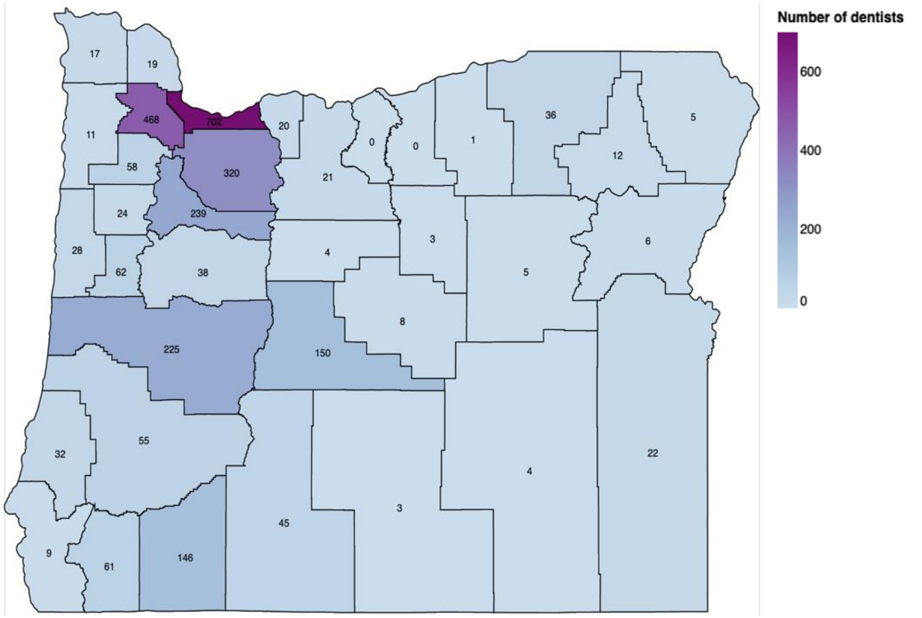

Number of dental hygienists

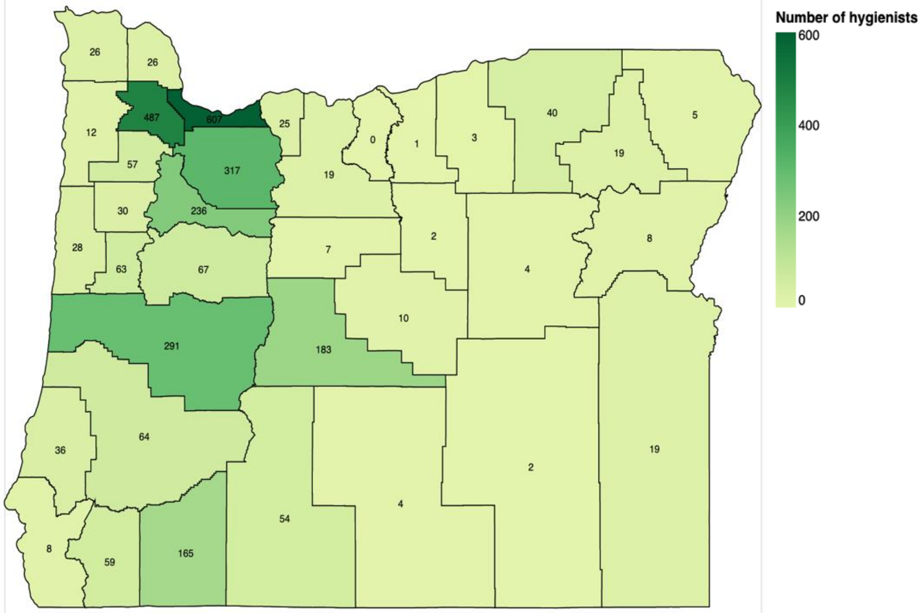

Number of dentists participating in Medicaid(+CHIP)

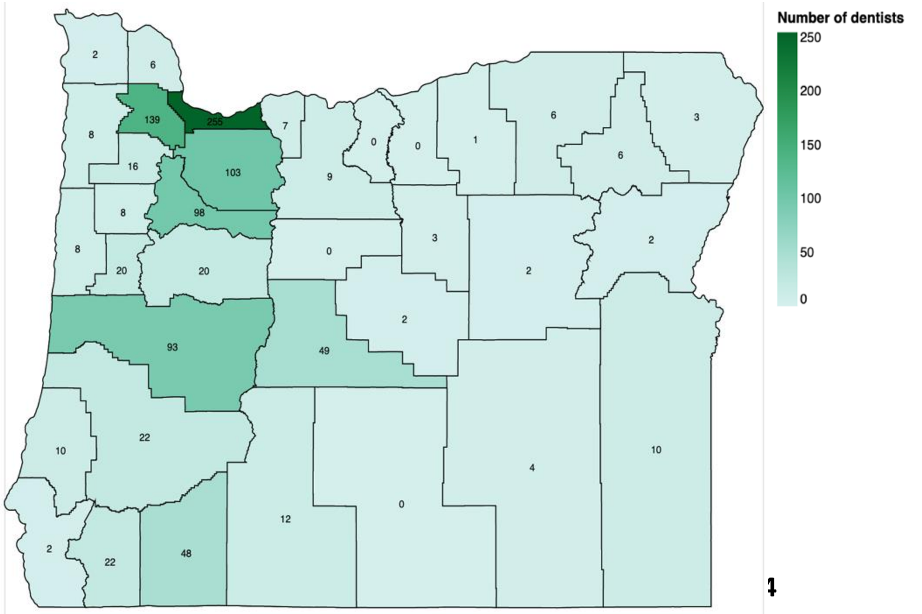

©2022 Serban N et al. *JAMA Network Open*.

Dental Care Supply
PENNSYLVANIA

This state report summarizes data on the dental care supply, differentiated by type of insurance program, provider taxonomy, and rurality-urbanicity of practice address.

Percentage of dentists by provider taxonomy

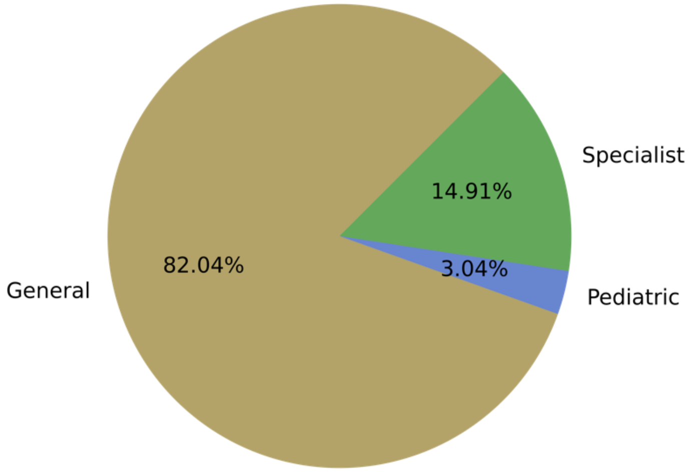

Number of dentists by rurality-urbanicity & provider taxonomy

Rurality-Urbanicity	General	Pediatric	Specialist
Urban	5940	237	1131
Suburban	410	3	42
Rural	202	3	18

Percentage of dentists by participation in public insurance programs

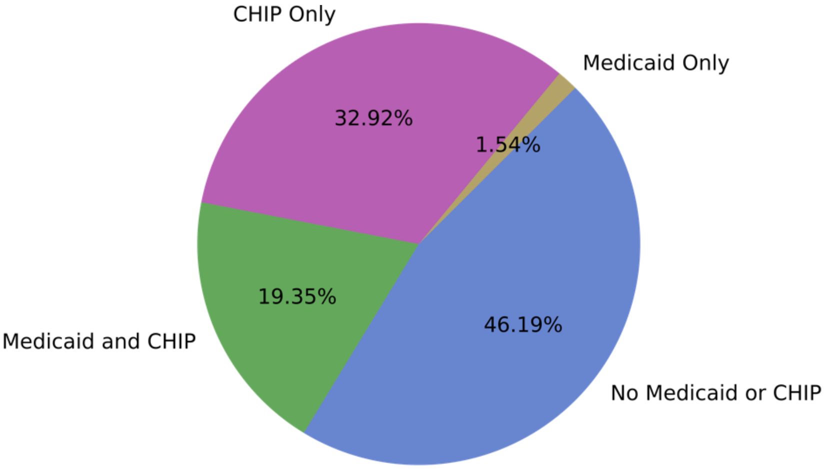

Number of dentists by rurality-urbanicity & participation in public insurance programs

Rurality-Urbanicity	Medicaid Only	CHIP Only	Medicaid and CHIP	No Medicaid or CHIP
Urban	109	2362	1392	3445
Suburban	10	187	85	173
Rural	4	81	68	70

Number of dentists by provider taxonomy & participation in public insurance programs

Provider Type	Medicaid Only	CHIP Only	Medicaid and CHIP	No Medicaid or CHIP
General	107	2231	1177	3037
Pediatric	8	52	120	63
Specialist	8	346	248	588

©2022 Serban N et al. *JAMA Network Open*.

Number of dentists

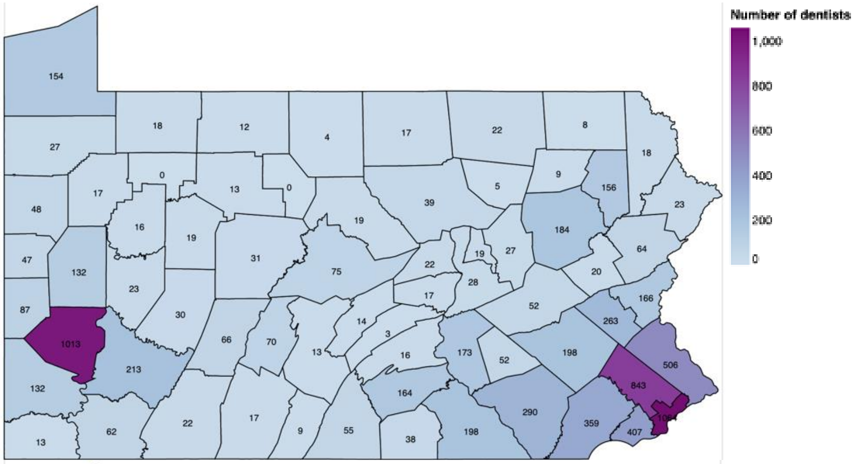

Number of dental hygienists

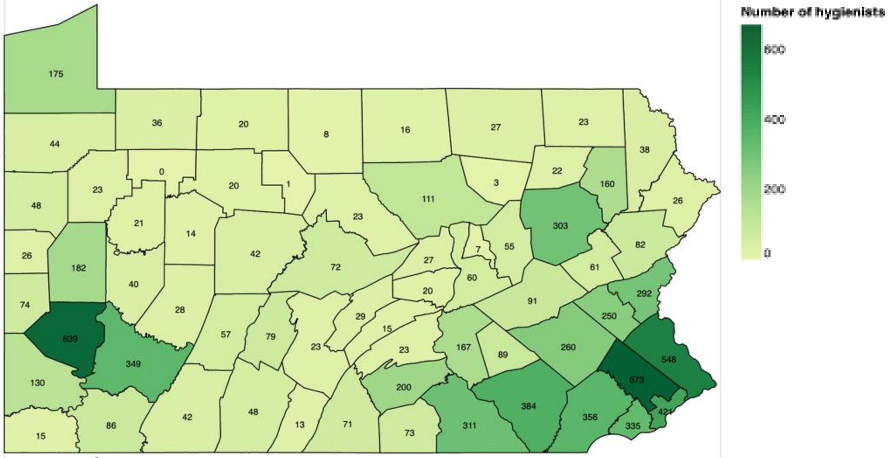

Number of dentists participating in Medicaid

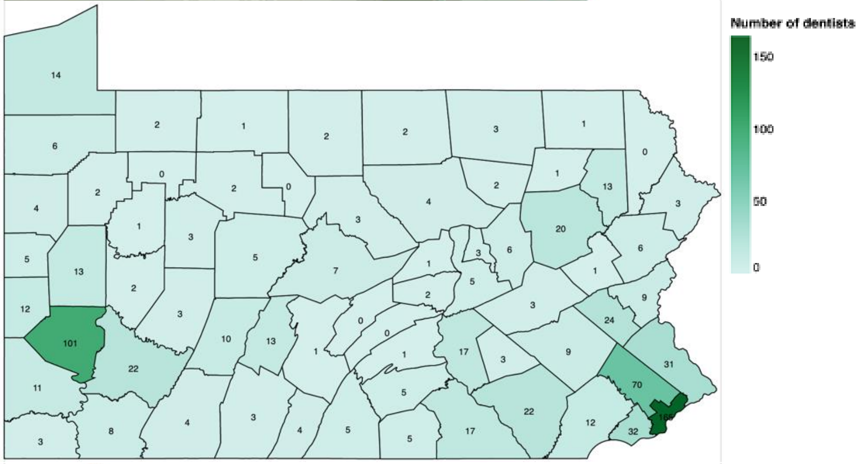

Number of dentists participating in CHIP

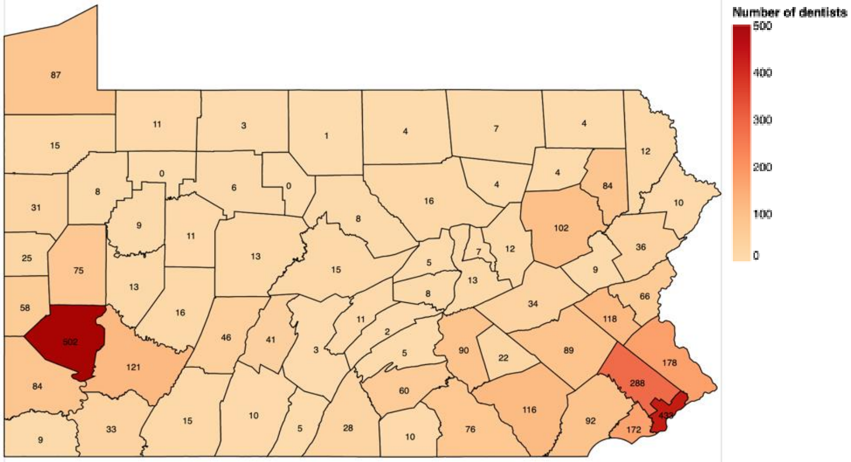

©2022 Serban N et al. *JAMA Network Open*.

Dental Care Supply
RHODE ISLAND

This state report summarizes data on the dental care supply, differentiated by type of insurance program, provider taxonomy, and rurality-urbanicity of practice address.

Percentage of dentists by provider taxonomy

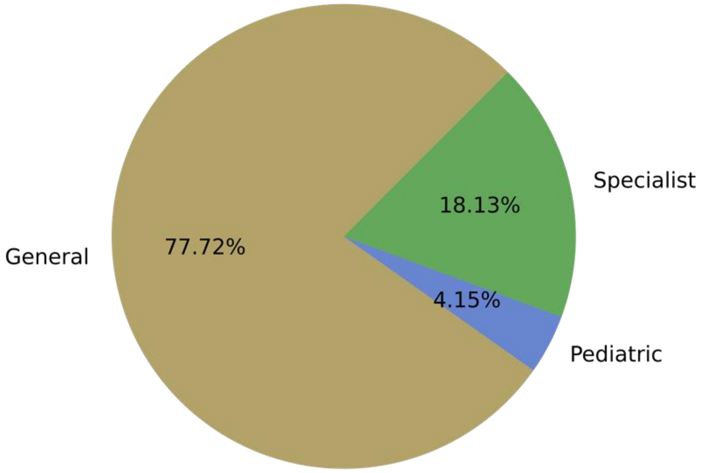

Number of dentists by rurality-urbanicity & provider taxonomy

Rurality-Urbanicity	General	Pediatric	Specialist
Urban	449	24	105
Suburban	0	0	0
Rural	1	0	0

Percentage of dentists by participation in public insurance programs

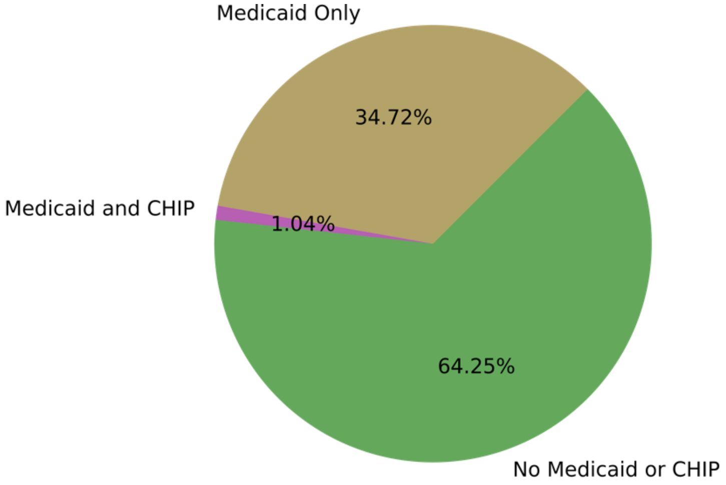

Number of dentists by rurality-urbanicity & participation in public insurance programs

Rurality-Urbanicity	Medicaid Only	CHIP Only	Medicaid and CHIP	No Medicaid or CHIP
Urban	201	0	6	371
Suburban	0	0	0	0
Rural	0	0	0	1

Number of dentists by provider taxonomy & participation in public insurance programs

Provider Type	Medicaid Only	CHIP Only	Medicaid and CHIP	No Medicaid or CHIP
General	151	0	6	293
Pediatric	22	0	0	2
Specialist	28	0	0	77

©2022 Serban N et al. *JAMA Network Open*.

Number of dentists

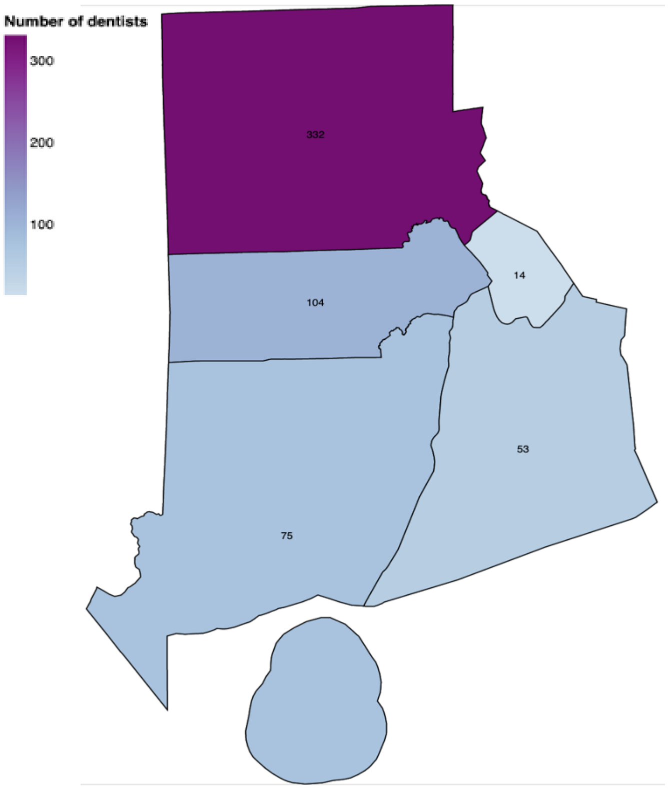

Number of dental hygienists

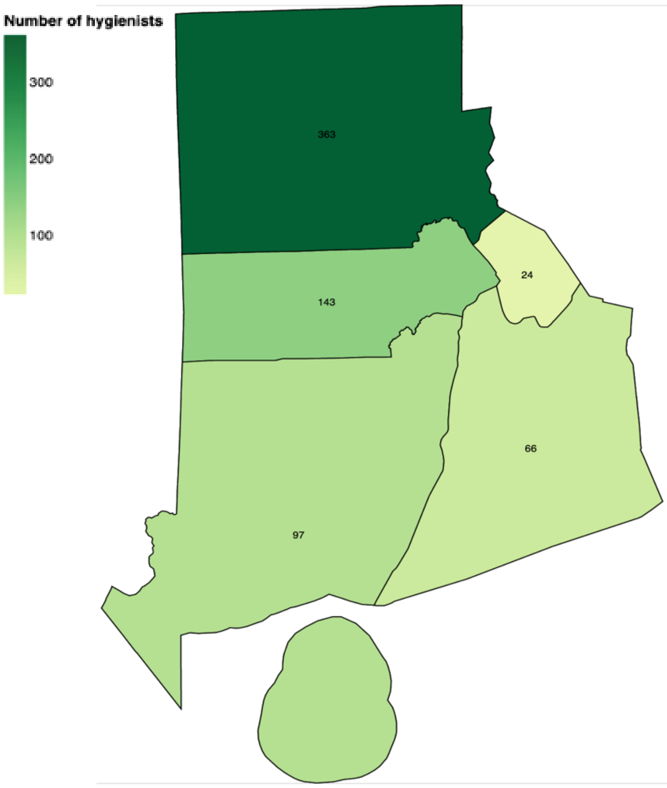

Number of dentists participating in Medicaid

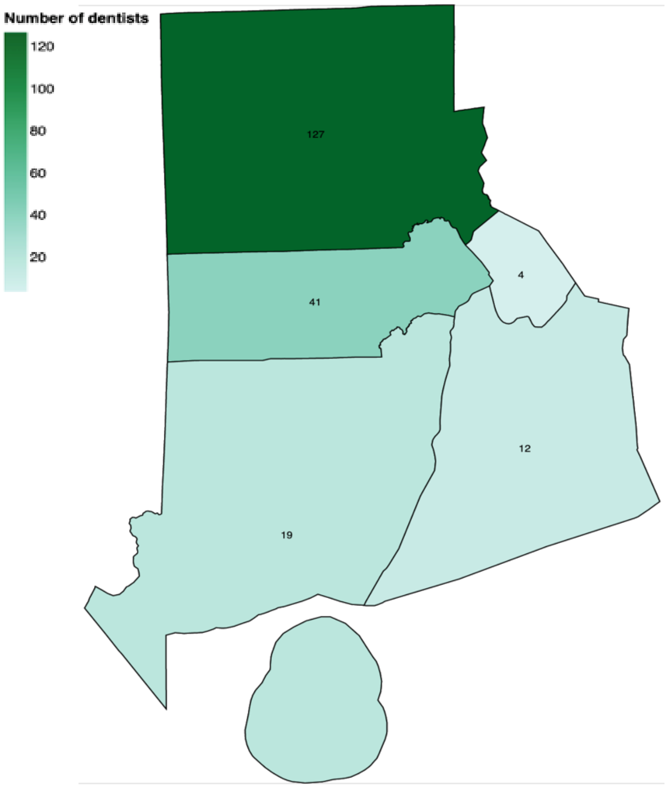

Number of dentists participating in CHIP

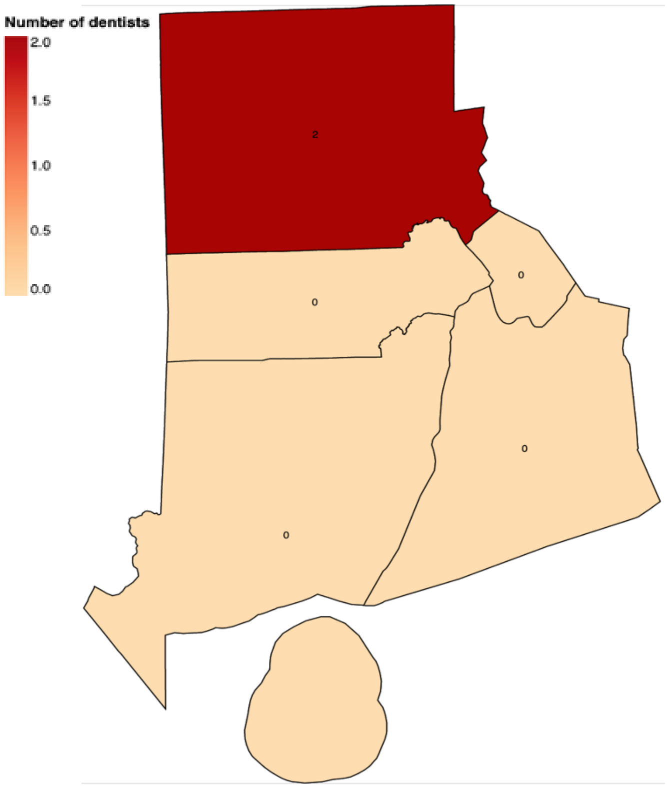

©2022 Serban N et al. *JAMA Network Open*.

Dental Care Supply
SOUTH CAROLINA

This state report summarizes data on the dental care supply, differentiated by type of insurance program, provider taxonomy, and rurality-urbanicity of practice address.

Percentage of dentists by provider taxonomy

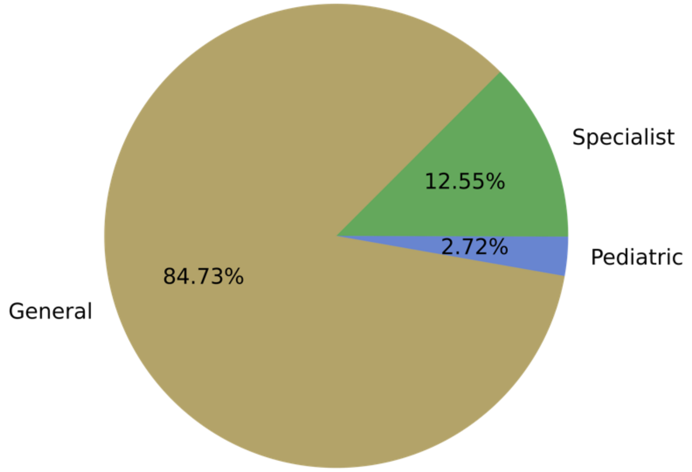

Number of dentists by rurality-urbanicity & provider taxonomy

Rurality-Urbanicity	General	Pediatric	Specialist
Urban	1931	67	302
Suburban	257	5	32
Rural	86	1	3

Percentage of dentists by participation in public insurance programs

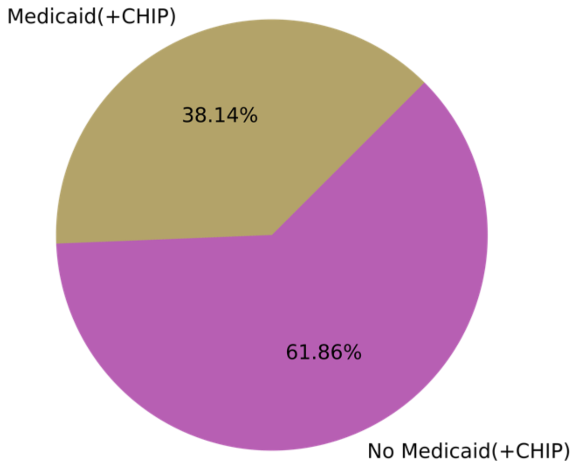

Number of dentists by rurality-urbanicity & participation in public insurance programs

Rurality-Urbanicity	Medicaid(+CHIP)	No Medicaid(+CHIP)
Urban	801	1500
Suburban	149	145
Rural	74	16

Number of dentists by provider taxonomy & participation in public insurance programs

Provider Type	Medicaid(+CHIP)	No Medicaid(+CHIP)
General	871	1404
Pediatric	55	18
Specialist	98	239

©2022 Serban N et al. *JAMA Network Open*.

Number of dentists

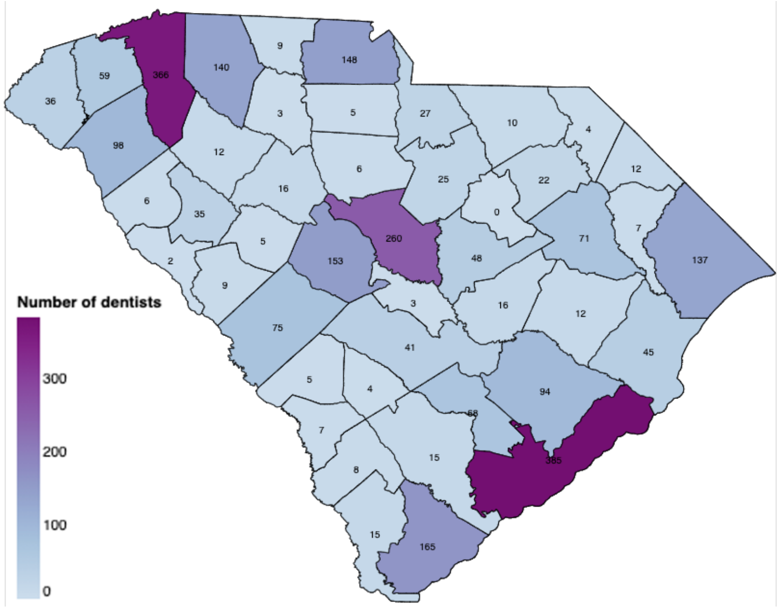

Number of dental hygienists

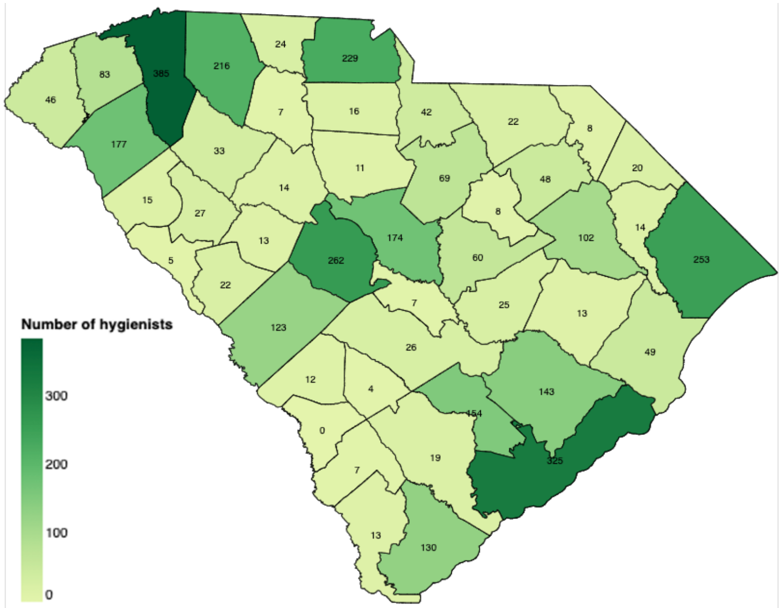

Number of dentists participating in Medicaid(+CHIP)

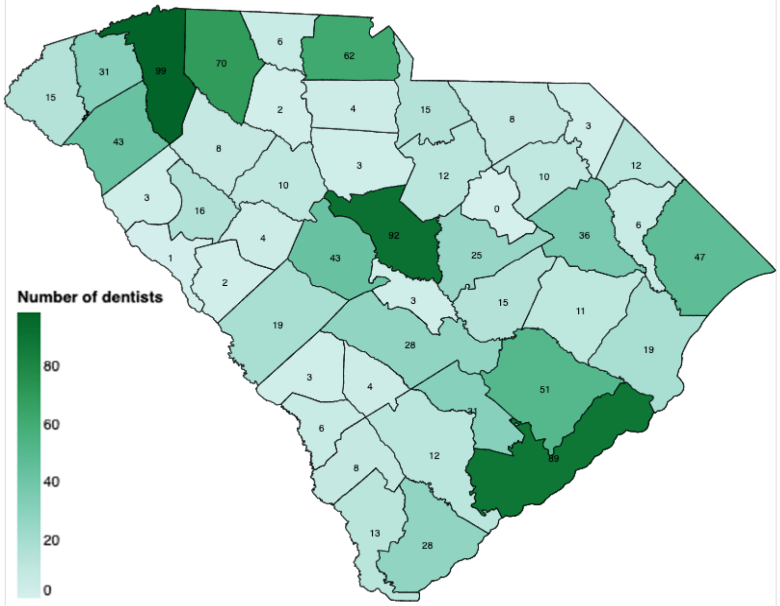

©2022 Serban N et al. *JAMA Network Open*.

Dental Care Supply
SOUTH DAKOTA

This state report summarizes data on the dental care supply, differentiated by type of insurance program, provider taxonomy, and rurality-urbanicity of practice address.

Percentage of dentists by provider taxonomy

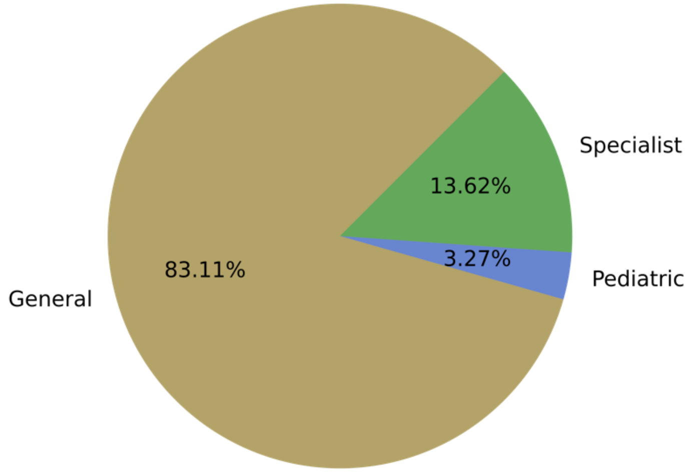

Number of dentists by rurality-urbanicity & provider taxonomy

Rurality-Urbanicity	General	Pediatric	Specialist
**Urban**	142	8	34
**Suburban**	87	2	14
**Rural**	76	1	2

Percentage of dentists by participation in public insurance programs

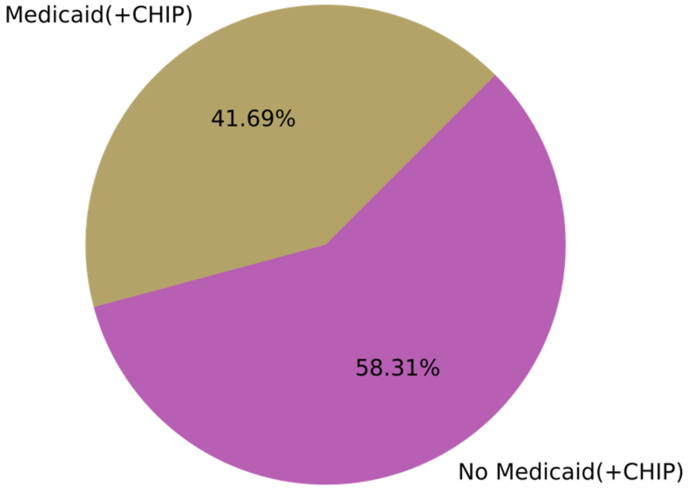

Number of dentists by rurality-urbanicity & participation in public insurance programs

Rurality-Urbanicity	Medicaid(+CHIP)	No Medicaid(+CHIP)
**Urban**	64	121
**Suburban**	51	53
**Rural**	39	40

Number of dentists by provider taxonomy & participation in public insurance programs

Provider Type	Medicaid(+CHIP)	No Medicaid(+CHIP)
**General**	119	186
**Pediatric**	10	2
**Specialist**	24	26

©2022 Serban N et al. *JAMA Network Open*.

Number of dentists

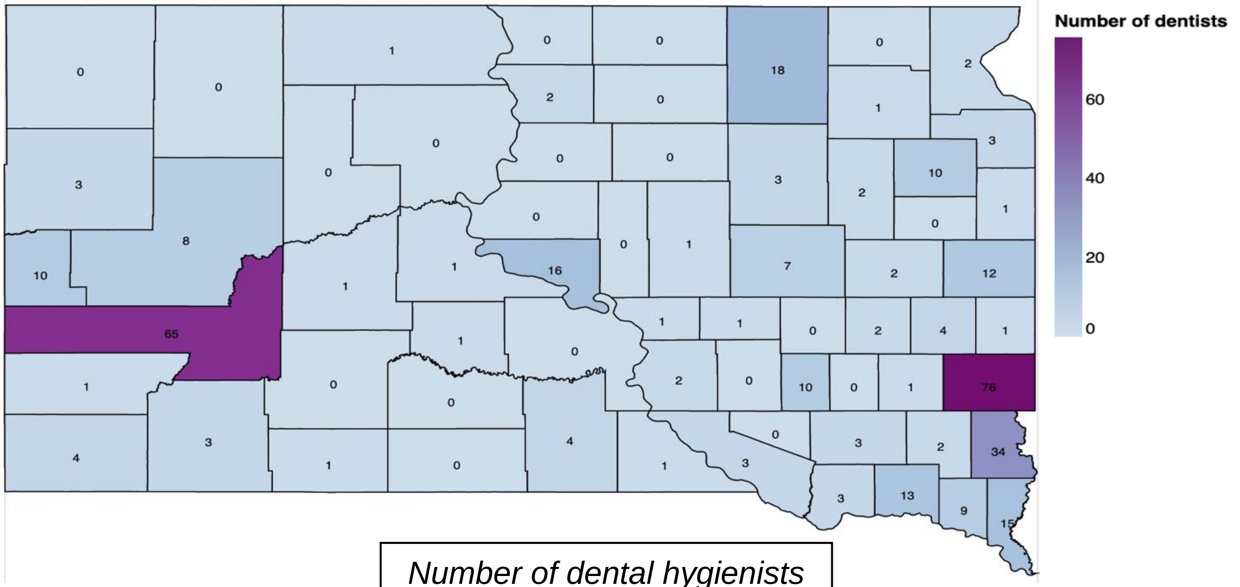

Number of dental hygienists

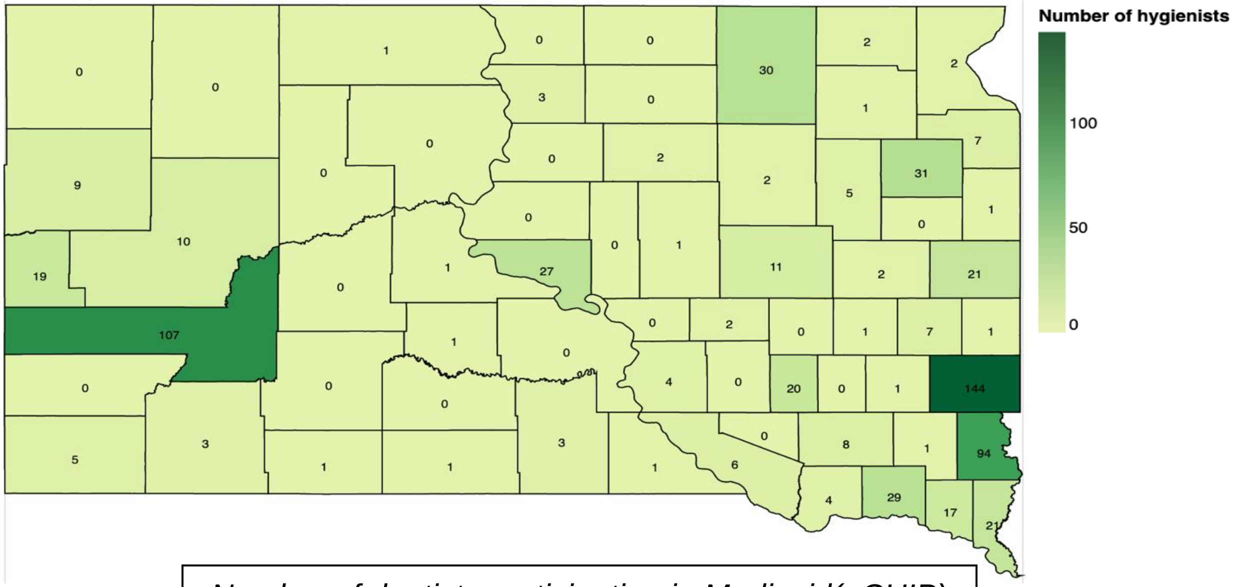

Number of dentists participating in Medicaid(+CHIP)

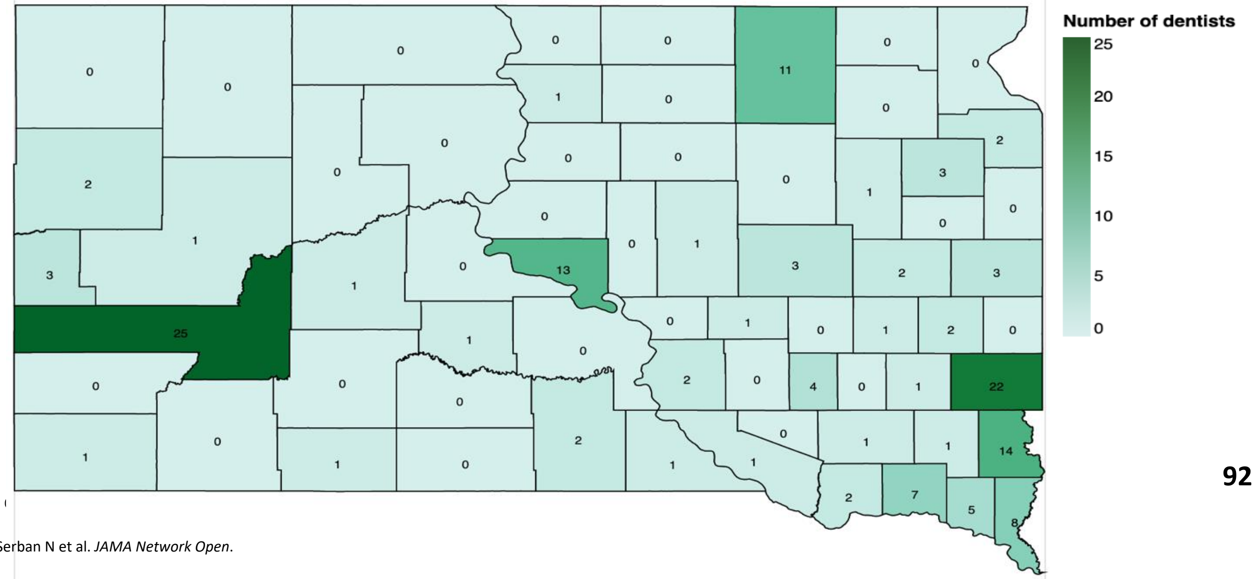

©2022 Serban N et al. *JAMA Network Open*.

Dental Care Supply
TENNESSEE

This state report summarizes data on the dental care supply, differentiated by type of insurance program, provider taxonomy, and rurality-urbanicity of practice address.

Percentage of dentists by provider taxonomy

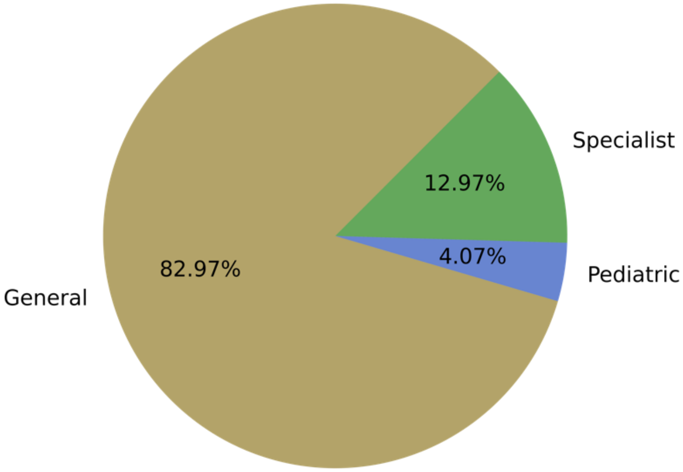

Number of dentists by rurality-urbanicity & provider taxonomy

Rurality-Urbanicity	General	Pediatric	Specialist
**Urban**	2003	97	331
**Suburban**	304	18	47
**Rural**	182	7	12

Percentage of dentists by participation in public insurance programs

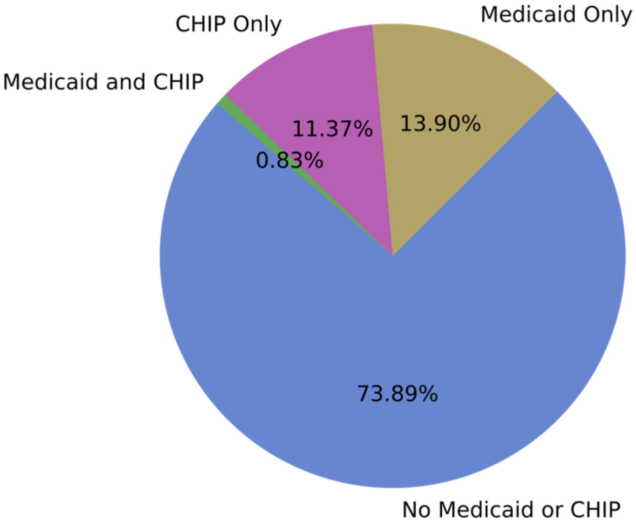

Number of dentists by rurality-urbanicity & participation in public insurance programs

Rurality-Urbanicity	Medicaid Only	CHIP Only	Medicaid and CHIP	No Medicaid or CHIP
**Urban**	290	265	17	1860
**Suburban**	86	55	4	224
**Rural**	42	22	4	132

Number of dentists by provider taxonomy & participation in public insurance programs

Provider Type	Medicaid Only	CHIP Only	Medicaid and CHIP	No Medicaid or CHIP
**General**	279	252	16	1942
**Pediatric**	48	39	3	31
**Specialist**	90	50	6	243

©2022 Serban N et al. *JAMA Network Open*.

Number of dentists

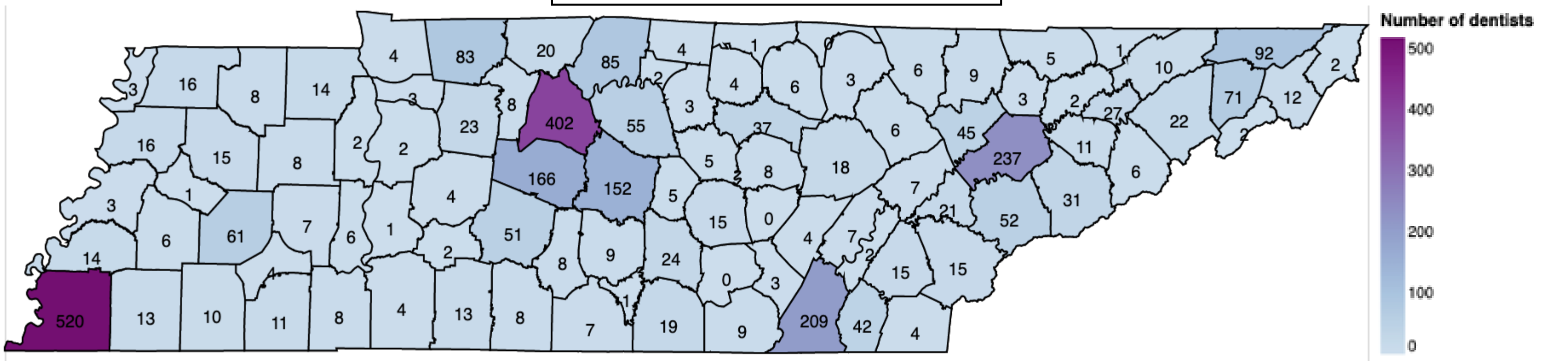

Number of dental hygienists

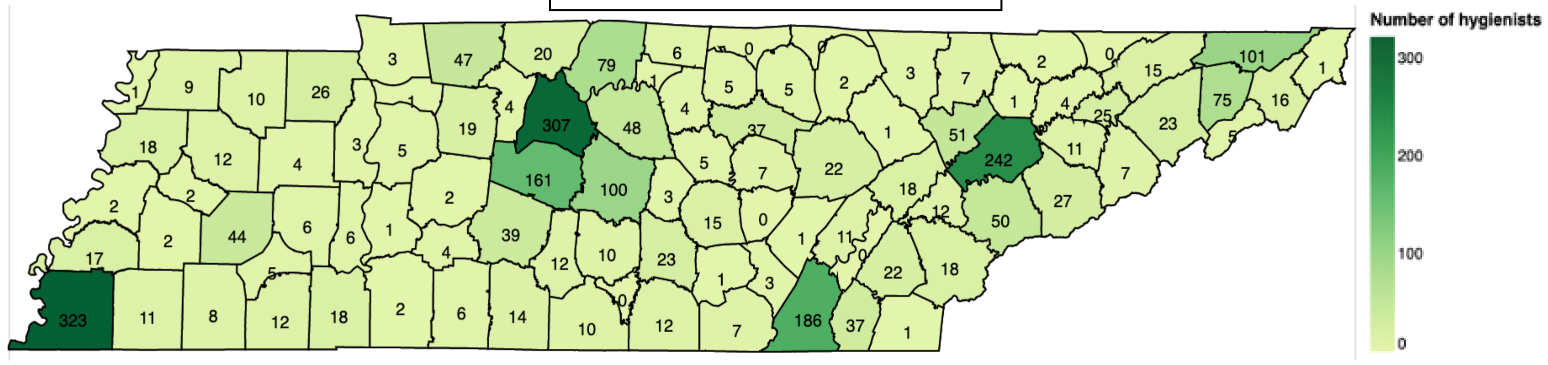

Number of dentists participating in Medicaid

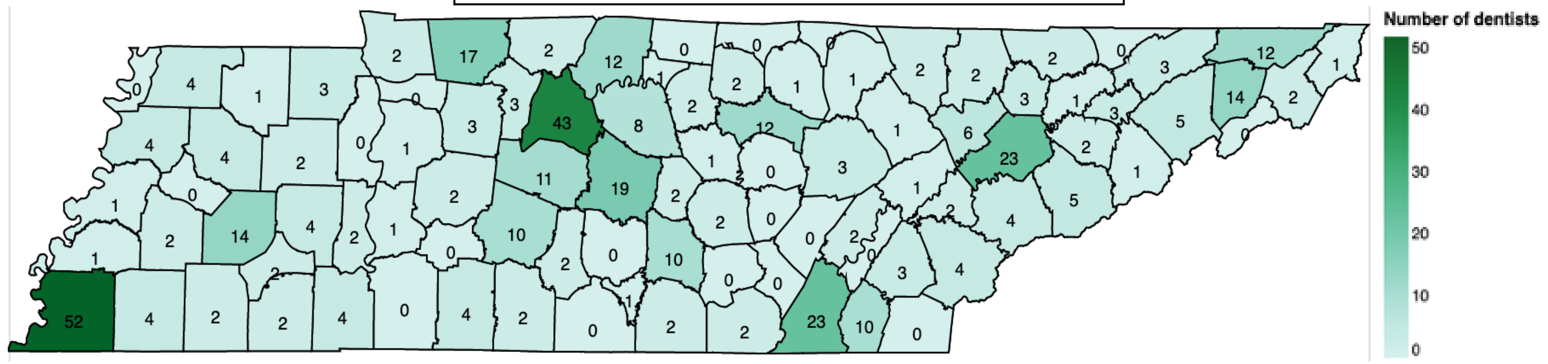

Number of dentists participating in CHIP

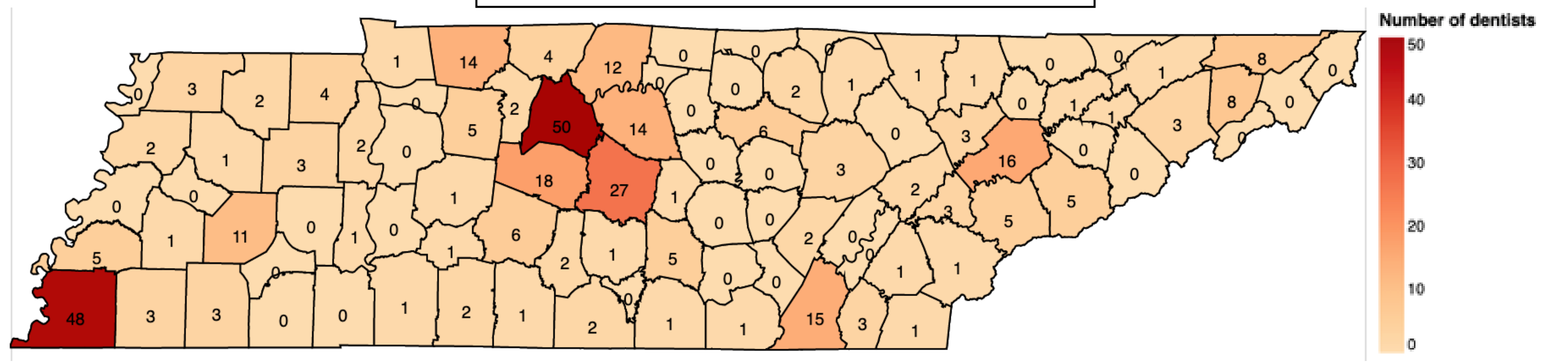

©2022 Serban N et al. *JAMA Network Open*.

Dental Care Supply
TEXAS

This state report summarizes data on the dental care supply, differentiated by type of insurance program, provider taxonomy, and rurality-urbanicity of practice address.

Percentage of dentists by provider taxonomy

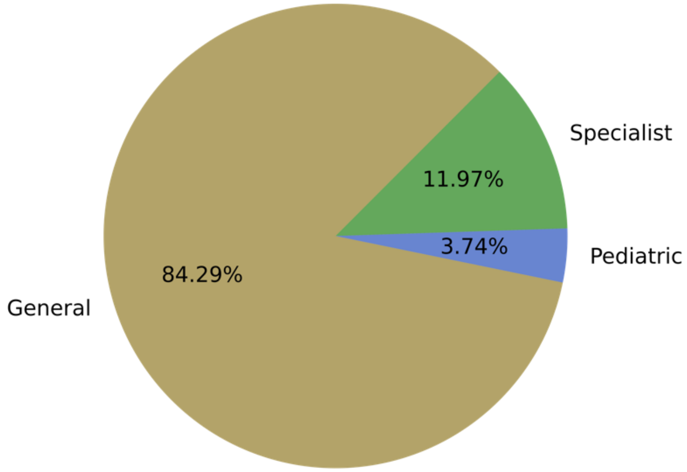

Number of dentists by rurality-urbanicity & provider taxonomy

Rurality-Urbanicity	General	Pediatric	Specialist
Urban	12406	564	1846
Suburban	691	25	45
Rural	376	9	23

Percentage of dentists by participation in public insurance programs

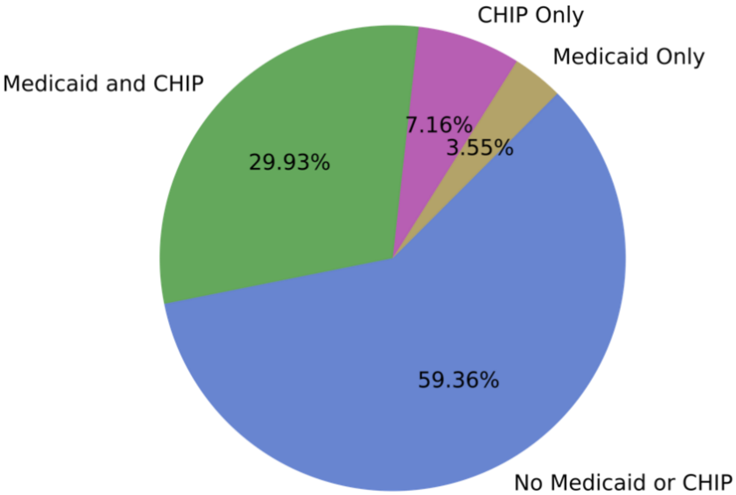

Number of dentists by rurality-urbanicity & participation in public insurance programs

Rurality-Urbanicity	Medicaid Only	CHIP Only	Medicaid and CHIP	No Medicaid or CHIP
Urban	526	1076	4428	8786
Suburban	29	39	260	432
Rural	13	30	96	270

Number of dentists by provider taxonomy & participation in public insurance programs

Provider Type	Medicaid Only	CHIP Only	Medicaid and CHIP	No Medicaid or CHIP
General	497	897	4092	7987
Pediatric	18	44	392	144
Specialist	53	204	300	1357

©2022 Serban N et al. *JAMA Network Open*.

Number of dentists

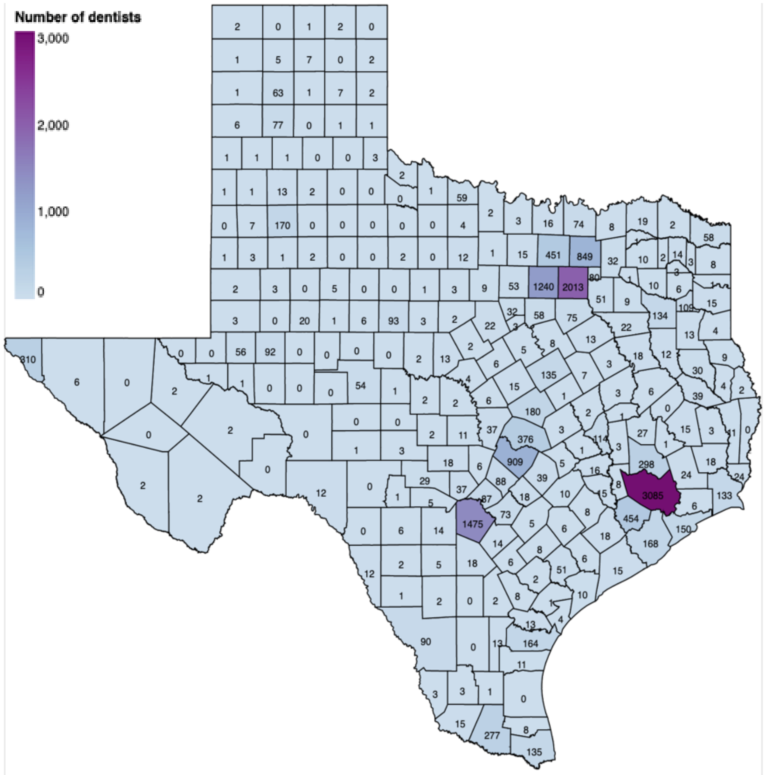

Number of dental hygienists

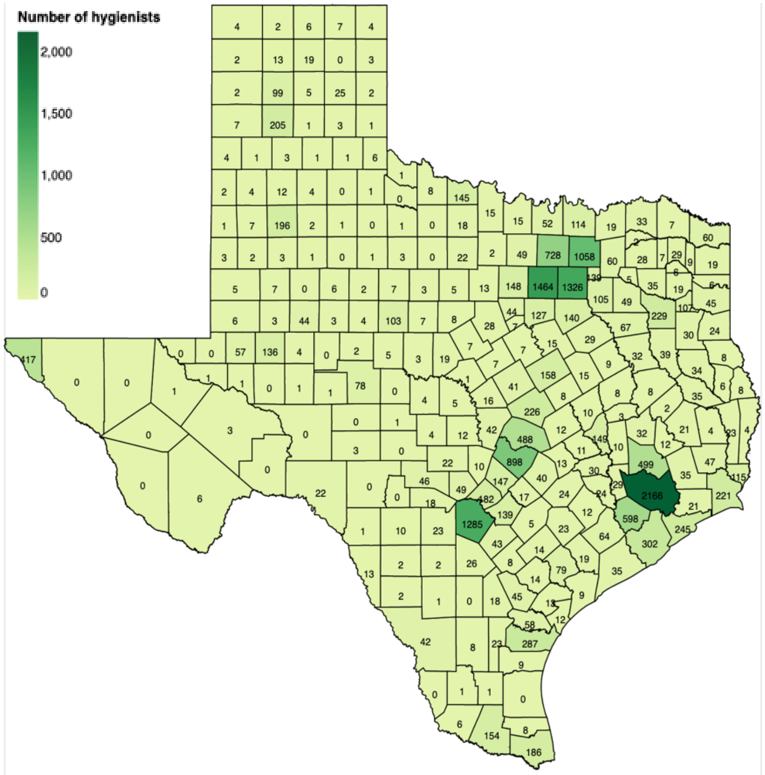

Number of dentists participating in Medicaid

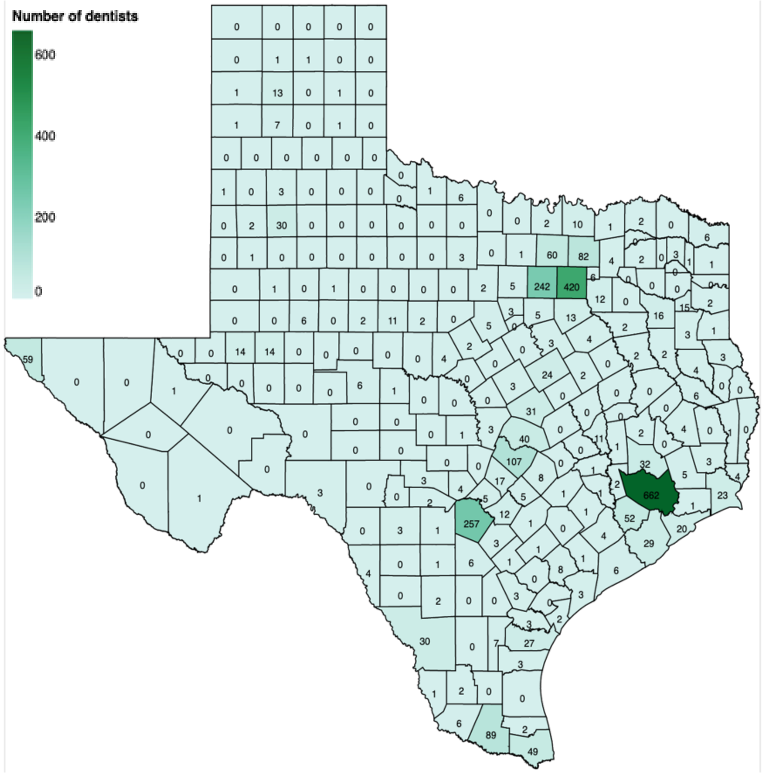

Number of dentists participating in CHIP

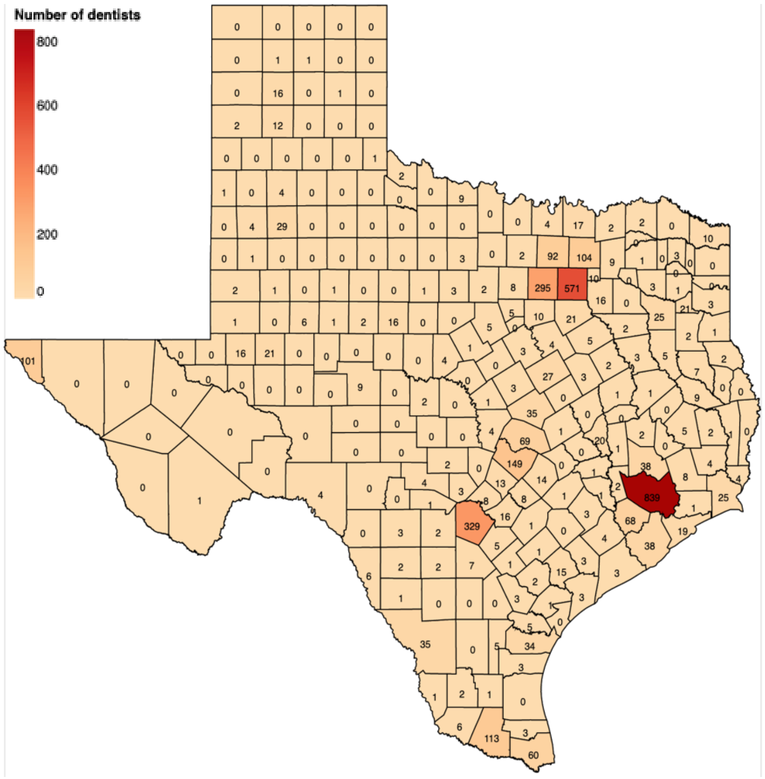

©2022 Serban N et al. *JAMA Network Open*.

Dental Care Supply
UTAH

This state report summarizes data on the dental care supply, differentiated by type of insurance program, provider taxonomy, and rurality-urbanicity of practice address.

Percentage of dentists by provider taxonomy

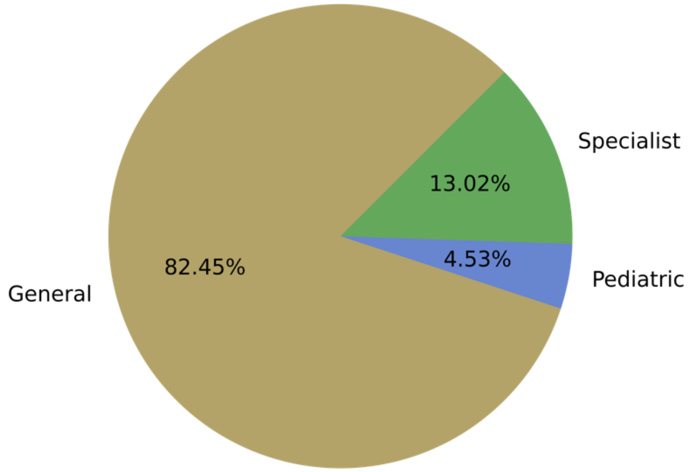

Number of dentists by rurality-urbanicity & provider taxonomy

Rurality-Urbanicity	General	Pediatric	Specialist
Urban	1691	98	278
Suburban	143	7	19
Rural	98	1	8

Percentage of dentists by participation in public insurance programs

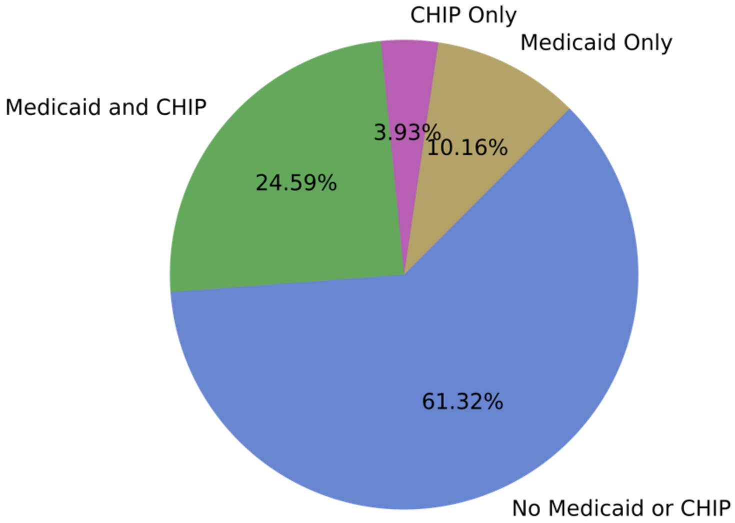

Number of dentists by rurality-urbanicity & participation in public insurance programs

Rurality-Urbanicity	Medicaid Only	CHIP Only	Medicaid and CHIP	No Medicaid or CHIP
Urban	201	85	480	1302
Suburban	26	4	47	91
Rural	11	4	50	43

Number of dentists by provider taxonomy & participation in public insurance programs

Provider Type	Medicaid Only	CHIP Only	Medicaid and CHIP	No Medicaid or CHIP
General	203	83	410	1235
Pediatric	9	0	86	11
Specialist	26	9	80	190

©2022 Serban N et al. *JAMA Network Open*.

Number of dentists

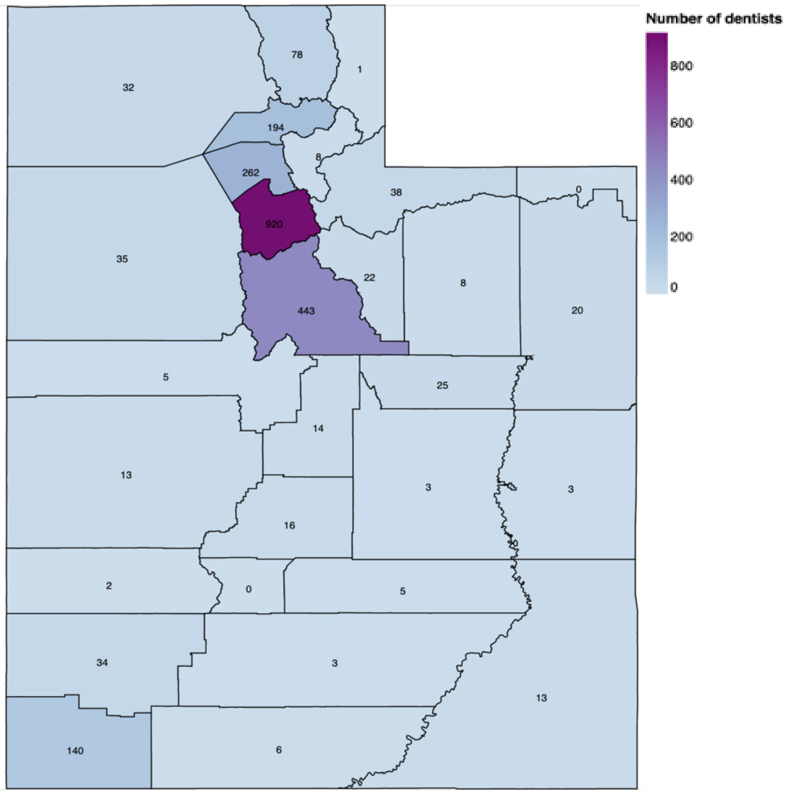

Number of dental hygienists

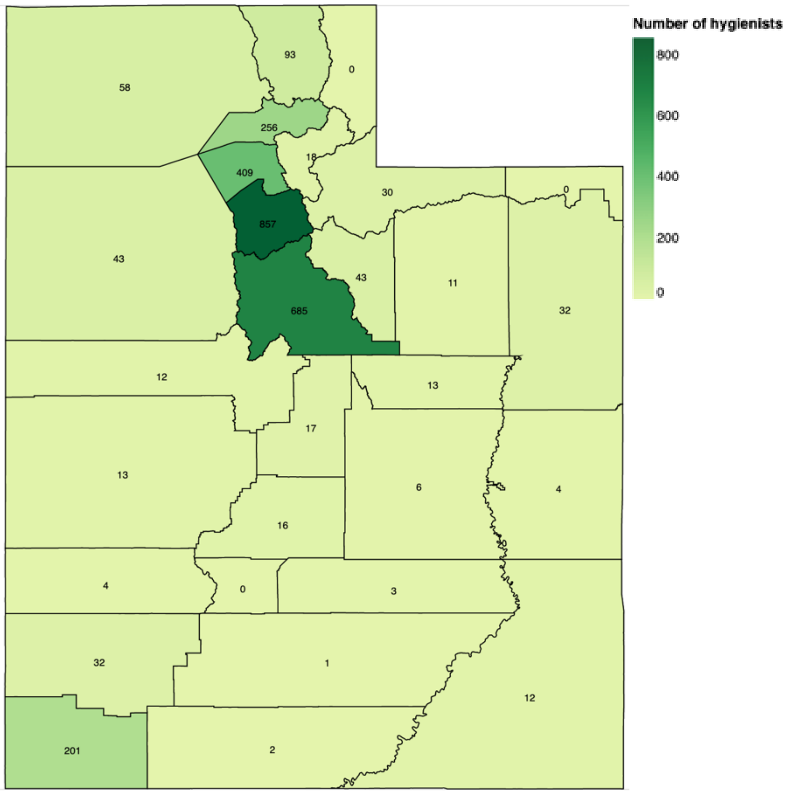

Number of dentists participating in Medicaid

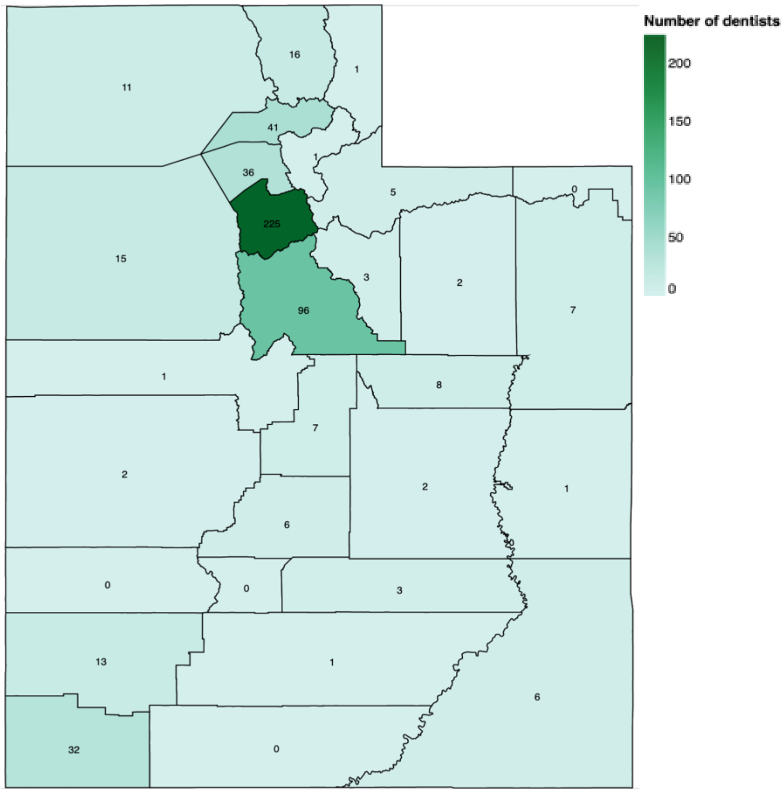

Number of dentists participating in CHIP

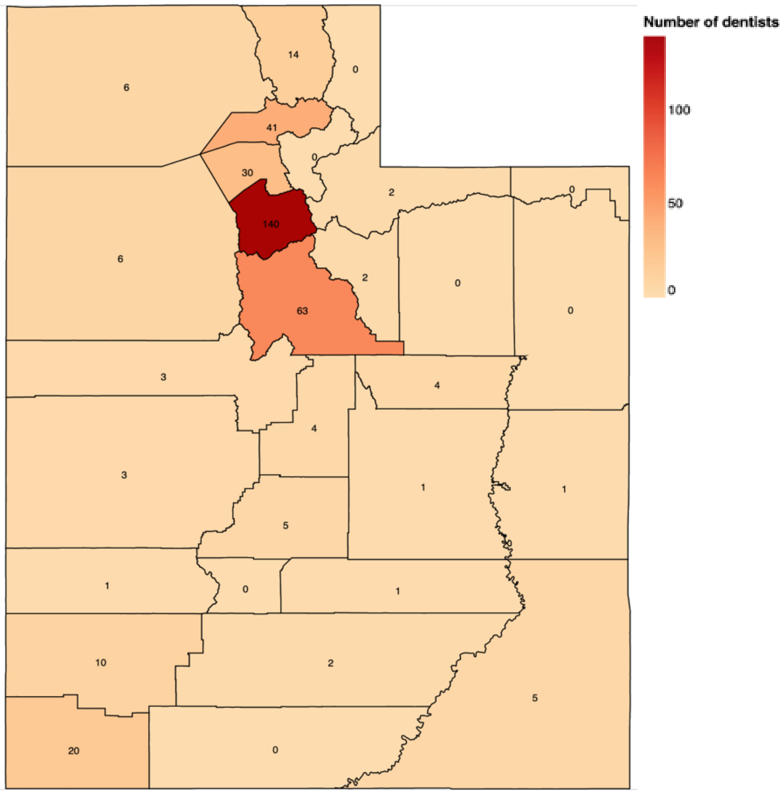

©2022 Serban N et al. *JAMA Network Open*.

Dental Care Supply
VERMONT

This state report summarizes data on the dental care supply, differentiated by type of insurance program, provider taxonomy, and rurality-urbanicity of practice address.

Percentage of dentists by provider taxonomy

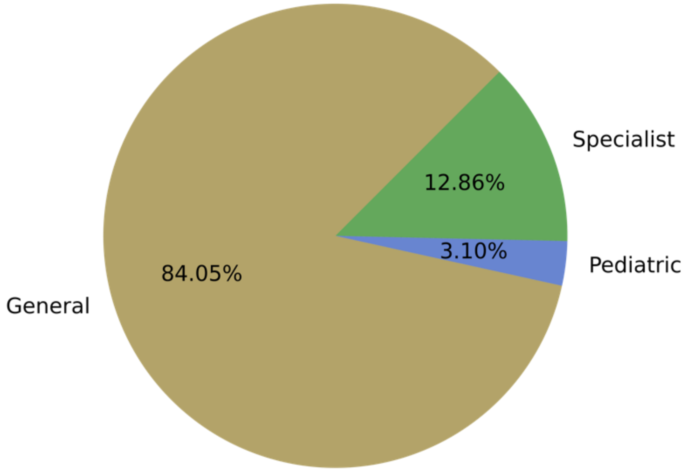

Number of dentists by rurality-urbanicity & provider taxonomy

Rurality-Urbanicity	General	Pediatric	Specialist
**Urban**	126	8	23
**Suburban**	73	1	19
**Rural**	154	4	12

Percentage of dentists by participation in public insurance programs

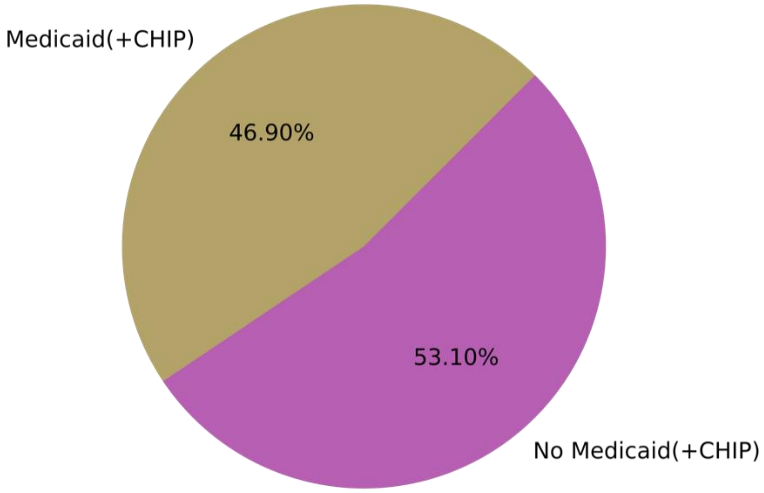

Number of dentists by rurality-urbanicity & participation in public insurance programs

Rurality-Urbanicity	Medicaid(+CHIP)	No Medicaid(+CHIP)
**Urban**	68	88
**Suburban**	42	51
**Rural**	86	84

Number of dentists by provider taxonomy & participation in public insurance programs

Provider Type	Medicaid(+CHIP)	No Medicaid(+CHIP)
**General**	157	196
**Pediatric**	10	3
**Specialist**	30	24

©2022 Serban N et al. *JAMA Network Open*.

Number of dentists

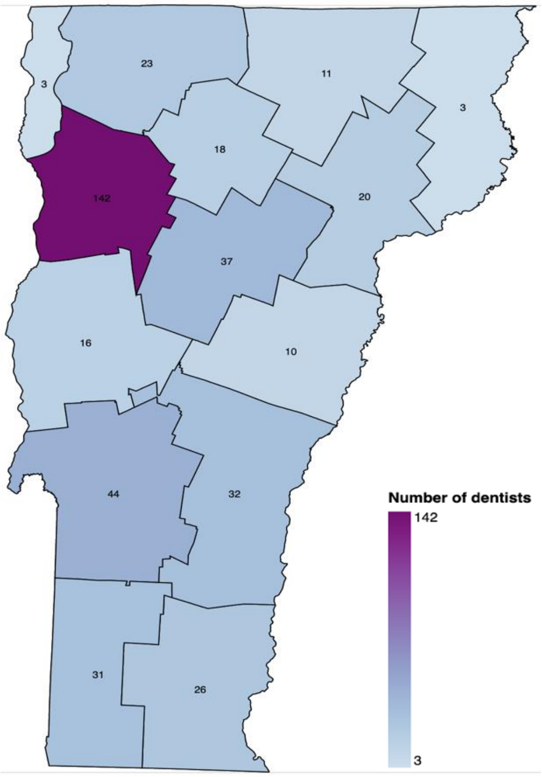

Number of dental hygienists

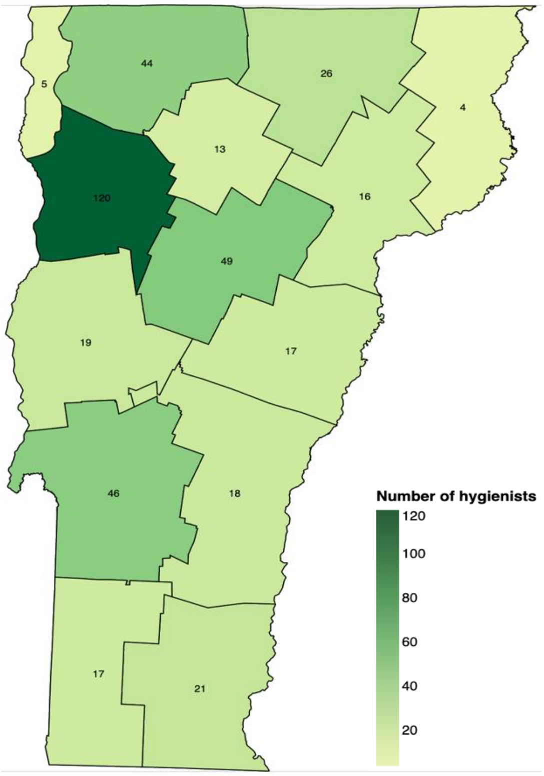

Number of dentists participating in Medicaid(+CHIP)

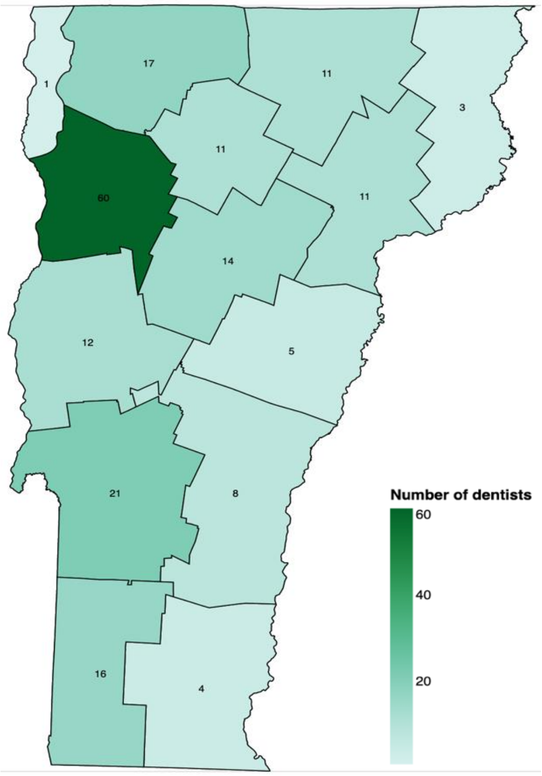

©2022 Serban N et al. *JAMA Network Open*.

Dental Care Supply
VIRGINIA

This state report summarizes data on the dental care supply, differentiated by type of insurance program, provider taxonomy, and rurality-urbanity of practice address.

Percentage of dentists by provider taxonomy

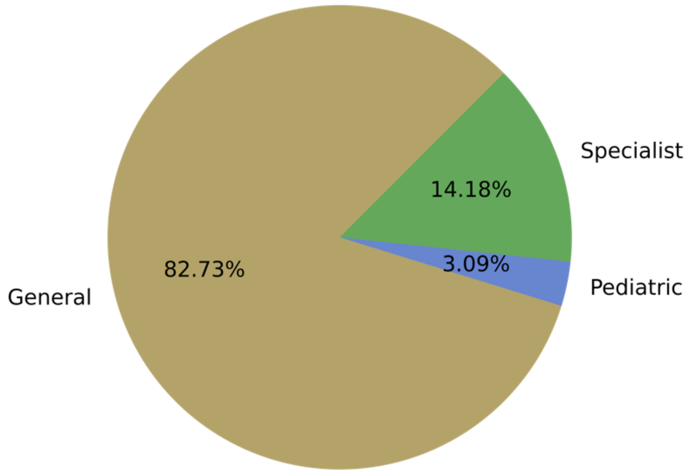

Number of dentists by rurality-urbanity & provider taxonomy

Rurality-Urbanity	General	Pediatric	Specialist
Urban	4386	172	780
Suburban	127	2	15
Rural	224	3	16

Percentage of dentists by participation in public insurance programs

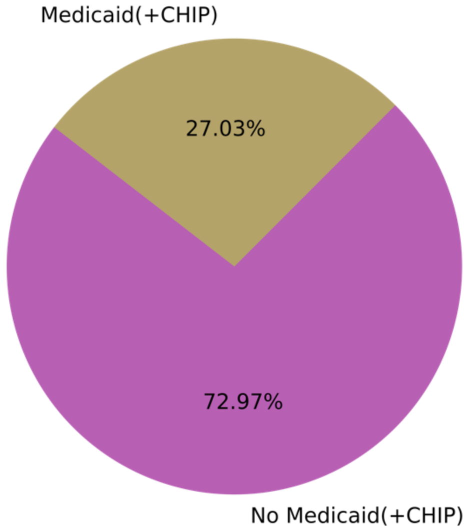

Number of dentists by rurality-urbanity & participation in public insurance programs

Rurality-Urbanity	Medicaid(+CHIP)	No Medicaid(+CHIP)
Urban	1401	3937
Suburban	49	95
Rural	98	146

Number of dentists by provider taxonomy & participation in public insurance programs

Provider Type	Medicaid(+CHIP)	No Medicaid(+CHIP)
General	1171	3566
Pediatric	131	46
Specialist	246	566

©2022 Serban N et al. *JAMA Network Open*.

Number of dentists

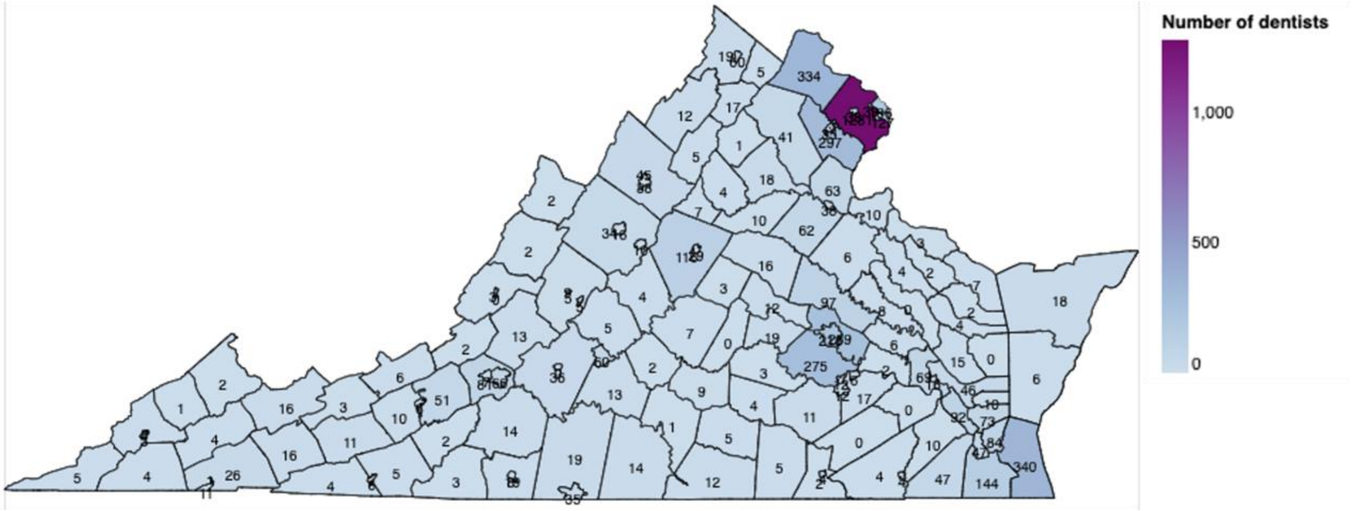

Number of dental hygienists

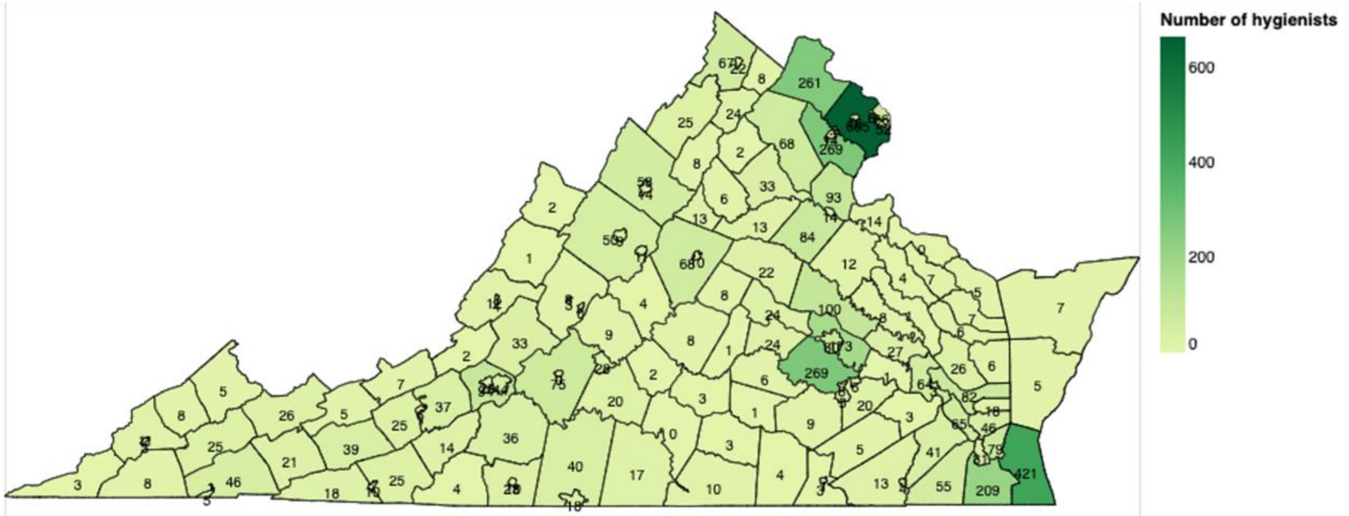

Number of dentists participating in Medicaid(+CHIP)

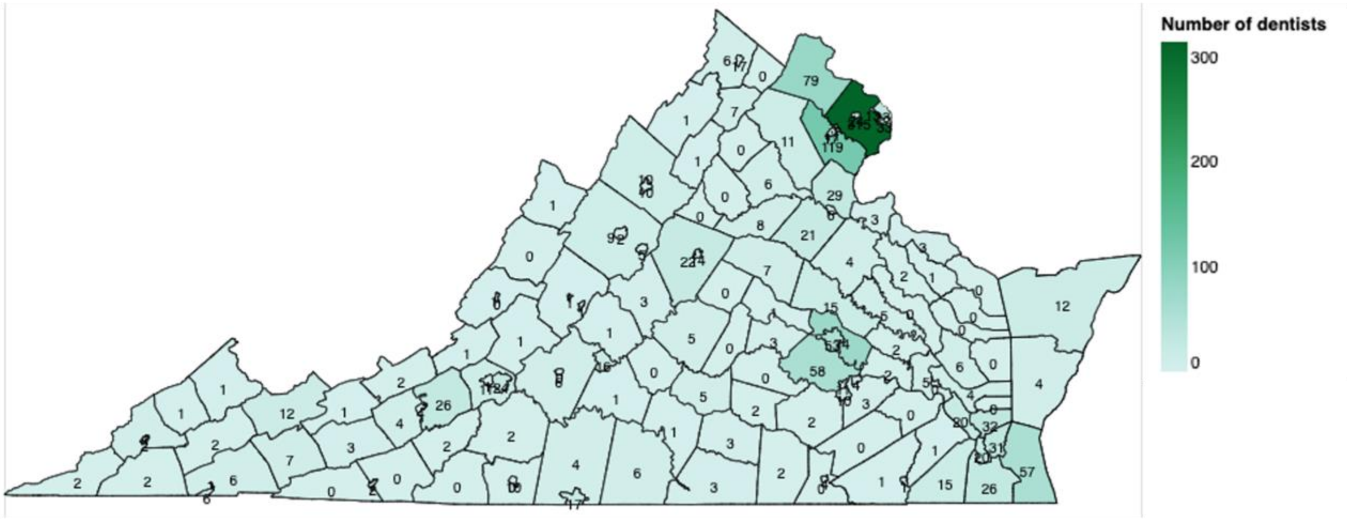

©2022 Serban N et al. *JAMA Network Open*.

Dental Care Supply
WEST VIRGINIA

This state report summarizes data on the dental care supply, differentiated by type of insurance program, provider taxonomy, and rurality-urbanicity of practice address.

Percentage of dentists by provider taxonomy

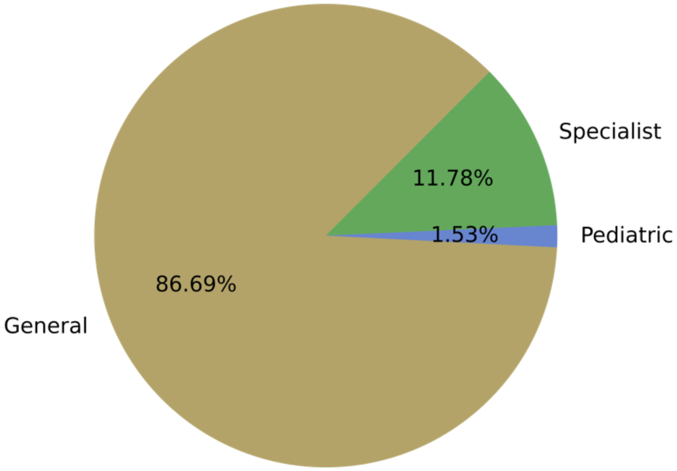

Number of dentists by rurality-urbanicity & provider taxonomy

Rurality-Urbanicity	General	Pediatric	Specialist
Urban	542	12	90
Suburban	91	0	8
Rural	103	1	3

Percentage of dentists by participation in public insurance programs

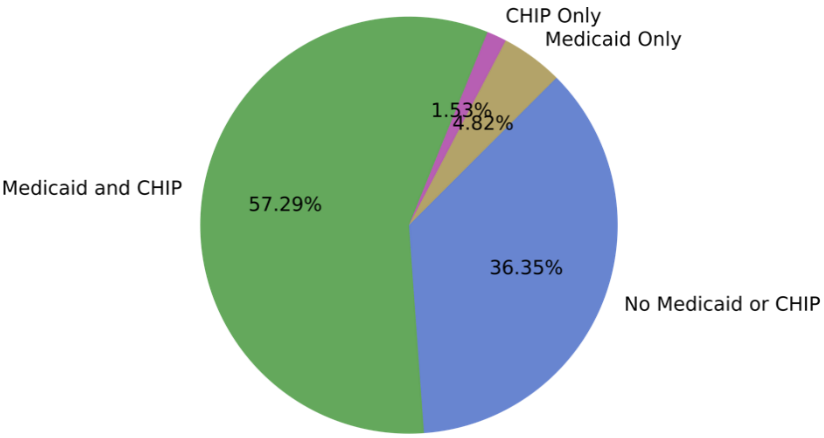

Number of dentists by rurality-urbanicity & participation in public insurance programs

Rurality-Urbanicity	Medicaid Only	CHIP Only	Medicaid and CHIP	No Medicaid or CHIP
Urban	34	10	349	251
Suburban	4	1	59	35
Rural	3	2	79	23

Number of dentists by provider taxonomy & participation in public insurance programs

Provider Type	Medicaid Only	CHIP Only	Medicaid and CHIP	No Medicaid or CHIP
General	40	12	426	258
Pediatric	0	0	11	2
Specialist	1	1	50	49

©2022 Serban N et al. *JAMA Network Open*.

Number of dentists

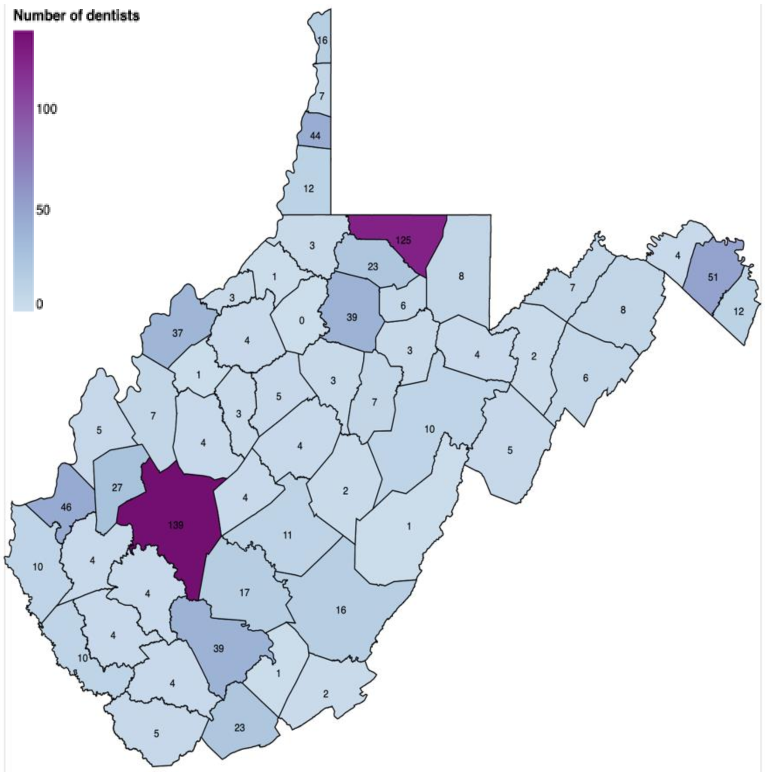

Number of dental hygienists

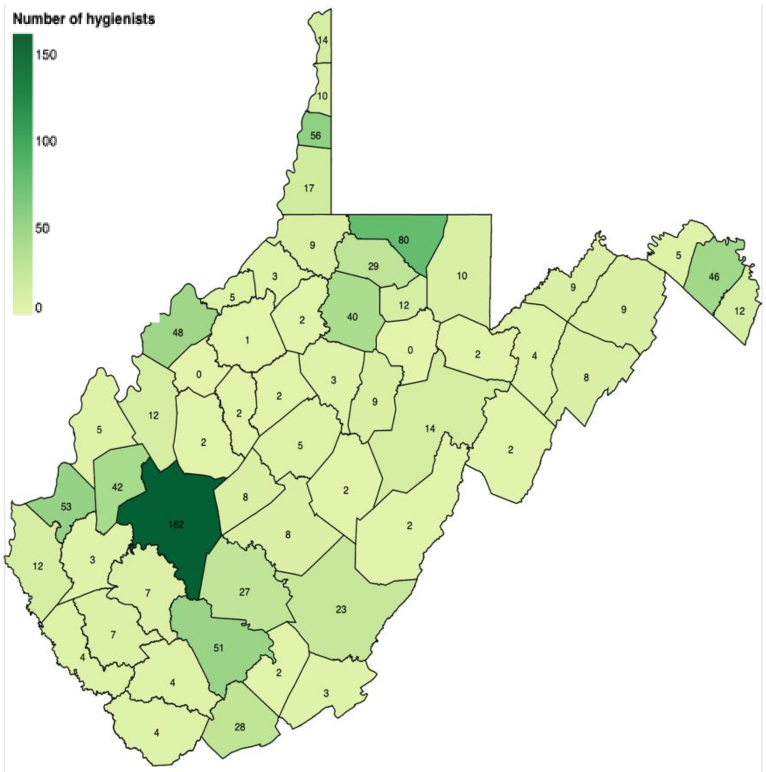

Number of dentists participating in Medicaid

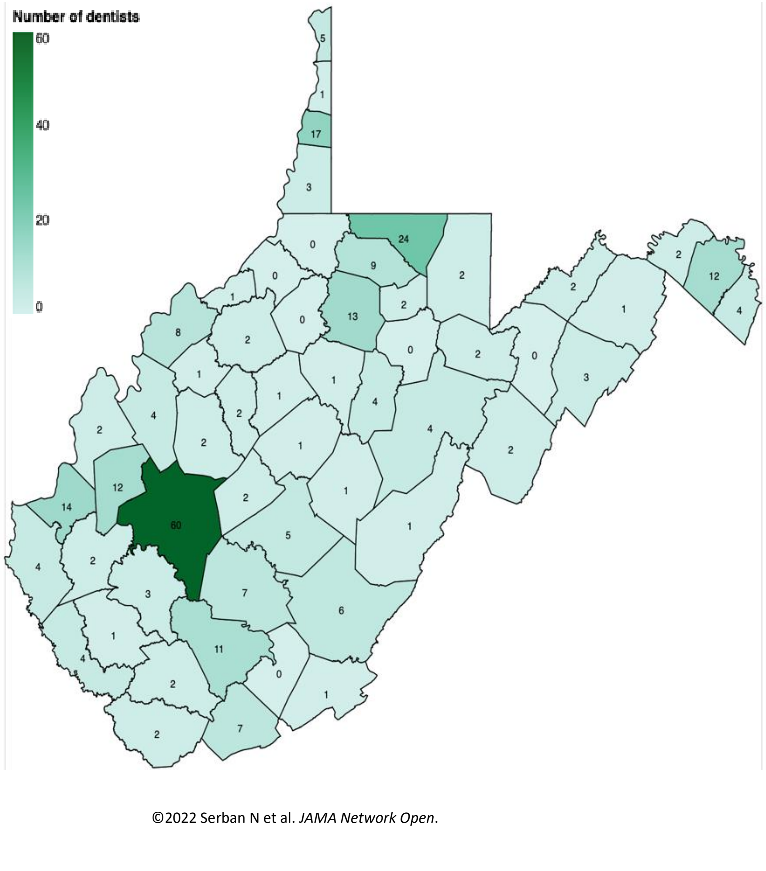

Number of dentists participating in CHIP

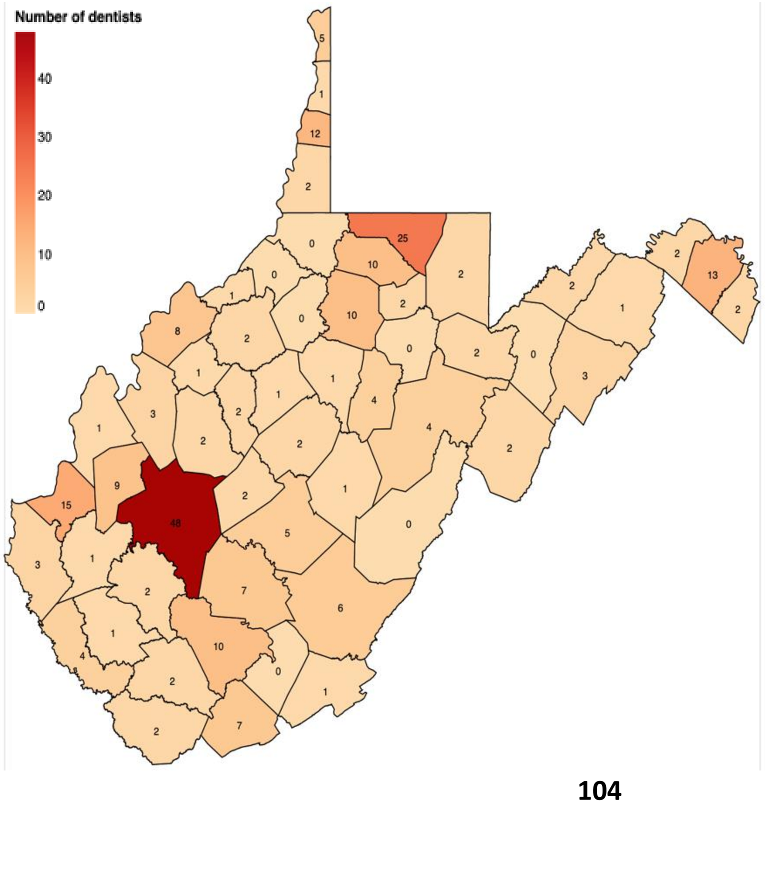

©2022 Serban N et al. *JAMA Network Open*.

Dental Care Supply
WISCONSIN

This state report summarizes data on the dental care supply, differentiated by type of insurance program, provider taxonomy, and rurality-urbanicity of practice address.

Percentage of dentists by provider taxonomy

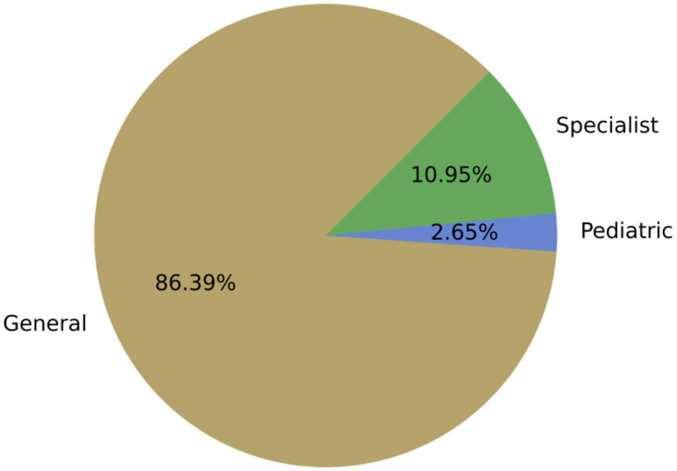

Number of dentists by rurality-urbanicity & provider taxonomy

Rurality-Urbanicity	General	Pediatric	Specialist
Urban	2453	87	363
Suburban	314	5	25
Rural	490	8	24

Percentage of dentists by participation in public insurance programs

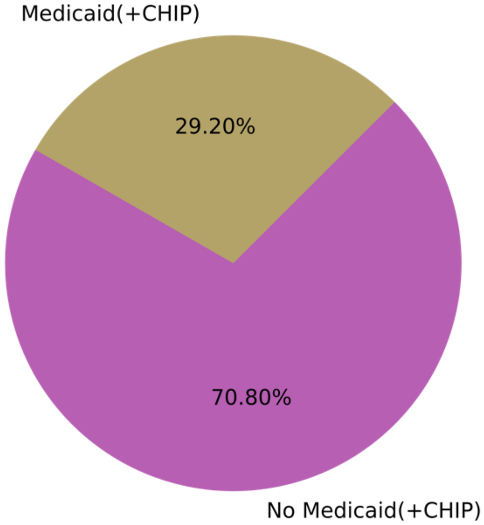

Number of dentists by rurality-urbanicity & participation in public insurance programs

Rurality-Urbanicity	Medicaid(+CHIP)	No Medicaid(+CHIP)
Urban	794	2109
Suburban	104	240
Rural	203	320

Number of dentists by provider taxonomy & participation in public insurance programs

Provider Type	Medicaid(+CHIP)	No Medicaid(+CHIP)
General	914	2343
Pediatric	70	30
Specialist	117	296

©2022 Serban N et al. *JAMA Network Open*.

Number of dentists

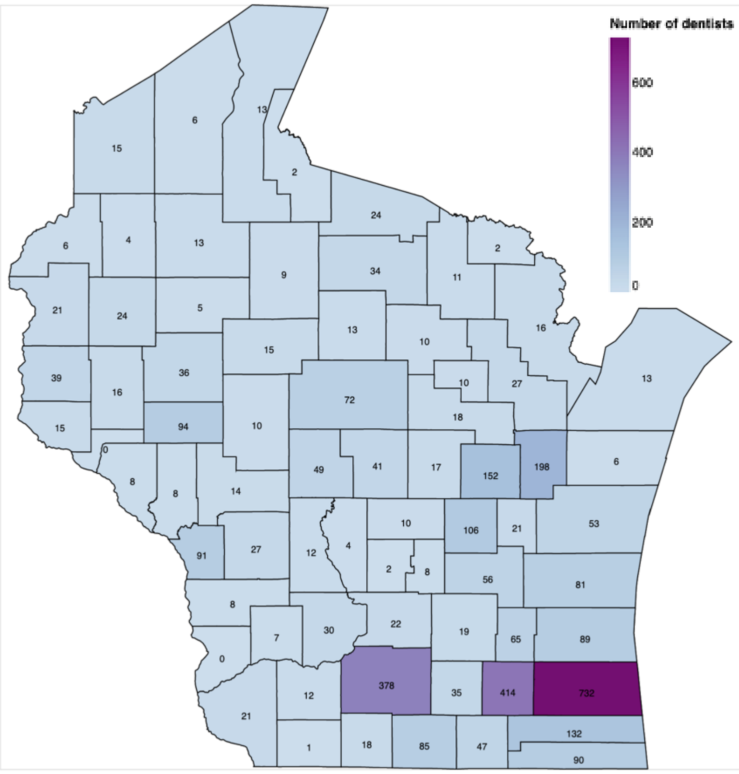

Number of dental hygienists

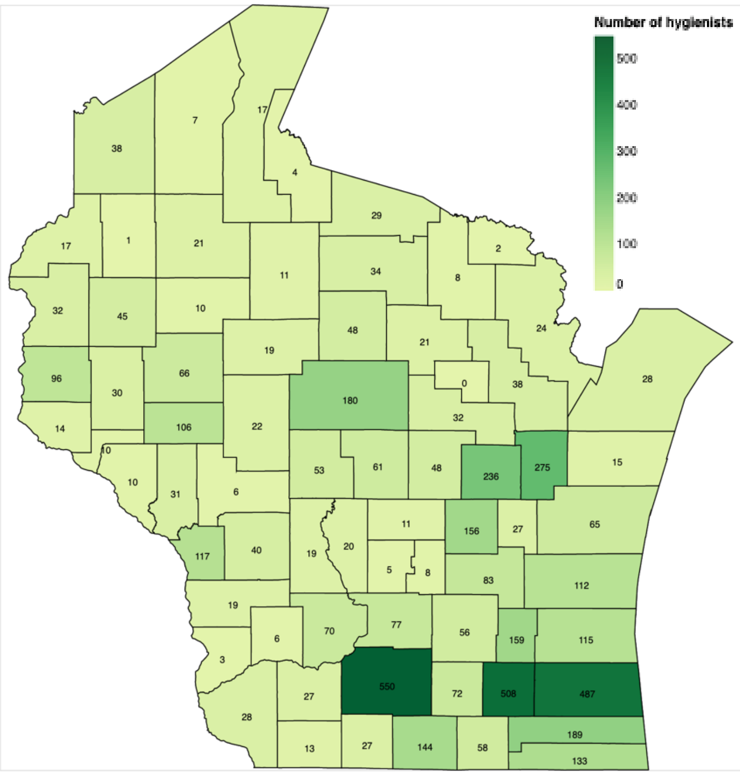

Number of dentists participating in Medicaid(+CHIP)

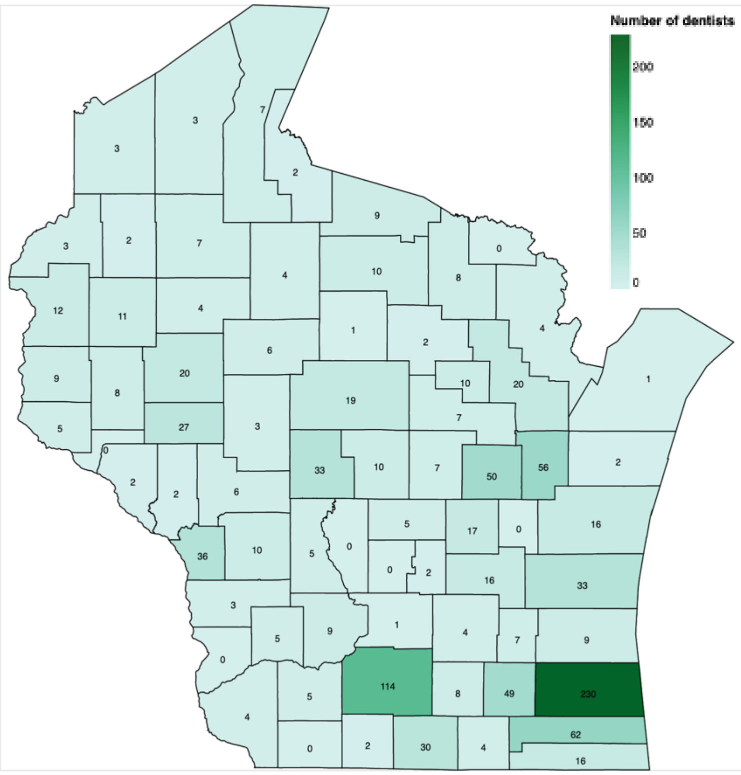

©2022 Serban N et al. *JAMA Network Open*.

Dental Care Supply
WYOMING

This state report summarizes data on the dental care supply, differentiated by type of insurance program, provider taxonomy, and rurality-urbanicity of practice address.

Percentage of dentists by provider taxonomy

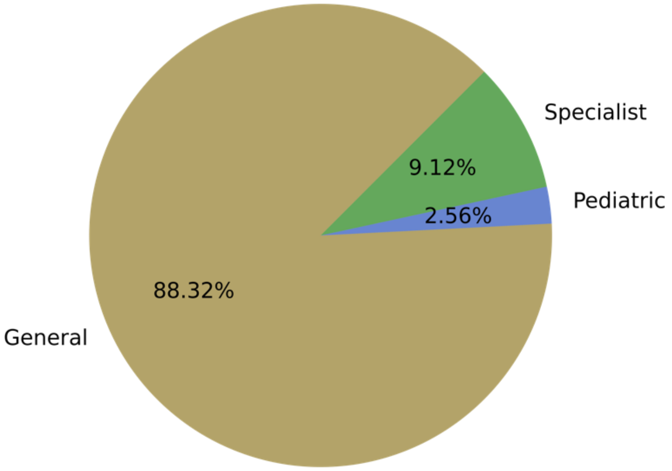

Number of dentists by rurality-urbanicity & provider taxonomy

Rurality-Urbanicity	General	Pediatric	Specialist
Urban	99	3	8
Suburban	120	4	10
Rural	91	2	14

Percentage of dentists by participation in public insurance programs

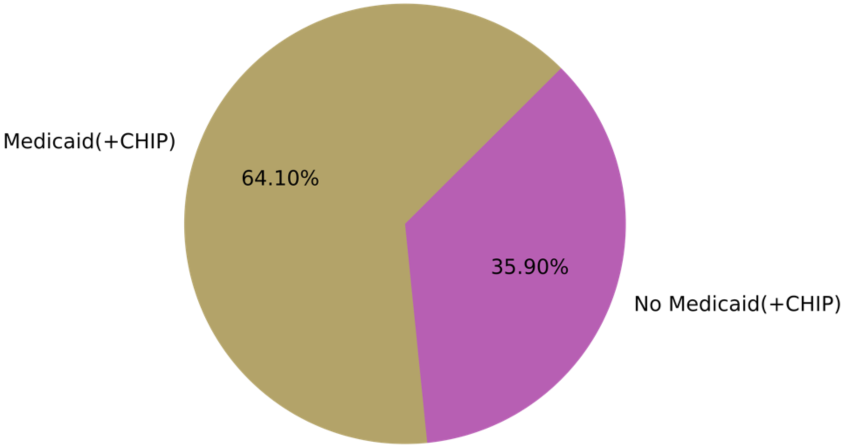

Number of dentists by rurality-urbanicity & participation in public insurance programs

Rurality-Urbanicity	Medicaid(+CHIP)	No Medicaid(+CHIP)
Urban	75	35
Suburban	86	48
Rural	64	43

Number of dentists by provider taxonomy & participation in public insurance programs

Provider Type	Medicaid(+CHIP)	No Medicaid(+CHIP)
General	204	106
Pediatric	8	1
Specialist	13	19

©2022 Serban N et al. *JAMA Network Open*.

Number of dentists

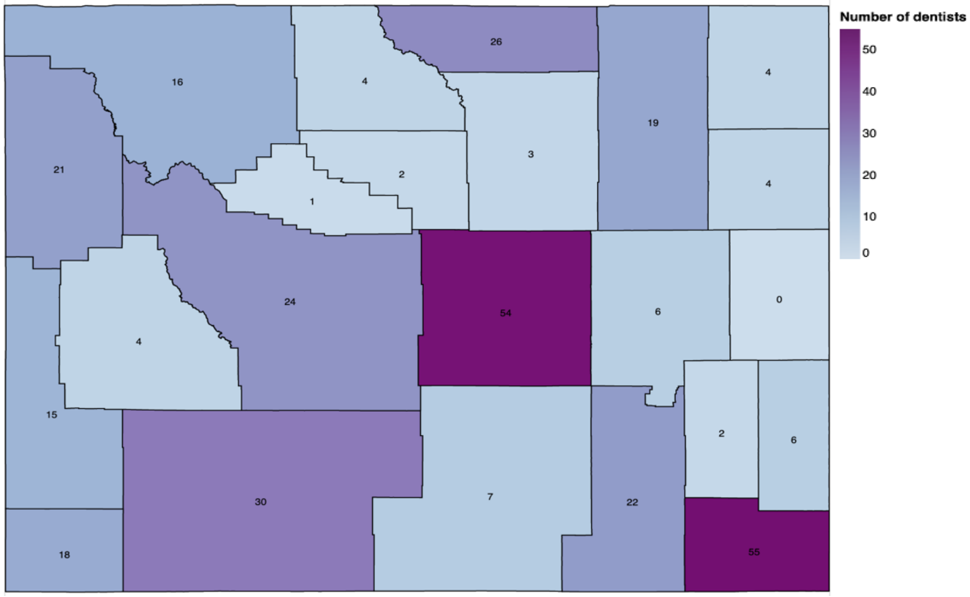

Number of dental hygienists

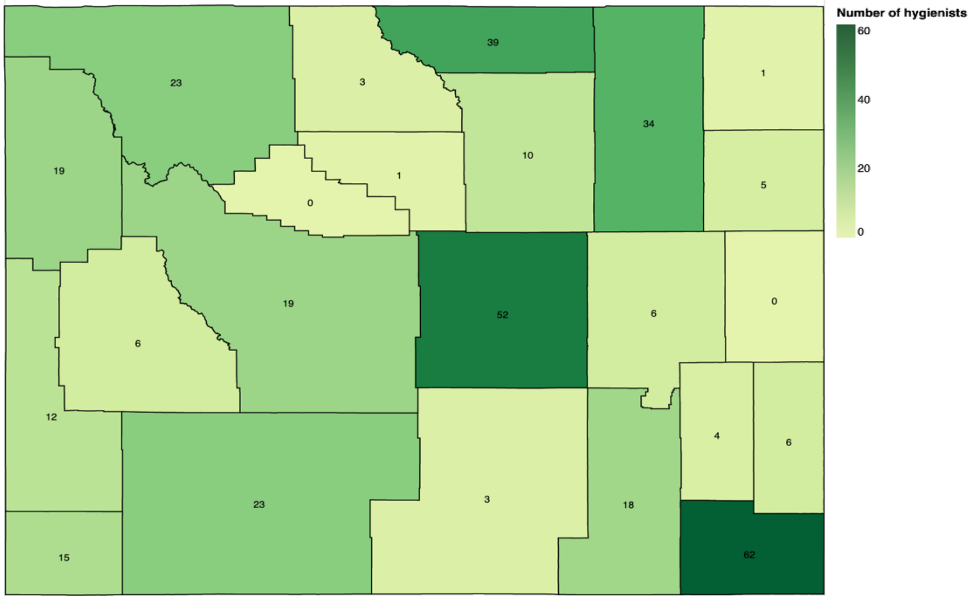

Number of dentists participating in Medicaid(+CHIP)

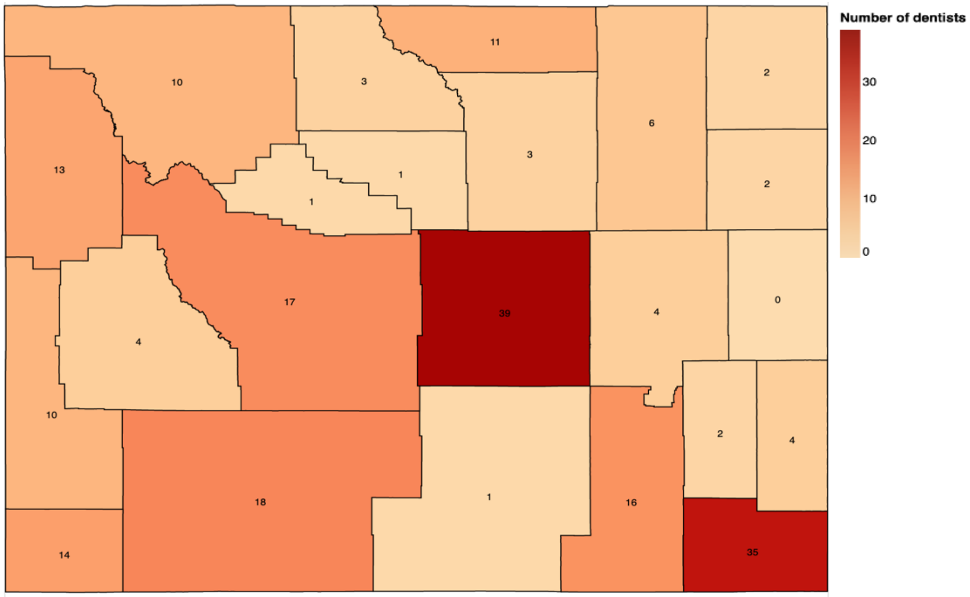

©2022 Serban N et al. *JAMA Network Open*.
